# Number and Position of Methoxy Substituents Govern Photophysics and Visible‐Light Photocatalysis in Earth‐Abundant Cu(I) Complexes

**DOI:** 10.1002/cssc.70931

**Published:** 2026-08-12

**Authors:** Kurt J. Haseloff, Max Wolf, Katharina Rediger, Christian Kleeberg, Maria Wächtler, Stefanie Tschierlei, Michael Karnahl

**Affiliations:** ^1^ Department of Energy Conversion Institute of Physical and Theoretical Chemistry Technische Universität Braunschweig Braunschweig Germany; ^2^ Department of Chemistry Rheinland‐Pfälzische Technische Universität Kaiserslautern‐Landau Kaiserslautern Germany; ^3^ Institute of Inorganic and Analytical Chemistry Technische Universität Braunschweig Braunschweig Germany; ^4^ Institute of Physical Chemistry and Kiel Nano, Surface and Interface Science (KiNSIS) Christian‐Albrechts‐Universität zu Kiel Kiel Germany

**Keywords:** copper(I) photosensitizers, C–H functionalizations, methoxy substitution, singlet oxygen, structure–property relationships, visible‐light photoredox catalysis

## Abstract

Earth‐abundant Cu(I) photosensitizers are promising systems for visible‐light‐driven photocatalysis, but further design guidelines are required to relate targeted ligand modification to catalytic performance. Herein, we present five new heteroleptic Cu(I) complexes with two or three methoxy substituents at the bathocuproine backbone and two single‐crystal structures to assess how substitution number and position affect structure, photophysics, redox properties, and photocatalytic performance. While all complexes retain the expected distorted tetrahedral Cu(I) coordination motif, their properties depend strongly on the substitution pattern. In particular, *ortho*/*para* substitution is much more effective than *meta* substitution in prolonging the excited‐state lifetimes (up to 1.46 µs) and emission quantum yields (up to 8.1%). Benchmark studies in energy‐transfer and electron‐transfer photocatalysis reveal that there is no single optimal substitution pattern for all reactions. *Ortho*/*para* substitution appears to be advantageous for excited‐state performance and triplet sensitization, whereas *meta* substitution benefits from higher excited‐state potentials, highlighting that the catalytic performance of these complexes depends strongly on the specific substrate and reaction mechanism. Overall, these results demonstrate that the position of the methoxy substituents is more decisive than their number and establish application‐specific methoxy substitution as a useful strategy for tailoring noble‐metal‐free Cu(I) photosensitizers toward photocatalytic applications.

## Graphical abstract

The number and position of methoxy substituents provide a versatile molecular toolbox for tuning the photophysics and visible‐light photocatalytic performance of heteroleptic Cu(I) photosensitizers.

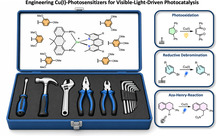

## 1 Introduction

Photoredox catalysis has emerged as a powerful strategy in modern synthetic chemistry, exploiting visible‐light‐driven single‐electron‐transfer (SET) and energy‐transfer (EnT) events to generate radical or excited‐state intermediates [1, 2, 3, 4, 5, 6]. By harnessing the abundant energy of visible light, these processes enable the activation of strong C—X bonds (e.g., C—H, C—C, C—N, and C—O) under remarkably mild conditions, often with exceptional tolerance toward sensitive functional groups [7, 8, 9, 10]. Such reactivity has unlocked new approaches to the assembly of complex molecules and offered more sustainable alternatives to thermally driven radical pathways [1, 11, 12]. Central to the design of effective photoredox catalysts is a set of interrelated photophysical and electrochemical parameters [13]. First, efficient absorption in the visible region ensures maximal overlap with common light sources such as blue or green LEDs [13, 14, 15]. Second, a sufficiently long‐lived excited state prolongs the window for electron‐ or EnT events [13, 16], while the nature of the excited state, for example, whether it is a metal‐to‐ligand charge transfer (MLCT) or a metal‐centered (d–d) transition, strongly influences the reactivity and selectivity of the subsequent photochemical step [17, 18, 19, 20]. Third, the redox potentials of both the ground and excited states must match the substrate oxidation or reduction requirements [21, 22, 23, 24].

In homogeneous photoredox catalysis, Ru(II) and Ir(III) polypyridine complexes have historically dominated the field [1, 25, 26, 27, 28]. Their enduring popularity stems from long‐lived MLCT states, often in the hundreds of nanoseconds regime, and broad, tunable redox windows that accommodate a wide variety of substrates [27, 29]. However, the high cost, limited availability, and environmental impact of these 4d and 5d metals have motivated the shift toward earth‐abundant alternatives [30, 31, 32, 33, 34]. Coordination complexes including first‐row transition metals such as Cu [35, 36, 37, 38, 39], Fe [31, 40, 41, 42], Co [43, 44, 45], Ni [17, 46, 47], Cr [48, 49, 50, 51], and Mn [18, 52] present lower‐cost and more sustainable options. At the same time, many first‐row systems still face important challenges, including low‐lying metal‐centered excited states that promote nonradiative deactivation, higher sensitivity toward ligand exchange or structural distortion, and, depending on the metal center and ligand environment, less favorable excited‐state properties than their Ru and Ir analogs [30, 31, 49, 51, 53].

Within this context, Cu(I) photosensitizers have emerged as particularly promising candidates [39, 54, 55]. Tetrahedral diimine complexes of Cu(I) can access strongly reducing or oxidizing MLCT states, enabling both reductive and oxidative quenching cycles [56, 57, 58]. A key design principle is the incorporation of sterically bulky, chelating ligands that inhibit excited‐state geometry changes, in particular the flattening distortion toward a more square‐planar conformation that accelerates nonradiative decay [38, 59, 60, 61]. Nevertheless, the photochemical performance of Cu(I) complexes remains highly sensitive to ligand structures. Heteroleptic diimine‐diphosphine frameworks have been established as a robust platform, combining π‐accepting diimines with σ‐donating phosphines to modulate the MLCT character and to stabilize long‐lived excited states [55, 56, 57, 62]. In this family of complexes, ligand design has mainly focused on three complementary strategies: steric protection of the Cu(I) center, extension of the diimine π‐system, and electronic tuning by substituent effects [63, 64, 65, 66, 67]. For example, steric modification at the 2,9‐positions of phenanthroline efficiently suppresses excited‐state distortion, whereas π‐extension and electronic substituents can be used to adjust absorption, excited‐state energies, emission behavior, and redox properties [67, 68, 69, 70, 71]. These studies collectively underscore the delicate balance between ligand rigidity, electronic tuning, and coordination geometry required to achieve efficient and long‐lived Cu(I) photosensitizers.

Among these approaches, methoxy substitution has proven to be especially useful for systematically tuning the electronic structure and excited‐state properties of heteroleptic Cu(I) complexes [72, 73, 74, 75]. Doettinger and coworkers demonstrated that Cu(I) complexes bearing electron‐donating methoxy (–OMe) or electron‐withdrawing trifluoromethyl (–CF_3_) groups exhibit pronounced differences in their photophysical and redox behavior [72, 73]. In particular, CF_3_ substitution affords the most positive excited‐state reduction potential (*E* ^*red^ = 0.58 V), enabling photocatalytic applications including singlet‐oxygen generation and reductive dehalogenation [73]. In a previous study, the direct impact of electron‐donating methoxy groups in different positions at the bathocuproine backbone was compared [75]. For this purpose, a single methoxy group was introduced at the *ortho*‐ (**Cu2**), *meta*‐ (**Cu3**), and *para*‐positions (**Cu4**). The resulting Cu(I) complexes exhibited clear differences in both their photophysical and photocatalytic behavior [75]. For instance, *ortho*‐ and *para*‐substitution (**Cu2**, **Cu4**) led to longer triplet excited‐state lifetimes and more cathodic ground‐state reduction potentials (*E* ^red,1/2^ **Cu2** = −2.11 V, **Cu4** = −2.08 V), which resulted in a lower excited‐state reduction potential (*E* ^*red^ **Cu2** = 0.46 V, **Cu4** = 0.48 V), compared to the *meta*‐ or unsubstituted complexes **Cu3** and **CuRef** [75].

**FIGURE 1:**
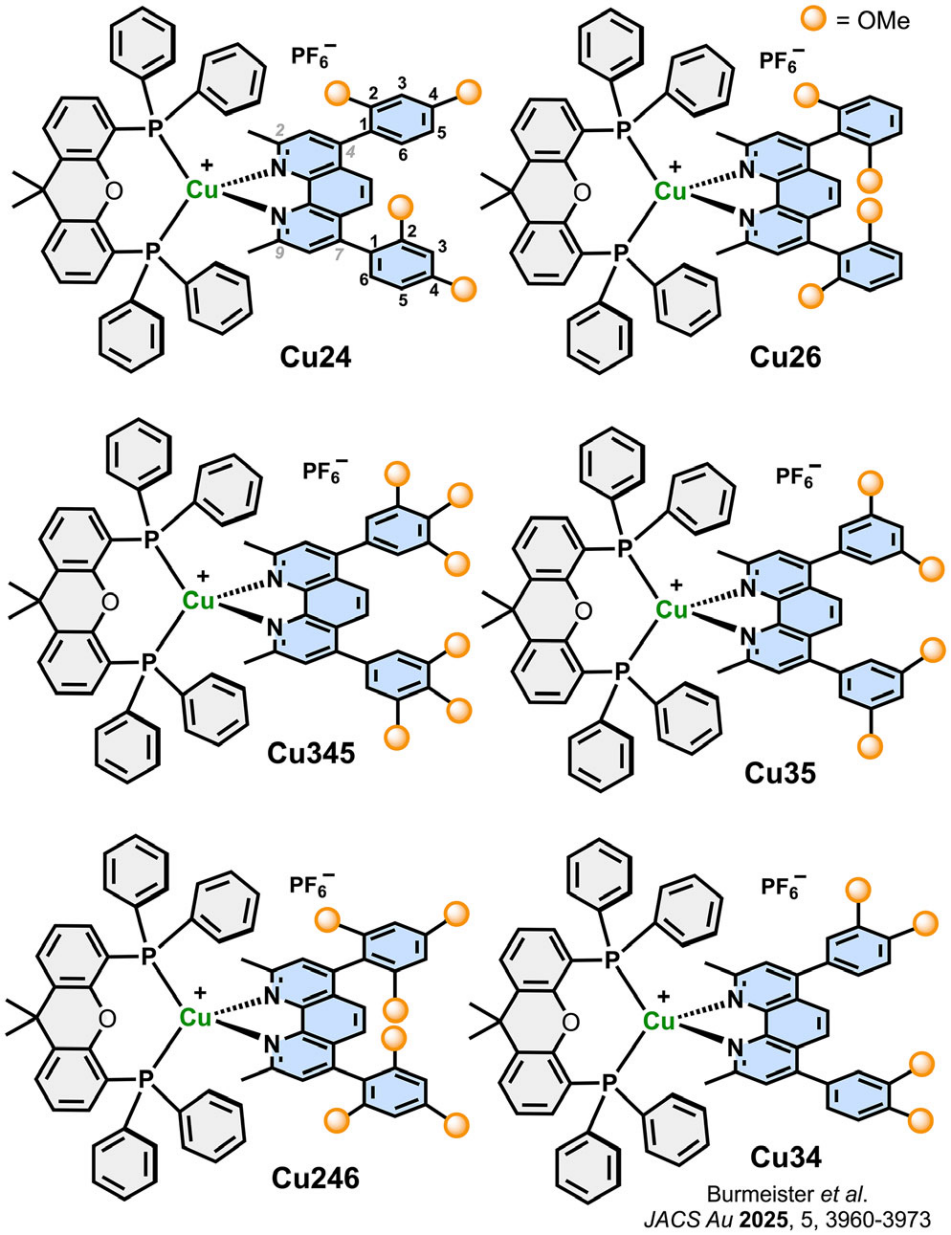
Overview of the Cu(I) complexes investigated in this study. The orange spheres highlight the methoxy substituents.

Herein, we extend this methoxy‐substitution concept from mono‐ to poly‐substituted systems and present five new heteroleptic Cu(I) complexes bearing two or three methoxy groups at the phenyl rings of the bathocuproine backbone (**Cu24**, **Cu26**, **Cu35**, **Cu345**, **Cu246,** cf. Figure 1). The complexes were investigated with regard to their photophysical and electrochemical behavior in solution and evaluated in a series of EnT and electron‐transfer benchmark reactions, including singlet oxygen (^1^O_2_) generation, photooxidation of 2,5‐diphenylfuran (DPF), visible‐light‐driven C—Br bond activation, and C—H functionalization. This approach allows us to identify how the number and positional arrangement of methoxy substituents influence distinct catalytic requirements and thereby establish practical structure–property–activity relationships for earth‐abundant Cu(I) photosensitizers.

## 2 Results and Discussion

### 2.1 Synthesis of the Cu(I) Complexes

**SCHEME 1:**
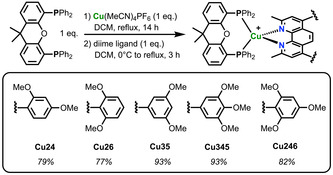
Synthetic route to the heteroleptic Cu(I) complexes via a two‐step, one‐pot procedure.

The methoxy‐substituted bathocuproine derivatives were synthesized via the Suzuki–Miyaura cross‐coupling reaction according to previously reported procedures [72, 73, 76]. The ligands were obtained as white/beige powders in yields ranging from 57% to 82% (see Supporting Information for synthetic details). Subsequently, the heteroleptic Cu(I) complexes were prepared using a literature‐known two‐step, one‐pot protocol (Scheme 1) [63, 65, 72]. Under an argon atmosphere, a dichloromethane solution of [Cu(MeCN)_4_]PF_6_ (1.0 equiv., MeCN = acetonitrile) and xantphos (1.0 equiv., 4,5‐bis(diphenylphosphino)‐9,9‐dimethylxanthene) was stirred at 45 °C for 14 h to generate the corresponding [Cu(MeCN)_2_(xantphos)]PF_6_ adduct [73]. The mixture was then cooled to 0 °C, and the respective diimine ligand was added slowly dropwise using a syringe pump (12 mL/h) to suppress the formation of homoleptic byproducts [37, 69, 77]. After an additional 30 min at 0 °C, the reaction was reheated to 45 °C and stirred for 3 h. The resulting yellow solutions were treated with *n*‐hexane to induce precipitation of the products.

All final compounds were isolated as yellow to brown crystalline solids in yields reaching 77% to 93% (Scheme 1). Furthermore, the ligands and Cu complexes were fully characterized by nuclear magnetic resonance spectroscopy (NMR, ^1^H, ^13^C{^1^H}, ^31^P{^1^H}), electrospray ionization high‐resolution mass spectrometry (ESI‐HRMS) and elemental analysis (EA). The obtained analytical data were fully consistent with the proposed structures and confirmed the selective formation of the target heteroleptic Cu(I) complexes. Full experimental details and characterization data are provided in the Supporting Information. In view of the known tendency of heteroleptic Cu(I) diimine‐diphosphine complexes to undergo partial ligand redistribution in solution [69, 77, 78], freshly synthesized and purified batches were used for the photophysical and catalytic measurements. In addition, single crystals of **Cu35** and **Cu345**, suitable for X‐ray diffraction (XRD) analysis, were grown by slow diffusion of *n*‐hexane into a saturated dichloromethane solution.

### 2.2 Structural Characterization

Single crystals suited for XRD analysis were obtained as [PF_6_]‐salts for **Cu35** and **Cu345**, while the solid‐state structure of the substituted bathocuproine ligand **L246** was also determined and is provided in Table S4.3. The experimentally determined structures of **Cu35** and **Cu345** were further compared with the corresponding DFT‐optimized geometries using B3LYP (D3‐BJ)/def2‐TZVP level of theory (Table 1).

**TABLE 1:** Selected bond lengths (pm), bite angles (°), interplane angles between the N–Cu–N/P–Cu–P planes (PP–Cu–NN,°), and torsion angles of backbone phenyl rings *α*_Sub1,2_ (1 = outer ring, 2 = front ring,°) for **Cu35** and **Cu345** obtained from X‐ray diffraction studies together with the corresponding DFT‐calculated data (calc.).

	Cu35		Cu345	
	exp.	calc.	exp.	calc.
Cu–N	208.2(16) 210.1(15)	211.6 212.0	210.2(3) 212.4(2)	211.6 212.1
Cu–P	223.7(5) 229.2(6)	226.4 231.1	225.2(9) 232.0(9)	226.4 231.2
N–Cu–N	80.2(6)	79.2	79.6(10)	79.2
P–Cu–P	120.3(2)	118.7	114.7(3)	118.7
Interplane	88.5	86.2	88.3	86.2
*α*_sub,1_	−50.3(3)	−52.9	−44.4(5)	−50.5
*α*_sub,2_	72.63(3)	54.9	−60.4(4)	52.4

**FIGURE 2:**
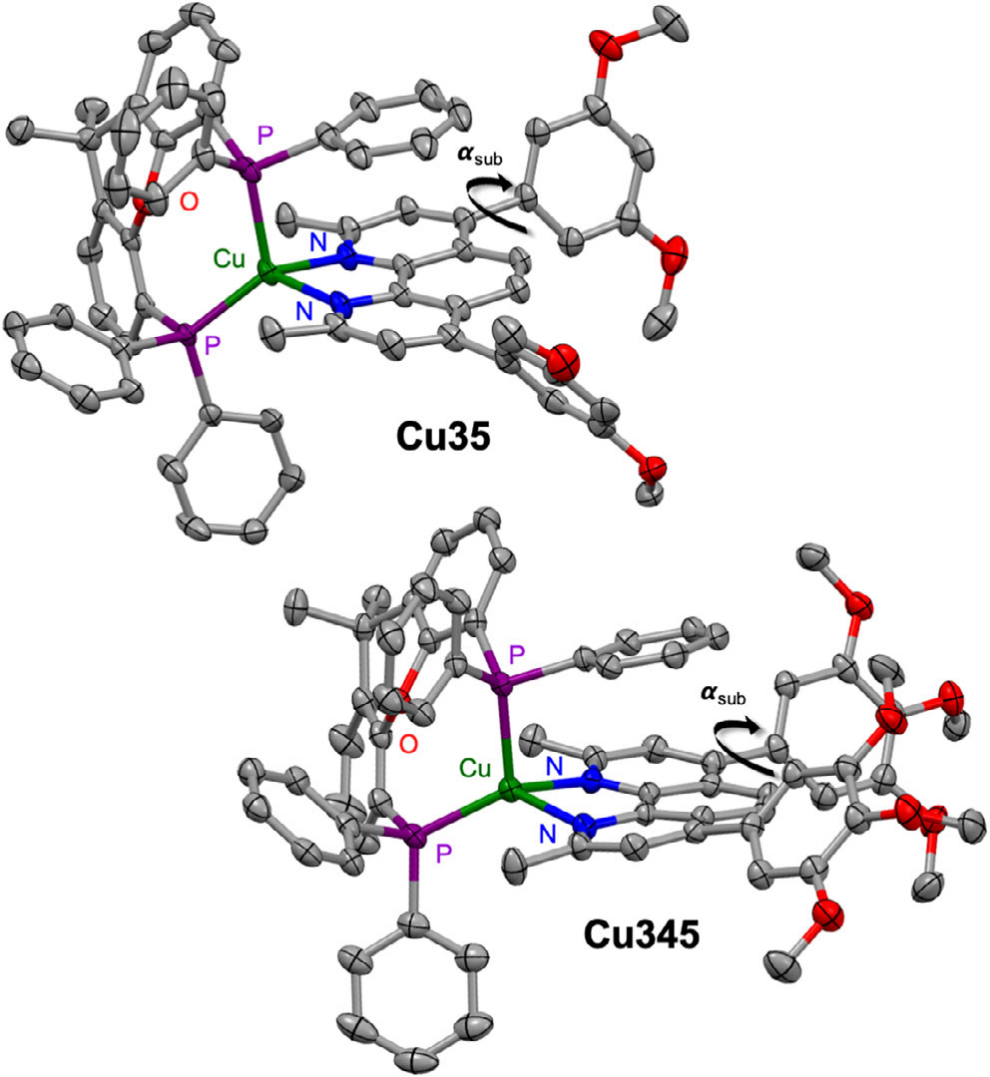
Solid‐state structures of **Cu35** and **Cu345** as determined by single crystal X‐ray diffraction analysis. Thermal ellipsoids are drawn at the 50% probability level. Hydrogen atoms, counter anions, and solvent molecules have been omitted for clarity. Selected bond lengths, angles, and torsion angles are summarized in Table 1.

Both Cu(I) complexes exhibit the characteristic distorted tetrahedral geometry of heteroleptic diimine‐diphosphine photosensitizers (Figure 2) [54, 56, 65, 79], with the distortion arising from the narrow N^N bite (≈80°) of the diimine ligand and the much wider P^P bite (≈115°–120°) of xantphos [64]. In **Cu35**, the Cu–N distances are 208.2(16) and 210.1(15) pm, the Cu–P distances are 223.7(5) and 229.2(6) pm, with N–Cu–N = 80.2(6)° and P–Cu–P = 120.3(2)°, whereas in **Cu345** the corresponding distances are slightly longer (Cu–N 210.2(3)/212.4(2) pm and Cu–P 225.2(9)/232.0(9) pm) and the P–Cu–P angle is smaller (114.7(3)°). The interplane angles between the N–Cu–N and P–Cu–P coordination planes are essentially unchanged (88.47° for **Cu35**, 88.26° for **Cu345**), indicating no unusual ground‐state flattening. Thus, the higher degree of methoxy substitution in **Cu345** exerts only a subtle influence on the local coordination environment around the Cu(I) center. This is reasonable because the methoxy substituents are located on peripheral aryl rings rather than in the immediate coordination sphere of the metal center and therefore exert only a minor steric influence on the Cu(I) ground‐state geometry.

The torsion angles of the backbone phenyl rings further illustrate the steric impact of the substitution pattern. In **Cu35**, torsion angles of −50.3(3)° and +72.6(3)° were determined, whereas **Cu345** shows values of −44.4(5)° and −60.4(4)°. Thus, the aryl substituents are clearly twisted out of the phenanthroline plane in both complexes (Figure 2), indicating limited coplanarity and therefore only partial π‐conjugation between the bathocuproine core and the peripheral phenyl rings. At the same time, the different torsional arrangement of **Cu35** and **Cu345** suggests that increasing methoxy substitution does not merely affect the electronic character of the ligand scaffold, but also its steric balance and conformational preferences. Overall, the experimentally determined structures are well reproduced by the DFT‐optimized geometries. The calculated Cu—N and Cu—P bond lengths are slightly longer than the experimental values (Table 1), but remain in good overall agreement with the X‐ray data. While the calculations reproduce the very similar N–Cu–N bite angles of both complexes well, the experimentally observed difference in the P–Cu–P angle between **Cu35** and **Cu345** is less pronounced in the optimized structures. The largest deviations are found for the torsion angles of the peripheral aryl substituents, which is reasonable given their sensitivity to local packing effects and the possible formation of different rotamers.

### 2.3 Electrochemical Characterization

The electrochemical properties of the newly synthesized Cu(I) complexes **Cu24**, **Cu26**, **Cu35**, **Cu345**, and **Cu246** were investigated by cyclic voltammetry in deaerated acetonitrile (Section S8). All complexes exhibit at least one reversible reduction event, assigned to the diimine‐centered reduction of the phenanthroline moiety, as typically observed for this class of heteroleptic Cu(I) photosensitizers [56, 73, 75]. A second, less prominent reduction wave is observed for some complexes, most clearly for **Cu246** and **Cu345** (Figure S8.1), and can be assigned to a higher‐lying ligand‐centered reduction of the phenanthroline‐based framework. Furthermore, all complexes display an irreversible oxidation wave between 0.85 V and 1.00 V versus Fc/Fc^+^ (Figure S8.1), which is attributed to oxidative decomposition associated with Cu—P bond cleavage, as commonly reported for this class of complexes [65, 77, 78, 80]. Whereas the oxidation potentials vary only slightly across the series, the ground‐state reduction potentials (*E* ^red,1/2^) show much more pronounced differences.

The *ortho*/*para*‐methoxy‐substituted complex **Cu246** exhibits the most negative potential (−2.18 V), followed by the double *ortho*‐substituted **Cu26** (−2.15 V) and the *ortho*/*para* isomer **Cu24** (−2.13 V, Table 2). These reversible reduction potentials are shifted cathodically relative to those of the singly methoxy‐substituted analogs, **Cu2** and **Cu4**, with potentials of −2.11 V and −2.08 V, respectively. This indicates that methoxy substitution at both *ortho* positions induces a cathodic shift in *E* ^red,1/2^. In contrast, the *meta*‐substituted derivatives **Cu345** and **Cu35** have less negative potentials of −2.05 V and −2.03 V, respectively, which closely match those of **Cu3** (−2.03 V) and the unsubstituted reference **CuRef** (−2.04 V). This confirms that a *meta*‐methoxy substitution produces only a negligible shift in the reduction potential. The slightly more negative value for **Cu345** likely reflects the additional influence of the *para* substituent. In addition, the literature‐known **Cu34** fits well into the observed trend with a reduction potential of −2.07 V (Table 2) [81].

**TABLE 2:** Summary of the photophysical and electrochemical properties of the Cu(I) complexes in deaerated acetonitrile at room temperature. Redox potentials, excited state reduction potentials (*E* ^*red^), and the zero‐zero excitation energy E_00_ of the investigated Cu(I) complexes referenced against Fc/Fc^+^. The determination of E_00_ and the calculation of *E* ^*red^ are described in Section S9. The calculation of the corresponding radiative (*k*_r_) and nonradiative (*k*_nr_) rate constants are specified in Section S1. The structures of the literature‐known complexes **CuRef**, **Cu2**, **Cu3**, and **Cu4** are shown in Figure S2.2.

	$E^{red,1/2}$, V	$E^{*,\mathrm{red}}$, V	*E*_00_, eV	*λ*_abs_/nm (*ε*, 10^3^ M^−1^cm^−1^)	*λ*_em_/nm^d^ (Φ_em_/%)^d^	*τ*_em_/μs^d^	*k*_*r*_, 10^4^ s^−1^	*k*_nr_, 10^6^ s^−1^
**CuRef**	−2.04^a^	0.49^a^	2.53^a^	387^a^ (5.2)^a^	578^a^ (1.4)^a^	0.31^a^	4.4^a^	3.2^a^
**Cu2**	−2.11^b^	0.46^b^	2.57^b^	384^b^ (4.7)^b^	575^b^ (2.1)^b^	0.58^b^	3.6	1.7
**Cu3**	−2.03^b^	0.50^b^	2.53^b^	385^b^ (4.9)^b^	582^b^ (1.5)^b^	0.33^b^	4.5	3.0
**Cu4**	−2.08^b^	0.48^b^	2.56^b^	382^b^ (6.4)^b^	573^b^ (2.1)^b^	0.63^b^	3.3^a^	1.6^a^
**Cu34**	−2.07^c^	0.48	2.55	390 (9.0)	573^c^ (2.4)^c^	0.84^c^	2.9	1.2
**Cu24**	−2.13	0.46	2.59	380 (5.2)	566 (4.2)	0.84	5.0	1.1
**Cu26**	−2.15	0.43	2.58	379 (5.5)	568 (4.5)	1.11	4.1	0.9
**Cu35**	−2.03	0.50	2.53	386 (5.5)	583 (1.2)	0.33	3.6	3.0
**Cu345**	−2.05	0.48	2.53	386 (5.2)	580 (2.4)	0.50	4.8	2.0
**Cu246**	−2.18	0.42	2.60	381 (6.6)	564 (8.1)	1.46	5.5	0.6

a Taken from ref. [73].

b Taken from ref. [75].

c Taken from ref. [81].

d Excited at *λ* = 385 nm.

The different behavior of *ortho/para* and *meta* substitution can be rationalized by the dominant electron‐donating mesomeric (+M) effect of the methoxy group in the *ortho* and *para* positions [82, 83, 84, 85]. The resulting increase in electron density on the phenanthroline ligand renders ligand‐centered reduction more difficult, leading to a cathodic shift in *E* ^red,1/2^. In contrast, only the slight negative inductive effect (−I) of the methoxy group is effective for *meta* substitution, and no pronounced cathodic shift is observed for **Cu35**.

Overall, methoxy substitution at the *ortho* position exerts the strongest electron‐donating effect on the phenanthroline π‐system, resulting in the largest cathodic shift. These trends are consistent with the structural and photophysical data presented below and highlight the importance of the substitution pattern in fine‐tuning the electronic properties of these Cu(I) photosensitizers. Furthermore, the excited‐state redox potentials (*E* ^*red^) were estimated using the Rehm–Weller approach [13, 54, 86].

The required zero‐zero excitation energy E_00_ (Table 2) was calculated using the tangent method (Section S9). **Cu35** exhibits the most positive excited‐state potential (*E* ^*red^ = 0.50 V), making it the most accessible compound for reductive quenching processes. It is followed by **Cu345** (0.48 V), **Cu34** (0.48 V) [81], **Cu24** (0.46 V), **Cu26** (0.43 V), and **Cu246** (0.42 V, Table 2). Given the known performance of the single methoxy‐substituted complexes **Cu2**, **Cu3**, and **Cu4** in photocatalytic reductive debromination reactions [75], the observed changes in excited‐state redox potentials for the new complexes **Cu26**, **Cu24**, **Cu35**, **Cu246**, and **Cu345** provide useful design guidelines for future optimization of Cu(I)‐based photosensitizers.

### 2.4 Photophysical Properties in Solution

**FIGURE 3:**
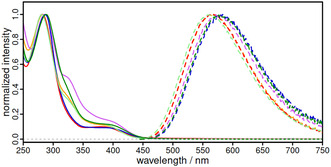
Normalized absorption spectra (solid lines) and emission spectra (dashed lines) of **Cu24** (orange), **Cu26** (red), **Cu34** (purple), **Cu35** (blue), **Cu246** (light green), and **Cu345** (dark green) in deaerated acetonitrile at room temperature. Emission spectra were recorded upon excitation at 385 nm.

The photophysical properties of the five novel Cu(I) photosensitizers **Cu24**, **Cu26**, **Cu35**, **Cu345**, and **Cu246** were studied in deaerated acetonitrile. In addition, the literature‐known complex, **Cu34**, was included for comparison [81]. Hence, absorption, emission, emission quantum yields, and lifetimes were systematically investigated and compared to the established trends of analogous methoxy‐substituted Cu(I) photosensitizers (Table 2) [72, 73, 74]. All complexes exhibit the characteristic absorption pattern of heteroleptic Cu(I) photosensitizers, comprising intense π–π* transitions below 300 nm and broad MLCT bands extending into the visible [56, 63]. This assignment is supported by TDDFT calculations using the B3LYP (D3‐BJ)/def2‐TZVP level of theory (Section S5). Depending on the number and position of the substituents, the modification exerts only a moderate effect on the absorption maxima (Figure 3). However, a slight hypsochromic shift is observed for the *ortho‐* and *para*‐substituted complexes, while all attenuation coefficients remain in the same order of magnitude. This effect appears to be particularly pronounced for substitution in the *ortho* position, as exemplified by the mono‐substituted **Cu2** (384 nm) as well as the bis‐substituted complexes **Cu24** (380 nm) and **Cu26** (379 nm), compared to the complexes without *ortho* substituents (**Cu3**: 385 nm, **Cu34**: 390 nm, **Cu35**: 386 nm, and **Cu345**: 386 nm; Table 2) [75, 81]. The emission properties are also most strongly influenced by methoxy substitution in the *ortho* and *para* positions, as evidenced by the pronounced hypsochromic shift of the emission maxima of **Cu24** (566 nm), **Cu26** (568 nm), and **Cu246** (565 nm) compared to the doubly *meta*‐substituted **Cu35** (583 nm) and the unsubstituted **CuRef** (578 nm) [73].

This trend is consistent with previous observations for related methoxy‐substituted Cu(I) complexes [73, 81]. These results can be explained by the stronger influence of *ortho* and *para* substitution on the MLCT transition energy, resulting in larger *E*_00_ values. Further, the *ortho*/*para*‐substituted complexes exhibit higher emission quantum yields (Φ_em_) and longer excited‐state lifetimes (*τ*_em_) (Table 2). The calculated radiative (*k*_r_) and nonradiative rate constants (*k*_nr_) (Table 2) show that the improved emission behavior is also associated with reduced nonradiative decay and, in some cases, also slightly enhanced radiative decay. These trends are in general consistent with the energy gap law [87, 88].

However, the gradual differences cannot be rationalized by energy‐gap effects alone, because steric constraints that limit excited‐state geometry relaxation and affect the coupling between excited and ground states also have to be considered. In contrast, *meta* substitution has little effect on E_00_, even in the case of double *meta* substitution, which is in accordance with previous studies [73, 75]. For **Cu35** (1.2%, 0.33 µs), there is thus hardly any change in the quantum yield and emission lifetime compared to the unsubstituted complex **CuRef** (1.4%, 0.31 µs) [75]. Conversely, double *ortho* substitution in the case of **Cu26** (4.5%, 1.11 µs) results in a nearly fourfold increase in quantum yield and a threefold increase in lifetime.

Taken together, the differences in absorption, emission, and excited‐state lifetimes suggest that the number and pattern of methoxy substitution can be used to tailor the performance of Cu(I) photosensitizers in a targeted manner. To probe how these differences translate into functional performance, the complexes were subsequently evaluated in a series of photocatalytic benchmark reactions covering both EnT and electron‐transfer pathways. This broad catalytic platform allows us to assess their versatility as earth‐abundant photoactive systems and to identify structure–activity relationships across different reaction classes.

### 2.5 Energy‐Transfer Photocatalysis: Singlet‐Oxygen Generation

The generation of singlet oxygen (^1^O_2_) was examined as a benchmark EnT reaction [37, 89, 90]. ^1^O_2_ is a reactive, high‐energy form of oxygen with relevance to photodynamic therapy and selective oxidation chemistry [89, 90, 91]. It is typically formed by energy transfer from the triplet excited state of a photosensitizer to triplet ground‐state oxygen (^3^O_2_) [92, 93, 94]. The resulting singlet oxygen can be detected via its characteristic near‐infrared phosphorescence at around 1275 nm, corresponding to an energy difference of 94.3 kJ mol^−1^ [92, 95]. Accordingly, singlet‐oxygen quantum yields (Φ^1^O_2_) were determined in dichloromethane by NIR emission spectroscopy using phenalenone as a reference (Table 3) [96]. This approach closely follows the methodology applied in related studies [37, 73].

**TABLE 3:** Singlet‐oxygen quantum yields Φ^1^O_2_ of the Cu(I) complexes in aerated dichloromethane and kinetic parameters derived from the DPF photooxidation analysis in aerated dichloromethane.

	Φ^1^O_2_ %^a^	*k*_1_, 10^−4^ s^−1^ b	K = *k*_1_/*k*_−1_ b	*k*_2_, 10^−5^ mol L^−1^ s^−1^ b
**Cu24**	41	5.75	3.71	3.78
**Cu26**	46	2.57	3.40	2.55
**Cu34**	43	12.2	4.82	4.53
**Cu35**	39	7.54	8.20	4.44
**Cu246**	54	4.75	2.65	3.43
**Cu345**	40	9.23	8.79	3.70

a Singlet‐oxygen quantum yields were determined by NIR emission spectroscopy upon excitation at *λ* = 385 nm.

b *k*_1_ and K values were obtained from an equilibrium‐based fit of the DPF consumption data, whereas *k*_2_ was obtained from a zero‐order fit of the DBE formation data, as detailed in Section S10.

All investigated complexes exhibit detectable ^1^O_2_ emission (Figure S10.1), confirming their ability to act as triplet photosensitizers in solution. The tri‐substituted *ortho*/*para*‐containing complex **Cu246** shows the highest Φ^1^O_2_ of 54%, which is consistent with the longest triplet lifetime (1.46 µs) and highest emission quantum yield (8.1%) within this series. The di‐substituted complexes **Cu24** and **Cu26**, bearing methoxy substituents in the *ortho* and *para* positions, give intermediate values of 41% and 46%, whereas the poly‐*meta‐*substituted complexes **Cu35** and **Cu345** show slightly lower values of 39% and 40%, respectively. Overall, the ^1^O_2_ data follow the general photophysical trend observed above, namely that stronger *ortho*/*para* substitution leads to more favorable excited‐state properties and, consequently, enhanced EnT efficiency.

### 2.6 Energy‐Transfer Photocatalysis: DPF Photooxidation

To complement the direct NIR detection of ^1^O_2_, the photooxidation of DPF to *cis*‐1,2‐dibenzoylethylene (DBE) was investigated as another EnT reaction under aerobic conditions [73, 92, 97]. This transformation is a well‐established indirect probe for ^1^O_2_ generation, as DPF reacts readily with ^1^O_2_ via a transient peroxo intermediate to afford DBE [90, 92, 97]. In contrast to the direct NIR phosphorescence measurements, the DPF assay reflects the functional outcome of ^1^O_2_ generation and provides a complementary method to probe the EnT performance of the Cu(I) complexes. The decay of DPF and the concomitant formation of DBE were monitored by in situ UV/vis spectroscopy in aerated dichloromethane under visible‐light irradiation (*λ* > 400 nm, Figure S10.2). The resulting kinetic traces were evaluated using a combined fitting approach comprising an equilibrium‐based model for DPF consumption and a zero‐order fit for DBE formation (Table 3 and Figure S10.3). Here, *k*_1_ describes the forward step of the initial DPF decay, *K* reflects the corresponding equilibrium position, and *k*_2_ represents the subsequent DBE formation step.

In contrast to the direct NIR‐based ^1^O_2_ measurements, the DPF assay does not only reflect the intrinsic ability of a photosensitizer to generate ^1^O_2_, but is additionally influenced by the overlap with the employed light source and by the subsequent reaction kinetics. Consequently, the trends observed for DPF photooxidation are related to, but not identical to, the ^1^O_2_ quantum yields. The fastest initial DPF consumption is observed for **Cu34**, which has the highest *k*_1_ value (1.22 × 10^−3^ s^−1^), followed by **Cu345** (9.23 × 10^−4^ s^−1^) and **Cu35** (7.54 × 10^−4^ s^−1^). The subsequent zero‐order fit of the DBE formation gives the highest *k*_2_ value for **Cu34** (4.53 × 10^−5^ mol L^−1^ s^−1^), confirming that **Cu34** also performs particularly well in the overall functional photooxidation experiment. By contrast, **Cu246**, despite having the highest Φ^1^O_2_, exhibits only intermediate kinetic parameters in the DPF assay (Table 3). These observations are consistent with the attenuation coefficient (Figure S10.4), where the absorption overlap in the emission range of the employed Xe lamp is strongest for **Cu34**. In addition, the somewhat higher performance of **Cu345** compared to **Cu35** is plausible in view of its longer excited‐state lifetime (**Cu345**: 0.50 µs and **Cu35**: 0.33 µs). Taken together, the DPF experiments confirm that all investigated Cu(I) complexes are competent photosensitizers for functional ^1^O_2_ chemistry and reveal that the application‐oriented DPF oxidation is governed by multiple factors beyond the intrinsic Φ^1^O_2_ alone. This explains why **Cu246** is the best direct ^1^O_2_ sensitizer, whereas **Cu34, Cu345,** and **Cu35** perform particularly well under the applied DPF photooxidation conditions.

### 2.7 Electron‐Transfer Photocatalysis: Reductive Debromination

**FIGURE 4:**
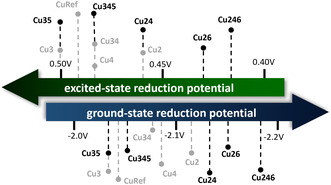
Summary of ground‐ and excited‐state potentials of all methoxy substituted Cu(I) complexes investigated in this study (black) and reported previously (gray).

In order to rigorously assess the electron‐transfer capabilities of our Cu(I) photosensitizers, two benchmark photoredox reactions were carried out: (i) reductive debromination of aryl bromides [1, 73, 98] and (ii) C–H alkylation of isoquinolines [99, 100, 101]. Based on the ground‐ and excited‐state reduction potentials (summarized in Figure 4), together with the excited‐state lifetimes discussed above, these reactions allow us to evaluate how the number and position of methoxy substituents affect electron‐transfer photocatalysis.

In our previous study of mono‐methoxy‐substituted Cu(I) photosensitizers, a moderate but clear influence of substituent position on reductive debromination performance was observed [75]. In addition, Stern–Volmer quenching experiments with triethylamine (TEA) for **Cu3** and **Cu4** revealed efficient reductive quenching, confirming that this system is well suited for photocatalytic benchmark reactions under reductive conditions [75].

Specifically, the *para*‐methoxy derivative **Cu4** as well as the *ortho*‐methoxy complex **Cu2** delivered the highest yields in the reductive debromination of 2‐bromoacetanilide, whereas the *meta*‐methoxy analog **Cu3** exhibited the lowest activity. However, **Cu3** was still more active than the unsubstituted reference complex **CuRef**.

**FIGURE 5:**
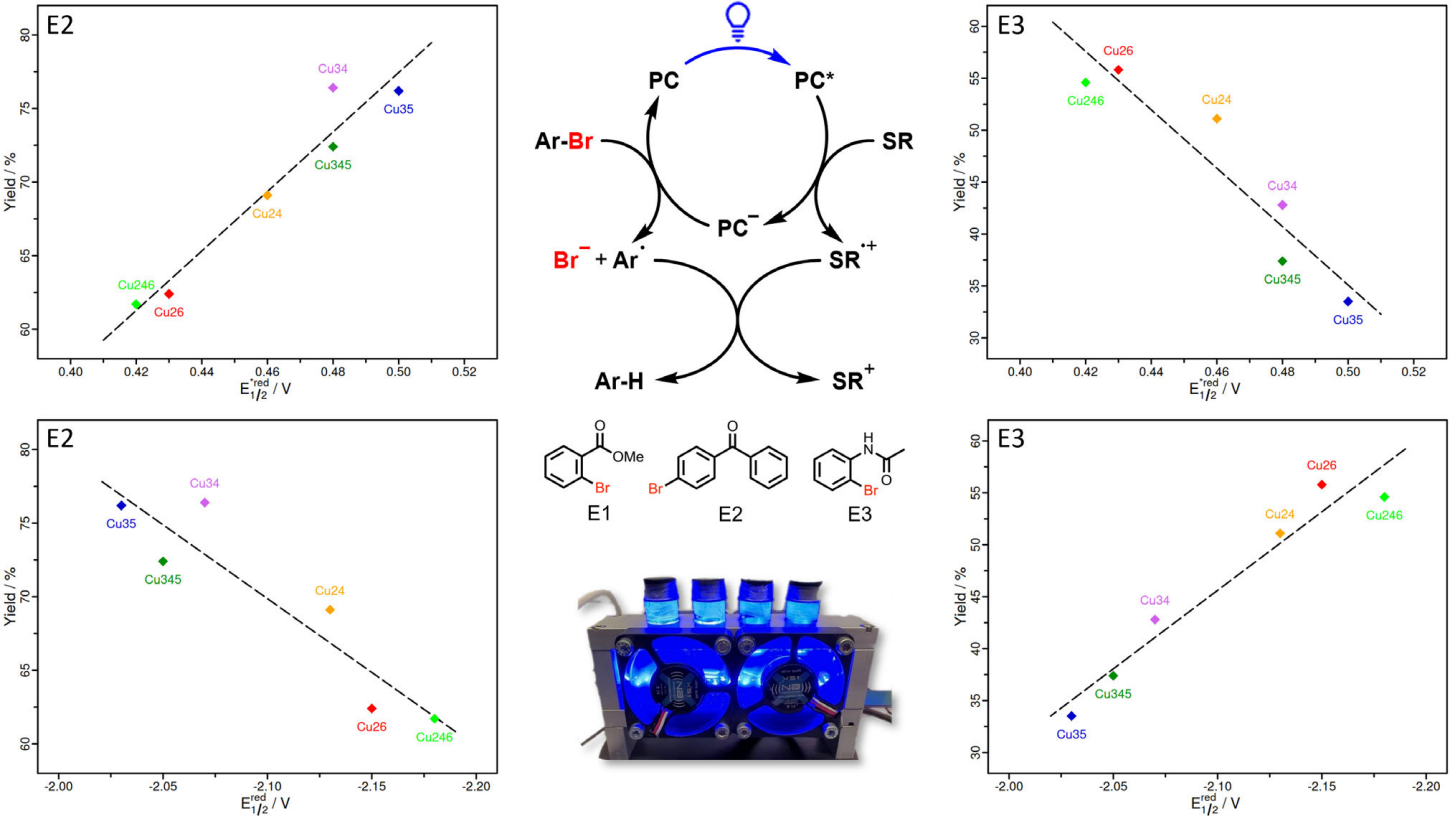
Overview of the reductive debromination study, including the correlation between product yields and the excited‐state redox potentials for E2 (top left) and E3 (top right), the correlation between product yields and the reduction potentials of the ground‐state species for E2 (bottom left) and E3 (bottom right), the simplified reaction mechanism, the chemical structure of the substrates E1–E3, and a photograph of the photoreactor. The depicted reaction scheme is intentionally simplified and does not explicitly resolve the detailed proton‐transfer interplay between BIH and TEA [98]. Black dashed lines in plots represent linear regression fits through the displayed data points.

In this study, visible‐light‐driven reductive debromination was performed on a series of three different aryl bromides (Figure 5) of varying reactivity: 2‐bromobenzoate (E1), 4‐bromobenzophenone (E2), and 2‐bromoacetanilide (E3). This substrate‐dependent reactivity is consistent with the stronger electron‐withdrawing character of the ester and carbonyl substituents in E1 and E2, which facilitate reductive C—Br bond activation, whereas the acetamido substituent in E3 renders the aryl bromide electronically less accessible toward reduction. Mechanistically, upon excitation, the photocatalyst is reductively quenched by a sacrificial reagent, followed by an electron transfer to the aryl bromide, generating a bromide anion and an aryl radical. The latter is subsequently converted to the dehalogenated product via repeated reduction and protonation [73, 98]. Accordingly, two key steps can be identified through which the photocatalyst influences the reaction rate: reductive quenching of the photocatalyst and subsequent electron transfer to the aryl bromide. Both steps are crucial for interpreting the observed product yields and are strongly dependent on the redox potentials of the excited state as well as the reduced ground state of the photocatalyst [102, 103, 104].

These relationships become particularly evident when examining the obtained results. For the faster‐reacting substrates, E1 and E2, the product yields closely follow the excited‐state potentials. Here, more positive excited‐state reduction potentials support more efficient reductive quenching, which in turn leads to increased product yields (Figure 5). Consequently, the partially *meta*‐substituted complexes **Cu34** (0.48 V), **Cu35** (0.50 V), and **Cu345** (0.48 V), which display the highest excited‐state potentials within this series, also give the highest yields for the fast‐reacting substrate E1 (**Cu34**: 78.0%, **Cu35**: 72.5%, and **Cu345**: 72.6%). A similar trend can be observed for the second fast‐reacting substrate E2, where **Cu34** (76.3%), **Cu35** (76.2%), and **Cu345** (72.4%) again provide the highest yields, consistent with their favorable excited‐state properties.

Interestingly, this trend is reversed for reactions involving the slower reacting substrate E3. In this case, complexes with higher excited‐state potentials no longer give the highest yields, indicating that the rate‐determining step has changed. A nearly linear correlation is now observed between the ground‐state reduction potential of the reduced species and the product yield, with more negative potentials corresponding to higher yields (Figure 5). A more negative reduction potential indicates that the reduced species has a stronger driving force to donate the acquired electron. This suggests that electron transfer to the aryl bromide has become the rate‐determining step for E3. Accordingly, the highest yields are obtained for **Cu26** and **Cu246**, with 55.8% and 54.6% and redox potentials of −2.15 V and −2.18 V, respectively. The remaining complexes follow the order **Cu24** (51.1%, −2.13 V) > **Cu34** (42.8%, −2.07 V) > **Cu345** (37.4%, −2.05 V) > **Cu35** (33.5%, −2.03 V), consistent with their respective redox potentials (Figure 4). For E3, the determined conversions and yields were identical within experimental error (Table S10.3), indicating a highly selective debromination under the applied conditions without detectable side‐product formation.

This behavior highlights the importance of tuning Cu(I) complexes according to the demands of the specific reaction. Depending on the substrate, either efficient reductive quenching of the excited state or strong reducing power of the reduced species can become the dominant factor. Thus, targeted substitution at different positions can be used to achieve improved performance for substrates with different reactivity requirements.

### 2.8 Electron‐Transfer Photocatalysis: Aza‐Henry Reaction

**FIGURE 6:**
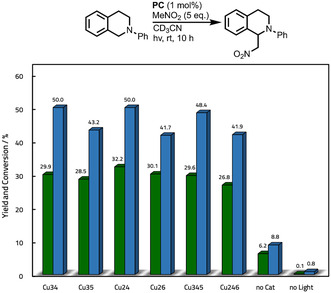
Yields (green) and conversions (blue) in % for the photocatalytic aza‐Henry reaction of the five new Cu(I) complexes and the corresponding control experiments (without photocatalyst, without light).

As a complementary electron‐transfer benchmark, the visible‐light‐driven aza‐Henry reaction of *N*‐phenyl‐1,2,3,4‐tetrahydroisoquinoline (THIQ) with nitromethane was investigated (Figure 6) [99, 100, 101, 105]. This transformation is a well‐established C—H functionalization reaction in which oxidative activation of the amine substrate gives access to an iminium‐type intermediate that can subsequently be trapped by a nitroalkane nucleophile [99, 100, 106]. In the context of this study, the reaction mainly serves to probe whether the new Cu(I) complexes are not only competent in reductive photoredox transformations, but can also mediate an oxidative C—C bond‐forming reaction under mild conditions.

Reactions were conducted in NMR tubes under continuous 460 nm irradiation for 10 h in deuterated acetonitrile using 1.0 mol% photocatalyst and nitromethane (5 equiv., reaction conditions are given in the Supporting Information). Control experiments confirmed that light is essential, as no reaction was observed in the dark. In contrast, a small but measurable background conversion was detected in the absence of Cu(I) photocatalyst (Figure 6), which is consistent with the known sensitivity of THIQ derivatives toward slow photooxidation under aerobic conditions.

Under the applied conditions, all five Cu(I) complexes exhibited comparable activity, giving 27%–32% yield at 42%–50% conversion (Figure 6) [100, 101]. Thus, the reaction clearly confirms that the investigated Cu(I) complexes are also active in an oxidative C–H functionalization reaction. However, the differences between the individual complexes remain small and should therefore not be overinterpreted in terms of detailed structure–activity relationships.

## 3 Conclusions

In this study, we extended the methoxy‐substitution concept in heteroleptic Cu(I) photosensitizers from mono‐substituted to poly‐substituted systems by synthesizing five new complexes and systematically comparing them with the previously reported mono‐substituted and reference compounds. The results demonstrate that both the number and, even more importantly, the position of methoxy groups govern the photophysical, electrochemical, and photocatalytic properties of this Cu(I) platform. While all complexes retain the expected distorted tetrahedral Cu(I) coordination motif, the different substitution patterns induce subtle structural and electronic changes that translate into clearly different functional behavior. **Cu246** has the highest emission quantum yield (8.1%), the longest excited‐state lifetime (1.46 μs), and the most efficient singlet‐oxygen sensitization (54%) in the series. By contrast, the doubly *meta*‐substituted **Cu35** remains close to the unsubstituted reference, with an emission quantum yield of 1.2%, an excited‐state lifetime of 0.33 μs, and a singlet‐oxygen quantum yield of 39%. These findings highlight that methoxy substitution does not uniformly enhance the performance of Cu(I) photosensitizers, but rather tunes the balance between absorptivity, excited‐state dynamics, and redox properties in a strongly position‐dependent manner. For the EnT benchmark reactions, the direct singlet‐oxygen measurements identify **Cu246** as the most effective sensitizer, whereas the DPF photooxidation studies reveal **Cu34** as the fastest photocatalyst under the applied conditions, indicating that the functional photooxidation outcome does not simply scale with the ^1^O_2_ quantum yield alone. However, a more differentiated picture emerges for the electron‐transfer benchmark reactions. In the reductive debromination, yields of faster‐reacting substrates correlate more closely with the excited‐state redox properties, whereas yields of the more demanding substrate E3 follow the reduction potentials of the reduced species. In the case of the latter, **Cu26** and **Cu246** give the highest yields (55.8% and 54.6%, respectively), showing that stronger ground‐state reducing power can become more important than favorable excited‐state quenching. The aza‐Henry reaction further demonstrates activity in oxidative C–H functionalization, although only small differences between the individual Cu(I) complexes were observed.

Together, the photocatalytic studies underline the versatility of these earth‐abundant Cu(I) photosensitizers across both energy‐ and electron‐transfer reaction manifolds and thus establish clear molecular design principles for engineering heteroleptic Cu(I) complexes with tailored photocatalytic performance. The results reveal practical structure–property–activity relationships that enable a rational balance between ground‐ and excited‐state properties governing catalytic function.

## Funding

This study was supported by the Fonds der Chemischen Industrie (FCI, 115571) and by the Deutsche Forschungsgemeinschaft (DFG, 524554621).

## Conflicts of Interest

The authors declare no conflicts of interest.

## Supporting information

Crystallographic data for the complexes **Cu35** and **Cu345** and the ligand **L246** have been deposited at the joint Cambridge Crystallographic Data Centre (CCDC) and Fachinformationszentrum Karlsruhe under deposition numbers CCDC 2550575, 2550576, and 2550577. The data can be obtained from www.ccdc.cam.ac.uk/structures/ or by emailing data_request@ccdc.cam.ac.uk.

## Data Availability

The data that supports the findings of this study are available in the Supporting Information of this article. Additional data are available from the corresponding authors upon reasonable request.
