# Supplementary material for: Number and Position of Methoxy Substituents Govern Photophysics and Visible‐Light Photocatalysis in Earth‐Abundant Cu(I) Complexes
Source: ChemSusChem. 2026 Aug 12;19(16):e70931. doi: 10.1002/cssc.70931 (PMC13464044; doi:10.1002/cssc.70931)
Supplement: Supplementary file 1 [file CSSC-19-e70931-s001.pdf]

Supporting Information

Number and Position of Methoxy Substituents Govern Photophysics and Visible-Light Photocatalysis in Earth-Abundant Cu(I) Complexes

Kurt J. Haseloff,[+,a] Max Wolf,[+,a] Katharina Rediger,[b] Christian Kleeberg,[c] Maria Wächtler,[d] Stefanie Tschierlei,[,a] and Michael Karnahl[*,a]*

[a] Department of Energy Conversion, Institute of Physical and Theoretical Chemistry, Technische Universität Braunschweig, Rebenring 31, 38106 Braunschweig, Germany.
[b] Department of Chemistry, Rheinland-Pfälzische Technische Universität Kaiserslautern-Landau, Erwin-Schrödinger-Straße 52, 67663 Kaiserslautern, Germany.
[c] Institute of Inorganic and Analytical Chemistry, Technische Universität Braunschweig, Hagenring 30, 38106 Braunschweig, Germany.
[d] Institute of Physical Chemistry, Christian-Albrechts-Universität zu Kiel, Max-Eyth-Straße 1, 24118 Kiel, Germany.

[+] These authors contributed equally and share first authorship.
*Correspondence:
Prof. Dr. Stefanie Tschierlei: s.tschierlei@tu-bs.de
PD Dr. Michael Karnahl: michael.karnahl@tu-bs.de

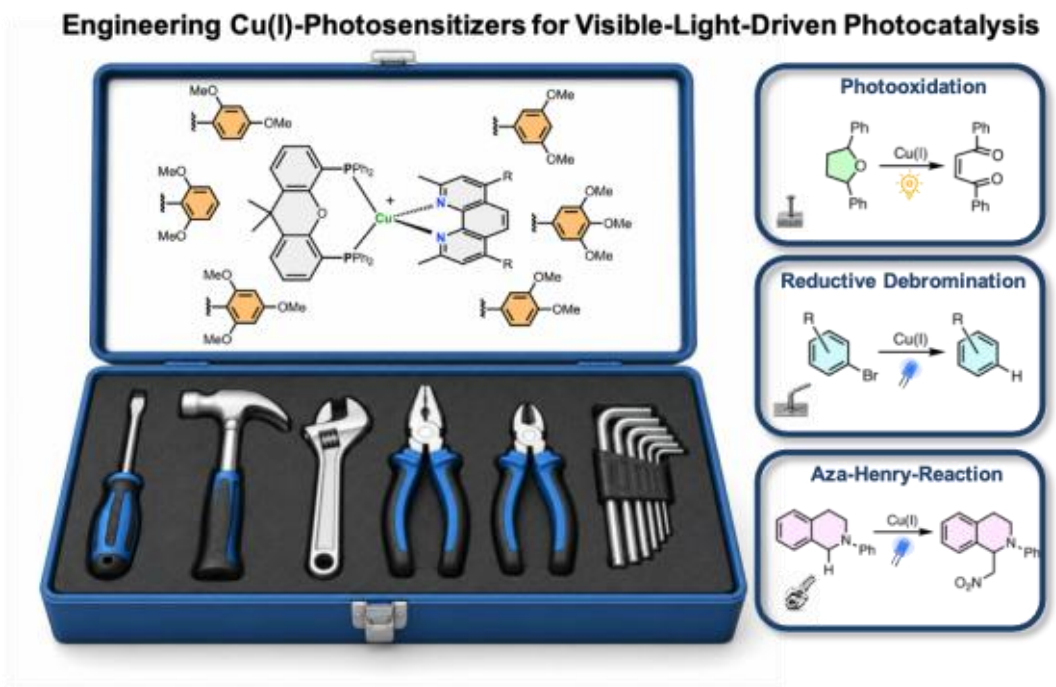

Table of Contents

1 Experimental and Computational Details 2
2 Synthetic Details 6
3 NMR Spectroscopy 11
4 Crystallographic Data 29
5 (Time-Dependent) Density Functional Theory (TD-DFT) 32
6 Steady-State UV/vis Absorption and Emission Spectroscopy 50
7 Time-Resolved Emission Spectroscopy 51
8 Electrochemical Data 53
9 Excited-State Reduction Potentials 54
10 Photocatalysis 56
11 References 66

1 Experimental and Computational Details

NMR Spectroscopy. Nuclear magnetic resonance (NMR) spectra, including ^{1}H NMR, $^{13}C\{^{1}H\}$ NMR, and $^{31}P\{^{1}H\}$ NMR, were acquired using spectrometers of the *Bruker Avance* Series (400 MHz, 500 MHz or 600 MHz) at 298 K. The operating frequency is specified in the respective NMR data. The measurements were performed by the Institute of Inorganic and Analytical Chemistry at the Technische Universität Braunschweig. Measurements were conducted using Wilmad 528-PP NMR tubes and the solvent for each sample is specified in the respective NMR data. The acquired NMR spectra were subsequently processed using TopSpin software (version 4.5.0). The chemical shifts δ are reported in ppm relative to tetramethylsilane (TMS), and all NMR spectra were referenced to the TMS signal. Coupling constants (J) are presented as absolute values in hertz (Hz). The NMR signal splitting was described using standard notations: '*s*' for singlet, '*d*' for doublet, '*t*' for triplet, '*dd*' for doublet of doublets, '*td*' for triplet of doublets, '*sept*' for septet, and '*m*' for multiplet.

Mass Spectrometry. Mass spectrometric (MS) analyses were performed by the analytical service of the Institute of Organic Chemistry, Technische Universität Braunschweig, using an LTQ-Orbitrap Velos mass analyzer from Thermo Fisher Scientific. High-resolution mass spectra were obtained *via* electrospray ionization (ESI). Compounds were dissolved in methanol and spiked with 0.1 mg/mL tetradecyltrimethylammonium bromide. Mass spectrometry values are reported as mass-to-charge ratios (*m/z*).

Elemental Analysis (EA). The elemental analyses were performed by the Institute of Inorganic and Analytical Chemistry at the Technische Universität Braunschweig, using a varioMICRO Cube apparatus from Elementar. Under the CHN configuration, only carbon (C), hydrogen (H), and nitrogen (N) were measured. Helium served as the inert gas.

X-ray Diffraction Studies. A single crystal was mounted on top of a Hampton loop using inert perfluoroether oil and placed in the cold nitrogen gas stream on the diffractometer.[1] The diffraction data were collected on a Rigaku Oxford Diffraction Synergy-S instrument using mirror-focused CuKα or MoKα radiation from a Rigaku Photonjet microfocus source. The reflections were indexed, integrated and corrected for absorption as implemented in the CrysAlisPro software package.[2] The structures were solved using SHELXT and refined anisotropically for all non-hydrogen atoms by full-matrix least squares on all F2 using SHELXL software.[3] During refinement and analysis of the crystallographic data, the programs Mercury, Platon and Olex2 were used.[4] Further details are found in in Tables S4.1–S4.3. Supplementary crystallographic data is available under CCDC 2550575-2550577. X-ray structures were visualized using Diamond (Crystal Impact). Hydrogen atoms were omitted for clarity, and non-hydrogen atoms were drawn as thermal ellipsoids at a 50% probability level.[5]

(TD-)DFT Calculations. The ground state S_0 geometries of the complexes were optimized using the Berny algorithm of Gaussian 16,[6] with the energies and gradients computed by Turbomole (v.7.8).[7] All optimizations were performed with the DFT functional B3LYP with additional dispersion correction (D3-BJ, as implemented in Turbomole) and the def2-TZVP basis set. For acceleration, the resolution of identity (RI) in combination with the multipole accelerated RI-J with a def2-TZVP auxiliary basis set was used. To also consider solvation effects, the conductor-like screening model (COSMO) for acetonitrile was applied. All optimized geometries were checked for negative frequencies and were assumed to be energy minima if no negative frequencies above 5 cm^{-1} were present. The vertical electronic transitions were

analyzed in terms of natural transition orbitals, which were calculated using TURBOMOLE as well. The obtained isosurfaces were visualized using CHEMCRAFT with an isovalue of ±0.03. The simulated spectrum was plotted using a Gaussian line shape and an FWHM of 2500 cm^{-1}.

Steady-state UV/vis Absorption Spectroscopy. UV/vis absorption spectra were obtained using a JASCO V770 spectrophotometer. The compounds were dissolved in acetonitrile or methanol (HPLC grade) and the resulting spectra were recorded using a standard 10 mm fluorescence quartz glass cuvette. All spectra were baseline corrected at 800 nm.

Steady-state Emission Spectroscopy. The emission spectra were recorded using a HORIBA JOBIN-YVON FLUOROMAX PLUS-C emission spectrometer. All samples were measured in dry acetonitrile under inert conditions using a sealed 10 mm fluorescence quartz glass cuvette. Optical densities (OD) were approximately 0.1 at the respective excitation wavelength. The emission quantum yields were calculated using the following equation (eq. 1.1).

$$\Phi_c = \Phi_R \left(\frac{\eta_C^2}{\eta_R^2}\right)\left(\frac{A_R}{A_C}\right)\left(\frac{I_C}{I_R}\right) \qquad \text{eq. 1.1}$$

Time-resolved Emission Spectroscopy. Time-resolved emission spectra were obtained using a streak camera system (HAMAMATSU PHOTONICS C10910-01 Main Unit, accompanied by a M10913 Slow Single Sweep Unit). The signal acquisition process was facilitated by the utilization of an ORCA-Flash 4.0 V3 camera from HAMAMATSU PHOTONICS. The spectrograph employed in this study is a Kymera 328i-A from ANDOR, which is directly connected to the streak unit. Excitation was performed using a titanium:sapphire laser (Solstice Ace from SPECTRA PHYSICS) with a repetition rate of 1 kHz and an approximate pulse width of 70 fs. The output was directly coupled to an optical parametric amplifier (LIGHT CONVERSION TOPAS PRIME with NirUVis extension) to set the excitation wavelength to 390 nm. The power at the sample was approximately 320 nW. The center wavelength of the spectrograph is specified for each spectrum. The emission was detected using the single photon counting mode in a time range of 10-50 µs. For all measurements, the input slit of the spectrograph was set to 250 µm, the slit in front of the streak camera to 20 µm, and a 50 grooves/mm grating was utilized. The measurements were obtained with an exposure time of 30 milliseconds and accumulated over 60000 exposures. All samples were prepared under inert conditions, and the optical density was set to 0.1 at 390 nm. The emission lifetime was determined by fitting equation (1.2).

$$f(t) = \sum_{i=1}^{N} A_i \cdot e^{-\frac{t}{\tau_i}} + A_0 \qquad \text{eq. 1.2}$$

Calculation of k_r and k_{nr}. Radiative (k_r) and non-radiative (k_{nr}) rate constants were estimated using following formalism (1.3).[8]

$$k_r = \frac{\phi_{em}}{\tau_{em}}\ ;\ k_{nr} = \frac{1}{\tau_{em}} - k_r \qquad \text{eq. 1.3}$$

Electrochemistry. Electrochemical measurements were performed on a METROHM AUTOLAB PGSTAT204 potentiostat in deaerated acetonitrile containing 1 mM of the analyte and 0.1 M $[Bu_4N][PF_6]$ as the supporting electrolyte. A three-electrode configuration was utilized, consisting of a glassy carbon working electrode with a diameter of 3 mm, a platinum wire counter electrode, and a non-aqueous Ag/Ag^+ reference electrode. Cyclic voltammograms were recorded at a scan rate of 100 mV s^{-1}.

Singlet Oxygen Measurement. Singlet-oxygen quantum yields (ϕ^1O_2) were determined by monitoring the near-infrared phosphorescence of $^1O_2$ at ca. 1276 nm using a HORIBA JOBIN-YVON FLUOROMAX PLUS-C spectrofluorometer. The instrument was equipped with a 150 W Xe-arc excitation source, Czerny-Turner monochromators fitted with an NIR grating blazed at 1000 nm, a R13456 photomultiplier-tube detector (190-930 nm) and a liquid-nitrogen-cooled DSS-IGA020L InGaAs photodiode (800-1550 nm). To ensure sample integrity, absorption spectra were recorded before and after each luminescence measurement on a JASCO V-770 UV–Vis–NIR spectrometer. Emission spectra of each sample were collected upon excitation at 385 nm. Phenalenone (ϕ^1O_2 = 0.98 in the literature[9]) was employed as an external standard under identical instrumental settings. Quantum yields for the unknowns were then calculated (*cf.* eq. 1.1) by comparing their integrated intensities to that of phenalenone.

Photooxidation of 2,5-diphenylfuran (DPF). Initially, a solution with a concentration of $1.9 \cdot 10^{-6}$ M was prepared for every photosensitizer (**Cu24**, **Cu26**, **Cu34**, **Cu35**, **Cu246** and **Cu345**), as well as a solution of DPF with a concentration of $1.9 \cdot 10^{-5}$ M. Yielding a ratio of 1:10, 1.5 mL of both solutions were added into a cuvette and irradiated with a xenon lamp (0.850 W at cuvette, distance: 16 cm, spectra see S10.4) over a time of 60 min. The decomposition of DPF (335 nm) and the formation of the product DBE (255 nm) were monitored using the corresponding absorption bands via *in situ* UV/vis absorption spectroscopy, employing an AVANTES StarLine AvaSpecULS2048CL-EVO-RS spectrophotometer connected to an AVANTES AvaLight-DH-S-BAL light source (integration time 2.00 ms, averaged over 100 measurements, measurement every 10 s).

Photocatalytic Reductive Debromination. A vial was charged with 0.05 mmol of BIH, evacuated and filled with argon. Under inert atmosphere, 0.5 mmol triethylamine was added. Subsequently, 1.5 mL of a previously prepared, deaerated acetonitrile solution containing 0.05 mmol starting material, 0.0005 mmol Cu(I) photosensitizer and 0.01705 mmol deaerated *n*-hexadecane was added to the vial under inert conditions. The vial was sealed with a screw cap with a Teflon plate, and placed in a photoreactor (blue LED irradiation (λ = 460 nm)) for the respective reaction time of 10 minutes for E_1 (methyl 2-bromobenzoate), 15 minutes for E_2 (4-bromobenzophenone) or 5 hours for E_3 (2-bromoacetanilide). An airstream was used to cool the reaction mixture, which was stirred at 300 rpm using a magnetic stirrer, while the distance to the light source remained constant. After the reaction, 30 µL of the solution was diluted in 1 mL dichloromethane and the amounts of Ar-H and Ar-Br were determined by GC-FID analysis, using *n*-hexadecane as the internal standard. All photocatalytic experiments were performed and quantified independently in duplicate. Control experiments confirmed that light, photocatalyst, and sacrificial donors are all needed.

Photocatalytic Aza-Henry Reaction. *N*-Phenyl-1,2,3,4-tetrahydroisoquinoline (THIQ) was prepared according to the literature.[10] THIQ (50 mM, 1.0 eq.), nitromethane ($MeNO_2$, 250 mM, 5.0 eq.) and the internal standard hexamethylcyclotrisiloxane (D3, 10 mM, 0.2 eq.) were dissolved in deuterated acetonitrile (CD_3CN). Corresponding Cu(I) complex (500 µM, 1.0 mol%)

was used as the photocatalyst. The reaction was carried out in an NMR tube (*WILMAD*, 528-PP) under aerobic conditions. Samples were irradiated with blue LED's (λ = 460 nm) at room temperature for 10 hours. ^1H NMR (400 MHz, 25°C) were recorded before (0 h) and after (10 h) the reaction. Final yields and conversions were calculated by using the internal standard D3. Blank reactions conducted in the absence of the photocatalyst and in the absence of light confirmed the necessity of both parameters.

2 Synthetic Details

General Techniques. All reactions were carried out under an argon atmosphere, unless otherwise specified. Standard Schlenk techniques were employed. Glassware was dried under vacuum and then flushed with argon. This process was repeated three times to ensure complete removal of any residual moisture or air. Solvent removal was achieved using a rotary evaporator from HEIDOLPH, followed by drying under high vacuum (10^{-3} mbar) and heating at 45 °C.

Chemicals and Solvents. The chemicals utilized in the experiments were purchased from different suppliers such as SIGMA-ALDRICH, CARL ROTH or BLDPHARM and were used as received. Solvents, acids, and bases were provided by the chemical supply of the Chemistry Institute at the Technische Universität of Braunschweig. Dry, inert solvents were obtained through distillation over suitable drying agents under an inert atmosphere. Degassed water was prepared in a Schlenk flask by purging with argon. Solvents used for column chromatography were employed without further treatment.

Thin Layer Chromatography. To monitor the progress of the reaction, neutral aluminum oxide plates (*60 F254*) from MERCK were employed and visualized using UV detection (λ = 254 nm or 365 nm).

Column Chromatography. Column chromatography utilized aluminum oxide (90 active neutral) from MERCK as the stationary phase. Overpressure was applied manually using a pump ball to facilitate the chromatographic process. Efforts were made to recycle solvents, when possible, to promote sustainability.

The syntheses of the ligands **L2, L3, L4,** and **L34** and of the corresponding complexes **Cu2**, **Cu3**, **Cu4,** and **Cu34** were adapted from the literature.[11,12] The synthesis of the new ligands **L24**, **L26**, **L35**, **L345** and **L246** and complexes **Cu24, Cu26, Cu35, Cu345** and **Cu246** is described below.

2.1 Synthesis of Ligands (**L24**, **L26**, **L35**, **L345**, **L246**)

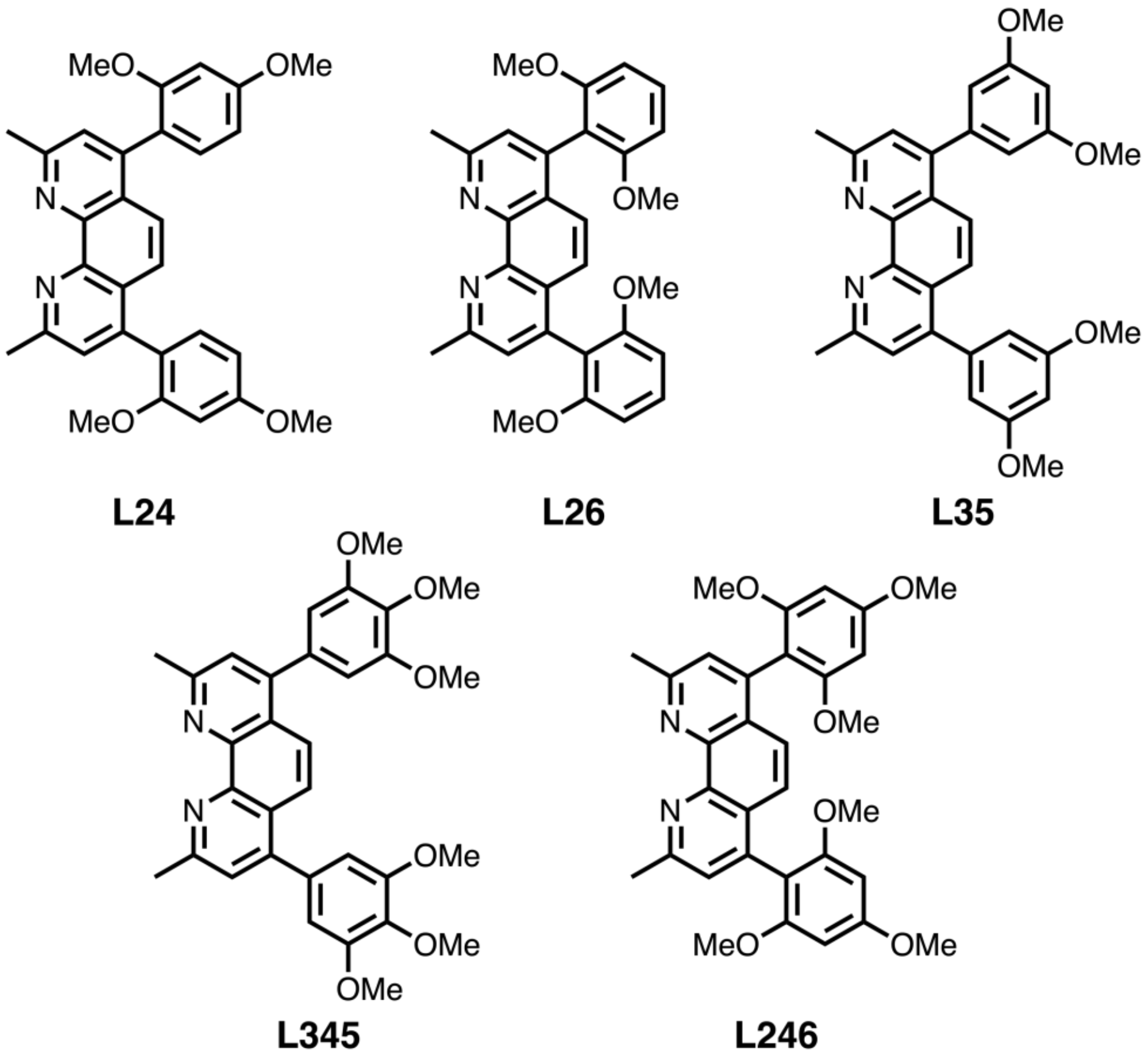

Figure S2.1. Overview of the methoxy-substituted bathocuproine ligands and their abbreviations.

Into a 100 mL flame-dried Schlenk flask, 4,7-dichloro-neocuproine (0.72 mmol, 1 eq.), boronic acid (2.17 mmol, 3 eq.), Xphos-Pd-G2-Cat. (0.02 mmol, 3 mol%) and tripotassium phosphate salt (12.27 mmol, 17 eq.) were added, and the apparatus was evacuated and backfilled with argon three times. Degassed tetrahydrofuran (18 mL) and degassed water (20 mL) were added to the solid mixture. The reaction mixture was refluxed for 16 h under vigorous stirring. The solvent was evaporated, and the aqueous phase was extracted with dichloromethane (3x). The combined organic phases were washed with aq. NaOH (2 M, 3x) and with water (2x). The organic phase was dried with $MgSO_4$ and the solvent was removed under reduced pressure. The crude product was purified over aluminum oxide (neutral, dichloromethane, 1% MeOH) to obtain white-beige solids.

L24 (66%, $C_{30}H_{28}N_2O_4$, M: 480.56 g/mol): **^{1}H NMR** ($CDCl_3$, 600 MHz, 25 °C) δ [ppm] = 7.42 (*s*, 1H, Ar*H*), 7.40 (*s*, 1H, Ar*H*), 7.37 (*s*, 1H, Ar*H*), 7.38 (s, 1H, Ar*H*), 7.36 (*s*, 1H, Ar*H*), 7.21 (*d*, *J* = 7.2 Hz, 1H, Ar*H*), 7.18 (*d*, *J* = 7.2 Hz, 1H, Ar*H*), 7.08 (*m*, 4H, Ar*H*), 3.89 (*s*, 6H, OC*H*$_3$), 3.69 (*s*, 3H, OC*H*$_3$), 3.65 (*s*, 3H, OC*H*$_3$), 2.96 (*s*, 6H, C*H*$_3$). **^{13}C{^{1}H} NMR** ($CDCl_3$, 150 MHz, 25 °C) δ [ppm] = 161.4, 158.4, 158.0, 145.8, 132.1, 125.9, 124.8, 123.3, 120.3, 120.1, 104.7, 99.0, 55.6, 26.1.

DEPT 135 NMR ($CDCl_3$, 150 MHz, 25 °C) *δ* [ppm] = 131.8(+), 124.6(+), 123.1(+), 104.5(+), 98.8(+), 55.4(+), 25.9(+). **HRMS (ESI)** *m/z*: calcd. for [**L24**+H]$^+$: 481.2127, found: 481.2126.

L26 (82%, $C_{30}H_{28}N_2O_4$, M: 480.56 g/mol): **^{1}H NMR** ($CDCl_3$, 500 MHz, 25 °C) *δ* [ppm] = 7.37 (*dd*, J_a = 8.4 Hz, J_b = 8.3 Hz, 2H, Ar*H*), 7.33 (*s*, 2H, Ar*H*), 7.23 (*s*, 2H, Ar*H*), 6.69 (*s*, 2H, Ar*H*), 6.67 (*s*, 2H, Ar*H*), 3.62 (*s*, 12H, OCH_3), 2.96 (*s*, 6H, CH_3). **^{13}C{^{1}H} NMR** ($CDCl_3$, 125 MHz, 25 °C) *δ* [ppm] = 158.1, 158.1, 146.0, 142.1, 130.0, 126.4, 125.5, 123.0, 115.8, 104.2, 99.0, 56.0, 26.0. **DEPT 135 NMR** ($CDCl_3$, 125 MHz, 25 °C) *δ* [ppm] = 129.8(+), 125.3(+), 122.8(+), 104.0(+), 55.8(+), 25.8(+). **HRMS (ESI)** *m/z*: calcd. for [**L26**+H]$^+$: 481.2127, found: 481.2127.

L35 (57%, $C_{30}H_{28}N_2O_4$, M: 480.56 g/mol): **^{1}H NMR** ($CDCl_3$, 500 MHz, 25 °C) *δ* [ppm] = 7.80 (*s*, 2H, Ar*H*), 7.45 (*s*, 2H, Ar*H*), 6.64 (*d*, *J* = 2.4 Hz, 4H, Ar*H*), 6.58 (*t*, J_a = 4.6 Hz, J_b = 2.4 Hz, 2H, Ar*H*), 3.84 (*s*, 12H, OCH_3), 2.99 (*s*, 6H, CH_3). **^{13}C{^{1}H} NMR** ($CDCl_3$, 125 MHz, 25 °C) *δ* [ppm] = 161.0, 158.9, 148.7, 146.0, 140.4, 124.8, 123.8, 123.2, 108.0, 100.5, 55.7, 26.1. **DEPT 135 NMR** ($CDCl_3$, 125 MHz, 25 °C) *δ* [ppm] = 123.6(+), 123.0(+), 107.8(+), 100.3(+), 55.5(+), 25.9(+). **HRMS (ESI)** *m/z*: calcd. for [**L35**+H]$^+$: 481.2127, found: 481.2127.

L345 (72%, $C_{32}H_{32}N_2O_6$, M: 540.62 g/mol): **^{1}H NMR** ($CDCl_3$, 500 MHz, 25 °C) *δ* [ppm] = 7.83 (*s*, 2H, Ar*H*), 7.46 (*s*, 2H, Ar*H*), 6.70 (*s*, 4H, Ar*H*), 3.94 (*s*, 6H, OCH_3), 3.89 (*s*, 12H, OCH_3), 3.01 (*s*, 6H, CH_3). **^{13}C{^{1}H} NMR** ($CDCl_3$, 125 MHz, 25 °C) *δ* [ppm] = 159.0, 153.5, 148.8, 138.3, 138.3, 133.9, 124.9, 123.9, 123.2, 107.1, 61.2, 56.5, 26.1. **DEPT 135 NMR** ($CDCl_3$, 125 MHz, 25 °C) *δ* [ppm] = 123.7(+), 123.0(+), 106.9(+), 61.0(+), 56.3(+), 25.9(+). **HRMS (ESI)** *m/z*: calcd. for [**L345**+H]$^+$: 541.2339, found: 541.2335.

L246 (80%, $C_{32}H_{32}N_2O_6$, M: 540.62 g/mol): **^{1}H NMR** ($CDCl_3$, 500 MHz, 25 °C) *δ* [ppm] = 7.31 (*s*, 2H, Ar*H*), 7.26 (*s*, 2H, Ar*H*), 6.25 (*s*, 4H, Ar*H*), 3.89 (*s*, 6H, OCH_3), 3.61 (*s*, 12H, OCH_3), 2.94 (*s*, 6H, CH_3). **^{13}C{^{1}H} NMR** ($CDCl_3$, 125 MHz, 25 °C) *δ* [ppm] = 161.7, 158.8, 158.0, 146.0, 142.0, 126.8, 126.0, 123.0, 108.6, 91.0, 56.0, 55.6, 26.0. **DEPT 135 NMR** ($CDCl_3$, 125 MHz, 25 °C) *δ* [ppm] = 125.8(+), 122.8(+), 90.8(+), 55.8(+), 55.4(+), 25.8(+). **HRMS (ESI)** *m/z*: calcd. for [**L246**+H]$^+$: 541.2339, found: 541.2335.

2.2 Synthesis of Complexes (**Cu24**, **Cu26**, **Cu35**, **Cu345**, **Cu246**)

$[Cu(MeCN)_4]PF_6$ (0.17 mmol, 1 eq.) and xantphos (0.17 mmol, 1 eq.) were added into a dried 100 mL Schlenk tube. The vessel was attached to a reflux cooler and then evacuated and backfilled with argon three times. Degassed dichloromethane (20 mL) was added *via* a syringe. The reaction was refluxed for 16 h. The solution was first cooled to room temperature and then to 0 °C. Under strong stirring, a solution of the ligand (0.17 mmol, 1 eq.) in dry and degassed dichloromethane (15 mL) was added dropwise at 0 °C using a syringe pump (ca. 12 mL h^{-1}). After complete addition, stirring was continued for several minutes at 0 °C followed by 3 h at 45 °C. The heteroleptic target complex was precipitated with *n*-hexane in the Schlenk tube. The solution was stored at +4 °C overnight. The solid precipitate was filtered, washed with *n*-hexane and diethyl ether and dried under vacuum to afford yellow to brownish solids.

Cu24 (79%, $C_{69}H_{60}CuF_6N_2O_5P_3$, M: 1267.71 g/mol): **^{1}H NMR** (CD_3CN, 500 MHz, 25 °C) δ [ppm] = 7.76 (*d*, *J* = 7.9 Hz, 2H, Ar*H*), 7.40 (*s*, 2H, Ar*H*), 7.25 (*m*, 16H, Ar*H*), 7.03 (*m*, 12H, Ar*H*), 6.71 (*m*, 4H, Ar*H*), 3.87 (*s*, 6H, OC*H*$_3$), 3.71 (*s*, 6H, OC*H*$_3$), 2.27 (*s*, 6H, C*H*$_3$), 1.72 (*s*, 6H, C*H*$_3$). **^{13}C{^{1}H} NMR** (CD_3CN, 125 MHz, 25 °C) δ [ppm] = 163.2, 158.8, 148.6, 144.2, 134.4, 133.4, 132.8, 132.3, 131.2, 129.9, 129.8, 129.5, 128.9, 127.6, 127.3, 126.4, 126.0, 124.7, 119.0, 106.5, 99.9, 56.4, 37.0, 28.8, 27.8. **DEPT 135 NMR** (CD_3CN, 125 MHz, 25 °C) δ [ppm] 134.4(+), 134.1(+), 133.9(+), 133.4(+), 132.8(+), 132.3(+), 131.2(+), 129.9(+), 129.5(+), 128.8(+), 127.3(+), 126.4(+), 126.0(+), 124.7(+), 106.5(+), 99.9(+), 56.4(+), 28.8(+), 27.8(+). **^{31}P{^{1}H} NMR** (CD_3CN, 202 MHz, 25 °C) δ [ppm] = -11.8 (*s*, 2P), -143.2 (*sept*, *J* = 706 Hz, PF_6). **HRMS (ESI)** *m/z*: calcd. for [**Cu24**-PF_6]$^+$: 1121.3268, found: 1121.3275. **EA (CHN)** calcd.: 65.37, 4.77, 2.21, found: 64.09, 4.82, 1.62.

Cu26 (77%, $C_{69}H_{60}CuF_6N_2O_5P_3$, M: 1267.71 g/mol): **^{1}H NMR** (CD_3CN, 500 MHz, 25 °C) δ [ppm] = 7.76 (*dd*, J_a = 7.9 Hz, J_b = 1.2 Hz, 2H, Ar*H*), 7.48 (*t*, J_a = 7.9 Hz, J_b = 1.2 Hz , 2H, Ar*H*), 7.39 (*s*, 2H, Ar*H*), 7.26 (*m*, 12H, Ar*H*), 7.11 (*m*, 16H, Ar*H*), 6.93 (*m,* 2H, Ar*H*), 6.81 (*d*, *J* = 8.6 Hz, 4H, Ar*H*), 3.64 (*s*, 12H, OC*H*$_3$), 2.27 (*s*, 6H, C*H*$_3$), 1.73 (*s*, 6H, C*H*$_3$). **^{13}C{^{1}H} NMR** (CD_3CN, 125 MHz, 25 °C) δ [ppm] = 158.6, 156.0, 145.4, 144.3, 135.0, 134.3, 133.9, 132.5, 132.1, 131.2, 130.9, 129.6, 128.7, 128.2, 126.3, 124.4, 122.9, 114.2, 105.4, 56.5, 37.0, 28.6, 27.7. **DEPT 135 NMR** (CD_3CN, 125 MHz, 25 °C) δ [ppm] 133.9(+), 132.1(+), 131.02+), 130.9(+), 129.6(+), 128.7(+), 128.1(+), 126.3(+), 124.4(+), 105.4(+), 56.5(+), 28.6(+), 27.7(+). **^{31}P{^{1}H} NMR** (CD_3CN, 202 MHz, 25 °C) δ [ppm] = -12.1 (*s*, 2P), -143.2 (*sept*, *J* = 706 Hz, PF_6). **HRMS (ESI)** *m/z*: calcd. for [**Cu26**-PF_6]$^+$: 1121.3268, found: 1121.3275. **EA (CHN)** calcd.: 65.37, 4.77, 2.21, found: 64.35, 4.51, 2.05.

Cu35 (93%, $C_{69}H_{60}CuF_6N_2O_5P_3$, M: 1267.71 g/mol): **^{1}H NMR** (CD_3CN, 500 MHz, 25 °C) δ [ppm] = 7.79 (*s*, 2H, Ar*H*), 7.76 (*dd*, J_a = 7.9 Hz, J_b = 1.2 Hz, 2H, Ar*H*), 7.51 (*s*, 2H, Ar*H*), 7.27 (*m*, 8H, Ar*H*), 7.11 (*m*, 16H, Ar*H*), 7.04 (*m,* 2H, Ar*H*), 6.66 (*t*, J_a = 4.6 Hz, J_b = 2.4 Hz, 2H, Ar*H*), 6.62 (*d*, 4H, Ar*H*), 3.83 (*s*, 12H, OC*H*$_3$), 2.30 (*s*, 6H, C*H*$_3$), 1.72 (*s*, 6H, C*H*$_3$). **^{13}C{^{1}H} NMR** (CD_3CN, 125 MHz, 25 °C) δ [ppm] = 162.1, 158.9, 155.8, 150.5, 144.3, 139.7, 134.9, 134.3, 133.9, 132.5, 131.2, 130.9, 129.5, 128.9, 126.4, 126.3, 124.3, 122.6, 108.7, 101.4, 56.3, 36.9, 28.8, 27.8. **DEPT 135 NMR** (CD_3CN, 125 MHz, 25 °C) δ [ppm] 134.0(+), 131.2(+), 131.0(+), 129.6(+), 128.9(+), 126.4(+), 126.4(+), 124.3(+), 108.7(+), 101.5(+), 56.3(+), 28.9(+), 27.9(+). **^{31}P{^{1}H} NMR** (CD_3CN, 202 MHz, 25 °C) δ [ppm] = -11.6 (*s*, 2P), -143.2 (*sept*, *J* = 706 Hz, PF_6). **HRMS (ESI)** *m/z*: calcd. for [**Cu35**-PF_6]$^+$: 1121.3268, found: 1121.3277. **EA (CHN)** calcd.: 65.37, 4.77, 2.21, found: 64.73, 4.44, 2.14.

Cu345 (93%, $C_{71}H_{64}CuF_6N_2O_7P_3$, M: 1327.76 g/mol): **^{1}H NMR** (CD_3CN, 400 MHz, 25 °C) *δ* [ppm] = 7.88 (*s*, 2H, Ar*H*), 7.77 (*dd*, *J* = 8.0 Hz, *J* = 1.3 Hz, 2H, Ar*H*), 7.44 (*s*, 2H, Ar*H*), 7.55 (*s*, 2H, Ar*H*), 7.28 (*m*, 8H, Ar*H*), 7.12 (*m*, 16H, Ar*H*), 7.05 (*m,* 2H, Ar*H*), 6.78 (*s*, 4H, Ar*H*), 3.87 (*s*, 12H, OC*H*3), 3.82 (*s*, 6H, OC*H*3), 2.32 (*s*, 6H, C*H*3), 1.73 (*s*, 6H, C*H*3). **^{13}C{^{1}H} NMR** (CD_3CN, 100 MHz, 25 °C) *δ* [ppm] = 158.9, 155.9, 154.6, 150.6, 144.5, 139.7, 134.9, 134.3, 134.0, 133.1, 132.5, 131.2, 130.9, 129.6, 128.9, 126.5, 126.4, 126.3, 124.4, 122.6, 108.2, 61.0, 57.0, 36.9, 28.9, 27.8. **DEPT 135 NMR** (CD_3CN, 100 MHz, 25 °C) *δ* [ppm] 133.9(+), 131.2(+), 130.9(+), 129.5(+), 128.7(+), 126.4(+), 126.3(+), 124.3(+), 108.1(+), 61.0(+), 56.9(+), 28.8(+), 27.8(+). **^{31}P{^{1}H} NMR** (CD_3CN, 162 MHz, 25 °C) *δ* [ppm] = -11.64 (*s*, 2P), -143.2 (*sept*, *J* = 706 Hz, PF_6). **HRMS (ESI)** *m/z*: calcd. for [**Cu345**-PF_6]$^+$: 1181.3479, found: 1181.3490. **EA (CHN)** calcd.: 64.23, 4.86, 2.11, found: 62.87, 4.80, 2.04.

Cu246 (82%, $C_{71}H_{64}CuF_6N_2O_7P_3$, M: 1327.76 g/mol): **^{1}H NMR** (CD_3CN, 500 MHz, 25 °C) *δ* [ppm] = 7.76 (*dd*, *J* = 7.9 Hz, *J* = 1.2 Hz, 2H, Ar*H*), 7.37 (*s*, 2H, Ar*H*), 7.30 (*s*, 2H, Ar*H*), 7.27 (*m*, 3H, Ar*H*), 7.24 (*t*, *J* = 14.0 Hz, *J* = 6.9 Hz, 3H, Ar*H*) 7.10 (*m*, 16H, Ar*H*), 6.91 (*m,* 2H, Ar*H*), 6.37 (*s*, 4H, Ar*H*), 3.88 (*s*, 6H, OC*H*3), 3.64 (*s*, 12H, OC*H*3), 2.25 (*s*, 6H, C*H*3), 1.72 (*s*, 6H, C*H*3). **^{13}C{^{1}H} NMR** (CD_3CN, 125 MHz, 25 °C) *δ* [ppm] = 163.6, 159.4, 158.4, 156.0, 145.5, 144.4, 135.0, 133.9, 132.6, 131.2, 130.9, 129.6, 128.7, 128.5, 128.5, 126.3, 124.4, 122.9, 106.9, 92.1, 56.5, 56.3, 36.9, 28.6, 27.6. **DEPT 135 NMR** (CD_3CN, 125 MHz, 25 °C) *δ* [ppm] 133.8(+), 131.1(+), 130.9(+), 129.6(+), 128.6(+), 128.5(+), 126.3(+), 124.4(+), 92.0(+), 56.5(+), 56.3(+), 28.6(+), 27.6(+). **^{31}P{^{1}H} NMR** (CD_3CN, 202 MHz, 25 °C) *δ* [ppm] = -12.2 (*s*, 2P), -143.2 (*sept*, *J* = 706 Hz, PF_6). **HRMS (ESI)** *m/z*: calcd. for [**Cu246**-PF_6]$^+$: 1181.3479, found: 1181.3491. **EA (CHN)** calcd.: 64.23, 4.86, 2.11, found: 63.53, 4.88, 2.11.

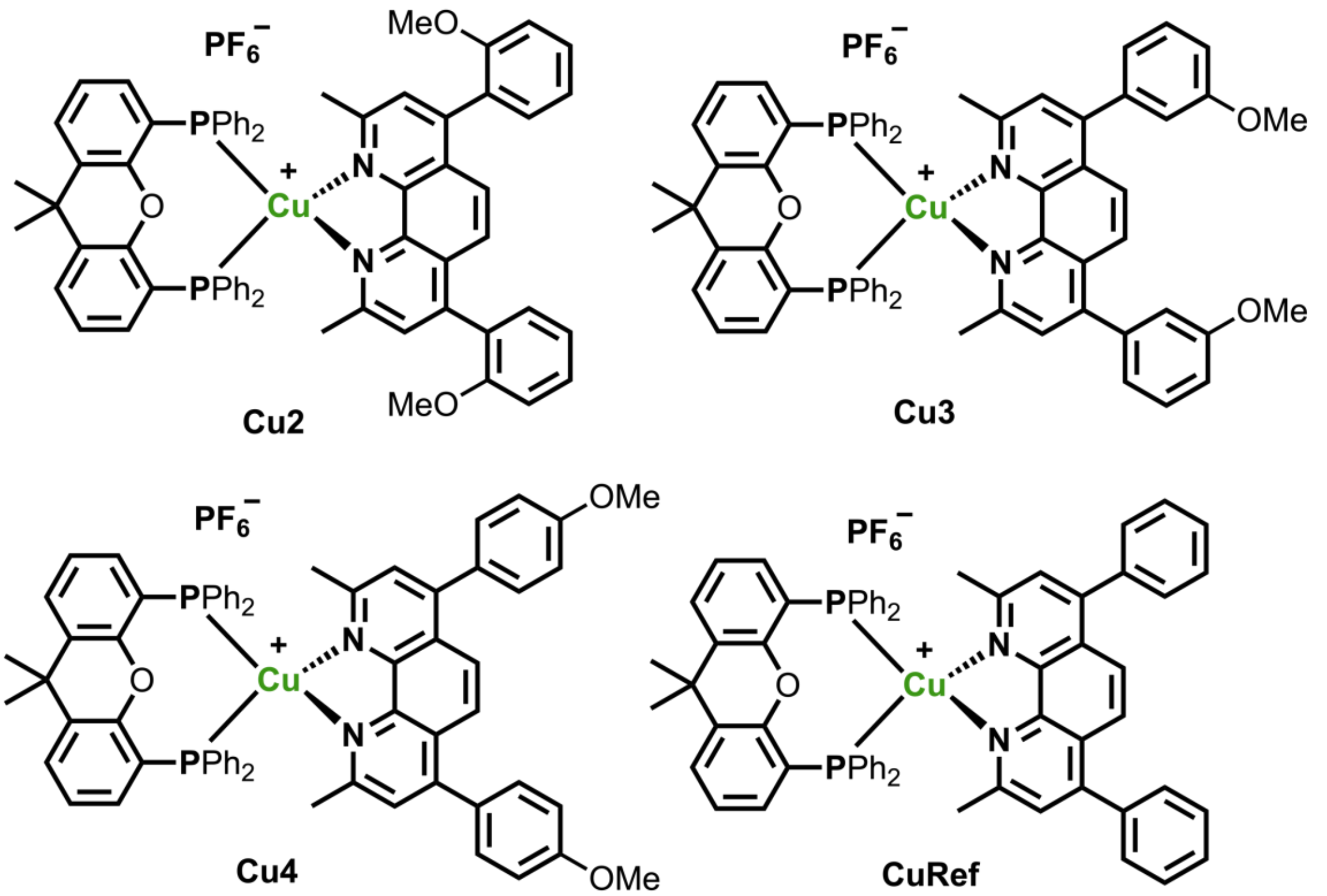

Figure S2.2. Overview of the reference complexes bearing single methoxy groups at *ortho*- (**Cu2**), *meta*-(**Cu3**) and *para*- (**Cu4**) position as well as the unsubstituted bathocuproine complex **CuRef**.

3 NMR Spectroscopy

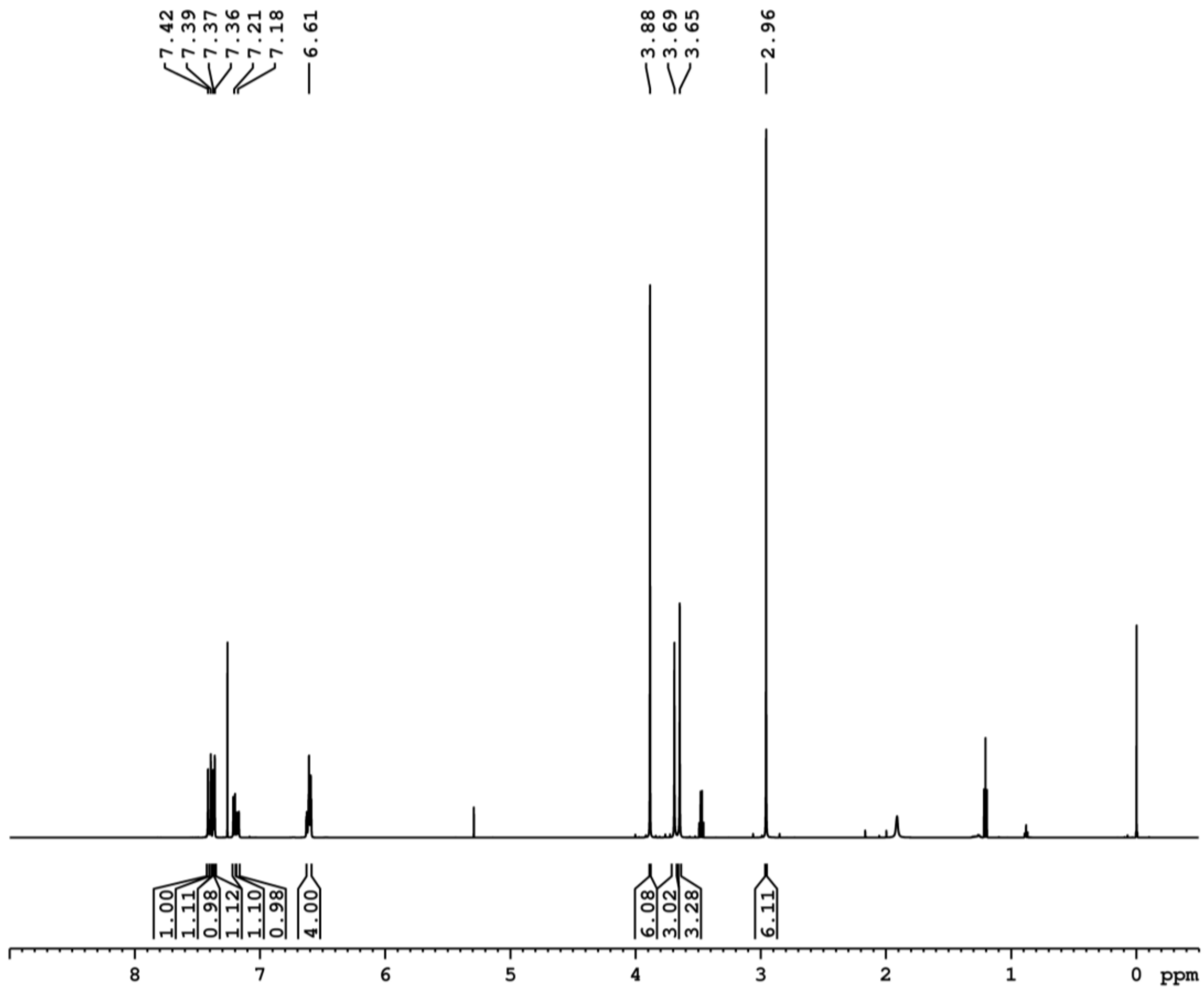

Figure S3.1. ^{1}H NMR of **L24** in $CDCl_3$ (600 MHz).

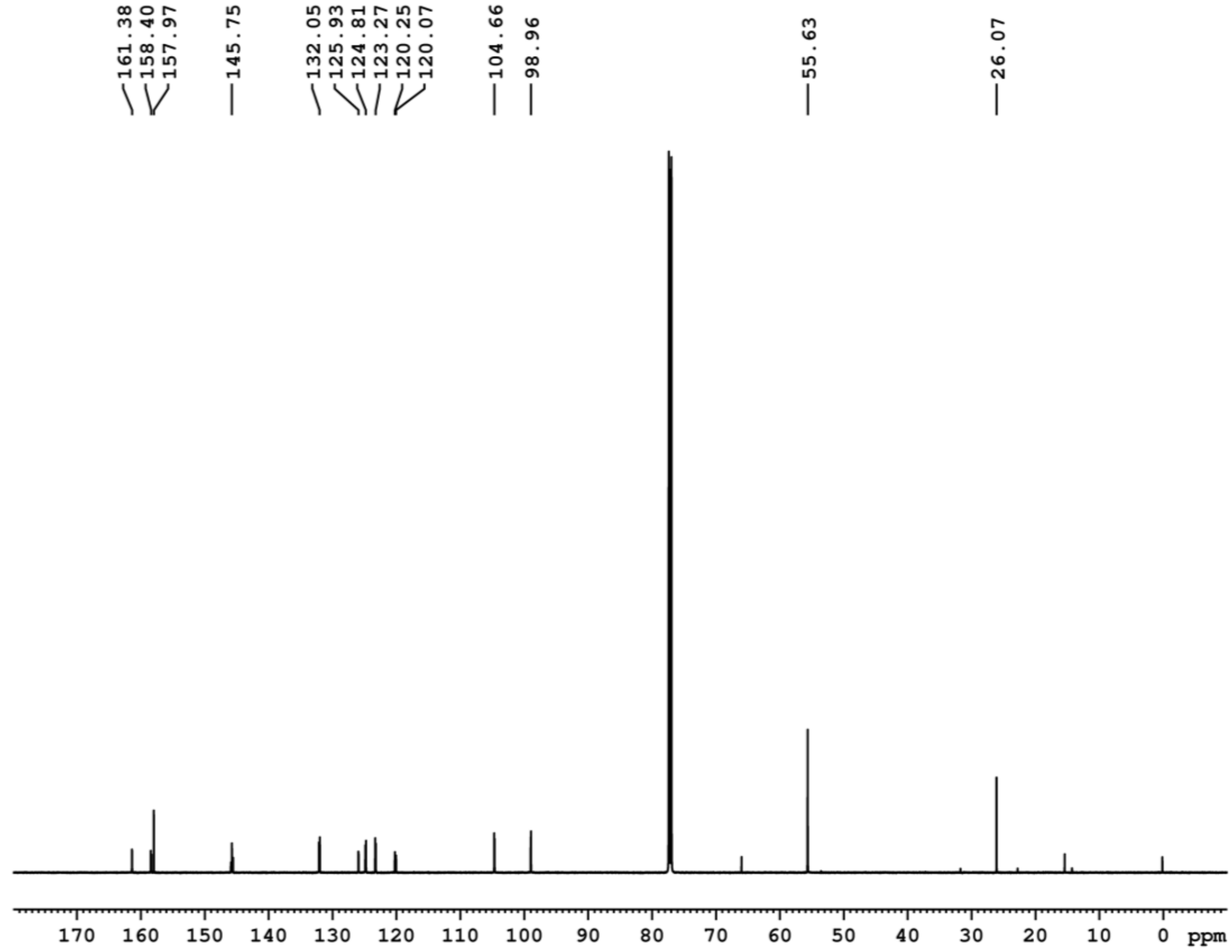

Figure S3.2. $^{13}C\{^{1}H\}$ NMR of **L24** in $CDCl_3$ (150 MHz).

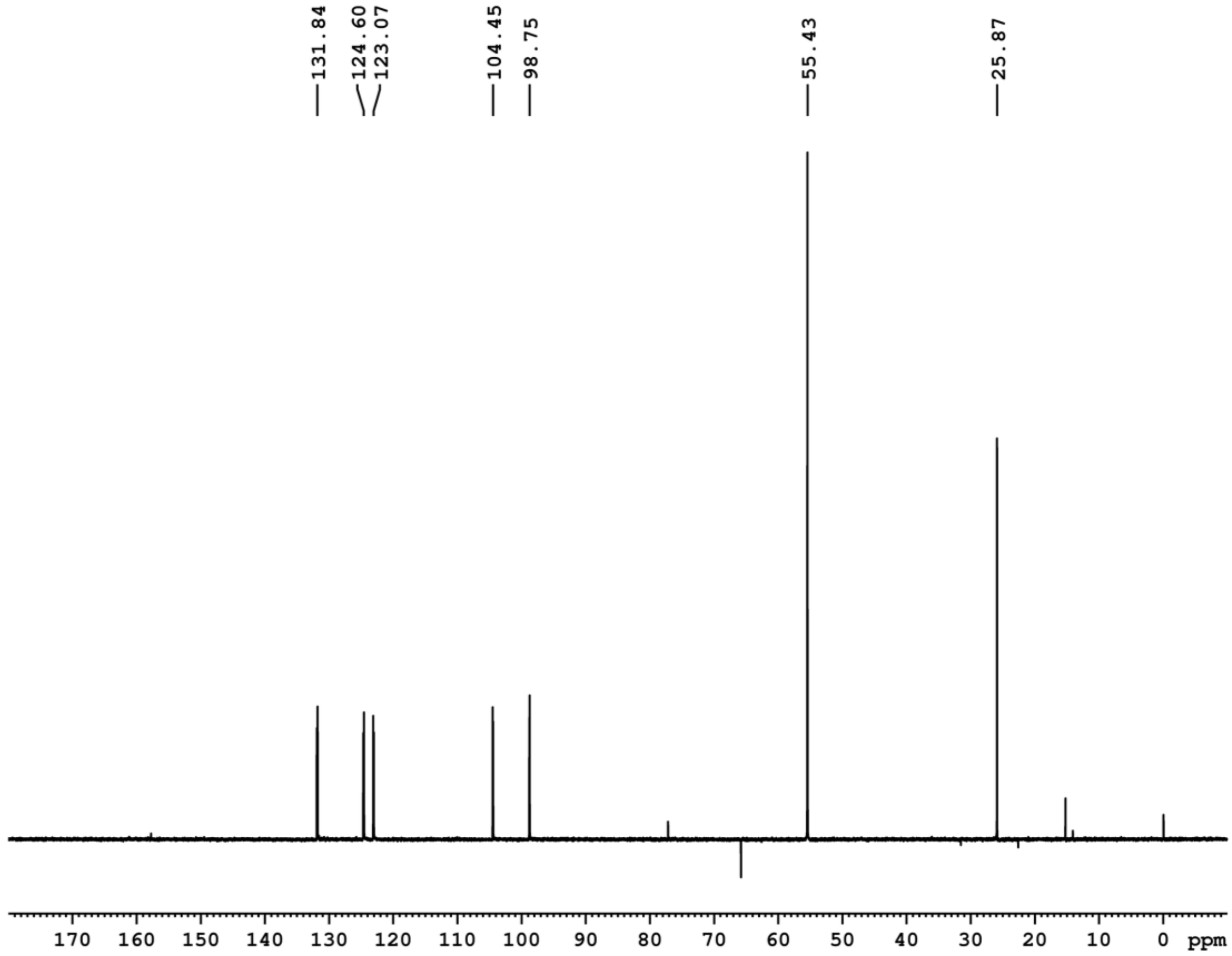

Figure S3.3. DEPT-135 NMR of **L24** in $CDCl_3$ (150 MHz).

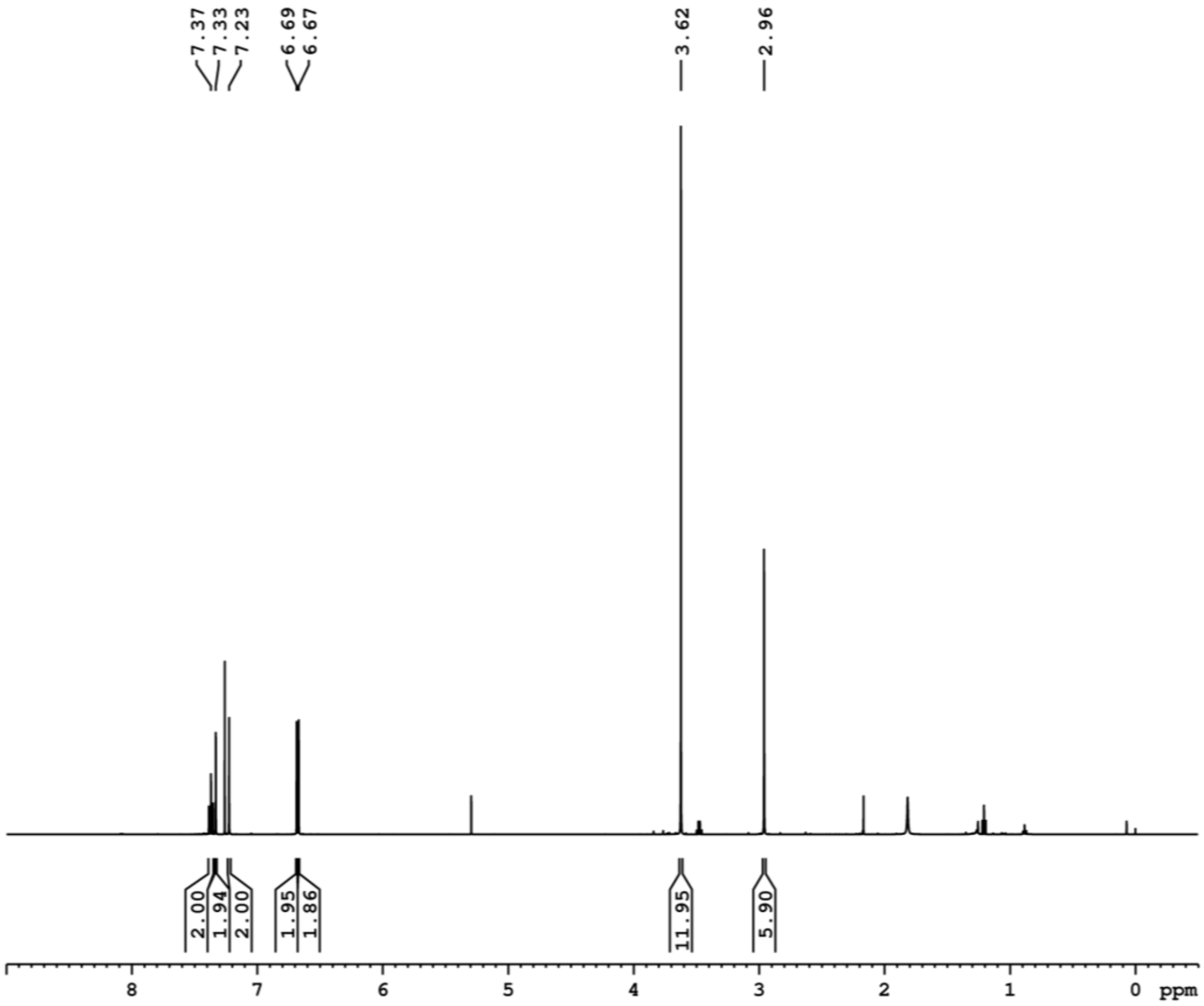

Figure S3.4. ^{1}H NMR of **L26** in $CDCl_3$ (500 MHz).

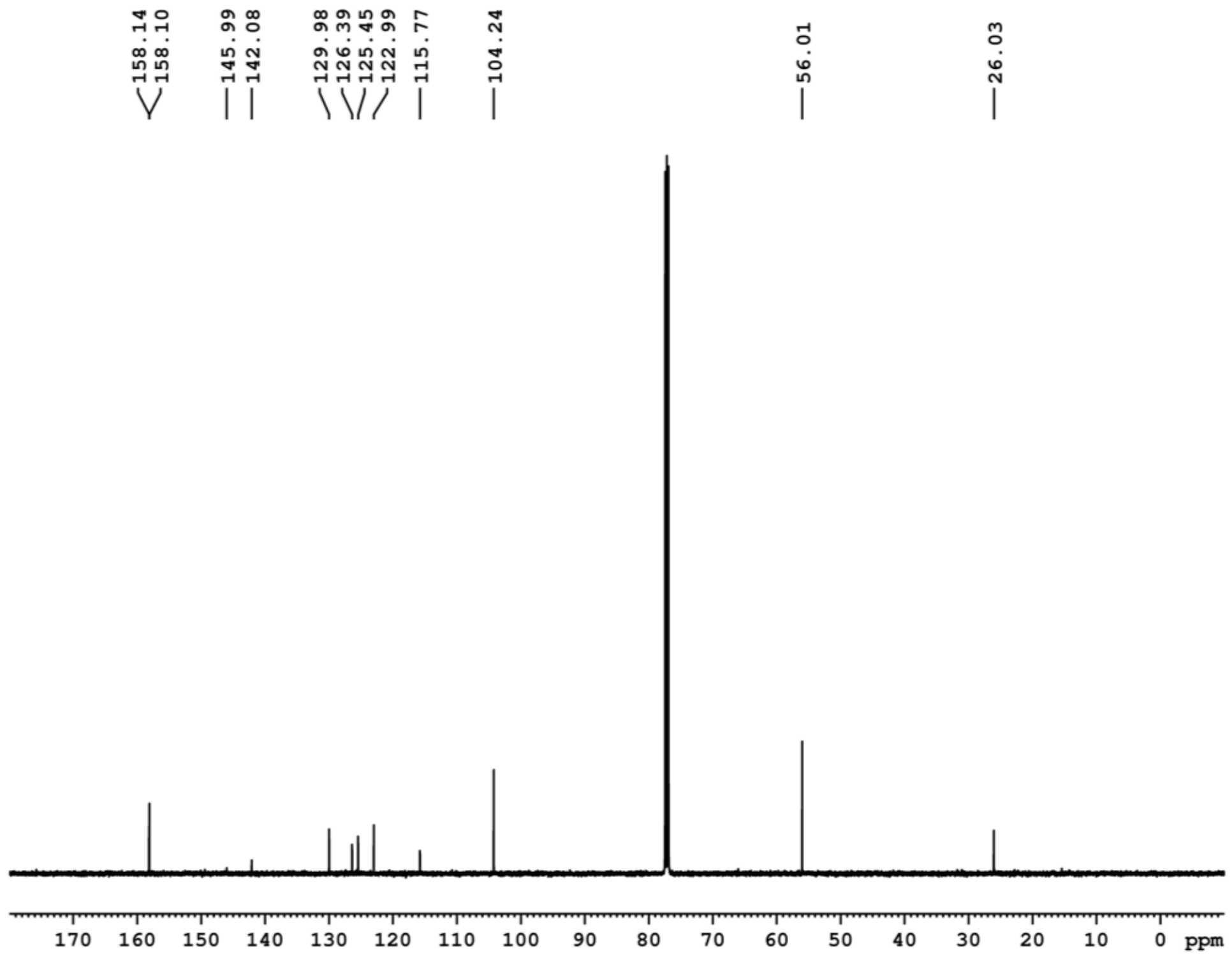

Figure S3.5. $^{13}C\{^{1}H\}$ NMR of **L26** in $CDCl_3$ (125 MHz).

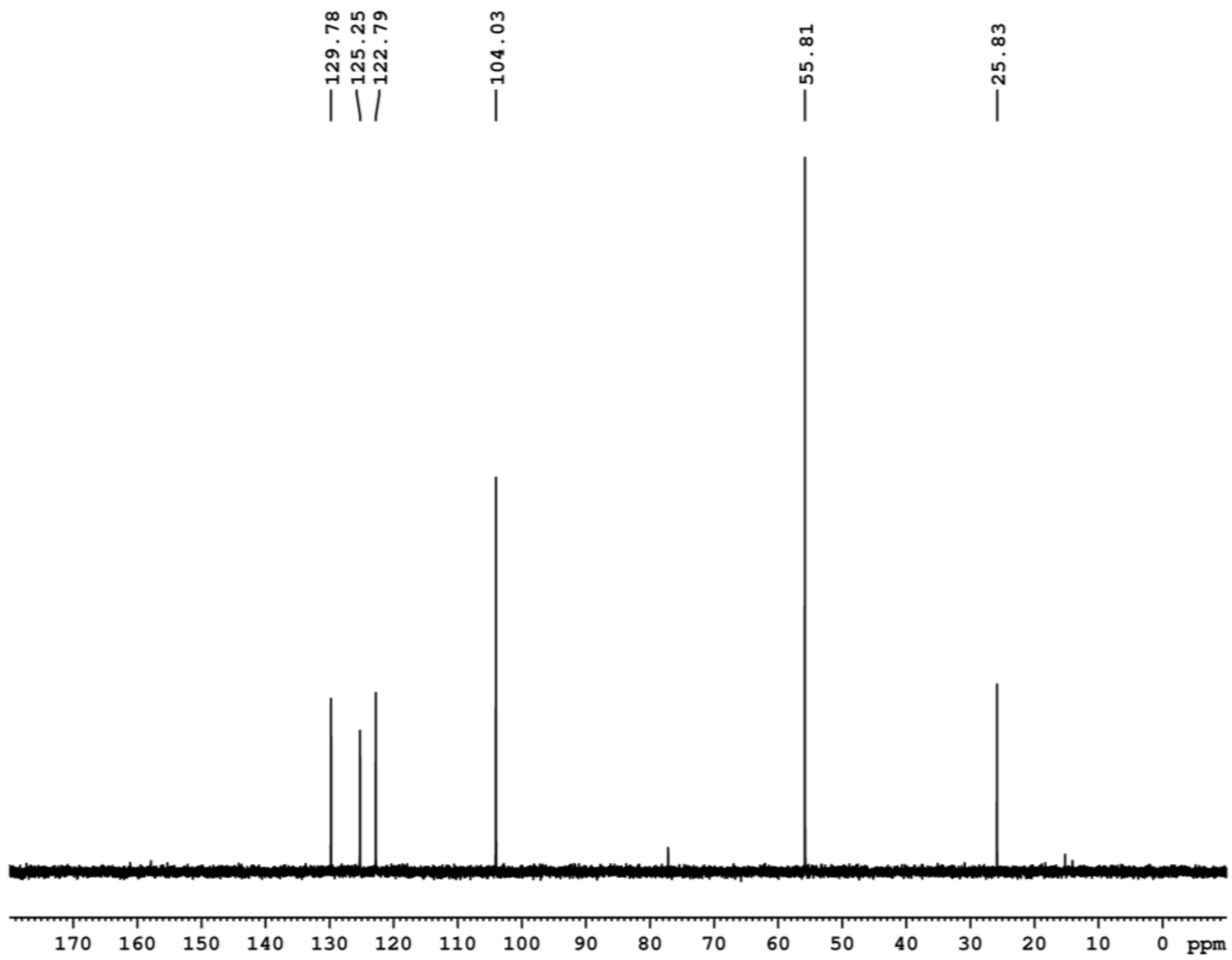

Figure S3.6. DEPT 135 NMR of **L26** in $CDCl_3$ (125 MHz).

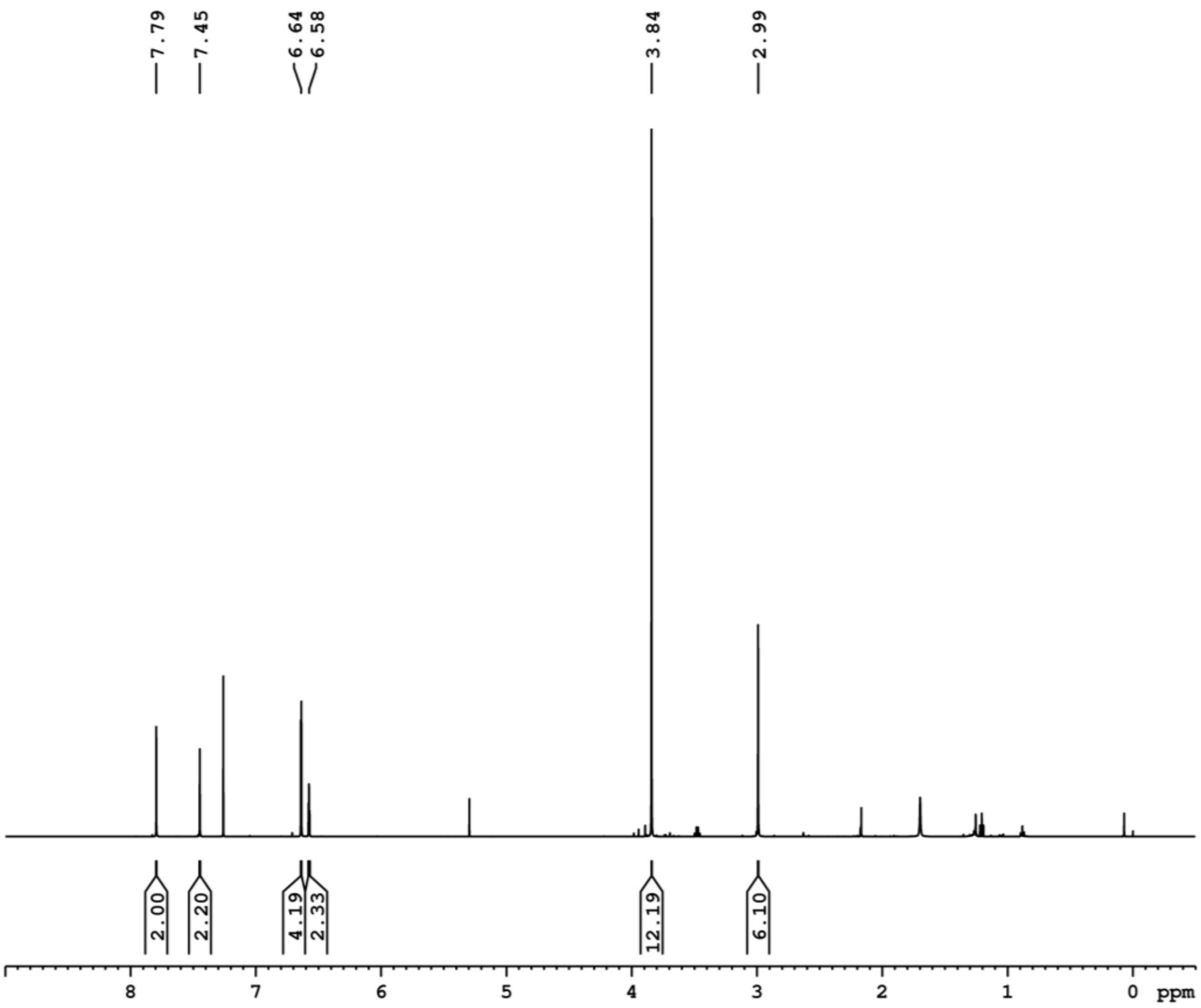

Figure S3.7. ^{1}H NMR of **L35** in $CDCl_3$ (500 MHz).

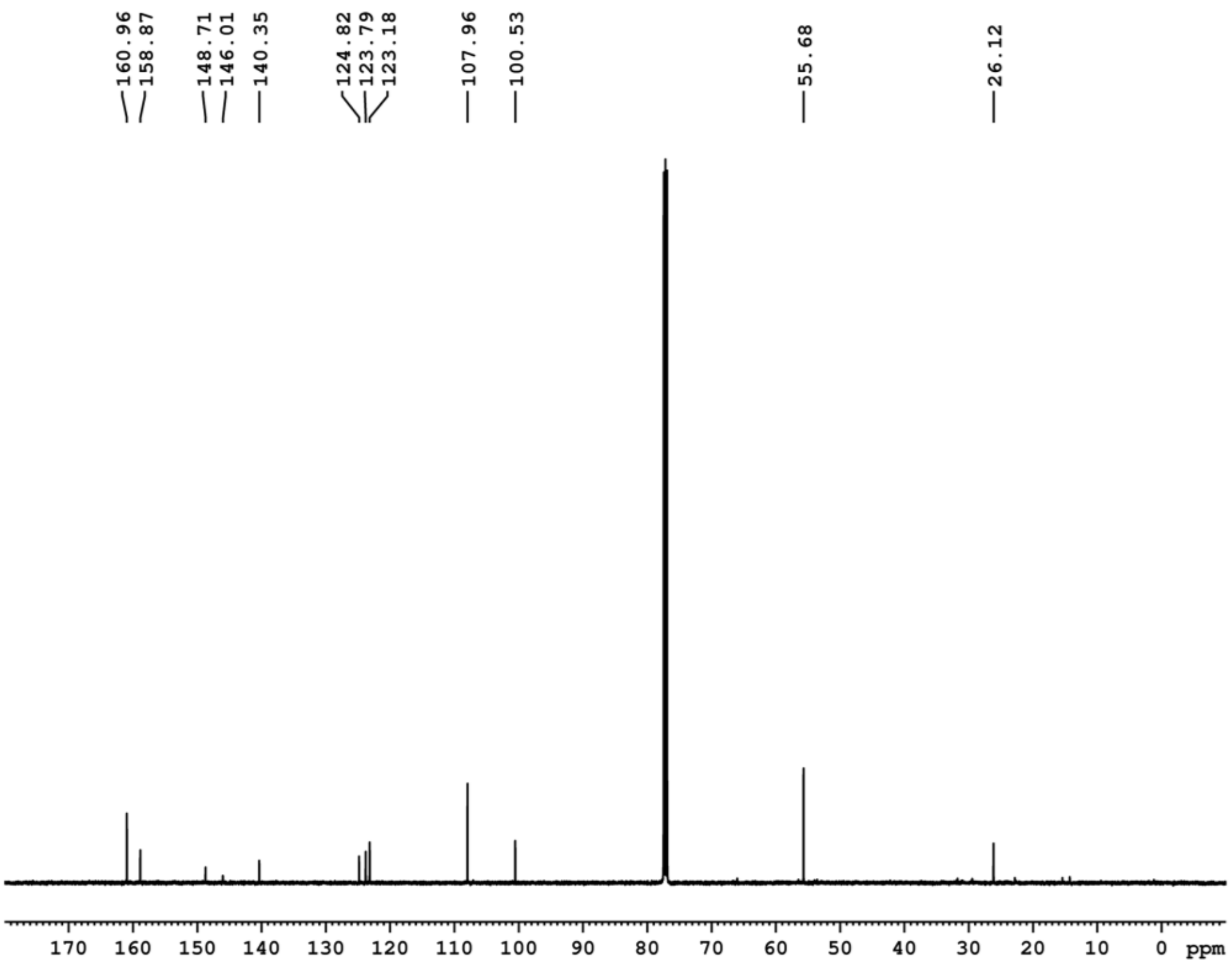

Figure S3.8. $^{13}C\{^1H\}$ NMR of **L35** in $CDCl_3$ (125 MHz).

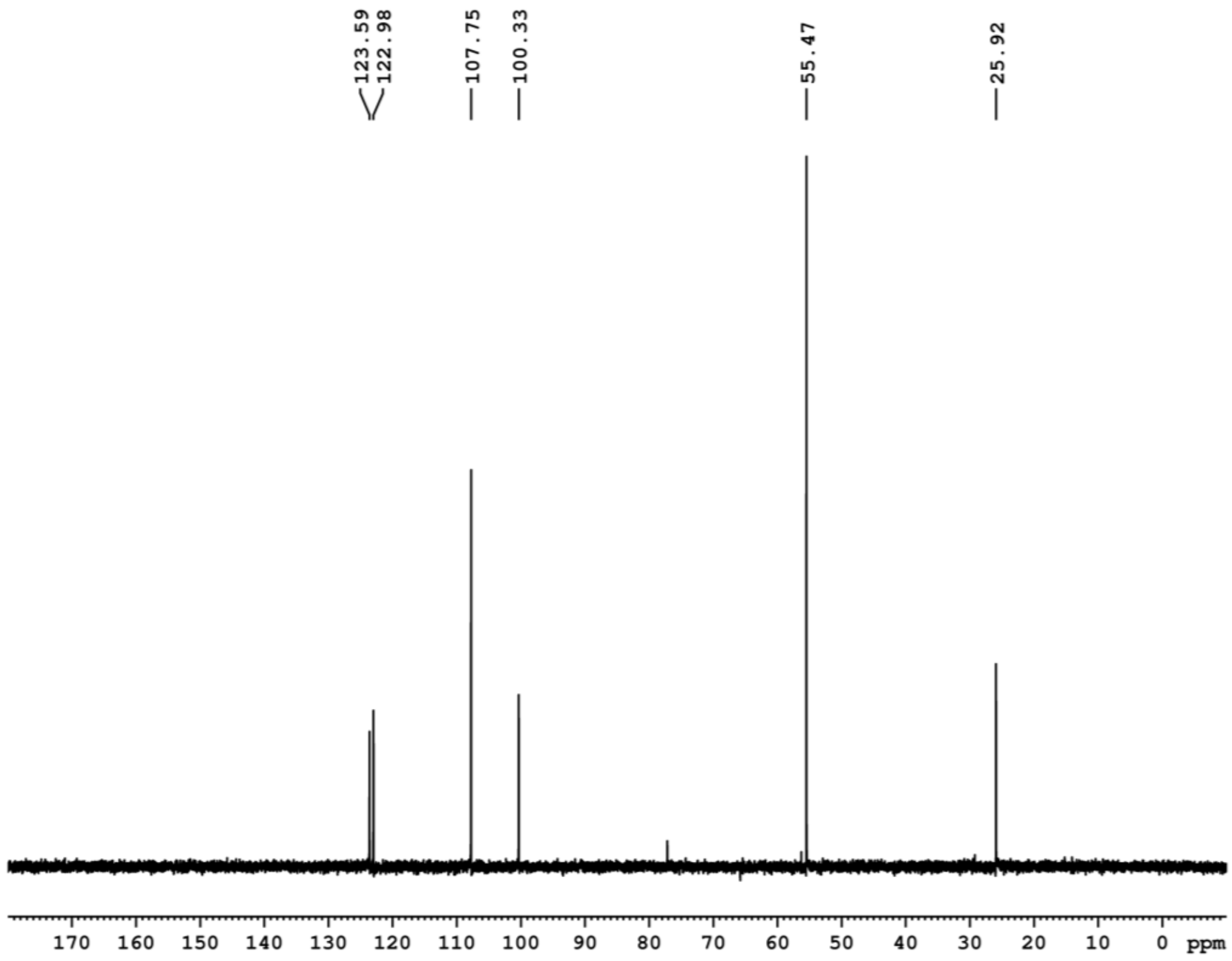

Figure S3.9. DEPT 135 NMR of **L35** in $CDCl_3$ (125 MHz).

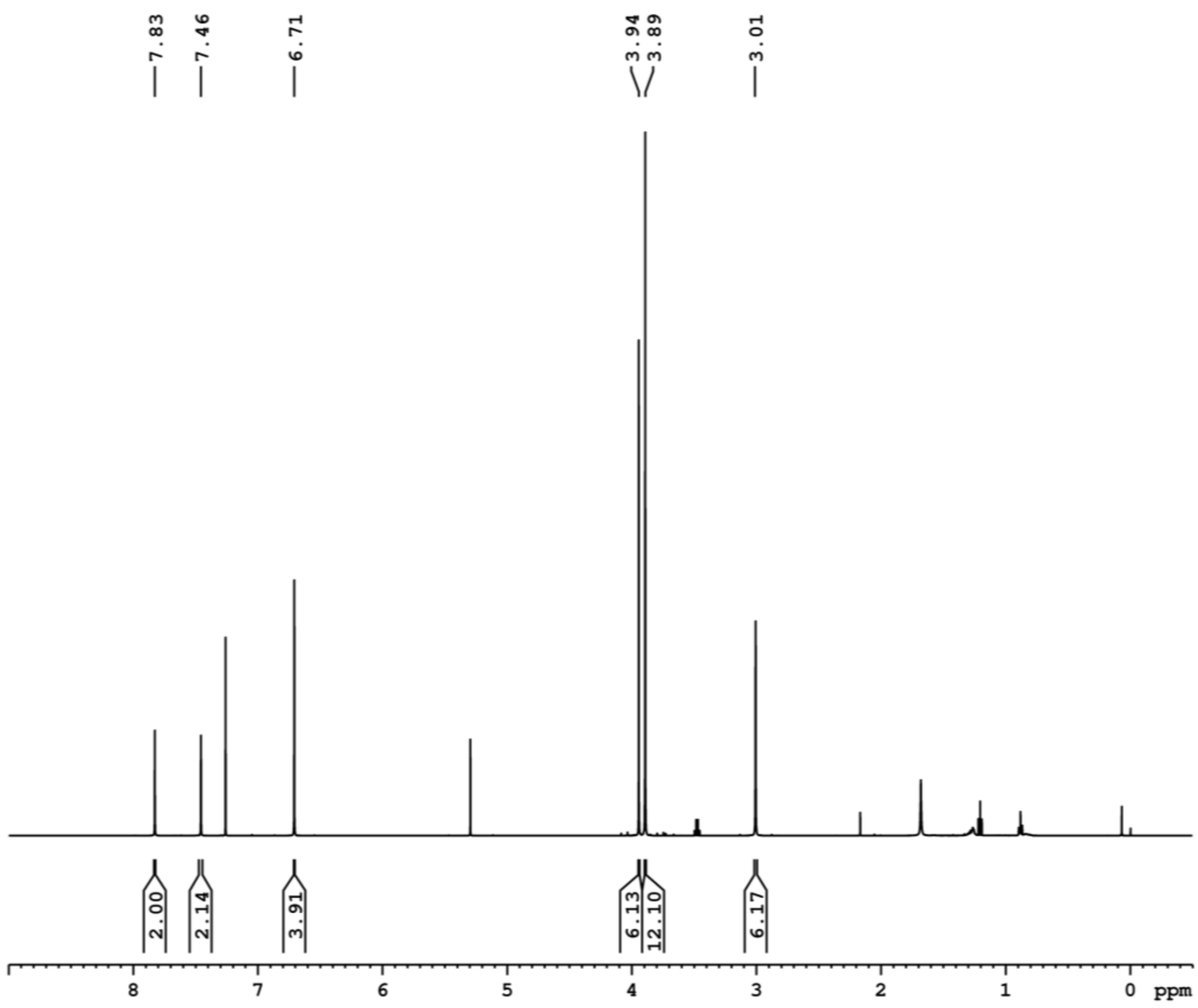

Figure S3.10. ^1H NMR of **L345** in $CDCl_3$ (500 MHz).

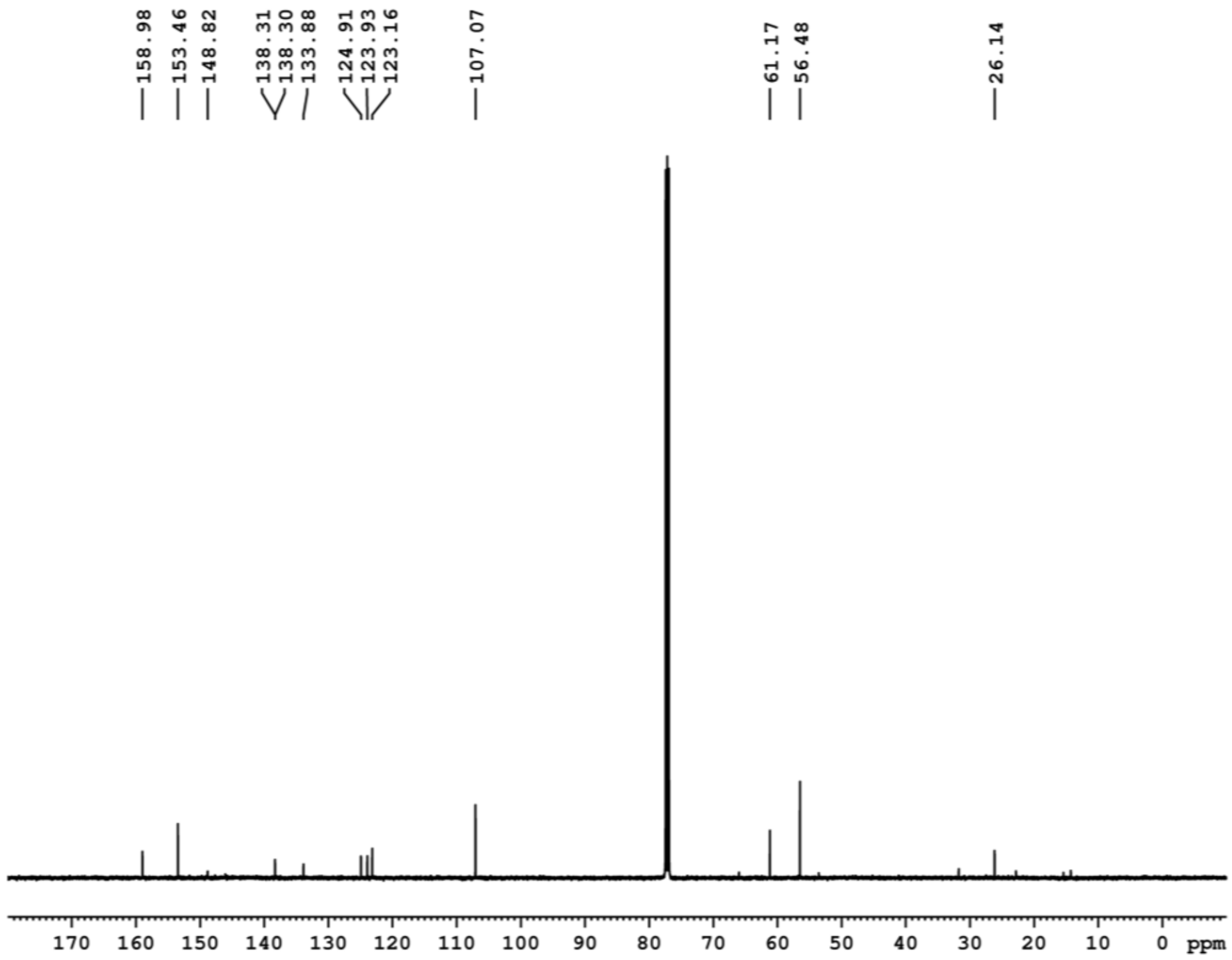

Figure S3.11. $^{13}C\{^{1}H\}$ NMR of **L345** in $CDCl_3$ (125 MHz).

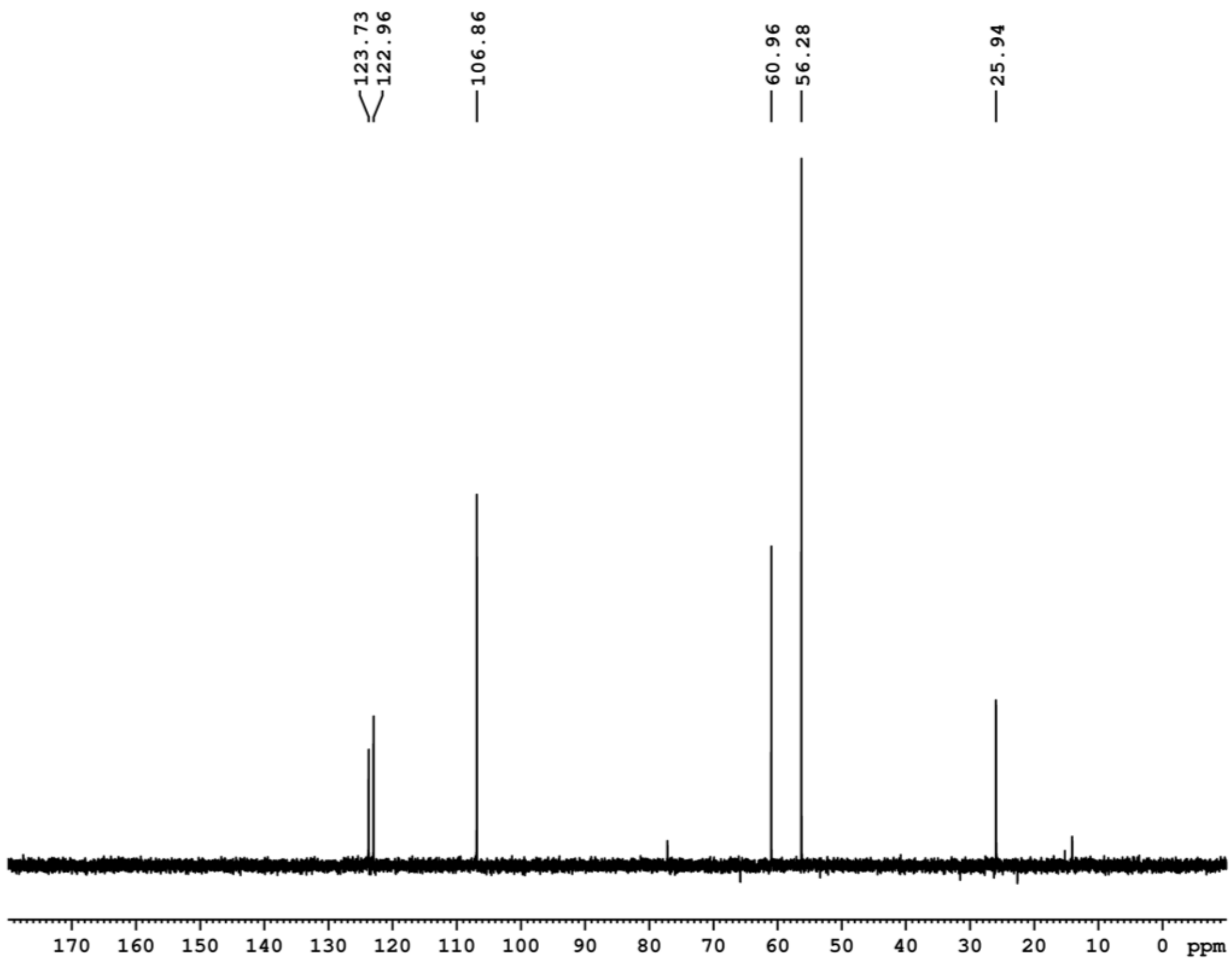

Figure S3.12. DEPT-135 NMR of **L345** in $CDCl_3$ (125 MHz).

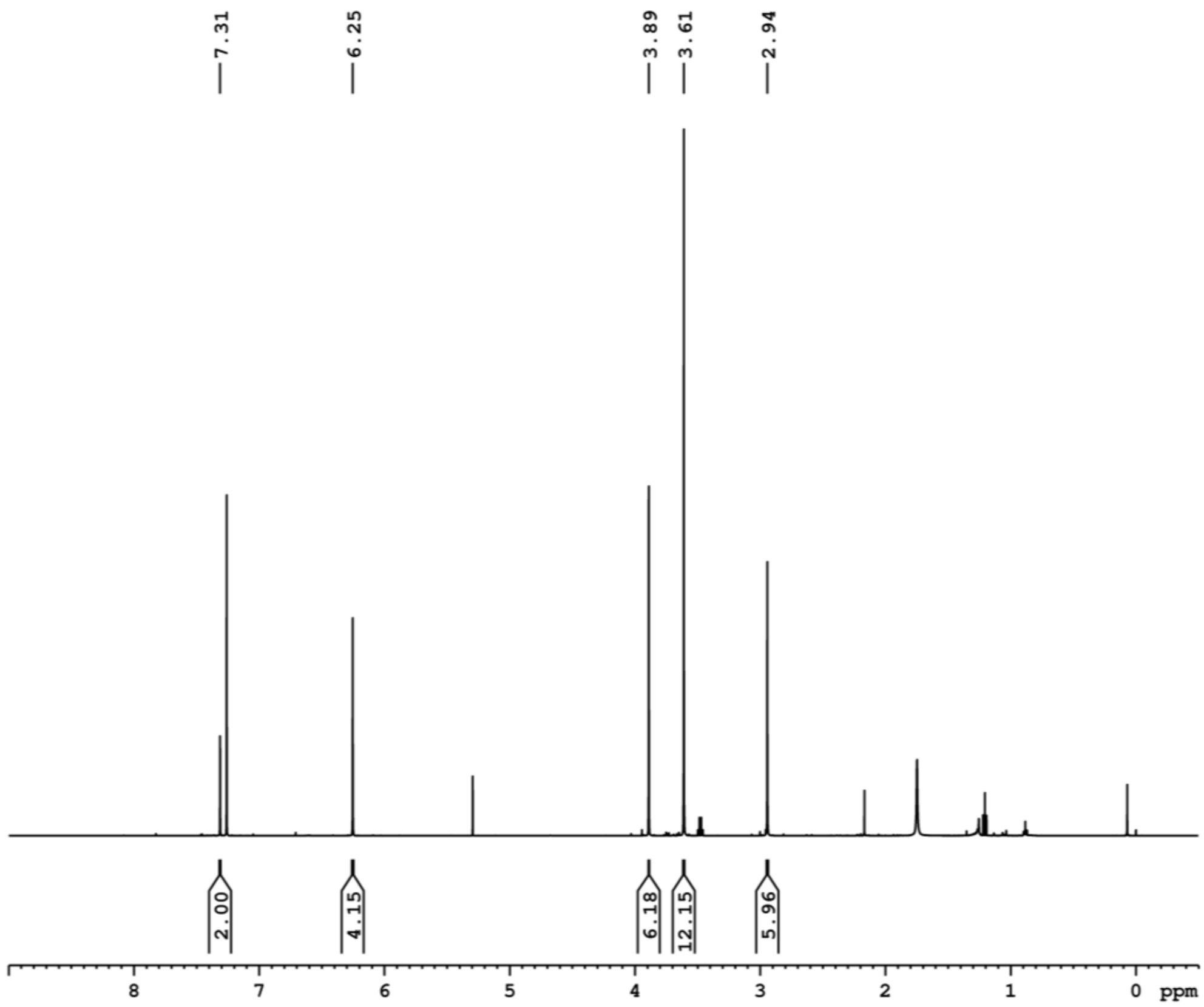

Figure S3.13. ^{1}H NMR of **L246** in $CDCl_3$ (500 MHz).

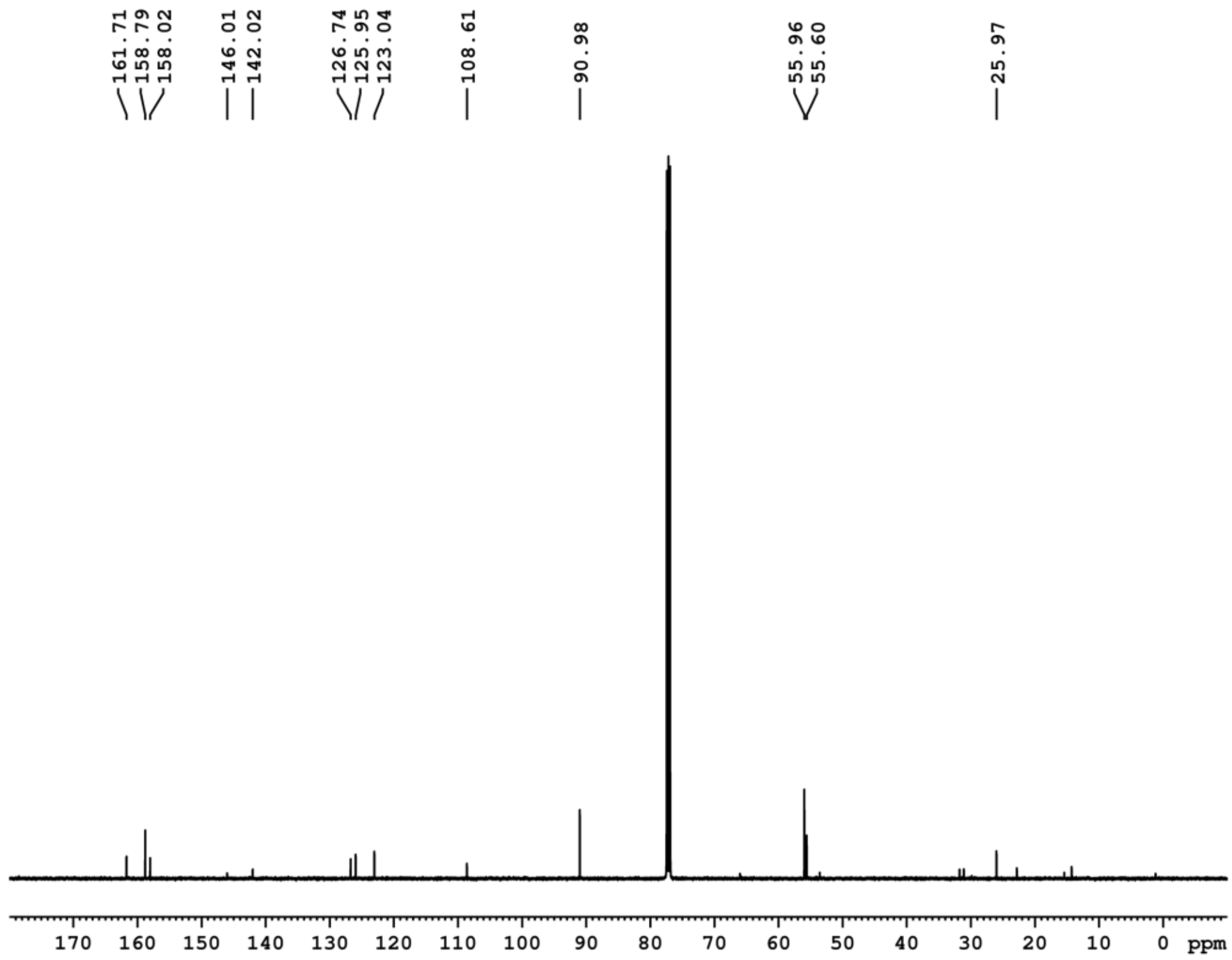

Figure S3.14. $^{13}C\{^{1}H\}$ NMR of **L246** in $CDCl_3$ (125 MHz).

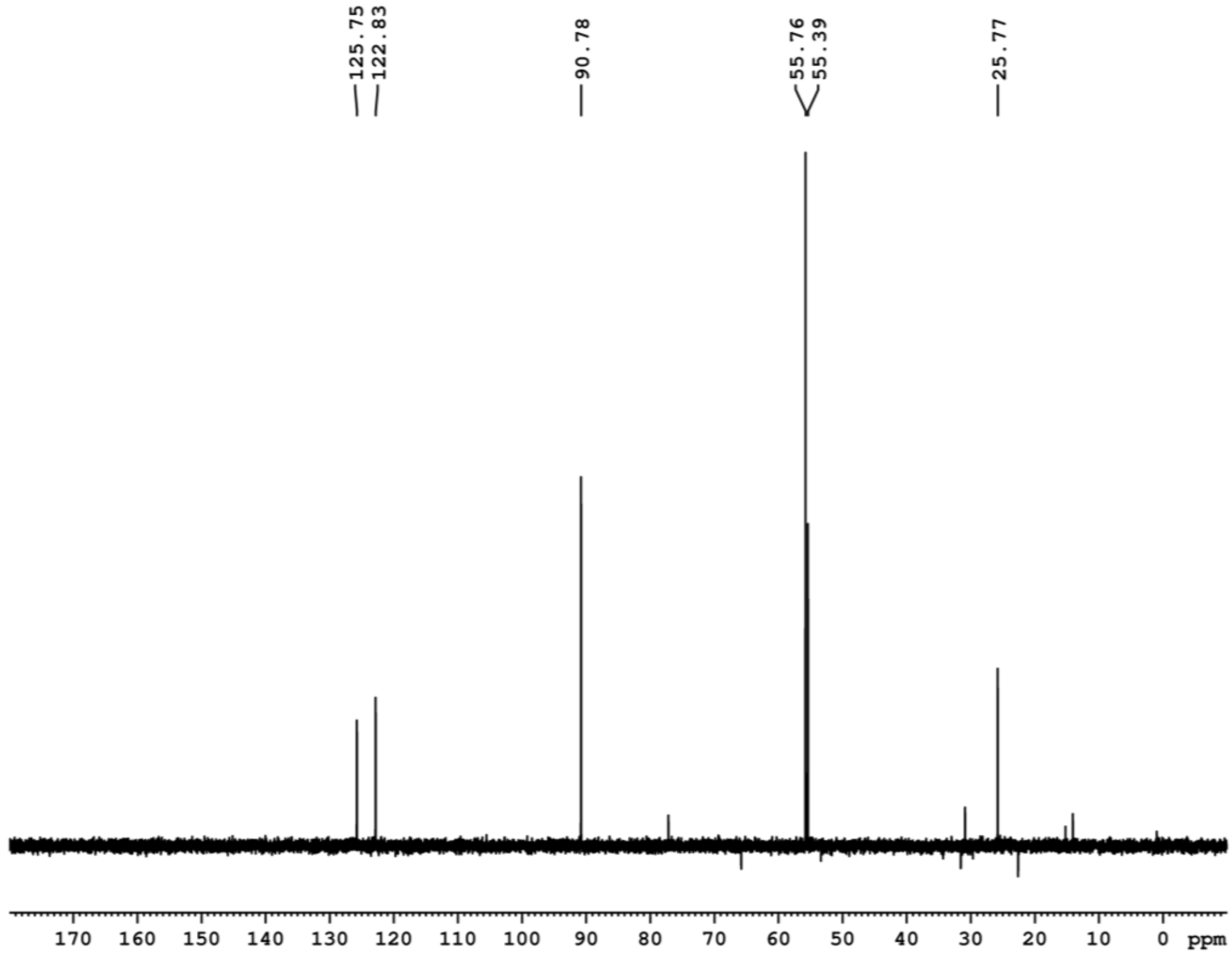

Figure S3.15. DEPT-135 NMR of **L246** in $CDCl_3$ (125 MHz).

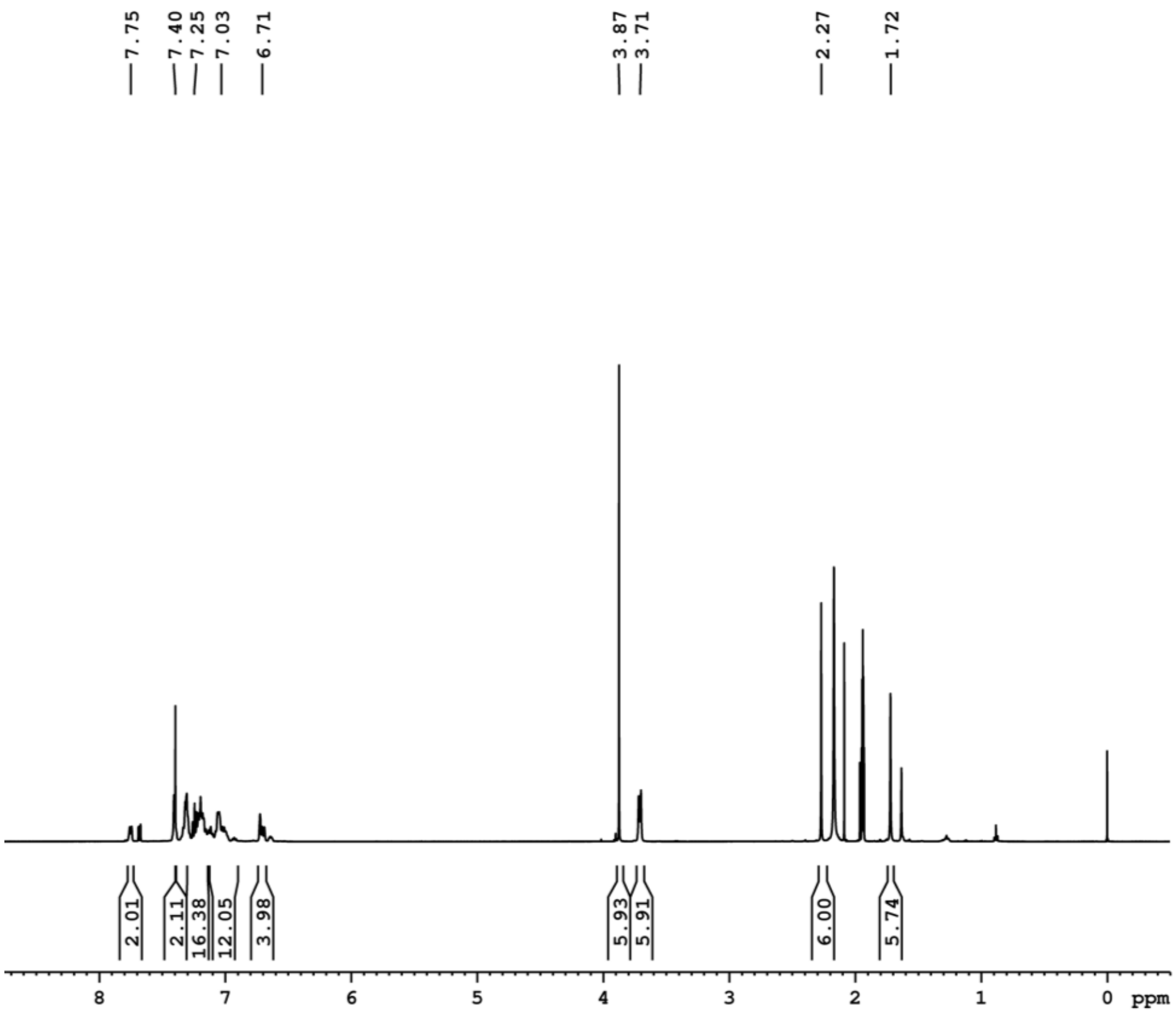

Figure S3.16. ^{1}H NMR of **Cu24** in CD_3CN (500 MHz).

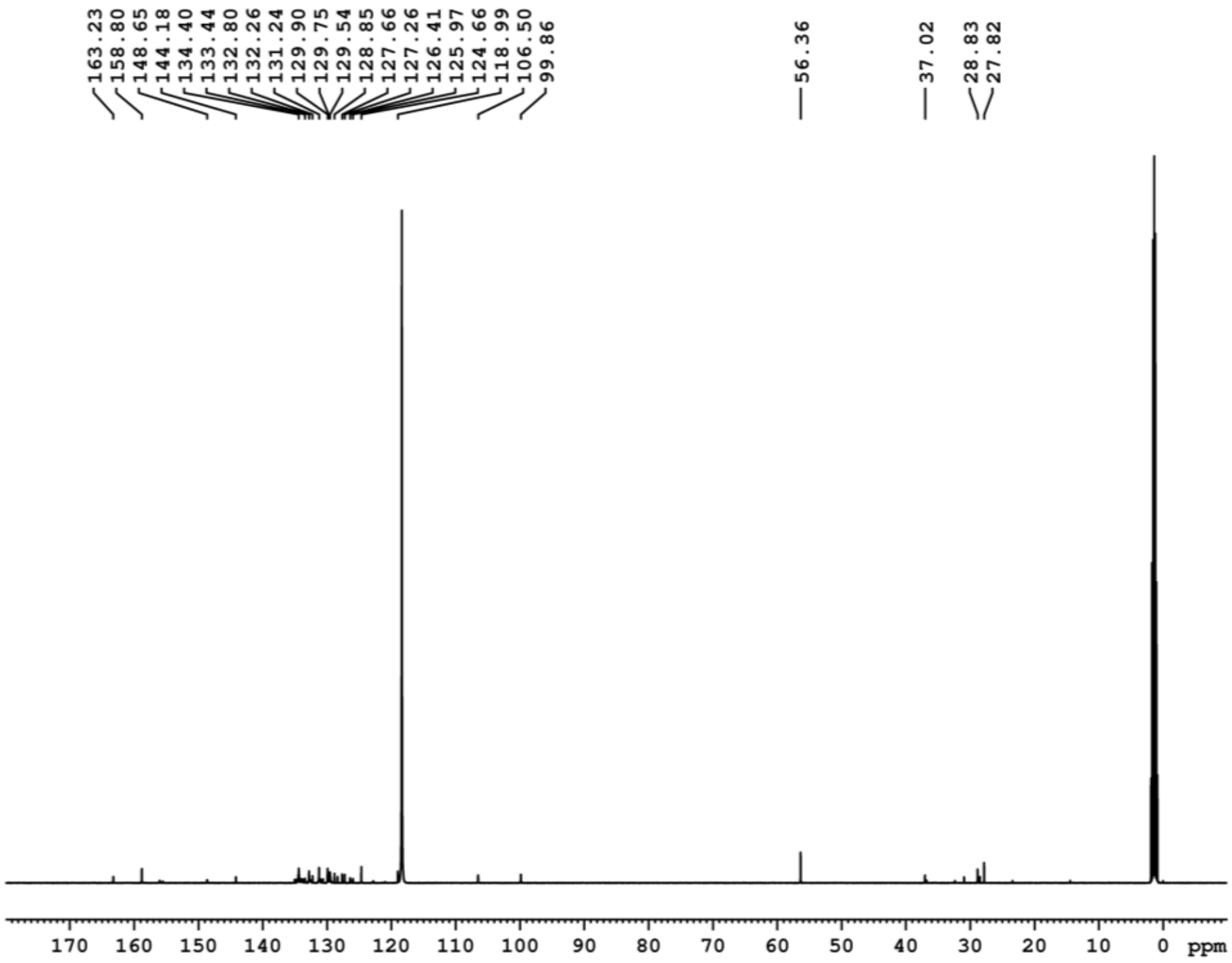

Figure S3.17. $^{13}C\{^{1}H\}$ NMR of **Cu24** in CD_3CN (125 MHz).

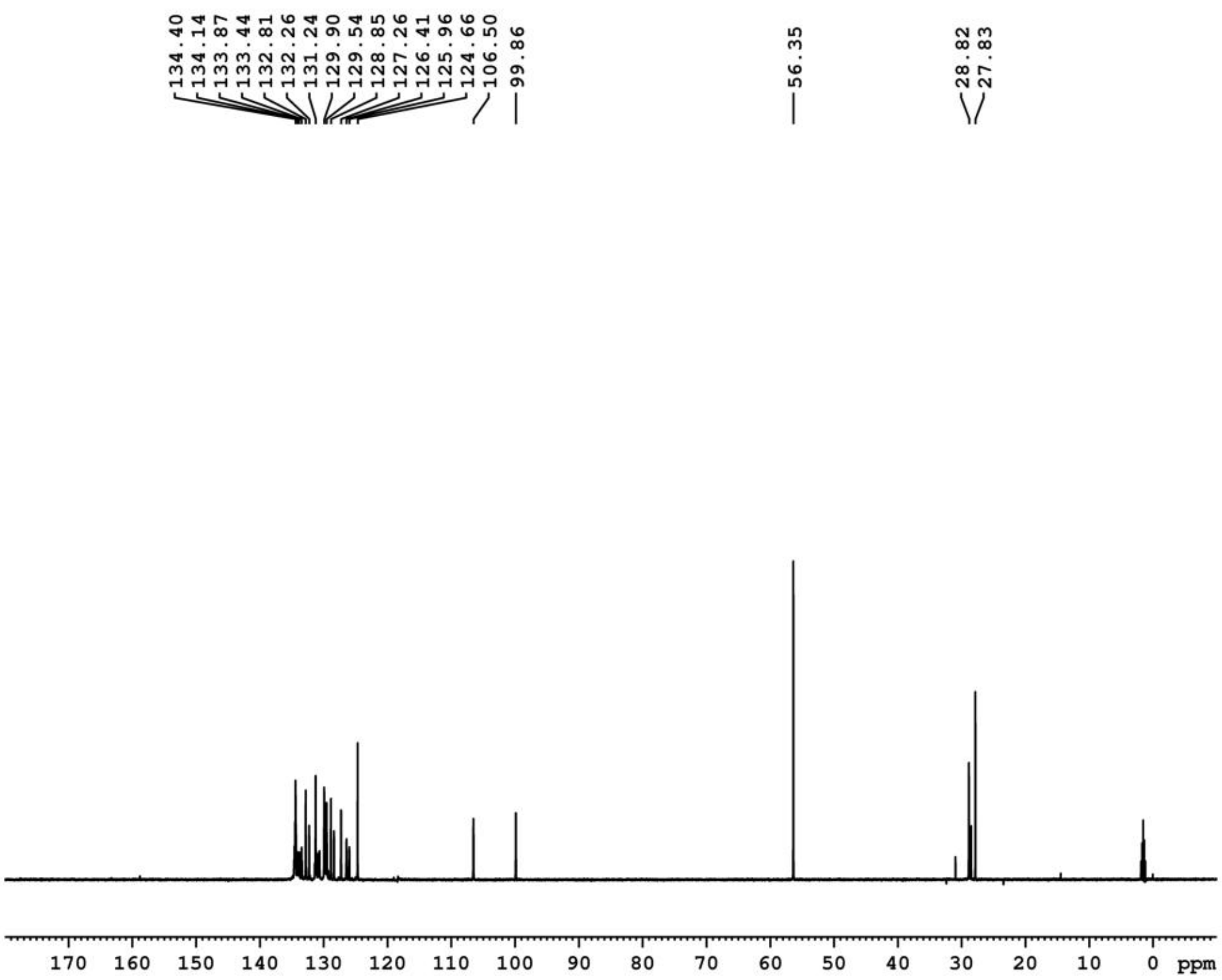

Figure S3.18. DEPT-135 NMR of **Cu24** in CD_3CN (125 MHz).

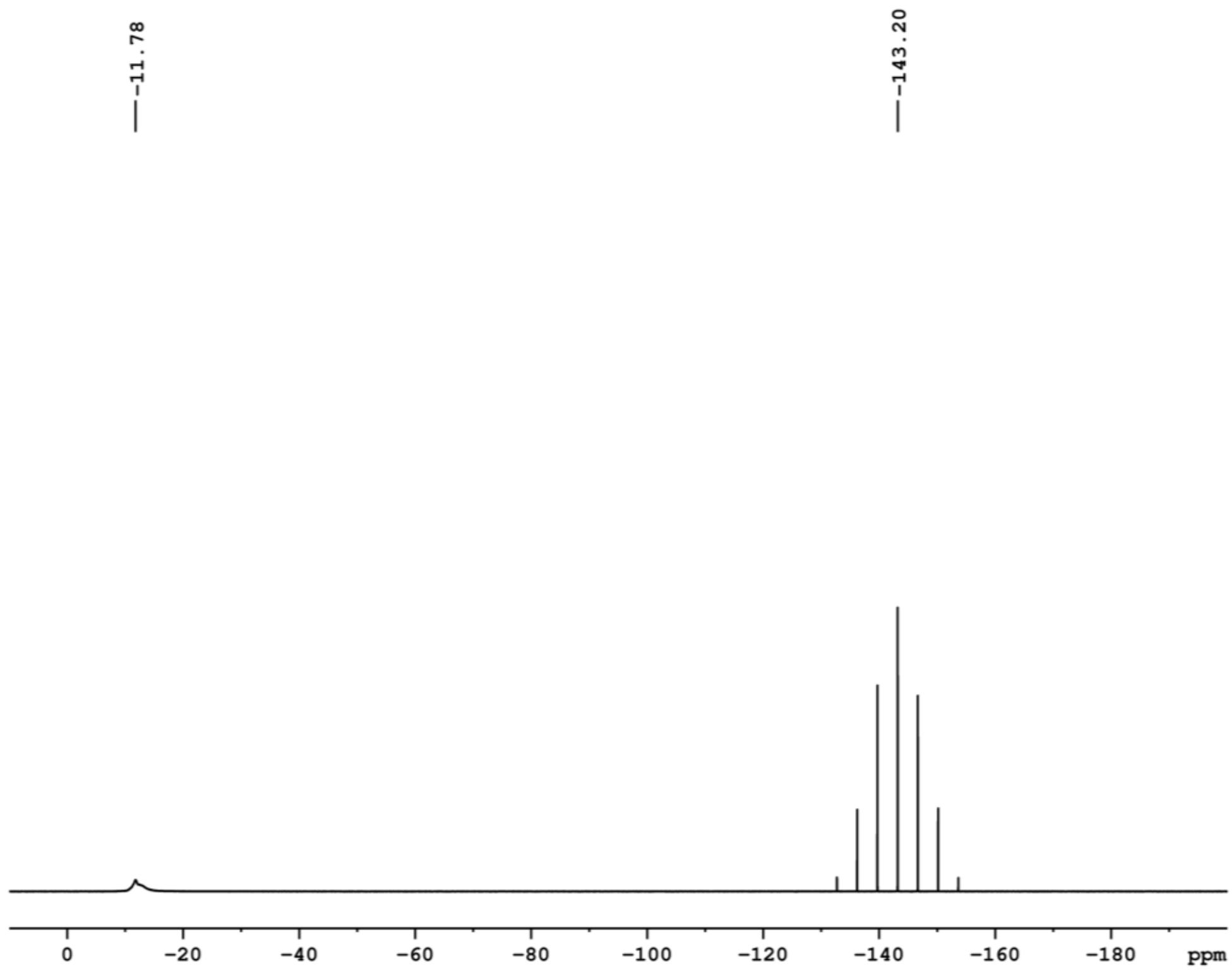

Figure S3.19. $^{31}P\{^1H\}$ NMR of **Cu24** in CD_3CN (202 MHz).

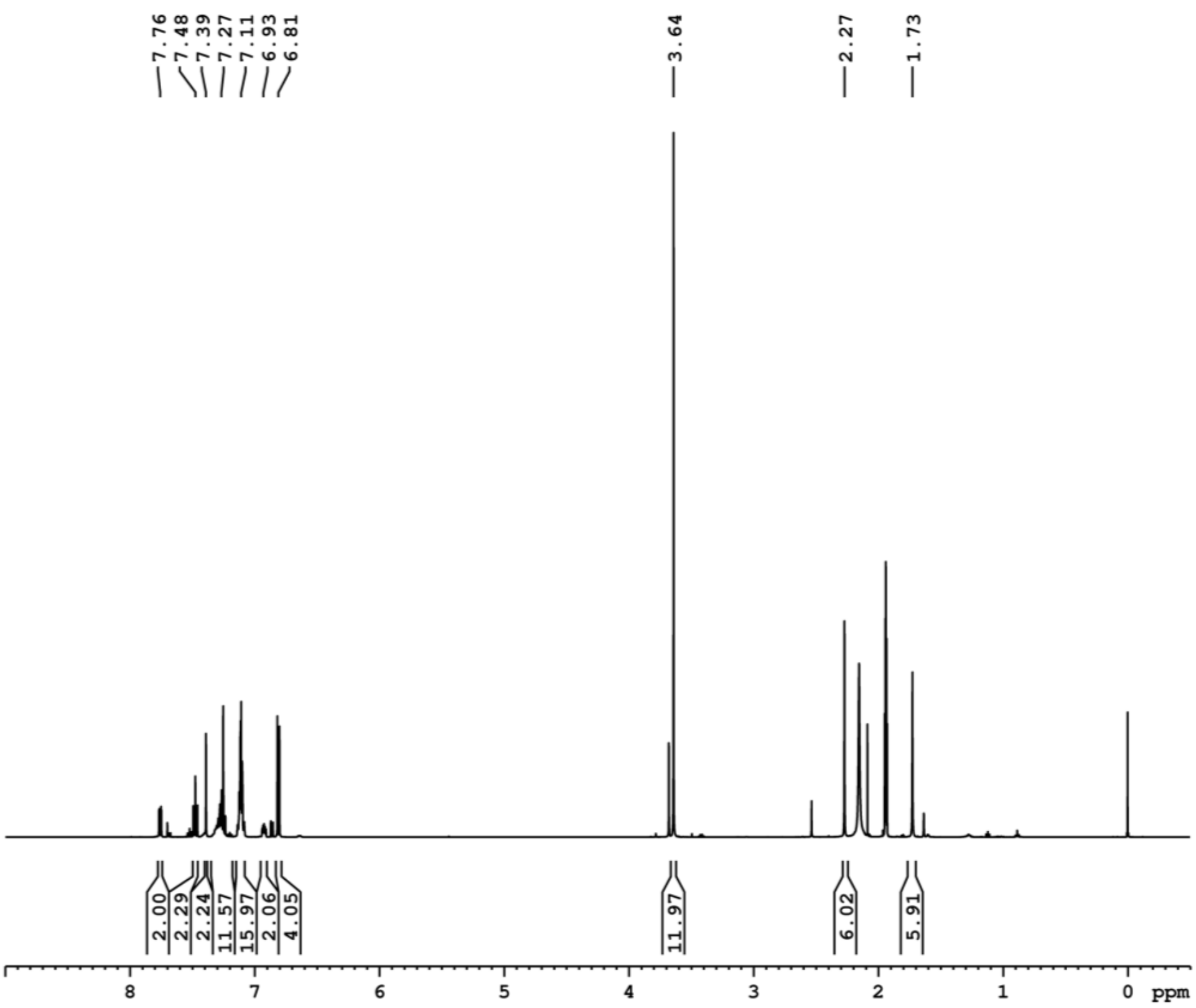

Figure S3.20. ^1H NMR of **Cu26** in CD_3CN (500 MHz).

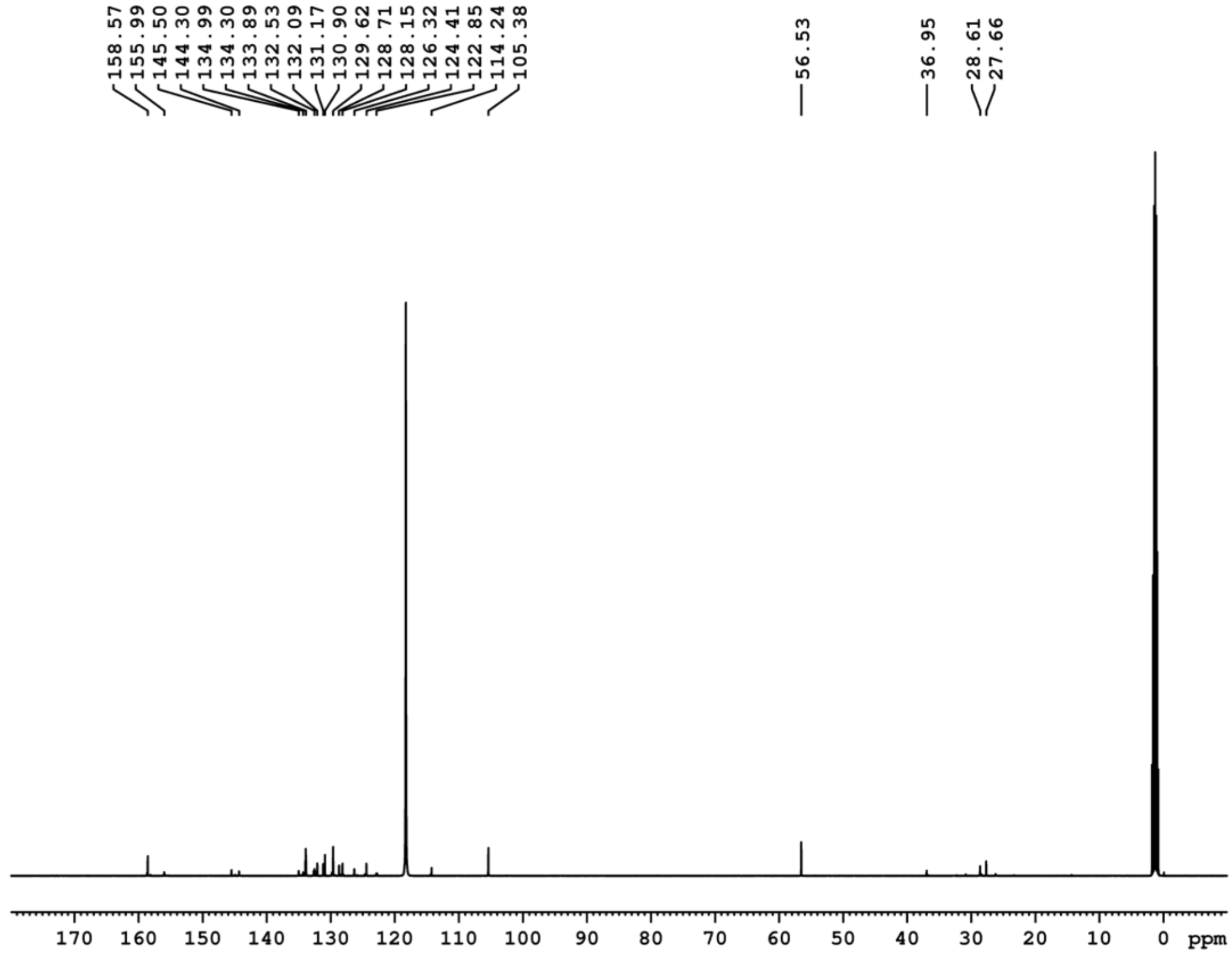

Figure S3.21. $^{13}C\{^{1}H\}$ NMR of **Cu26** in CD_3CN (125 MHz).

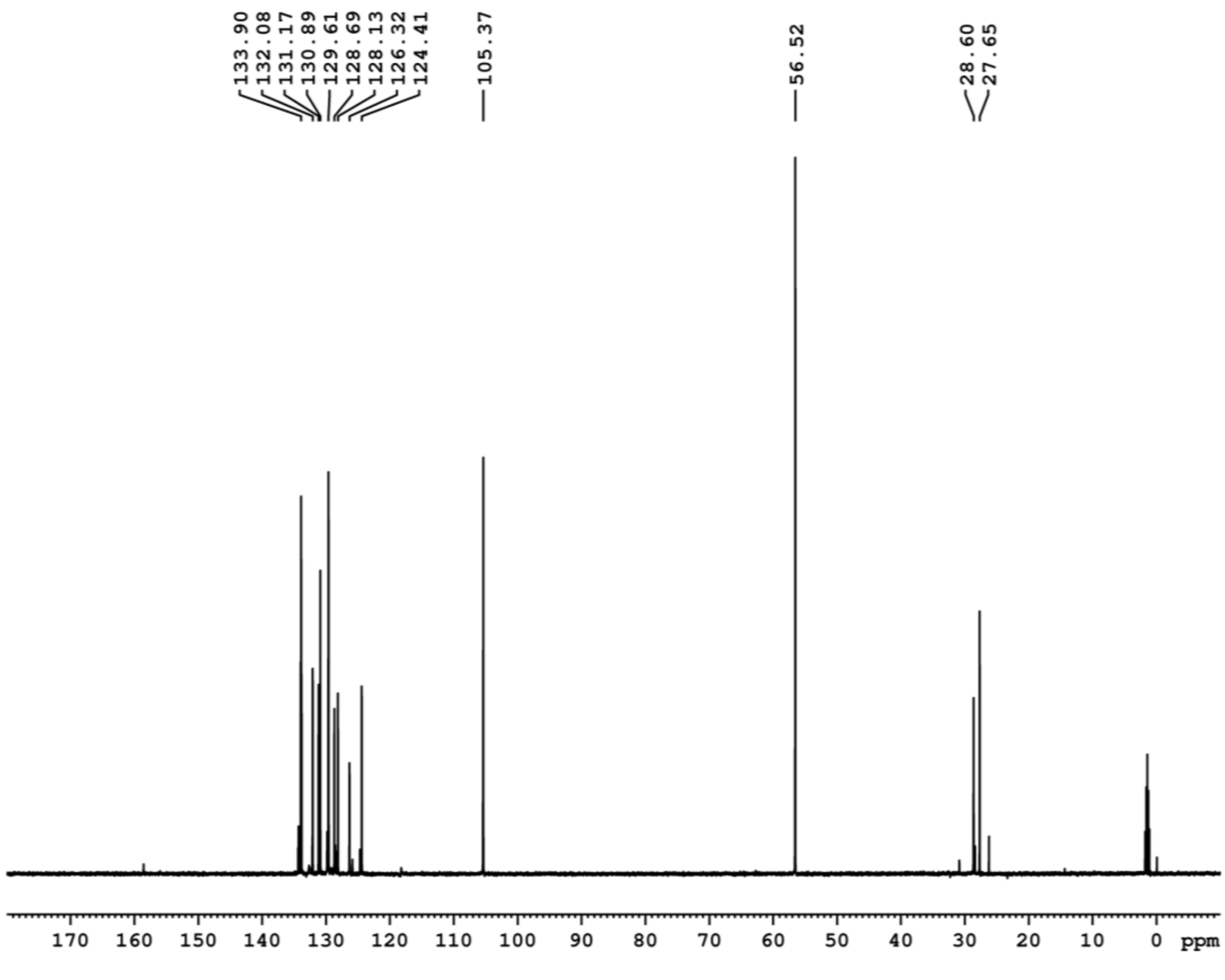

Figure S3.22. DEPT-135 NMR of **Cu26** in CD_3CN (125 MHz).

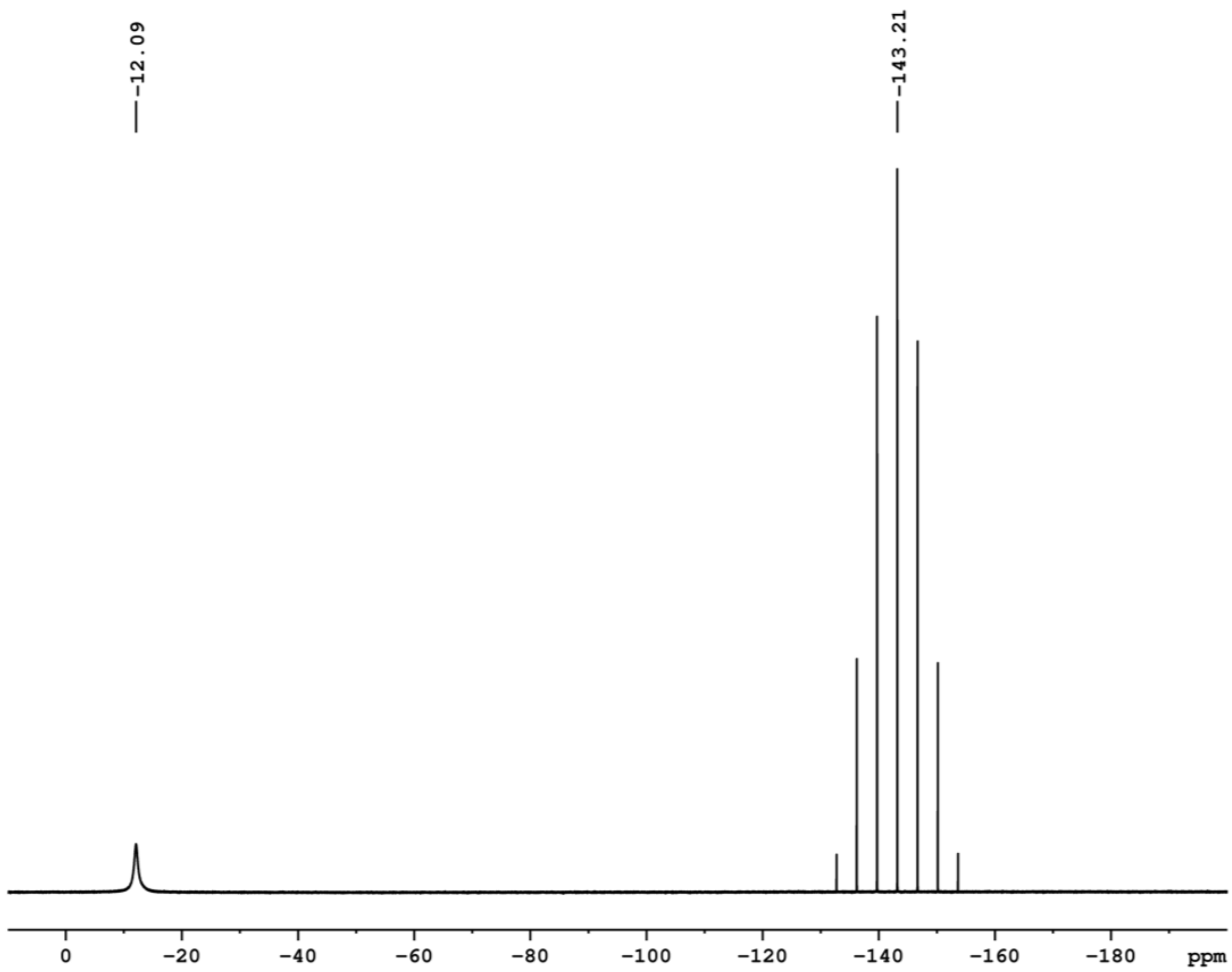

Figure S3.23. $^{31}P\{^{1}H\}$ NMR of **Cu26** in CD_3CN (202 MHz).

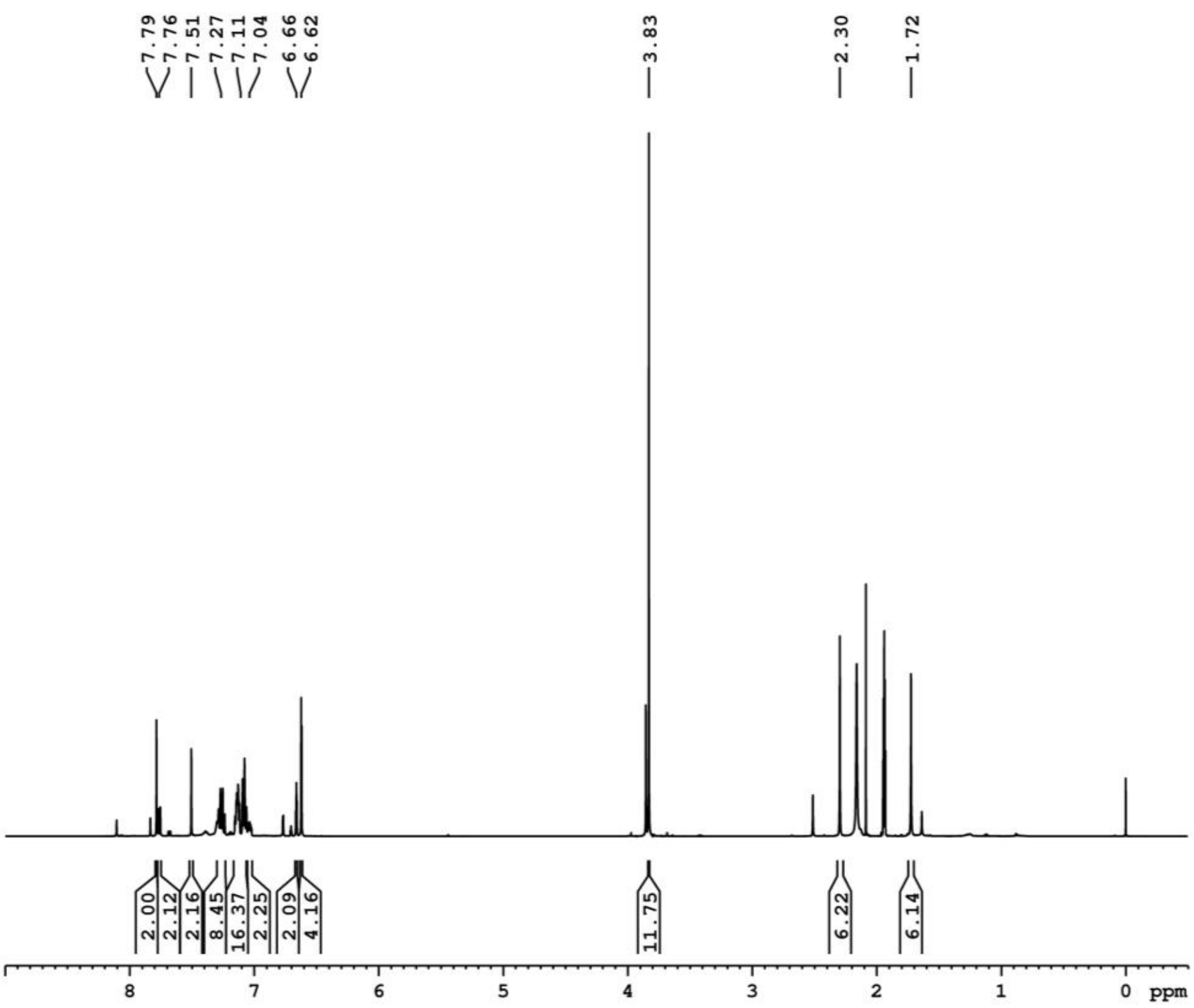

Figure S3.24. ^{1}H NMR of **Cu35** in CD_3CN (500 MHz).

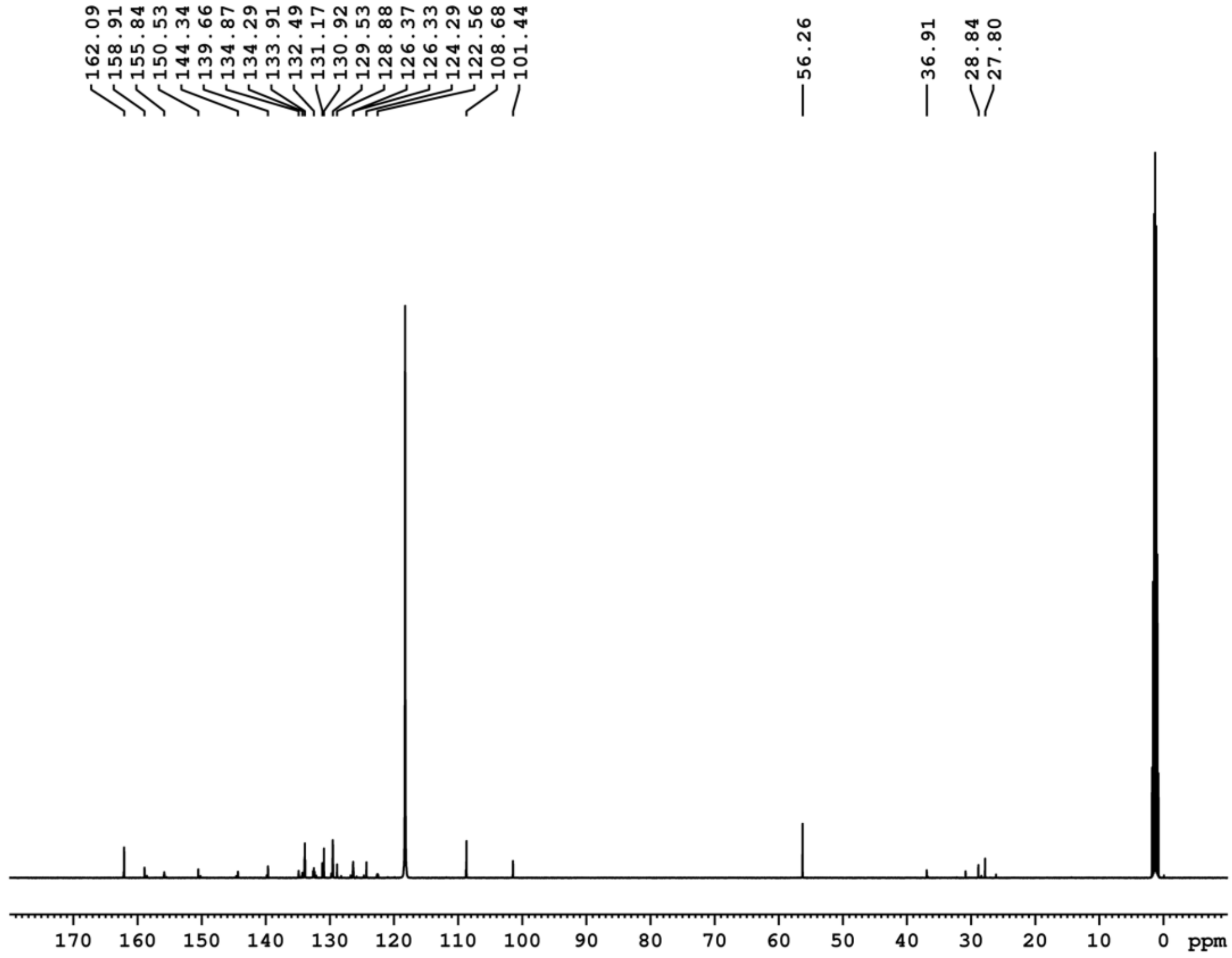

Figure S3.25. $^{13}C\{^1H\}$ NMR of **Cu35** in CD_3CN (125 MHz).

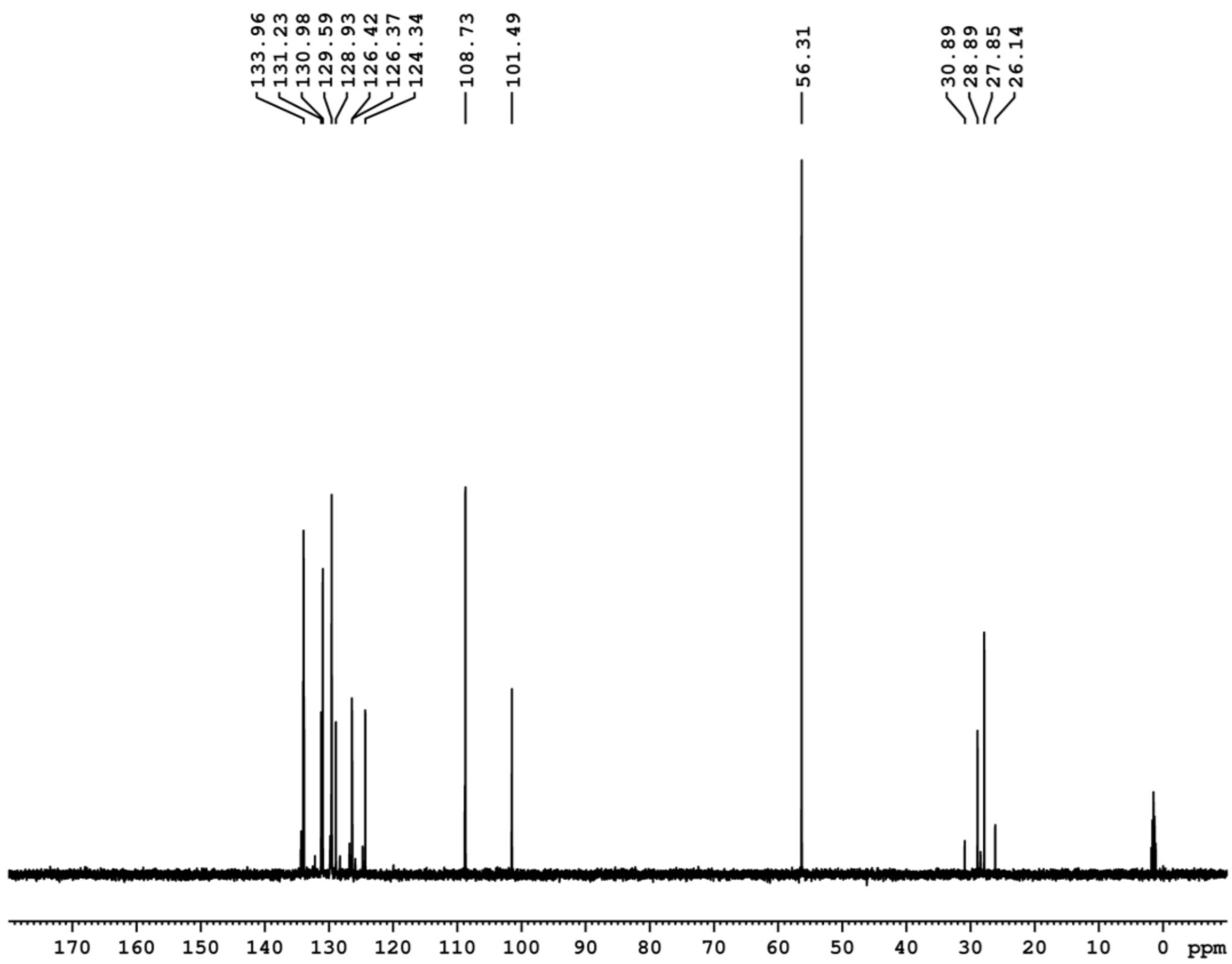

Figure S3.26. DEPT-135 NMR of **Cu35** in CD_3CN (125 MHz).

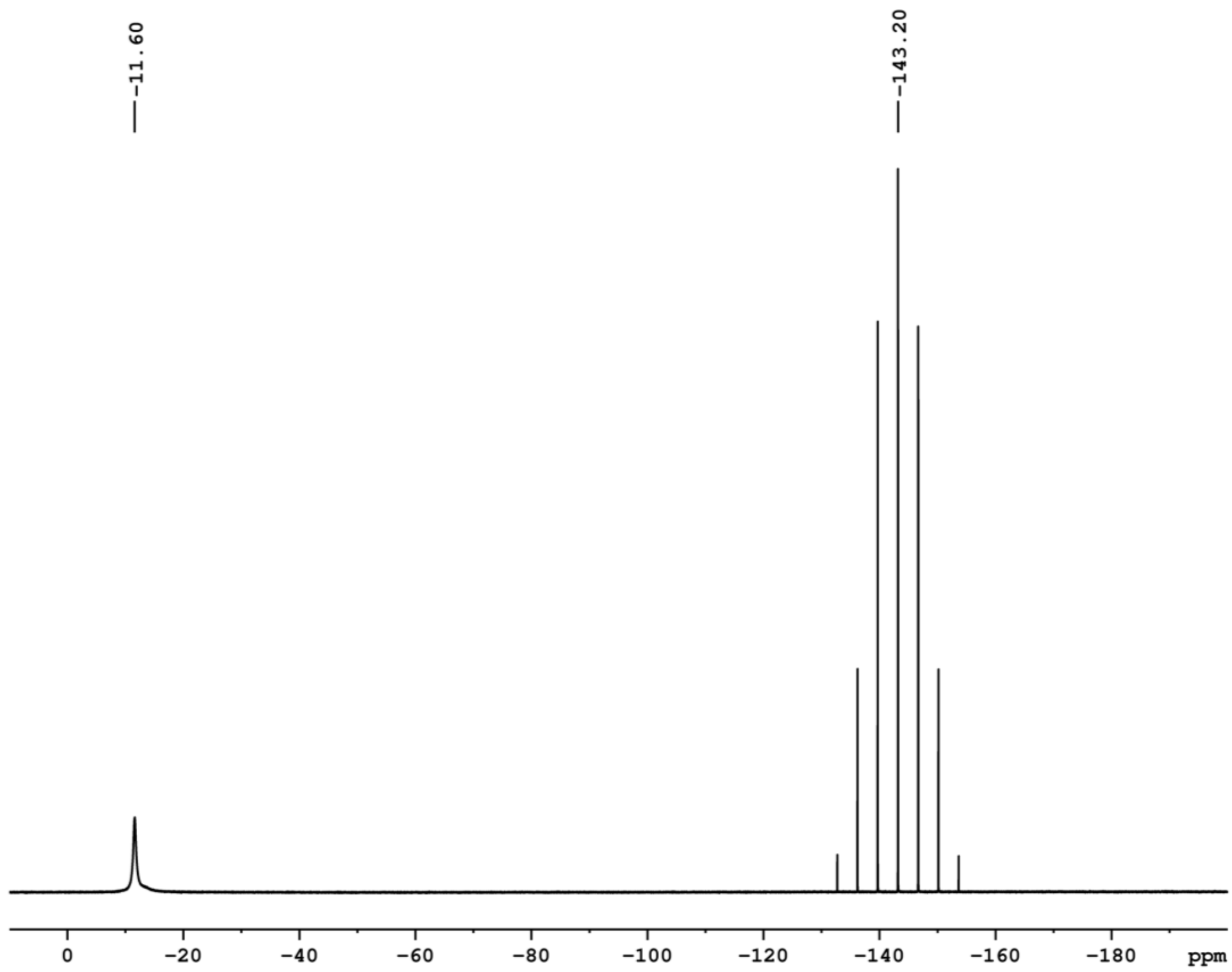

Figure S3.27. $^{31}P\{^1H\}$ NMR of **Cu35** in CD_3CN (202 MHz).

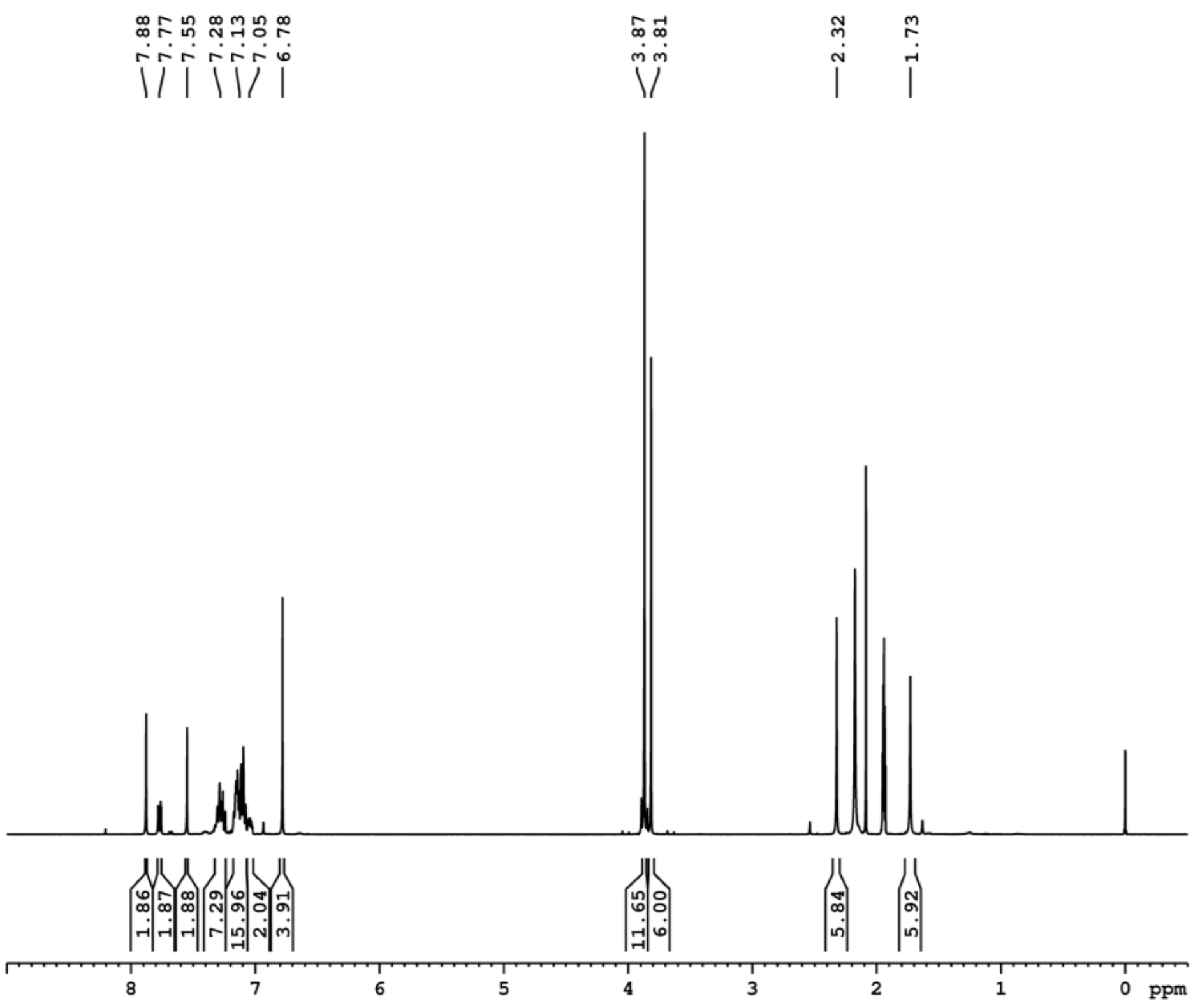

Figure S3.28. ^1H NMR of **Cu345** in CD_3CN (400 MHz).

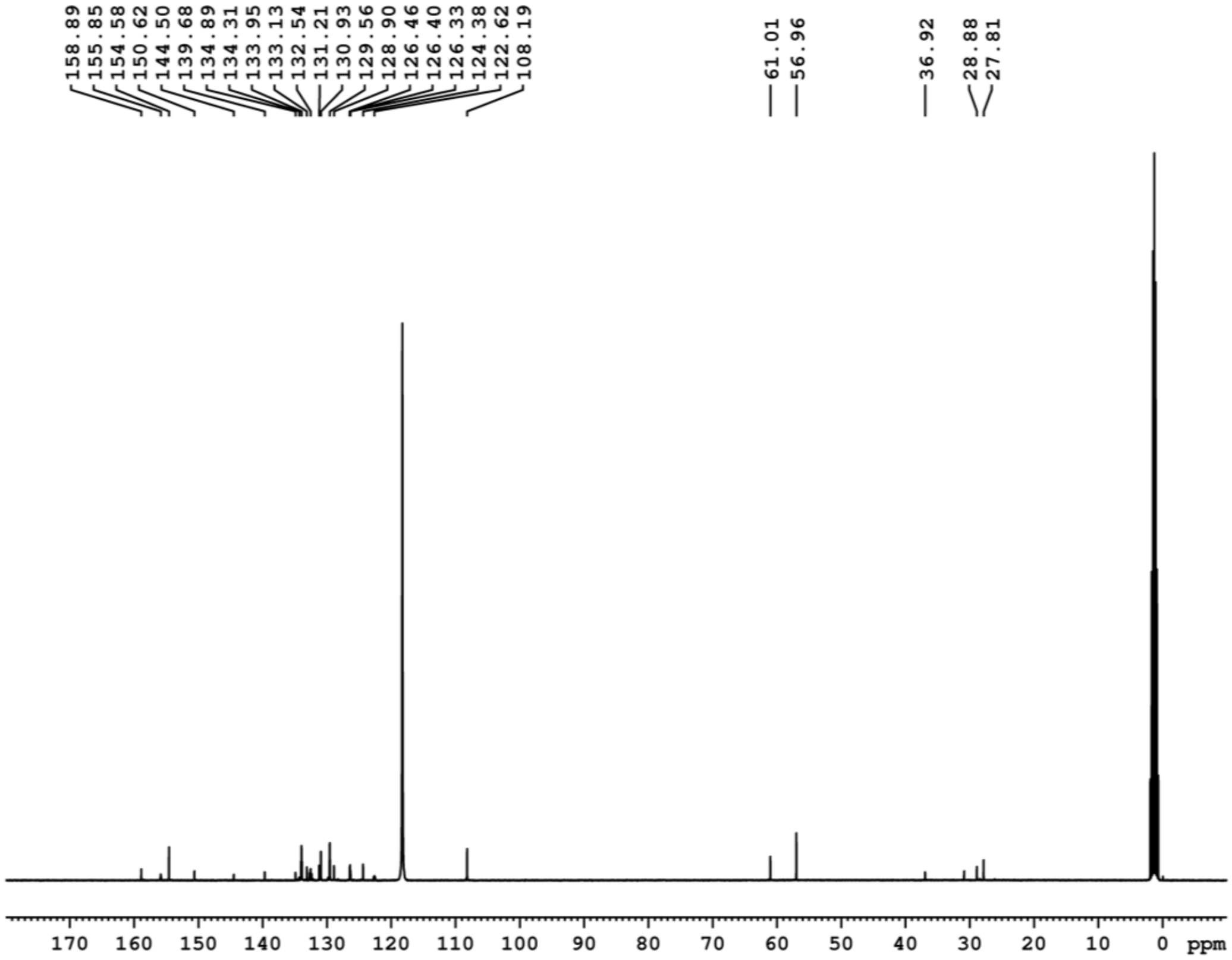

Figure S3.29. $^{13}C\{^{1}H\}$ NMR of **Cu345** in CD_3CN (100 MHz).

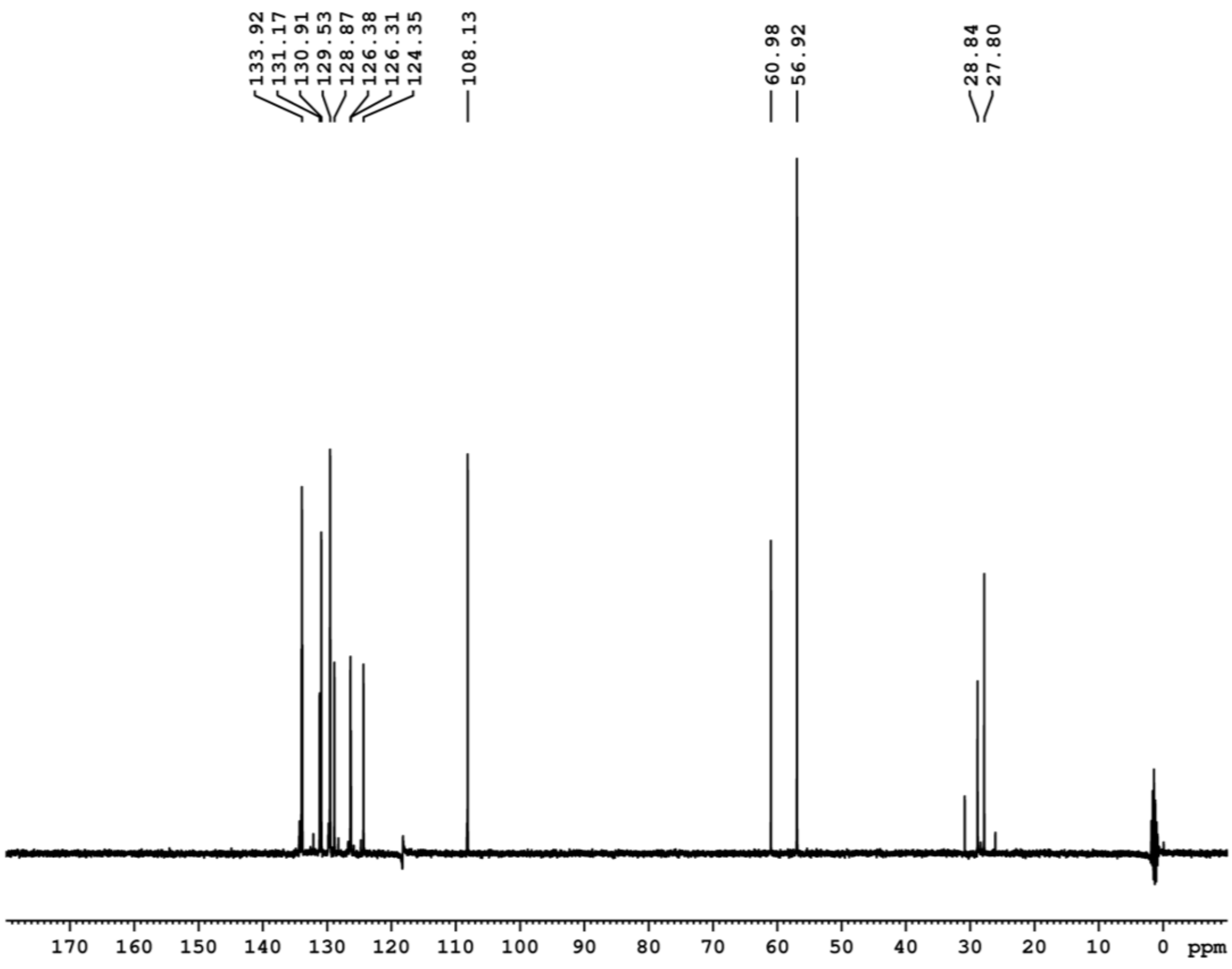

Figure S3.30. DEPT-135 NMR of **Cu345** in CD_3CN (100 MHz).

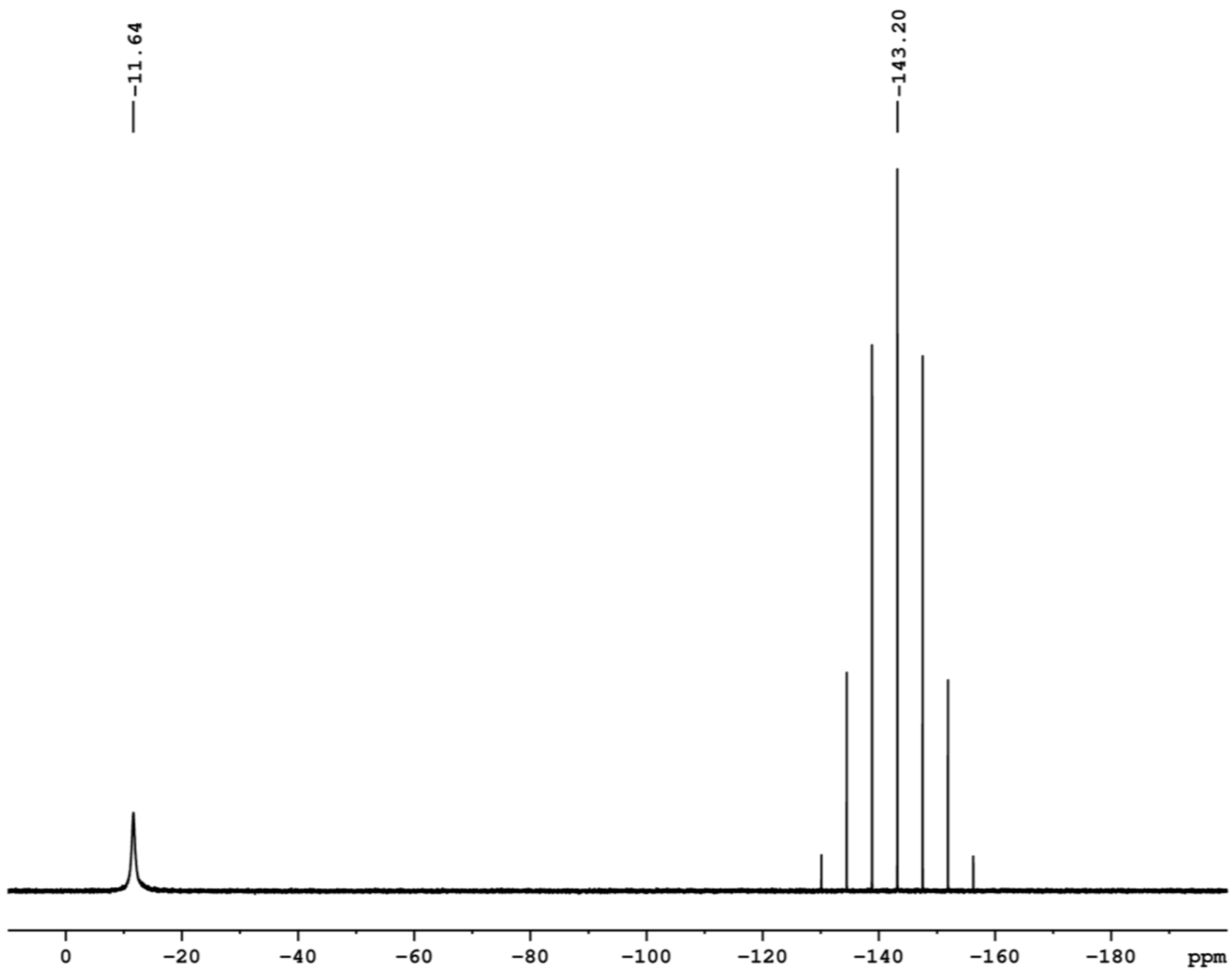

Figure S3.31. $^{31}P\{^{1}H\}$ NMR of **Cu345** in CD_3CN (162 MHz).

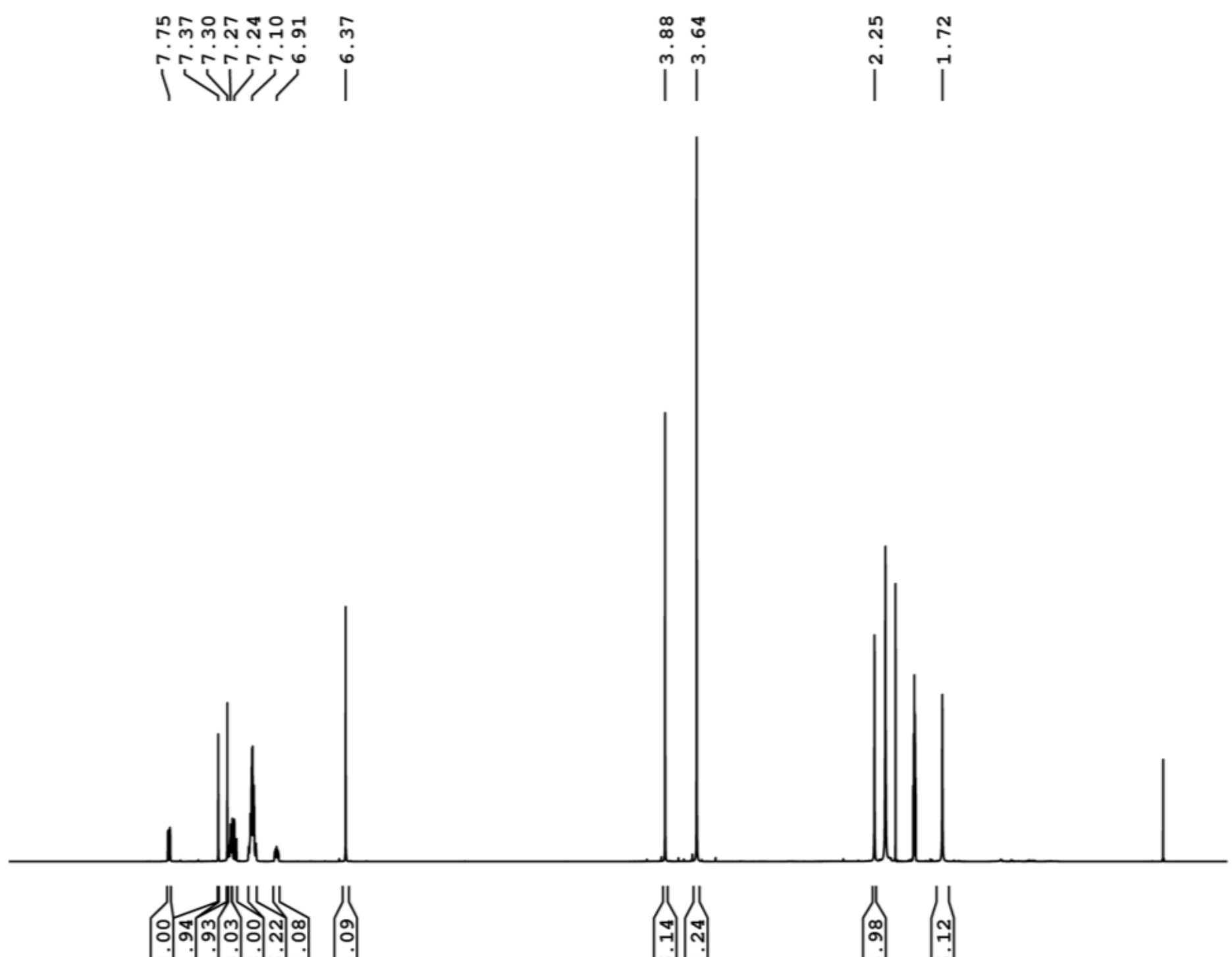

Figure S3.32. ^{1}H NMR of **Cu246** in CD_3CN (500 MHz).

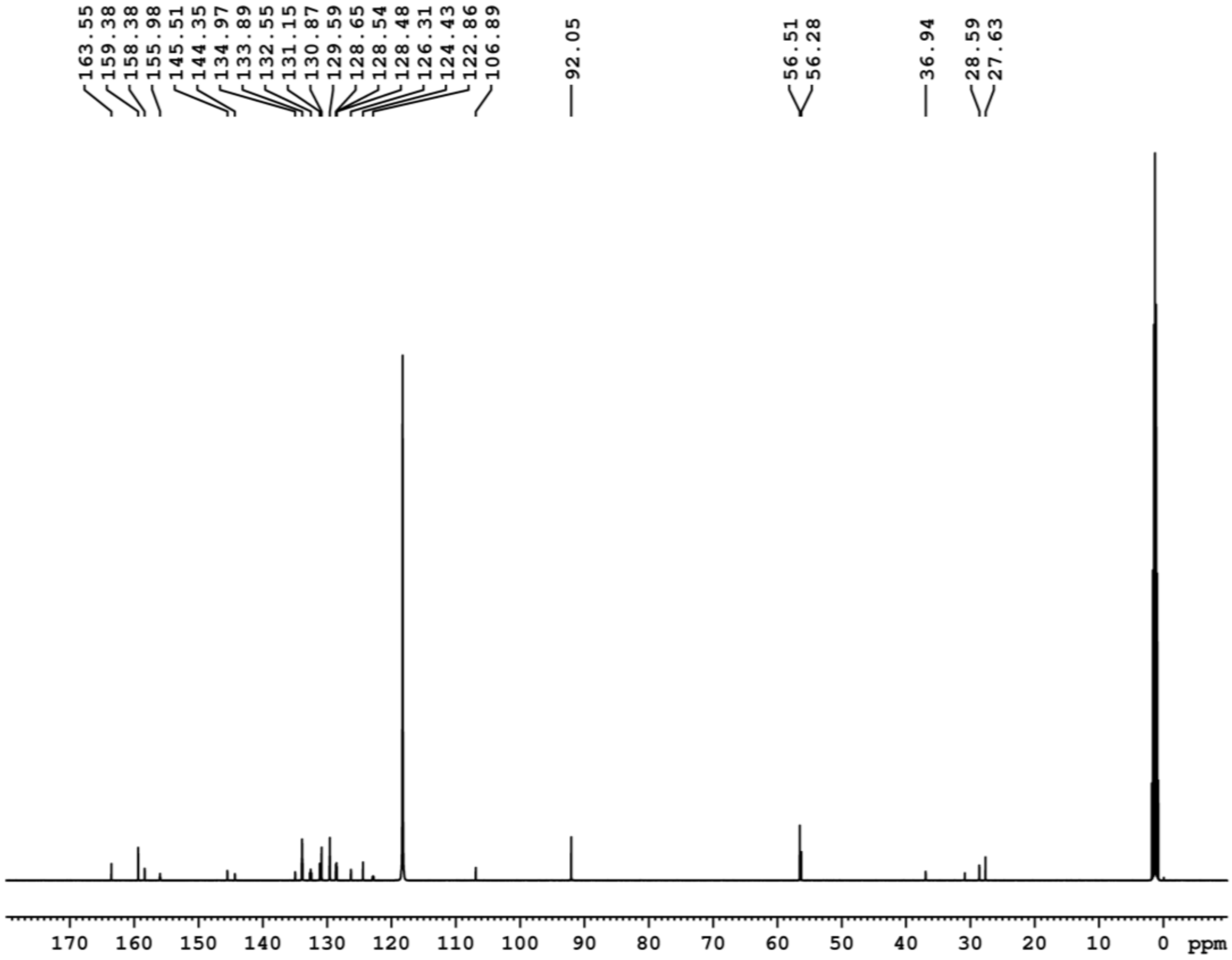

Figure S3.33. $^{13}C\{^1H\}$ NMR of **Cu246** in CD_3CN (125 MHz).

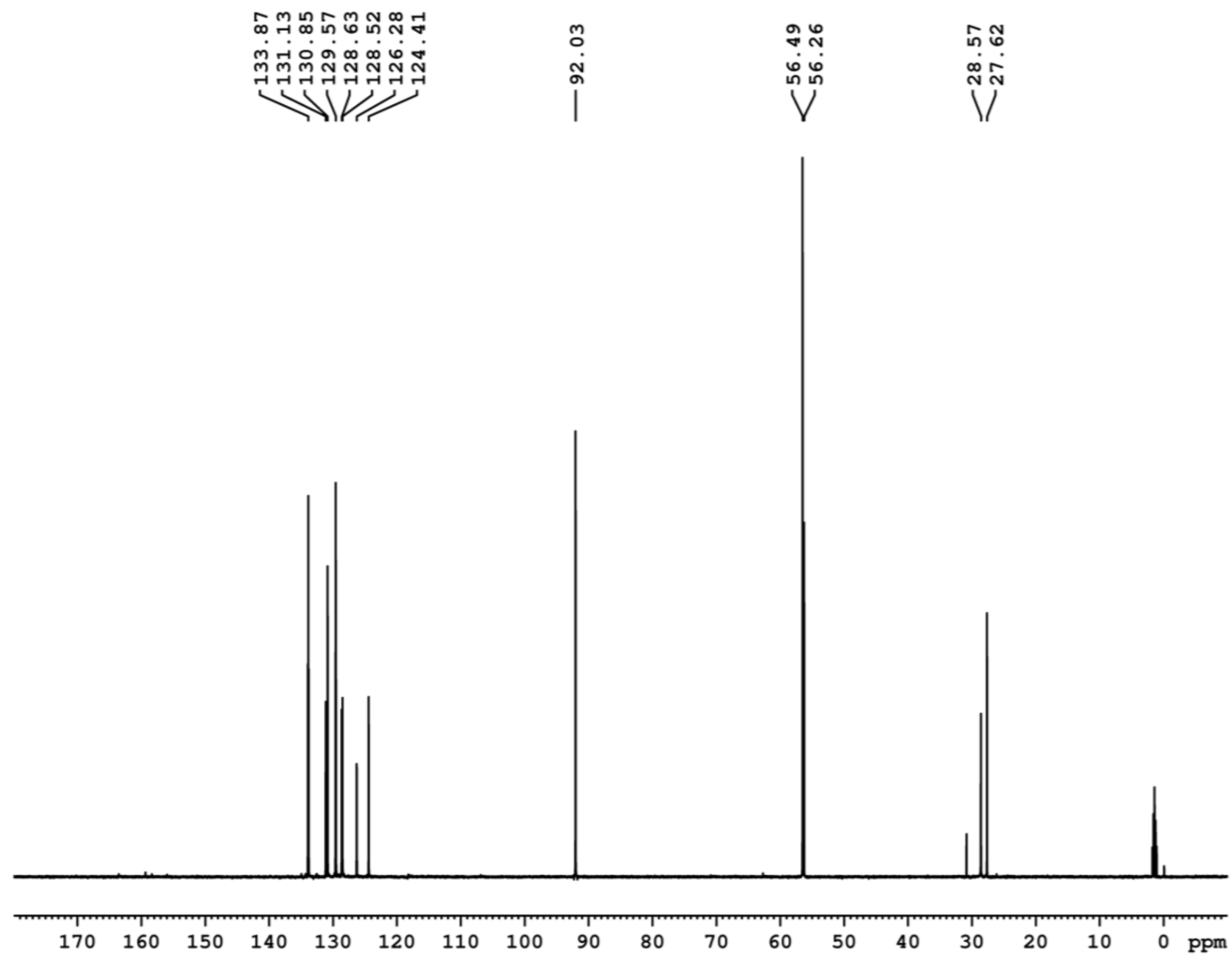

Figure S3.34. DEPT-135 NMR of **Cu246** in CD_3CN (125 MHz).

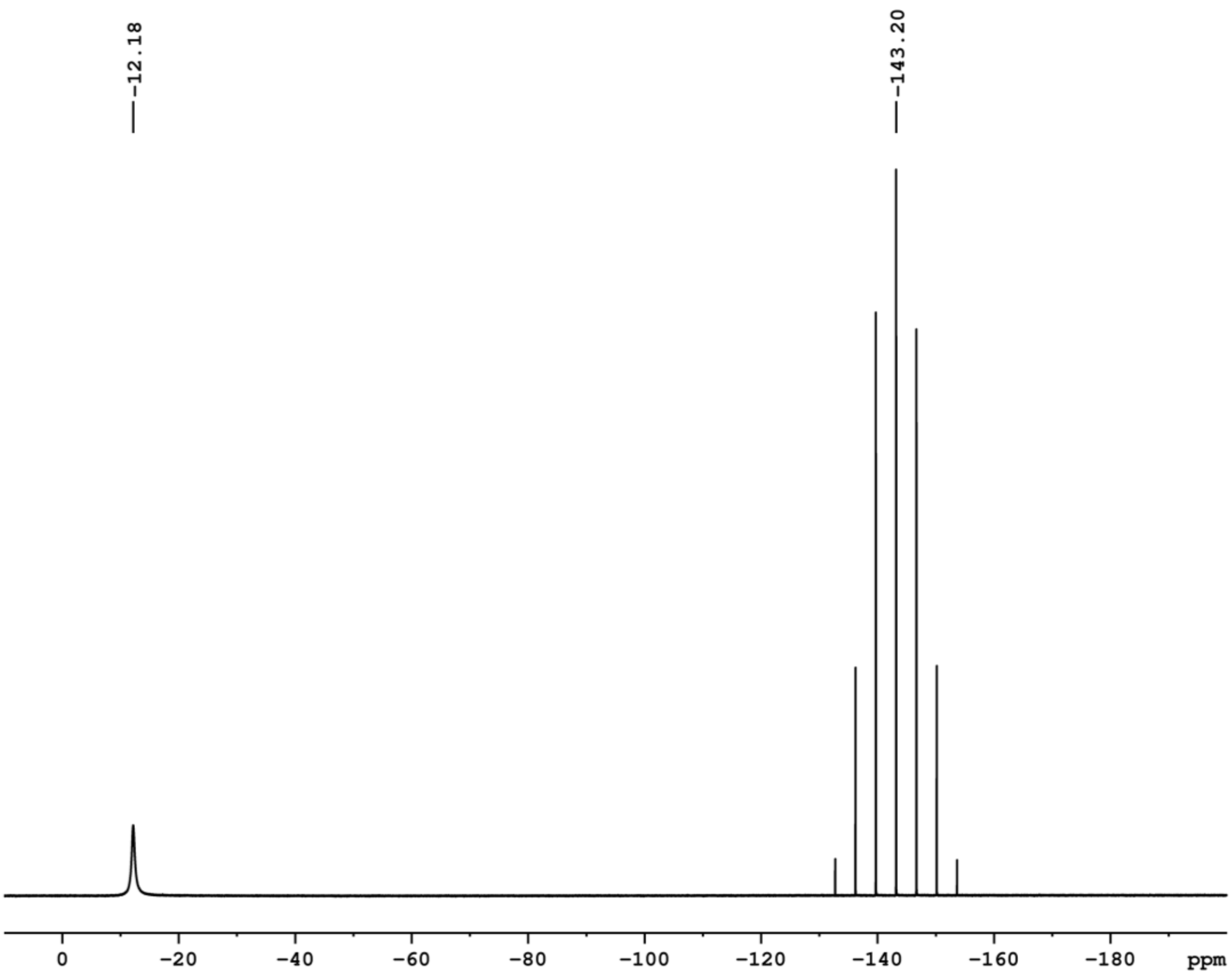

Figure S3.35. $^{31}P\{^{1}H\}$ NMR of **Cu246** in CD_3CN (202 MHz).

4 Crystallographic Data

Table S4.1. Crystallographic data of the structural determination of **Cu345**.

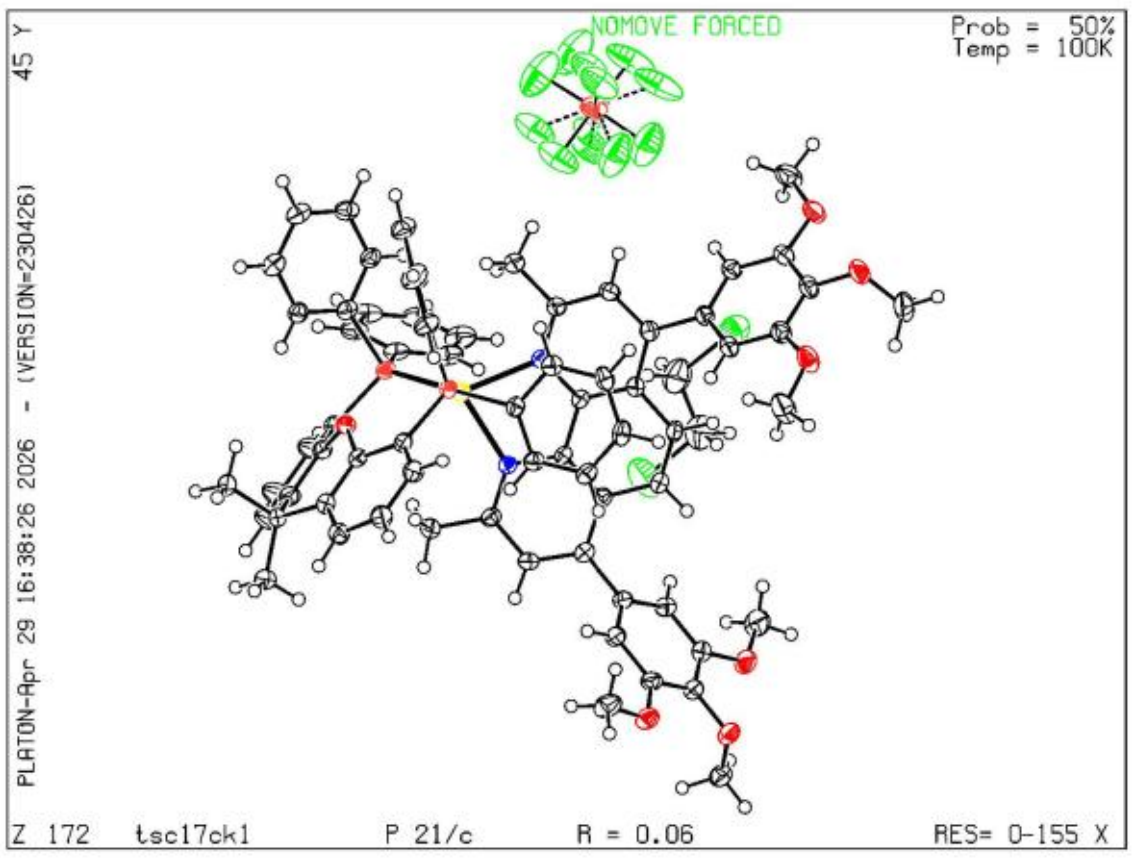

Sum formula	$C_{73}H_{68}Cl_2CuF_6N_2O_7P_3$
Moiety formula	$[C_{71}H_{64}CuN_2O_7P_2][PF_6]$, $(C_2H_4Cl_2)$
Formula mass	1426.64 g mol^{-1}
Density (calculated)	1.395 g cm^{-3}
Crystal shape, color	fragment (clear light yellow)
Crystal size	0.276 × 0.141 × 0.040 mm^3
Temperature, Radiation	100(2) K, 1.54184 Å
Abs. coefficient	2.454 mm^{-1}
F(000)	2952
Crystal system	monoclinic
Space group type (no.)	$P2_1/c$ (no. 14)
Z	4
a, b, c	14.6799(4) Å, 26.0429(6) Å, 18.5667(4) Å
α, β, γ	90°, 106.913(2)°, 90°
Volume	6791.2(3) $Å^3$
Reflections collected	121797
indep.	14051
observed [$I>2\sigma(I)$]	12000
Data collection ranges	$-18 \le h \le 18$, $-33 \le k \le 33$, $-23 \le l \le 18$
Completeness to $\theta = 77.0°$	99.2%
Data / restr. / param.	14501 / 12 / 879
R_{int}	0.0696
R_1 [$I > 2\sigma(I)$]	0.0632
wR_2 (all data)	0.1502
GoF on F^2	1.047
Largest peak/hole	0.840 and –0.736e $Å^{-3}$
CCDC Number	CCDC 2550575
Crystallization Details	From a solution in $C_2H_4Cl_2$/*n*-hexane at rt

Measurement and Refinement Details: A disordered PF_6^- moiety was refined using a split atom model (0.792(4) SOF of main component), applying geometrical restraints on the P–F distances (DFIX).

Table S4.2. Crystallographic data of the structural determination of **Cu35**.

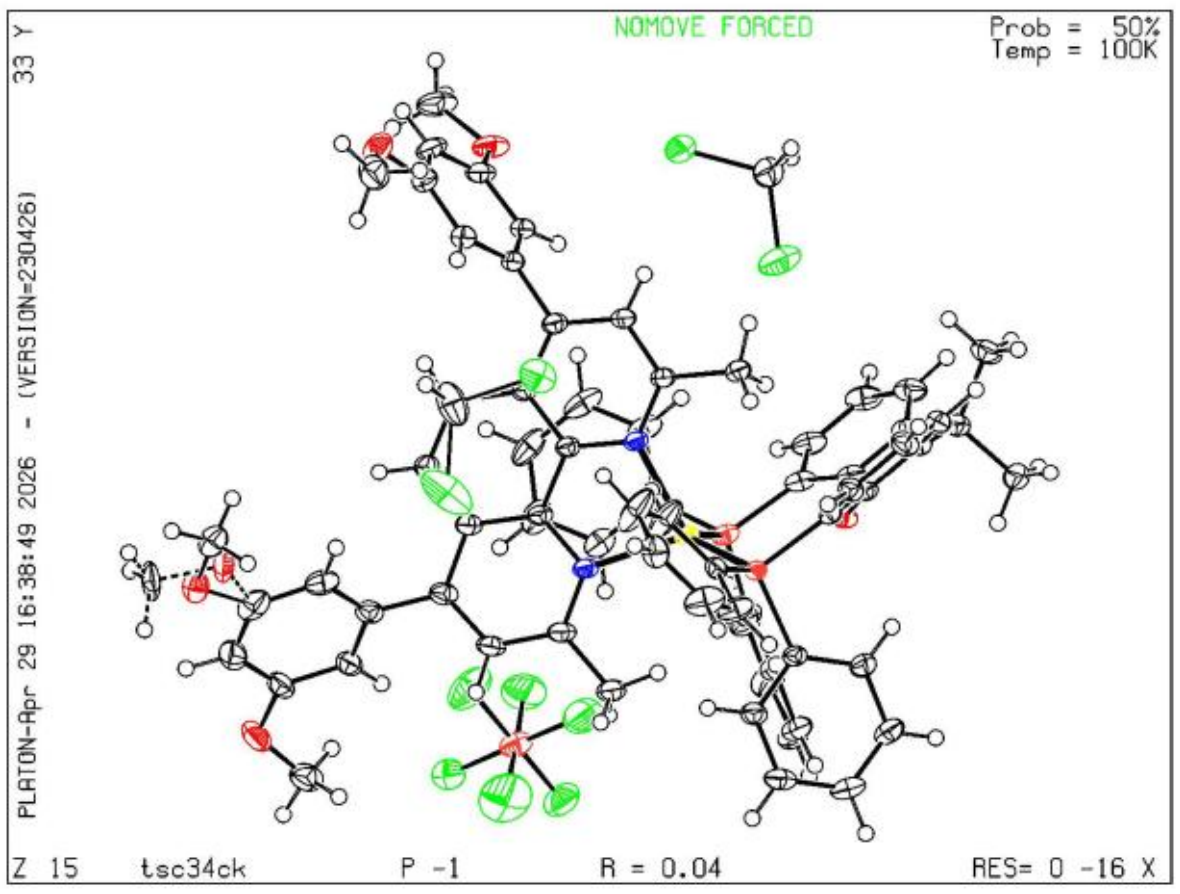

Sum formula	$C_{70.5}H_{63}Cl_3CuF_6N_2O_5P_3$
Moiety formula	$[C_{69}H_{60}CuN_2O_5P_2][PF_6]$, 2 ($CH_2Cl_2$), 0.5 ($CH_2Cl_2$)
Formula mass	1395.03 g mol^{-1}
Density (calculated)	1.465 g cm^{-3}
Crystal shape, color	lath-like fragment (clear intense yellow)
Crystal size	0.175 × 0.066 × 0.034 mm^3
Temperature, Radiation	100(2) K, 1.54184 Å
Abs. coefficient	2.9575 mm^{-1}
F(000)	1438
Crystal system	triclinic
Space group type (no.)	*P* -1 (no. 2)
Z	2
a, b, c	12.5306(4) Å, 14.1187(5) Å, 19.7150(6) Å
α, β, γ	77.388(3)°, 86.360(3)°, 68.368(3)°
Volume	3163.46(19) Å^3
Reflections collected	170132
indep.	13646
observed [$I>2\sigma(I)$]	12314
Data collection ranges	$-16 \le h \le 15$, $-17 \le k \le 17$, $-23 \le l \le 25$
Completeness to $\theta = 67.7°$	100%
Data / restr. / param.	13646 / 0 / 857
R_{int}	0.0552
R_1 [$I > 2\sigma(I)$]	0.0416
wR_2 (all data)	0.1087
GoF on F^2	1.040
Largest peak/hole	0.997 and –0.937e Å^{-3}
CCDC Number	CCDC 2550577
Crystallization Details	From a solution in CH_2Cl_2 layered with *n*-hexane at 6 °C

Measurement and Refinement Details: A disordered methoxy group was refined using a split atom model (0.583(8) SOF of main component).

Table S4.3. Crystallographic data of the structural determination of **L246**.

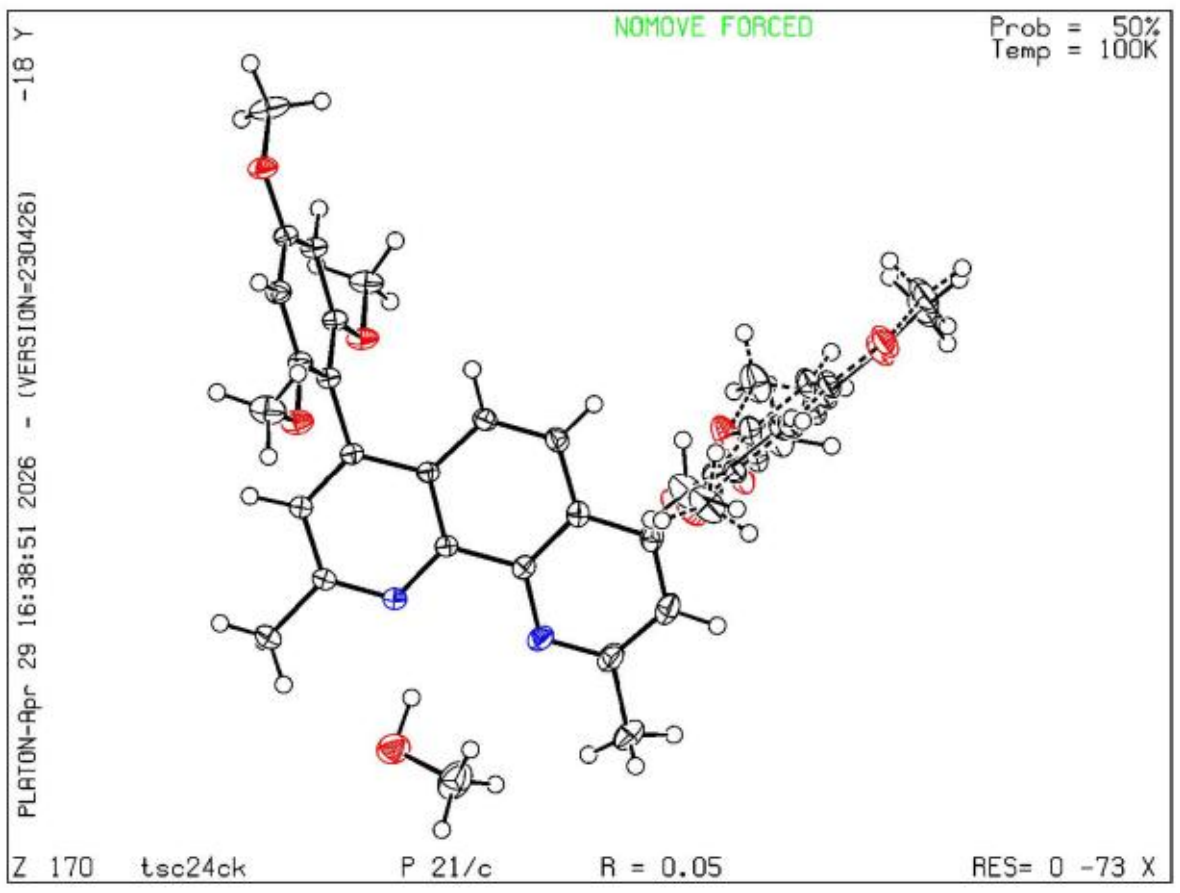

Sum formula	$C_{34}H_{40}N_2O_8$
Moiety formula	$C_{32}H_{32}N_2O_6$, 2 (CH_3OH)
Formula mass	604.68 g mol^{-1}
Density (calculated)	1.247 g cm^{-3}
Crystal shape, color	fragment of prism (clear orange)
Crystal size	0.677 × 0.525 × 0.445 mm^3
Temperature, Radiation	100(2) K, 0.71073 Å
Abs. coefficient	0.089 mm^{-1}
F(000)	1288
Crystal system	monoclinic
Space group type (no.)	$P2_1/c$
Z	4
a, b, c	13.8503(4) Å, 14.0573(3) Å, 16.5554(4) Å
α, β, γ	90°, 92.134(2)°, 90°
Volume	3221.06(14) Å^3
Reflections collected	186976
indep.	170079
Data collection ranges	$-23 \le h \le 23$, $-24 \le k \le 24$, $-27 \le l \le 28$
Completeness to $\theta = 25.242°$	99.9%
Data / restr. / param.	17079 / 28 / 426
R_{int}	0.0382
R_1 [$I > 2\sigma(I)$]	0.0533
wR_2 (all data)	0.1684
GoF on F^2	1.056
Largest peak/hole	1.195 and –0.585e Å^{-3}
CCDC Number	CCDC 2550576
Crystallization Details	From a solution in MeOH at 4 °C

Measurement and Refinement Details: A [$C_6H_2(OCH_3)_3$] moiety was refined using a split atom model (0.752(1) SOF of main component), applying geometrical restraints (SAME, SADI). A common ADP was refined for each disordered atom pair. The relatively high residual electron density may be caused by unresolved disorder of a molecule of co-crystallized MeOH. No appropriate model could be established for additional co-crystallized solvent molecules, presumably MeOH; the data were processed using the BYPASS algorithm as implemented in OLEX2.

5 (Time-Dependent) Density Functional Theory (TD-DFT)

Table S5.1. Excitation energies (Δ*E* in eV), wavelengths (*λ* in nm) and oscillator strengths (*f*) of the first ten singlet-singlet vertical excitations of **C24**. The transitions are visualized in as natural transition orbitals (NTOs) of the initial and final state with the highest contribution (cont. in %) of each transition is displayed.

state	*λ* / nm	Δ*E* / eV	*f*	cont. / %	initial state	final state	character
S_1	425	2.914	0.1328	99	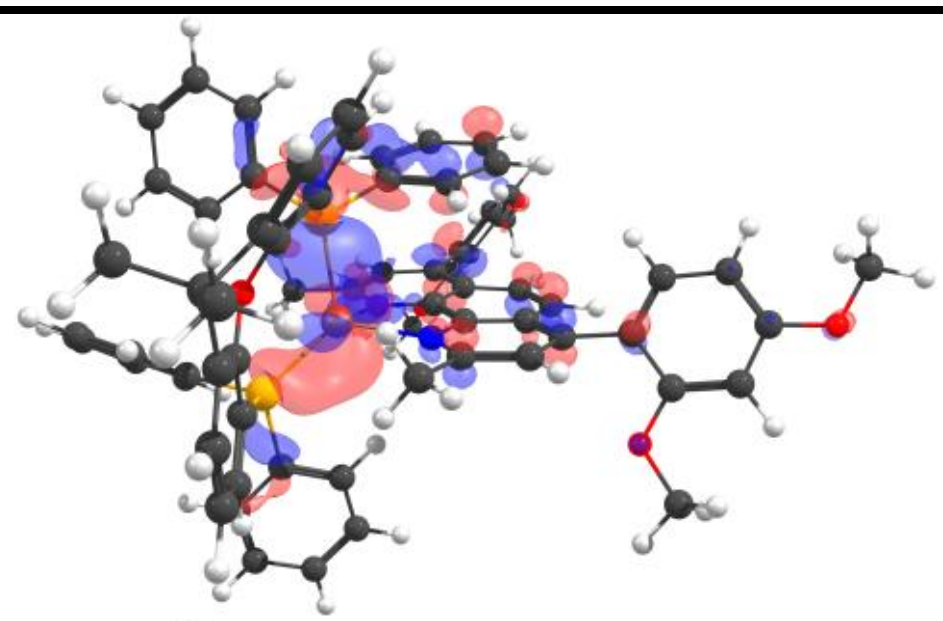	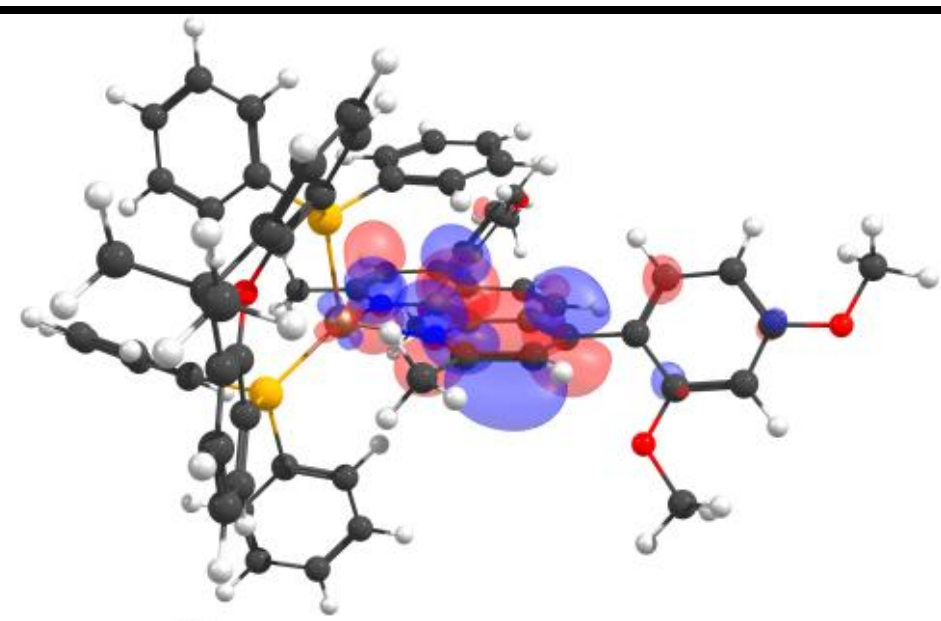	$ML_{phen}CT$
S_2	393	3.157	0.1159	88	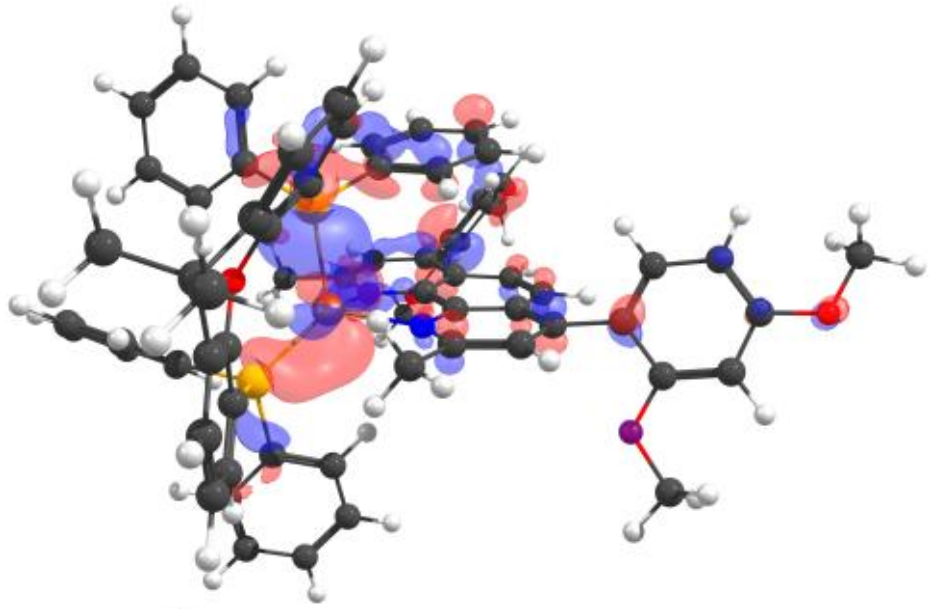	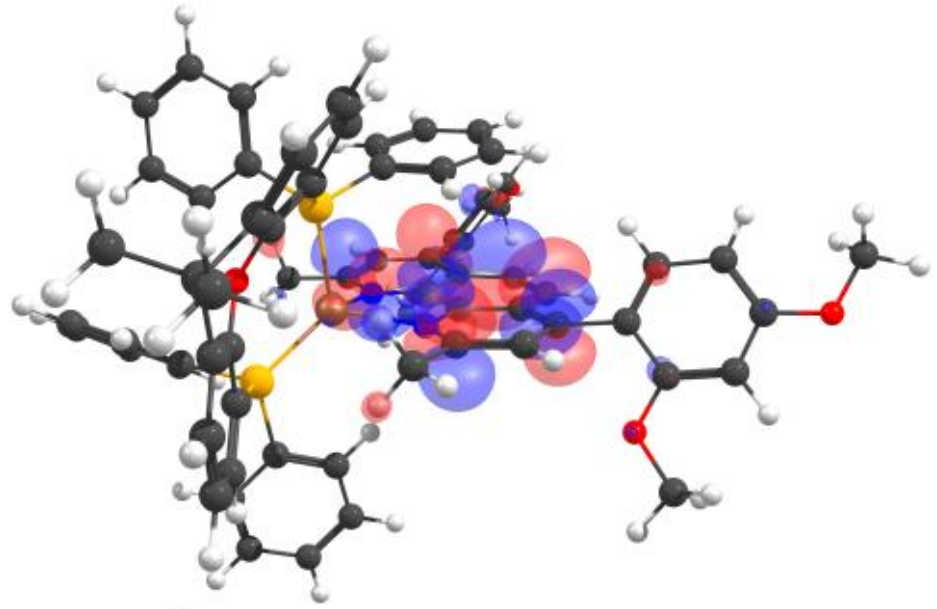	$ML_{phen}CT$
S_3	384	3.229	0.0252	93	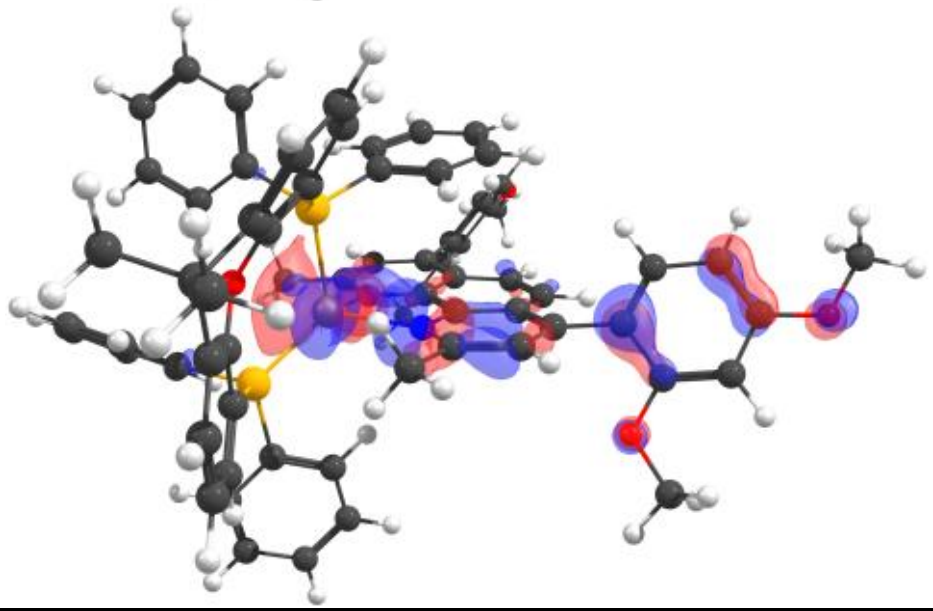	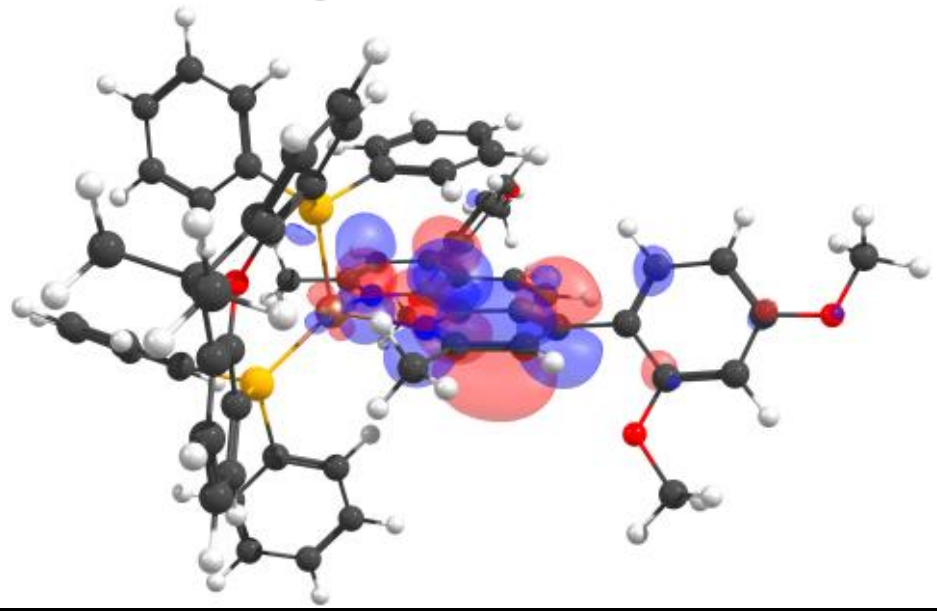	$ML_{phen}CT$

state	λ / nm	ΔE / eV	f	cont. / %	initial state	final state	character
S_4	376	3.295	0.0157	95	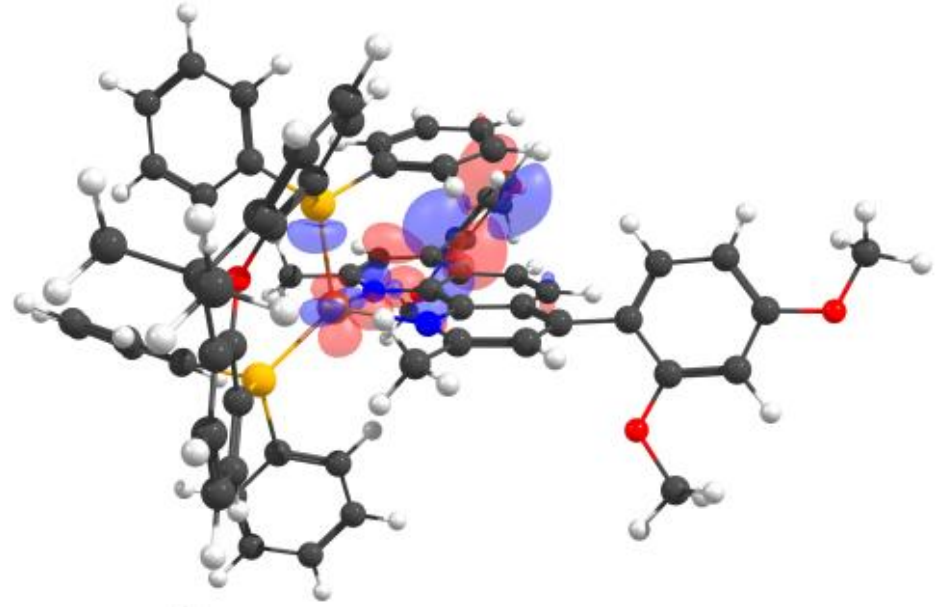	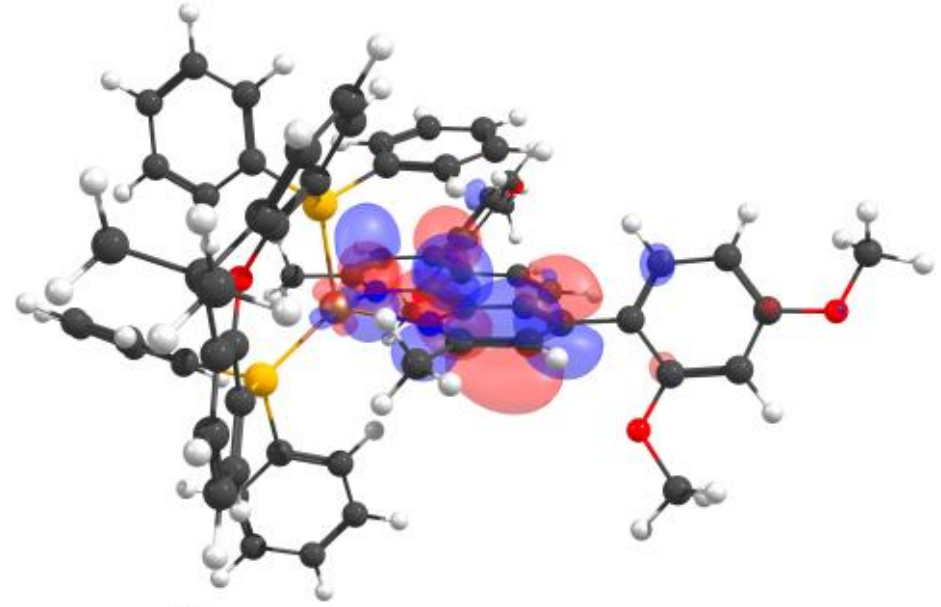	$IL_{py\rightarrow phen}CT$
S_5	369	3.360	0.0720	95	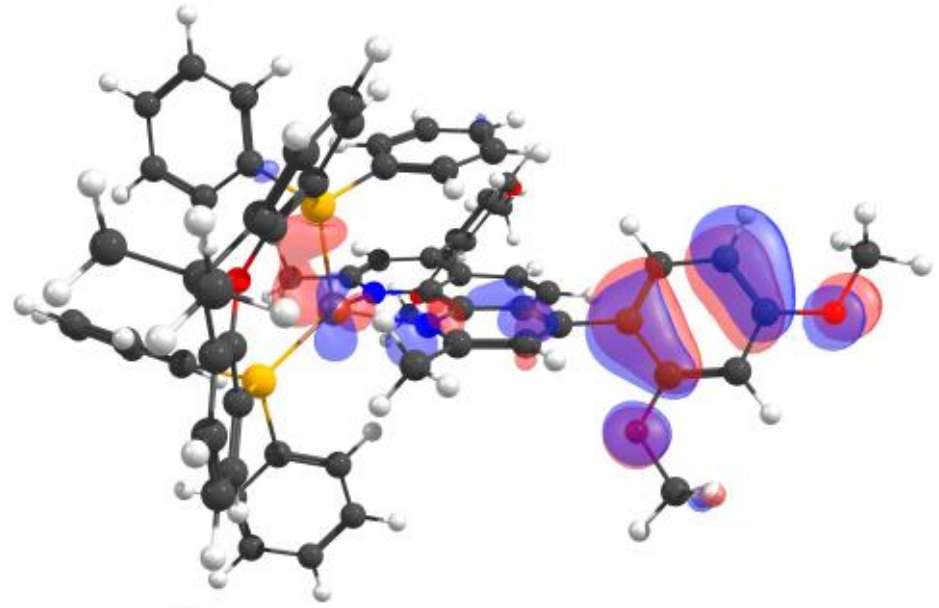	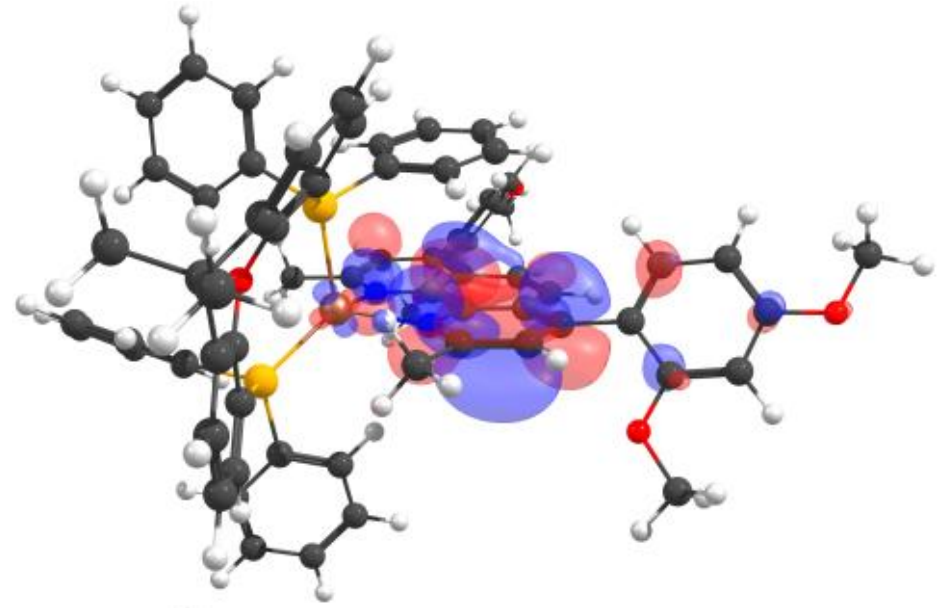	$IL_{py\rightarrow phen}CT$
S_6	358	3.462	0.0876	95	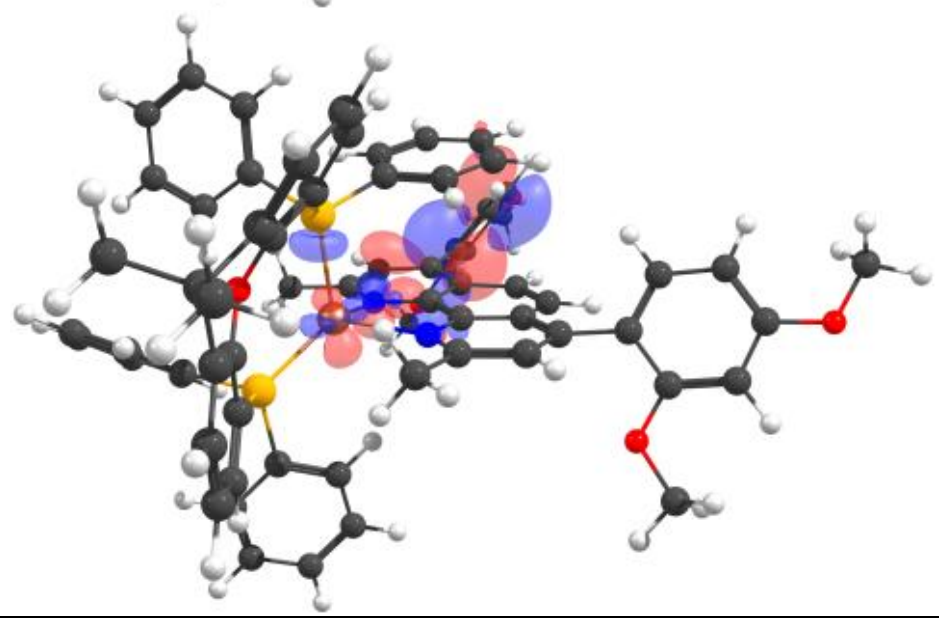	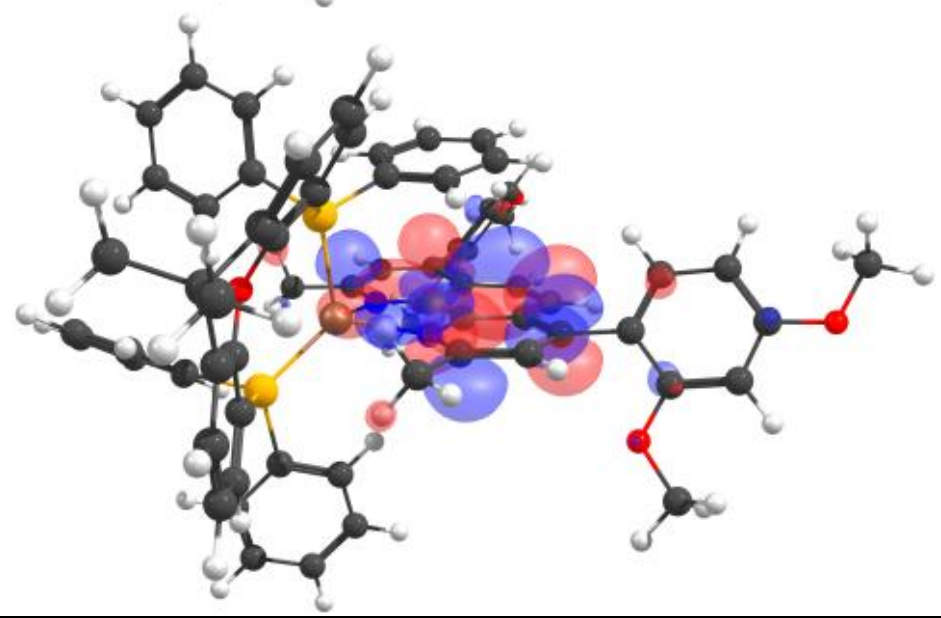	$IL_{py\rightarrow phen}CT$

state	λ / nm	ΔE / eV	f	cont. / %	initial state	final state	character
S_7	358	3.465	0.0031	91	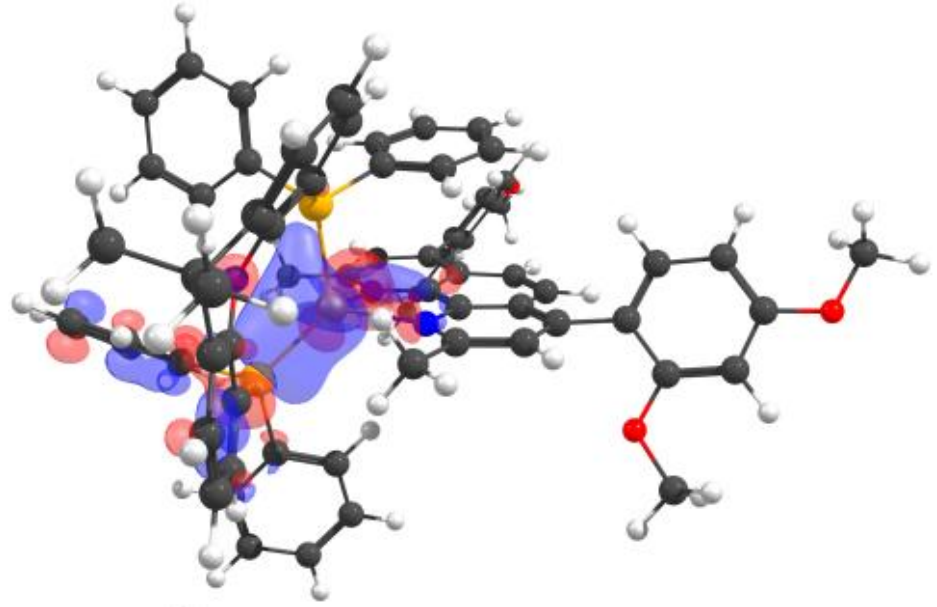	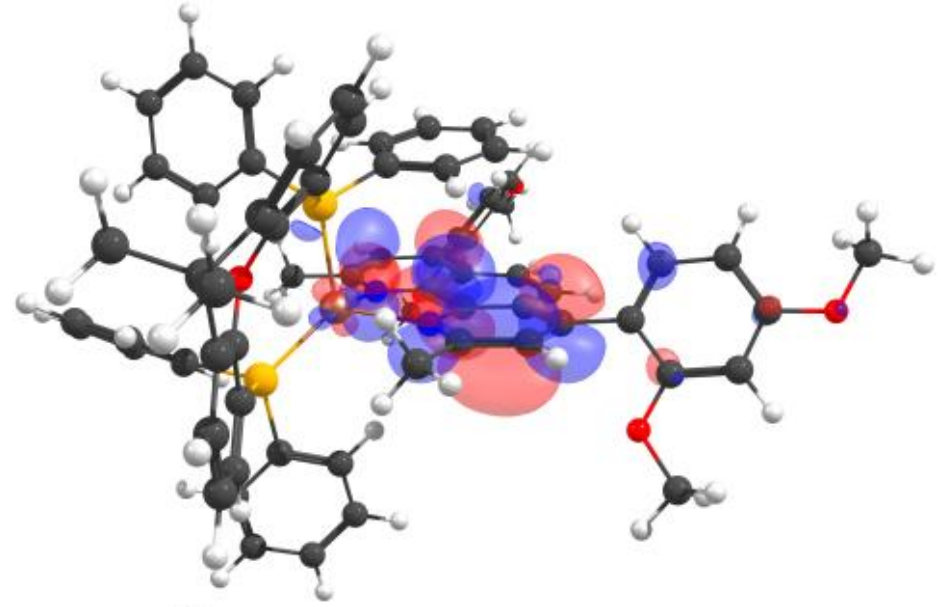	$ML_{phen}CT$
S_8	349	3.550	0.0352	87	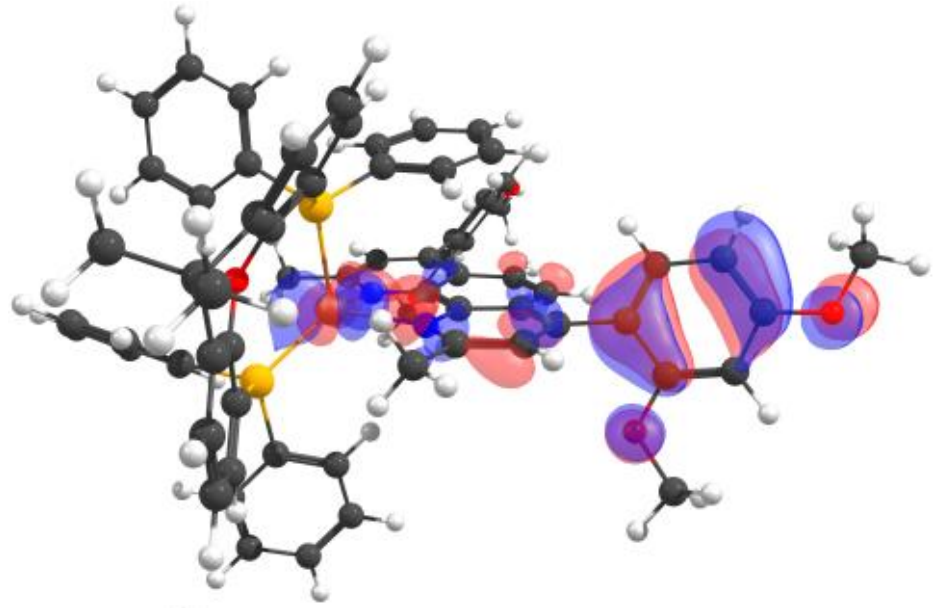	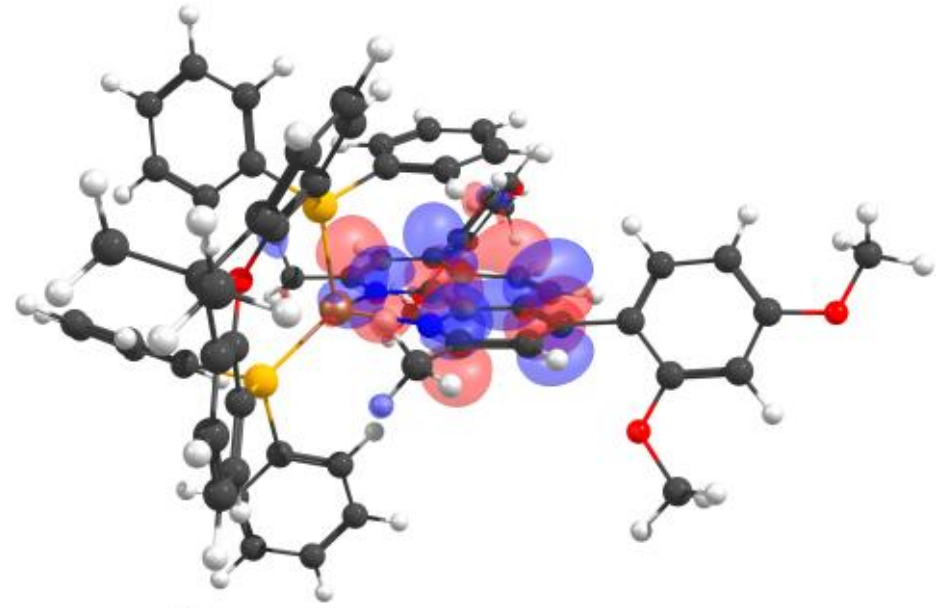	$IL_{py\rightarrow phen}CT$
S_9	342	3.629	0.0026	99	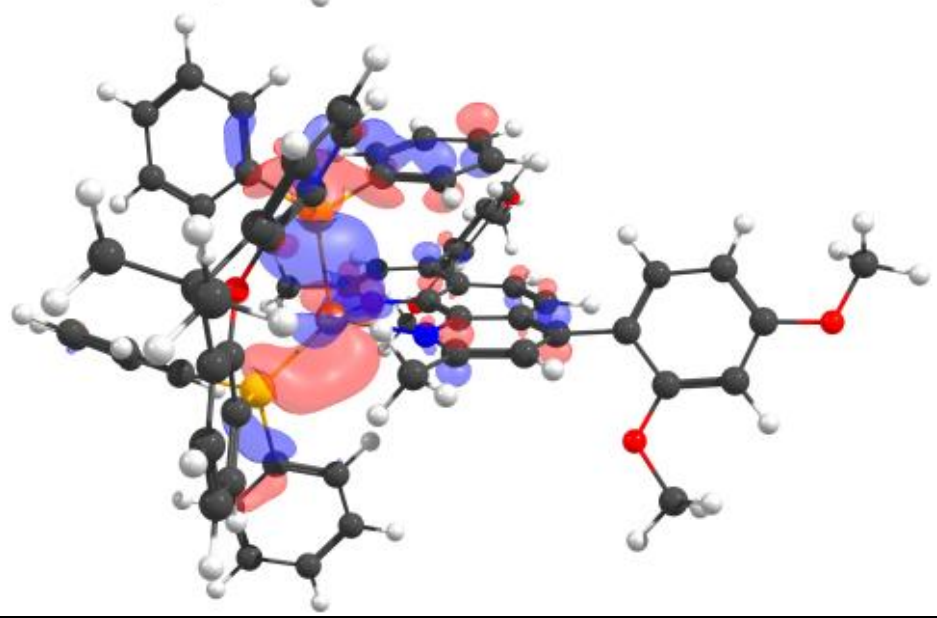	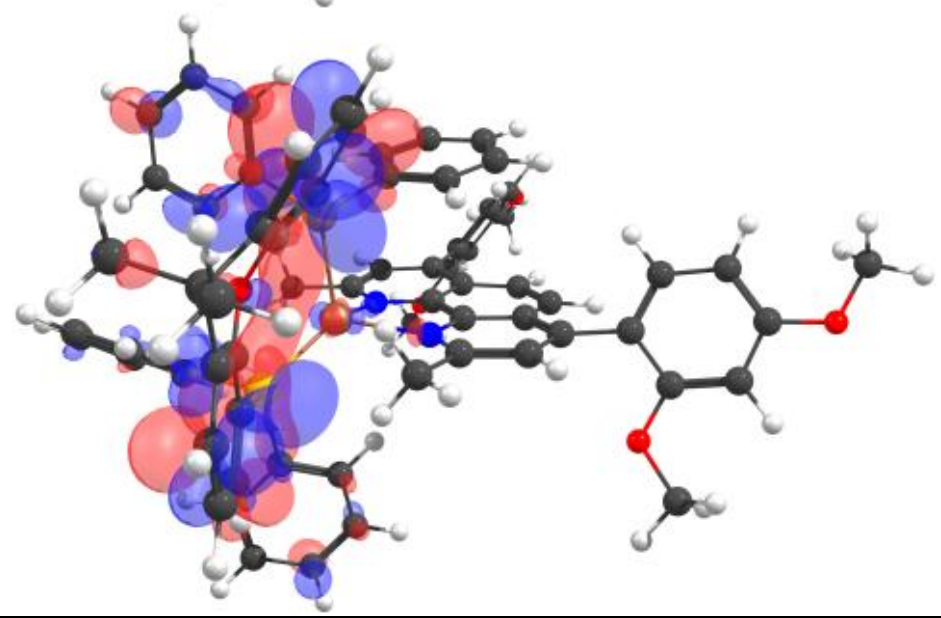	$ML_{xant}CT$

state	λ / nm	ΔE / eV	f	cont. / %	initial state	final state	character
S_{10}	340	3.652	0.0101	97	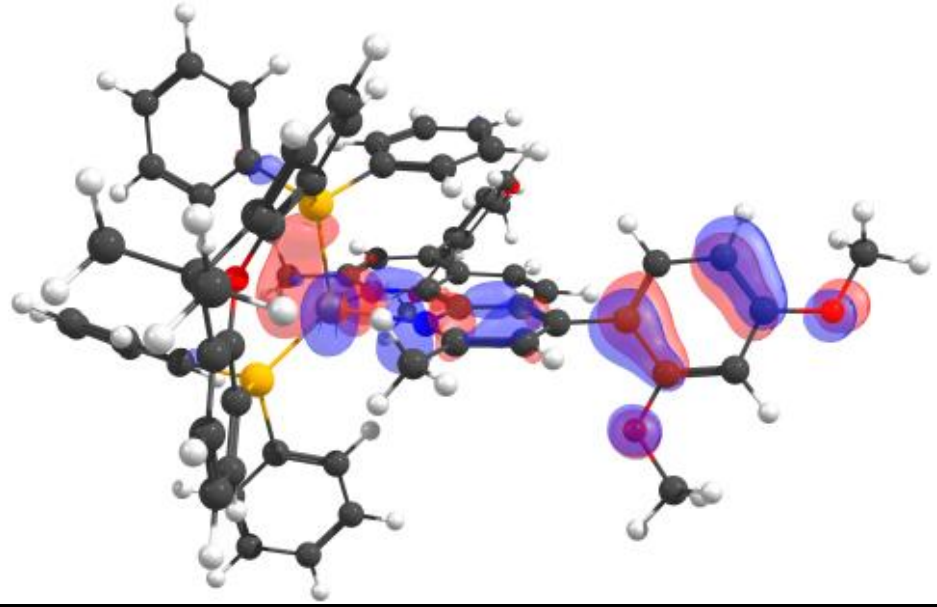	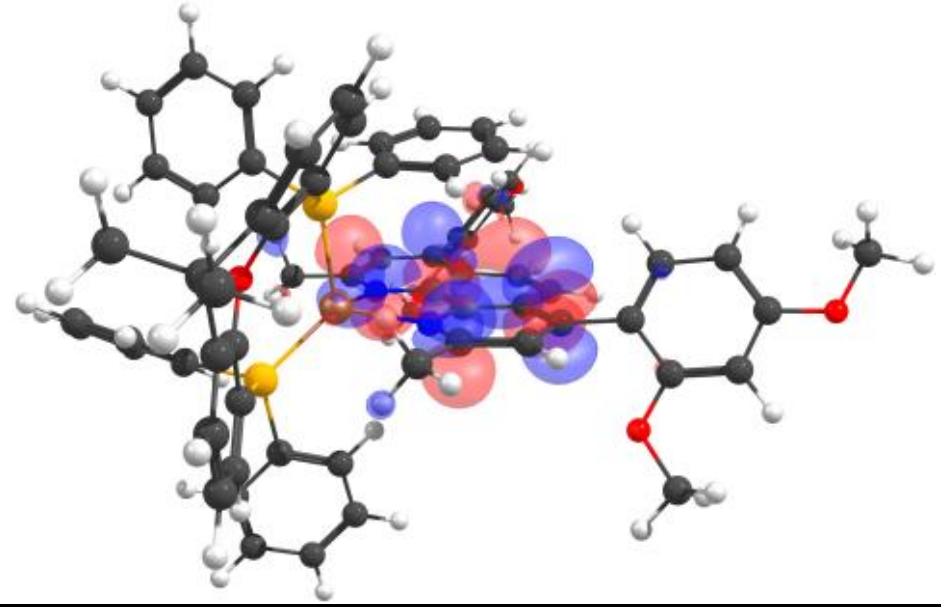	$IL_{py \rightarrow phen}CT$ and $ML_{phen}CT$

Table S5.2 Excitation energies (ΔE in eV), wavelengths (λ in nm) and oscillator strengths (f) of the first ten singlet-singlet vertical excitations of **C26**. The transitions are visualized in as natural transition orbitals (NTOs) of the initial and final state with the highest contribution (cont. in %) of each transition is displayed.

state	λ / nm	ΔE / eV	f	cont. / %	initial state	final state	character
S_1	419	2.956	0.0807	99	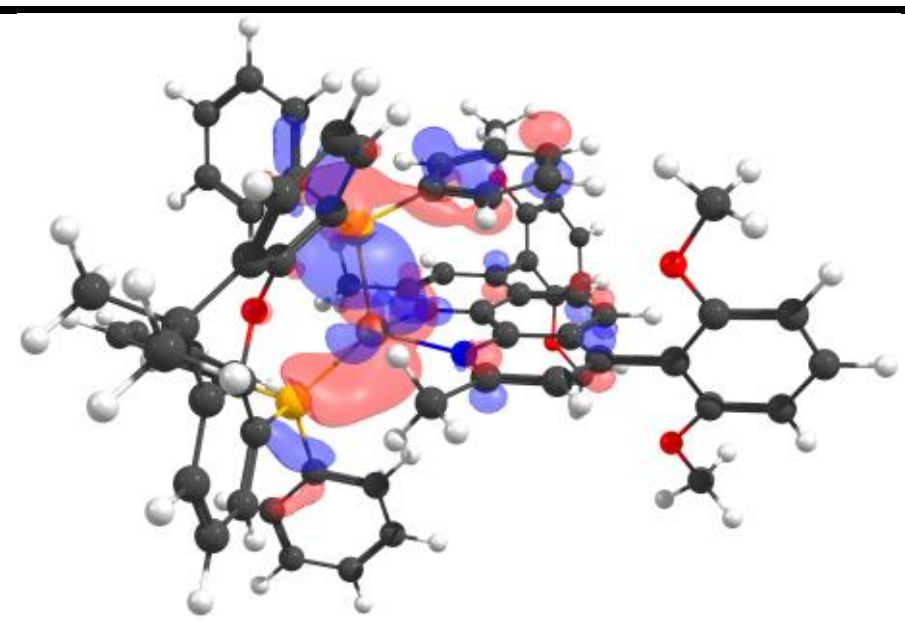	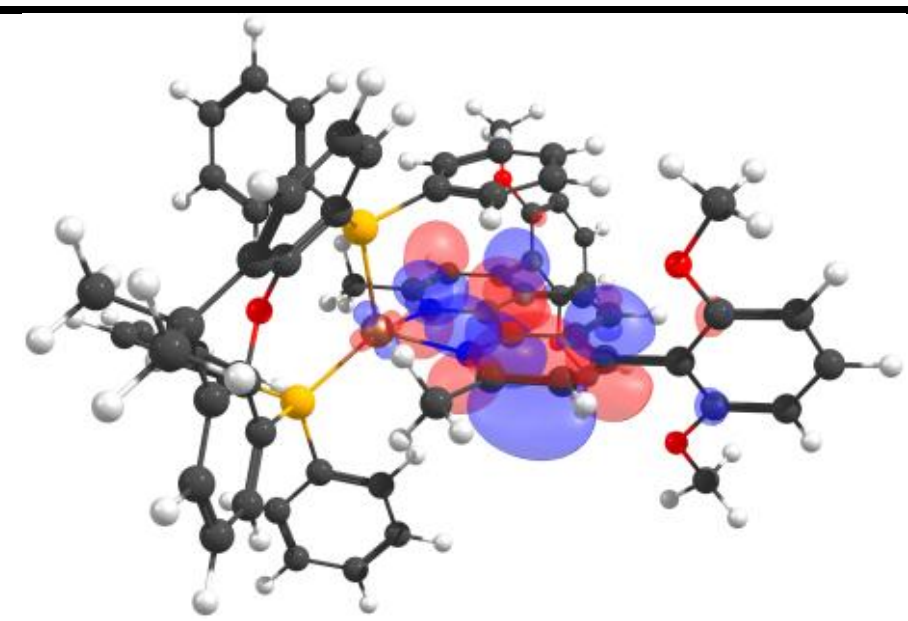	$ML_{phen}CT$

state	λ / nm	ΔE / eV	f	cont. / %	initial state	final state	character
S_2	389	3.188	0.0292	98	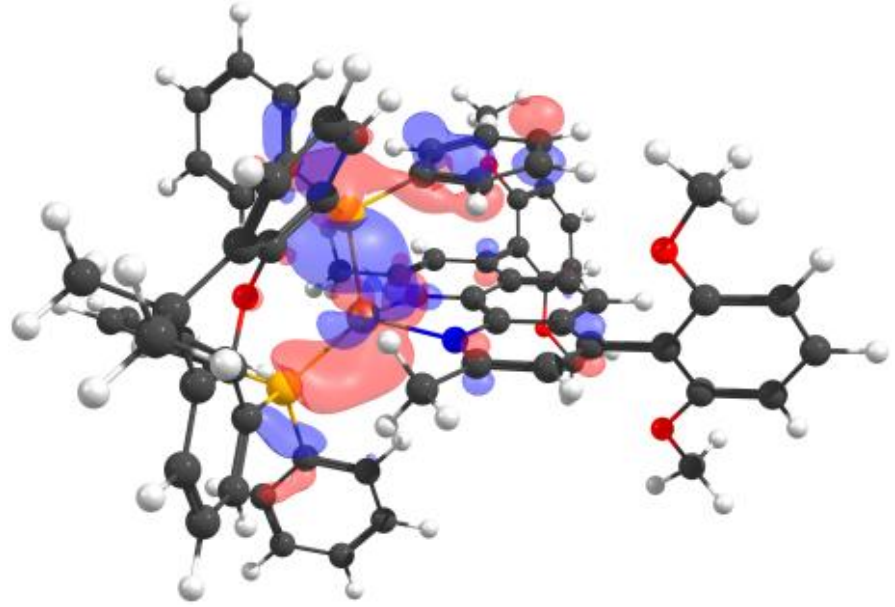	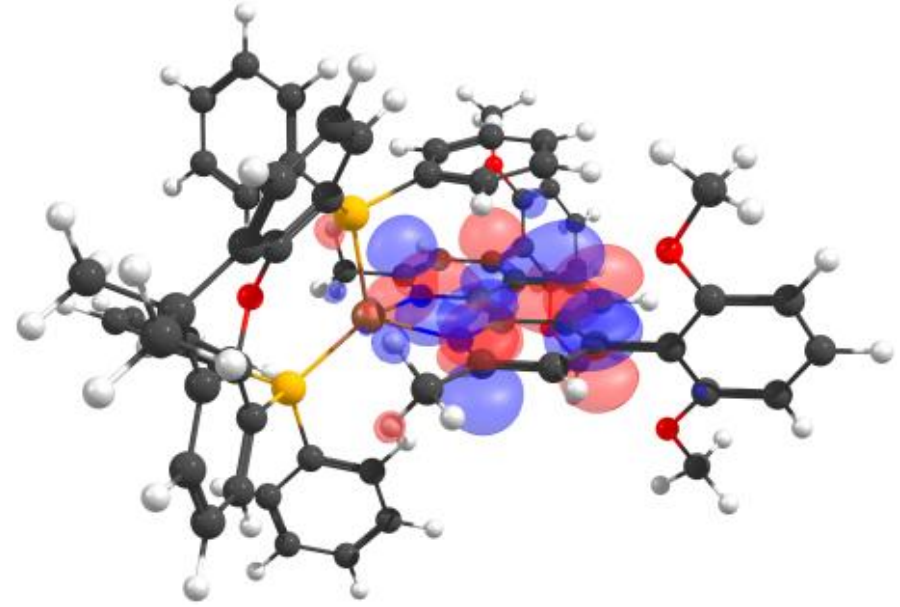	$ML_{phen}CT$
S_3	381	3.256	0.0070	98	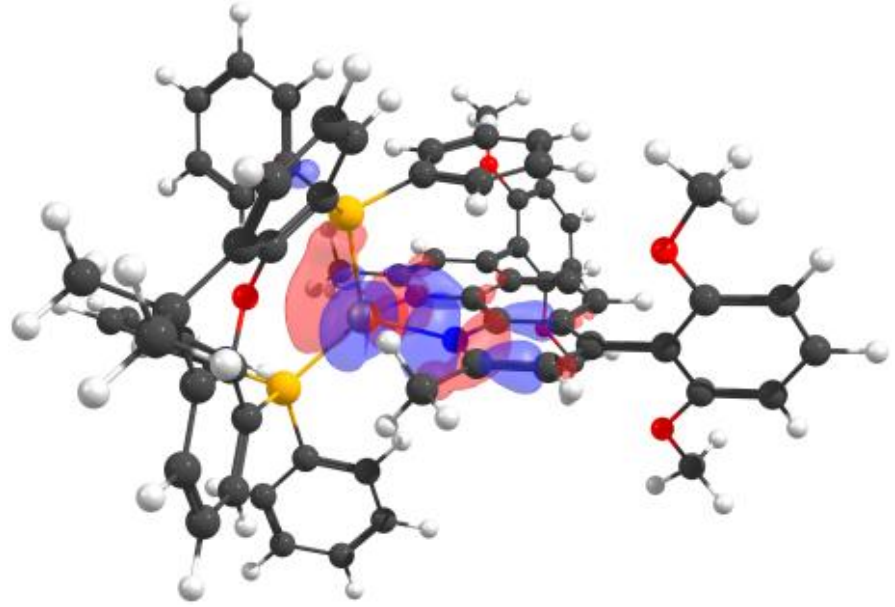	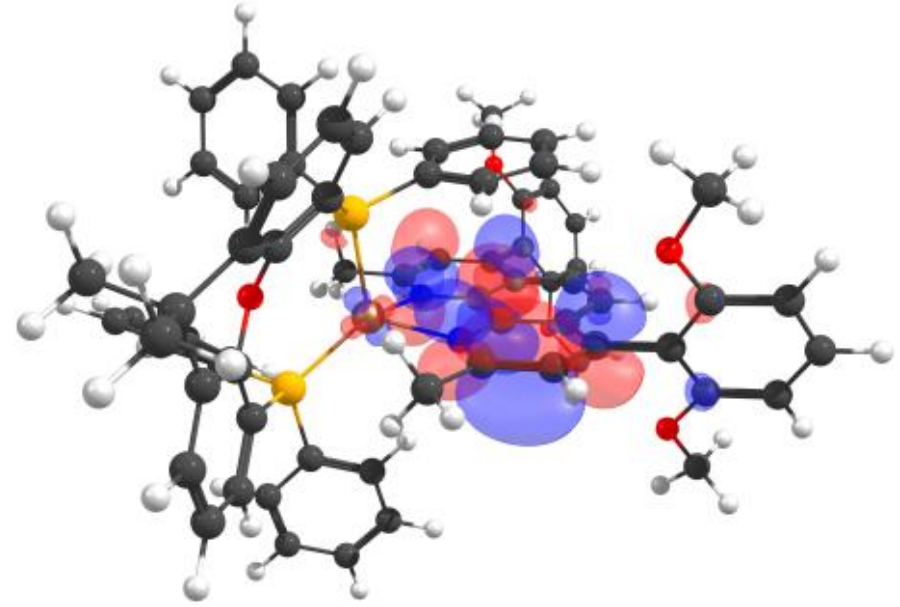	$ML_{phen}CT$
S_4	359	3.452	0.0123	97	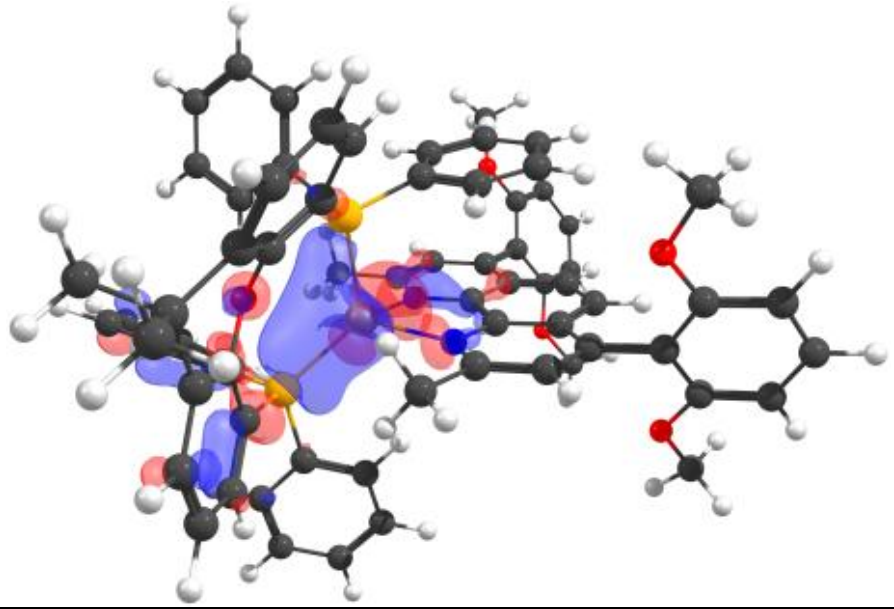	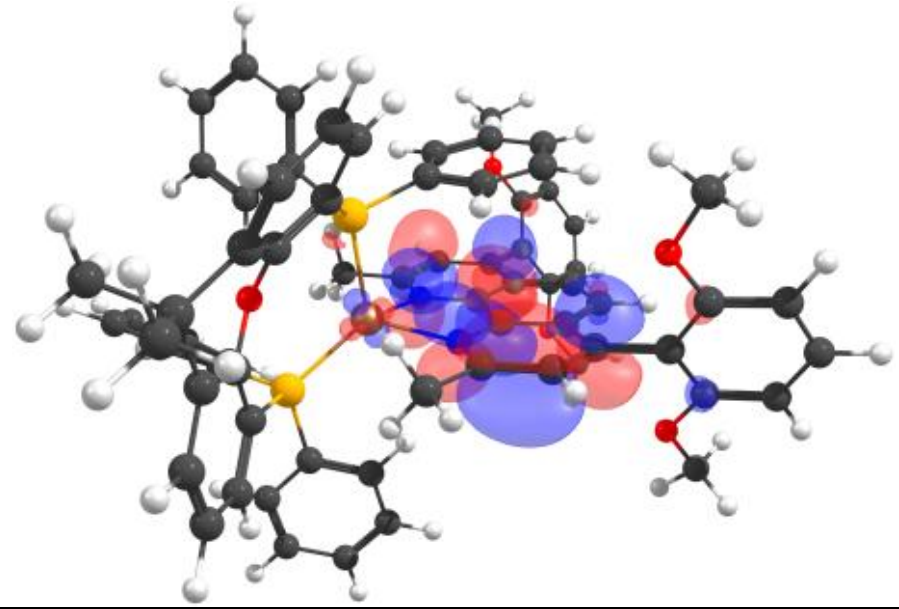	$ML_{phen}CT$

state	λ / nm	ΔE / eV	f	cont. / %	initial state	final state	character
S_5	346	3.585	0.0020	95	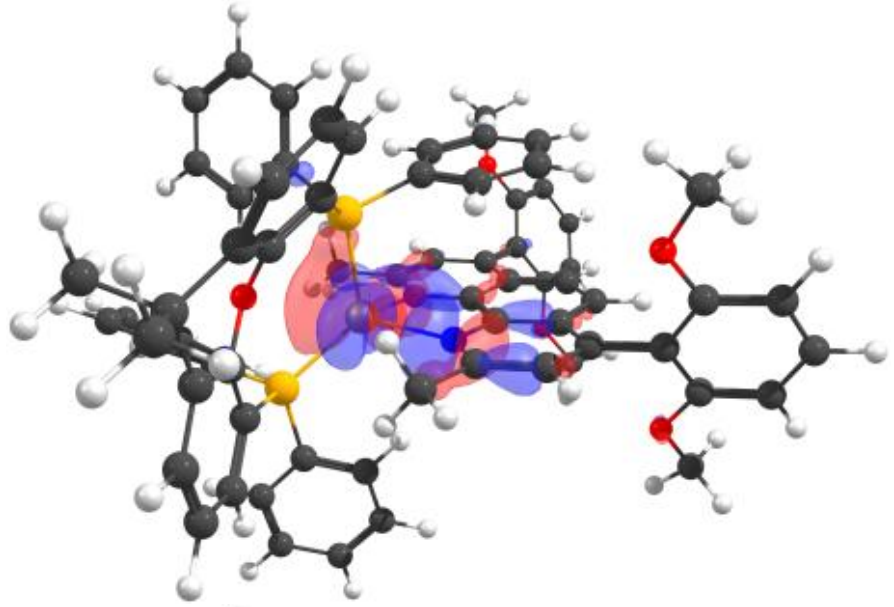	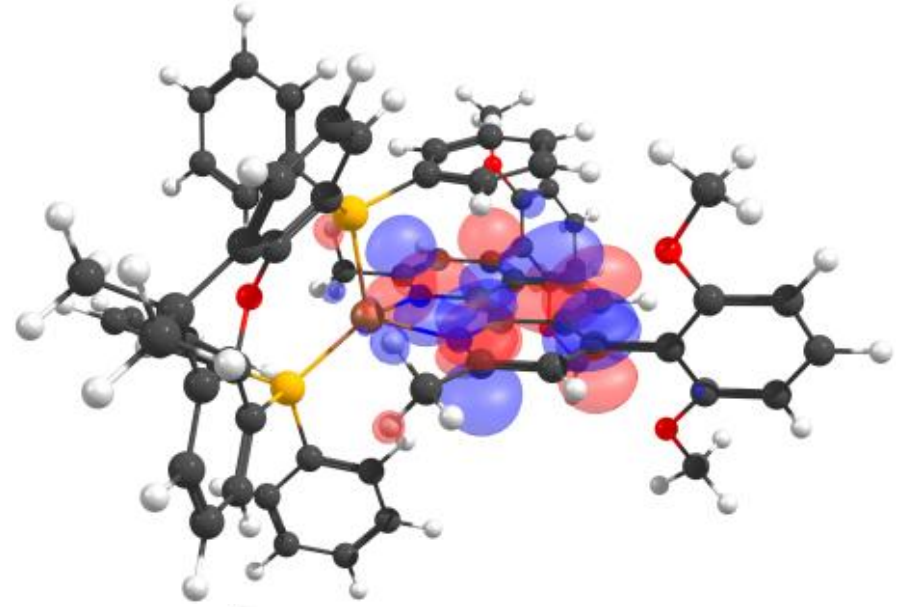	$ML_{phen}CT$
S_6	343	3.613	0.0030	98	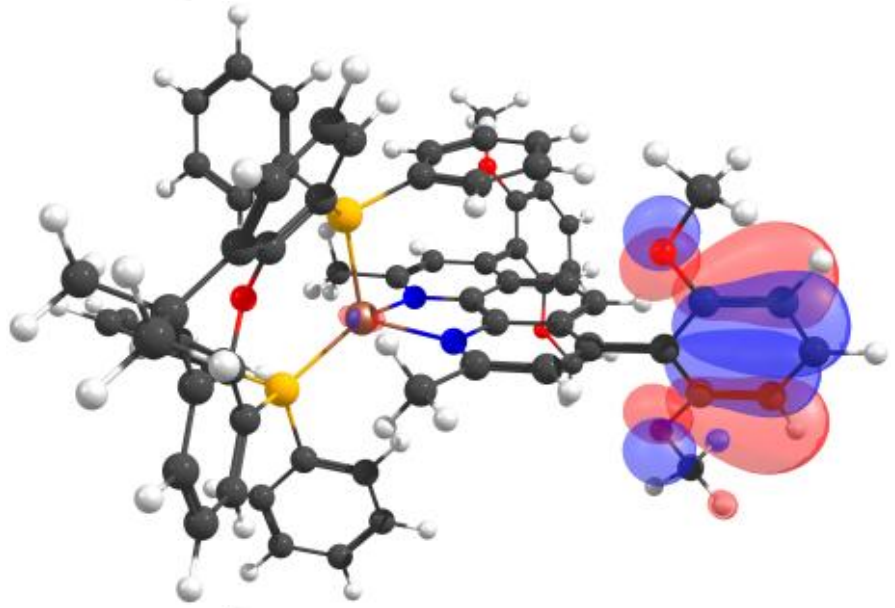	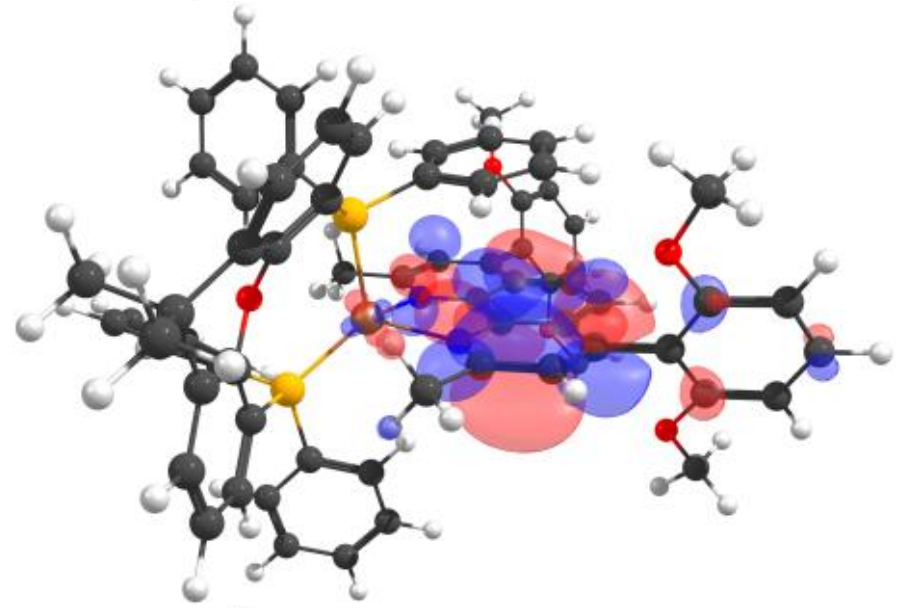	$IL_{py\rightarrow phen}CT$
S_7	342	3.630	0.0050	99	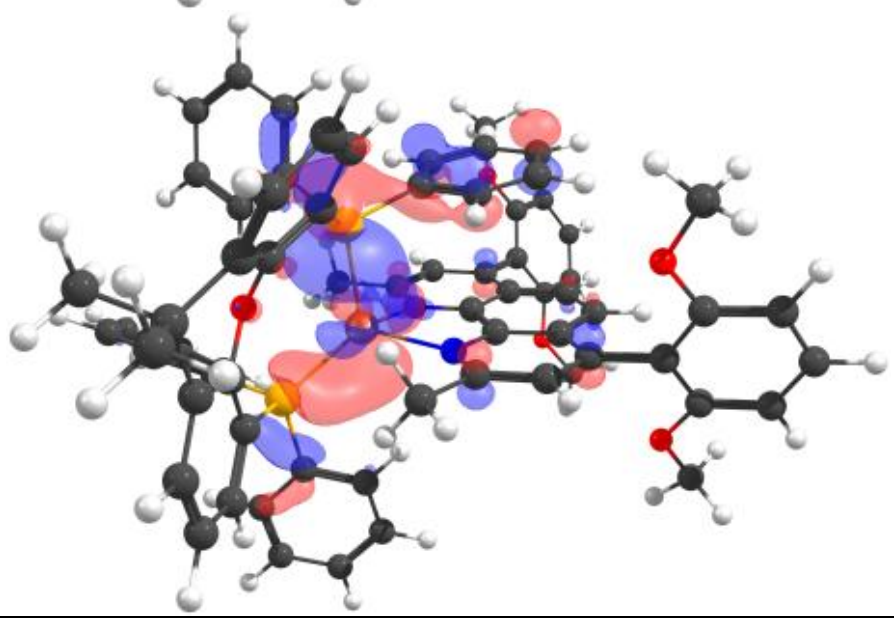	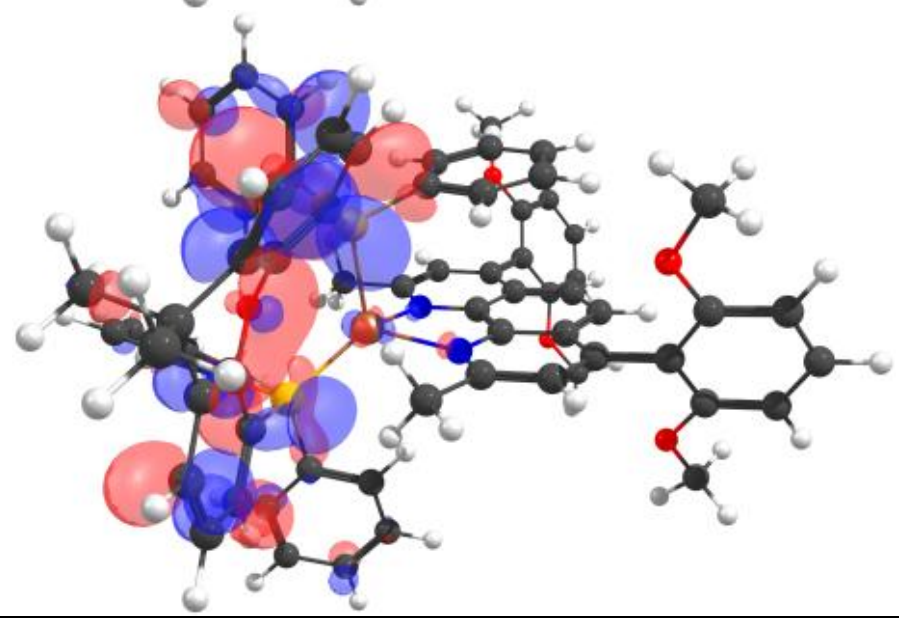	$ML_{xant}CT$

state	λ / nm	ΔE / eV	f	cont. / %	initial state	final state	character
S_8	340	3.644	0.0037	99	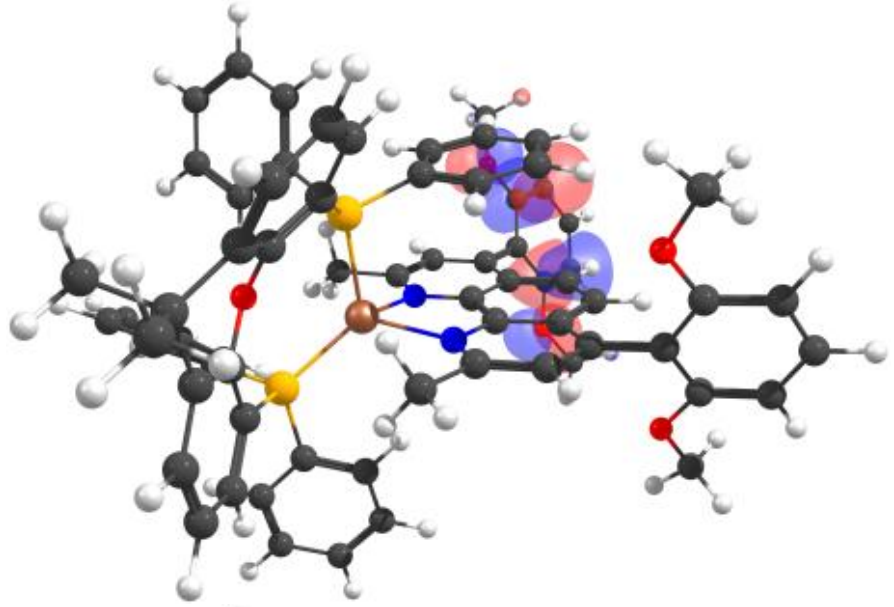	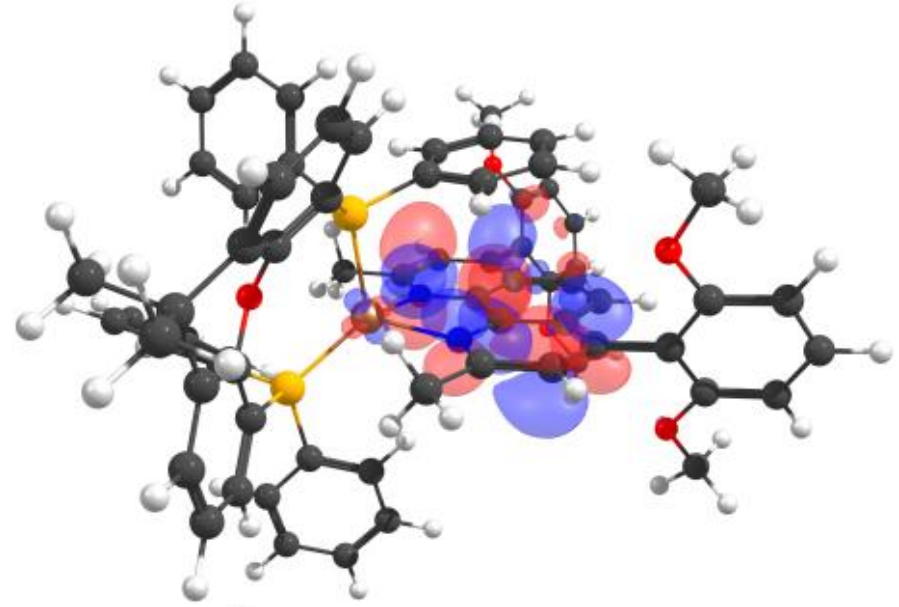	$IL_{py \rightarrow phen}CT$
S_9	334	3.710	0.0496	92	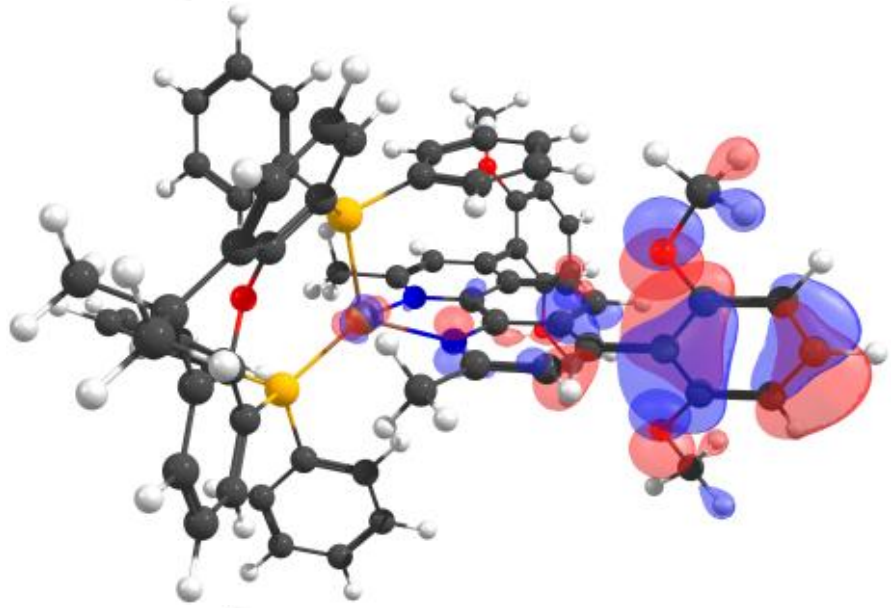	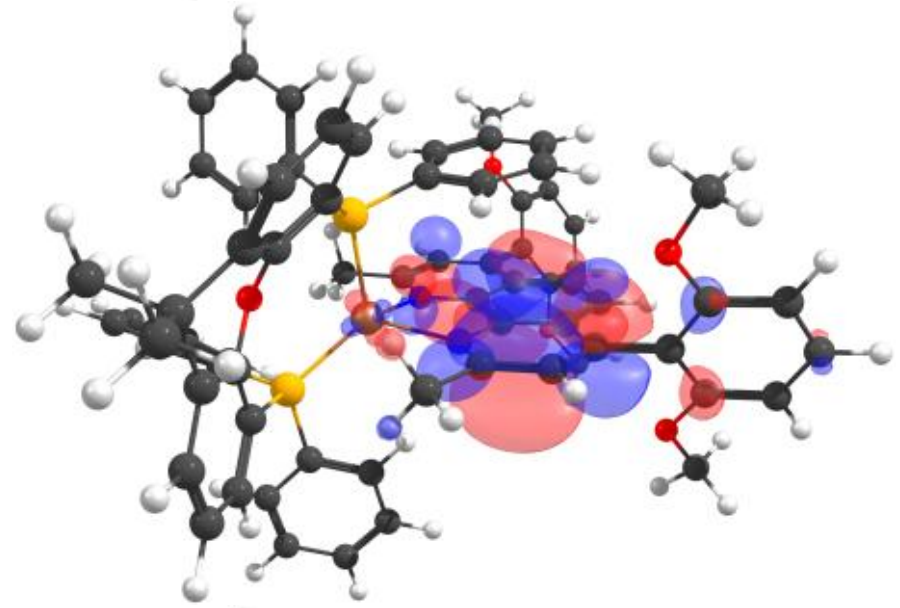	$IL_{py \rightarrow phen}CT$
S_{10}	332	3.732	0.0333	66	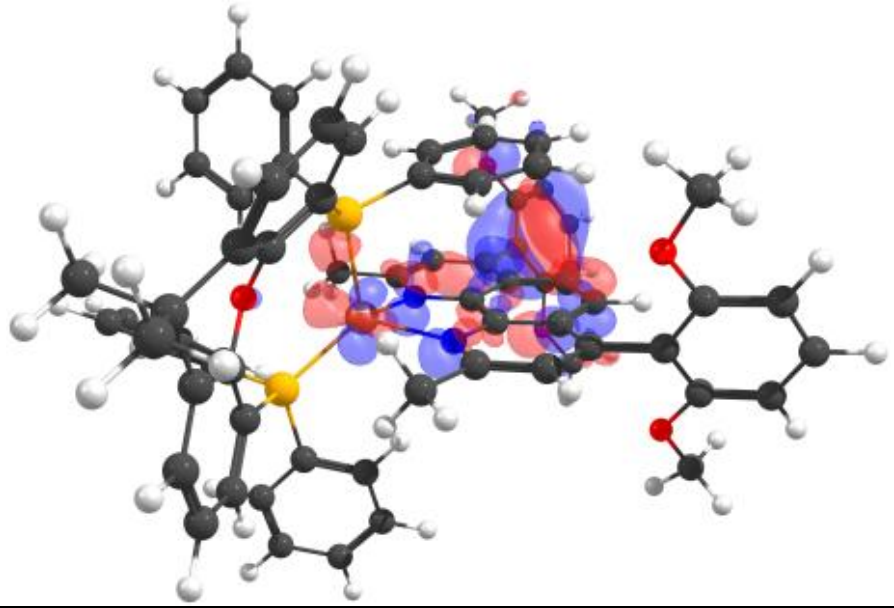	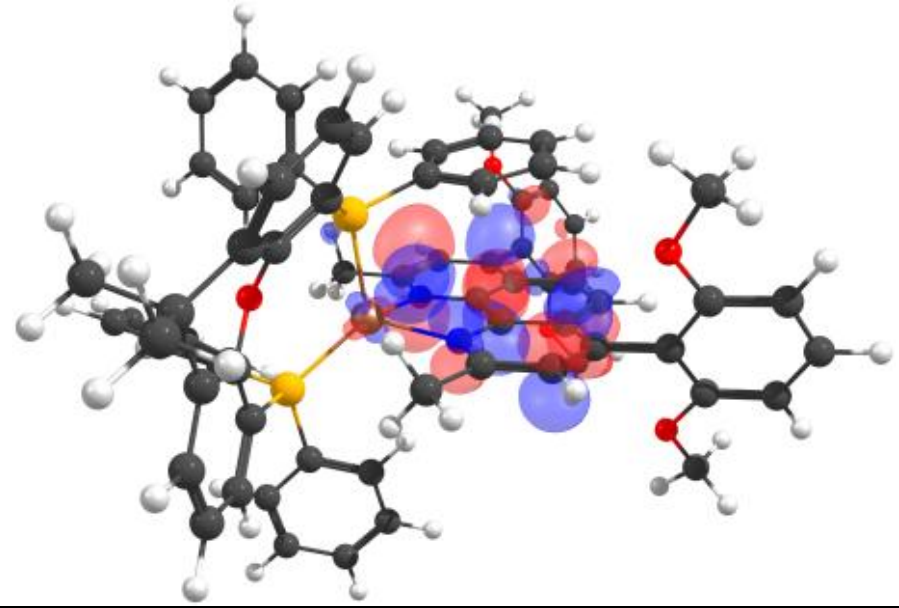	$IL_{py \rightarrow phen}CT$

Table S5.3 Excitation energies (Δ*E* in eV), wavelengths (*λ* in nm) and oscillator strengths (*f*) of the first ten singlet-singlet vertical excitations of **C35**. The transitions are visualized in as natural transition orbitals (NTOs) of the initial and final state with the highest contribution (cont. in %) of each transition is displayed.

state	*λ* / nm	Δ*E* / eV	*f*	cont. / %	initial state	final state	character
S_1	432	2.873	0.0988	99	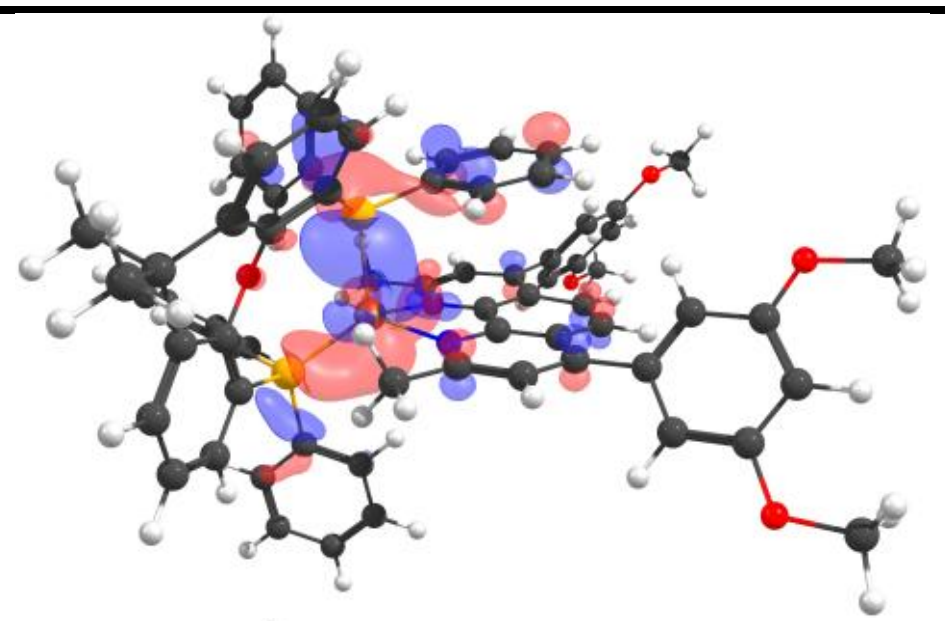	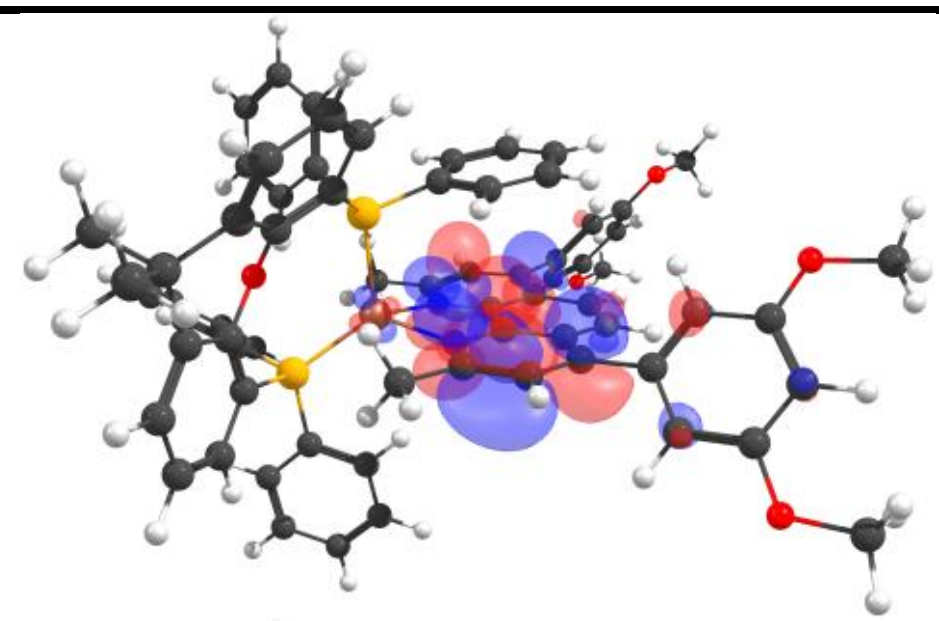	$ML_{phen}CT$
S_2	398	3.112	0.0337	92	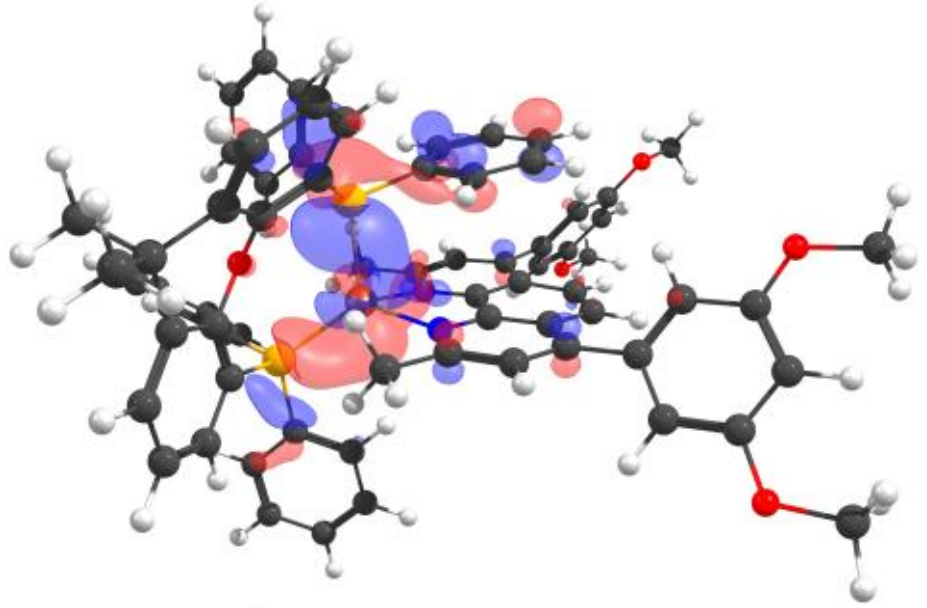	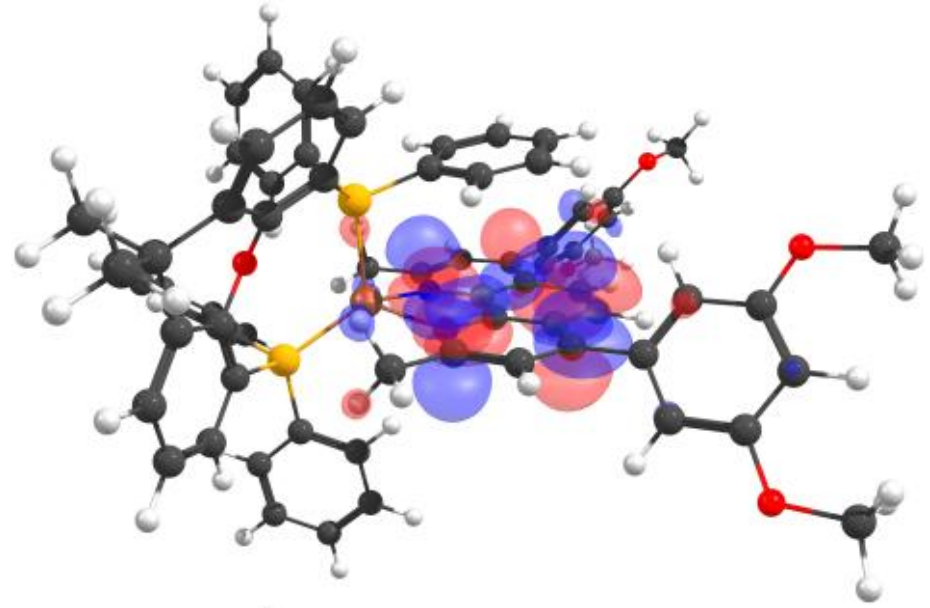	$ML_{phen}CT$
S_3	395	3.139	0.0083	95	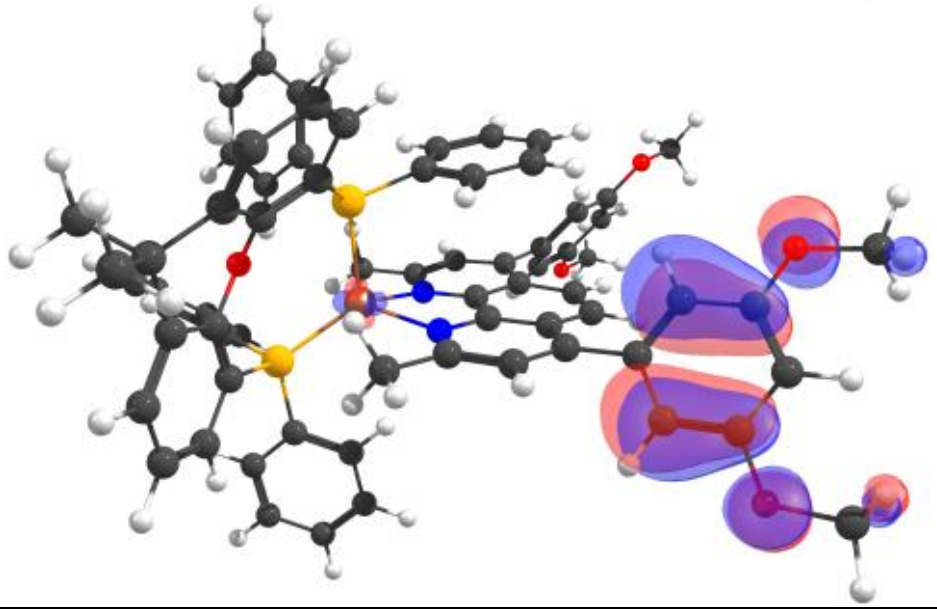	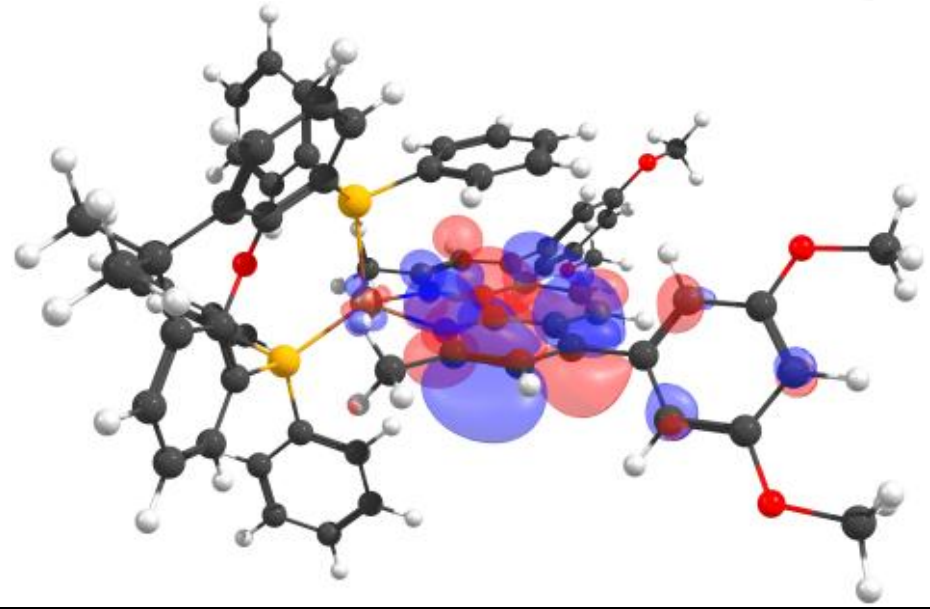	$IL_{py\rightarrow phen}CT$

state	λ / nm	ΔE / eV	f	cont. / %	initial state	final state	character
S_4	391	3.168	0.0072	98	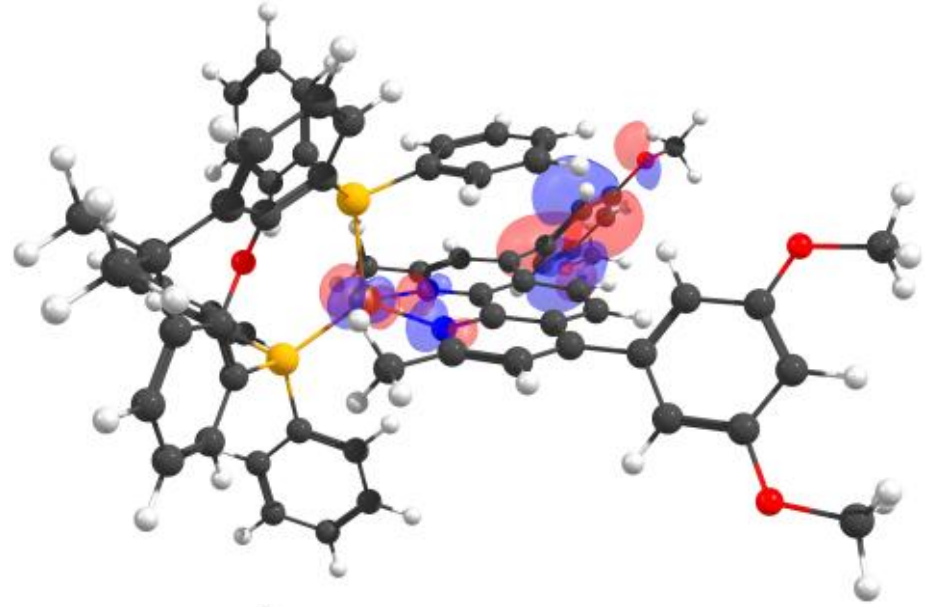	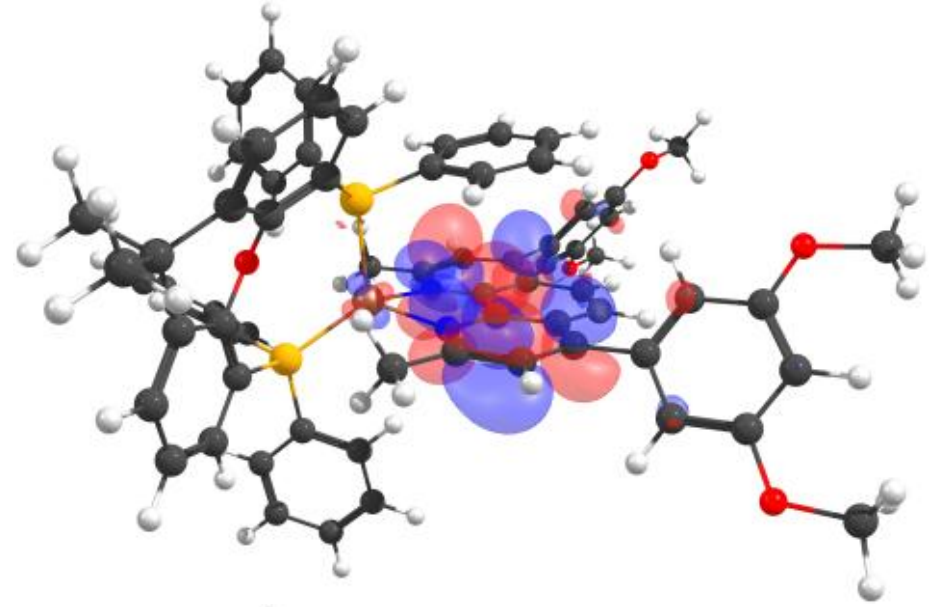	$IL_{py \rightarrow phen}CT$
S_5	388	3.200	0.0096	96	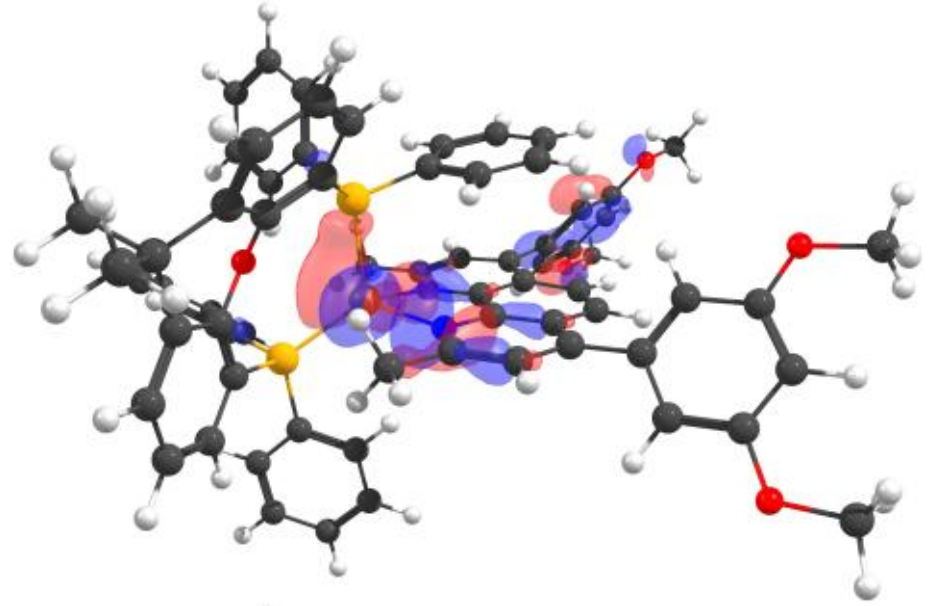	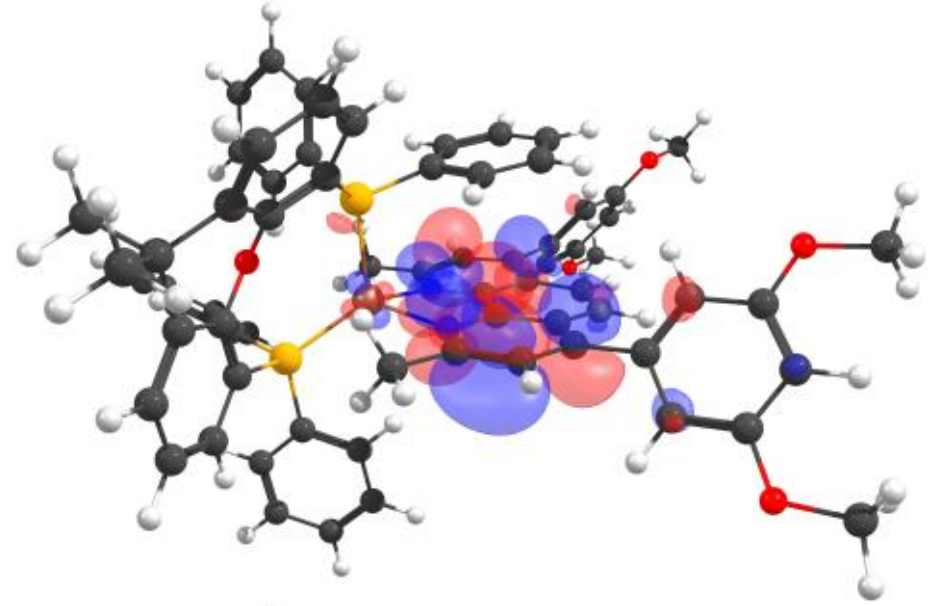	$ML_{phen}CT$
S_6	371	3.346	0.0079	99	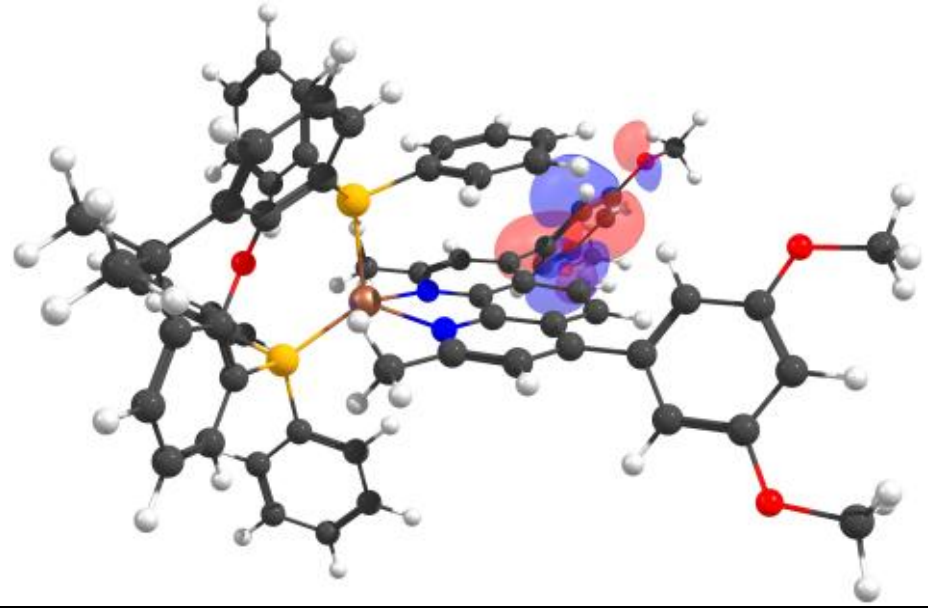	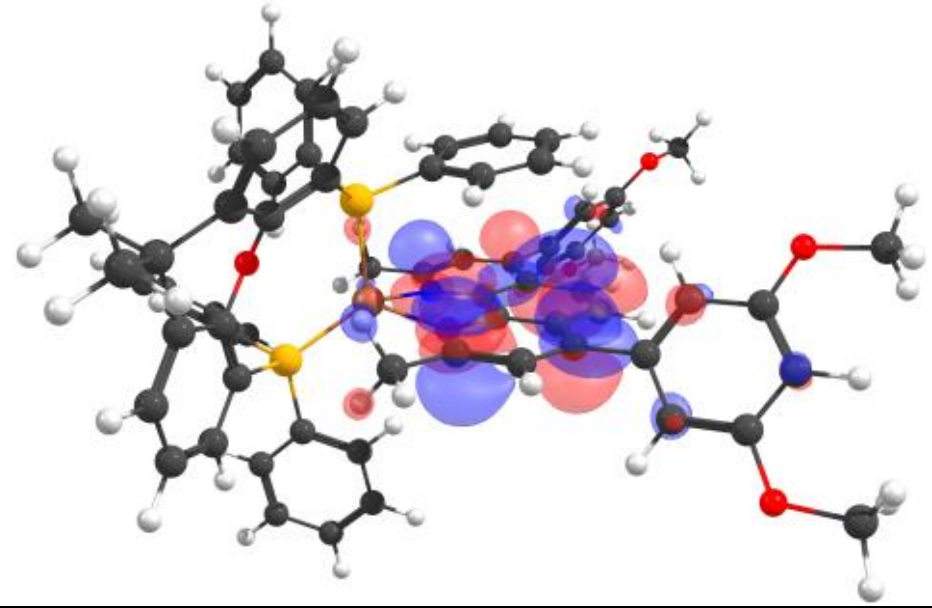	$IL_{py \rightarrow phen}CT$

state	λ / nm	ΔE / eV	f	cont. / %	initial state	final state	character
S_7	366	3.391	0.0098	98	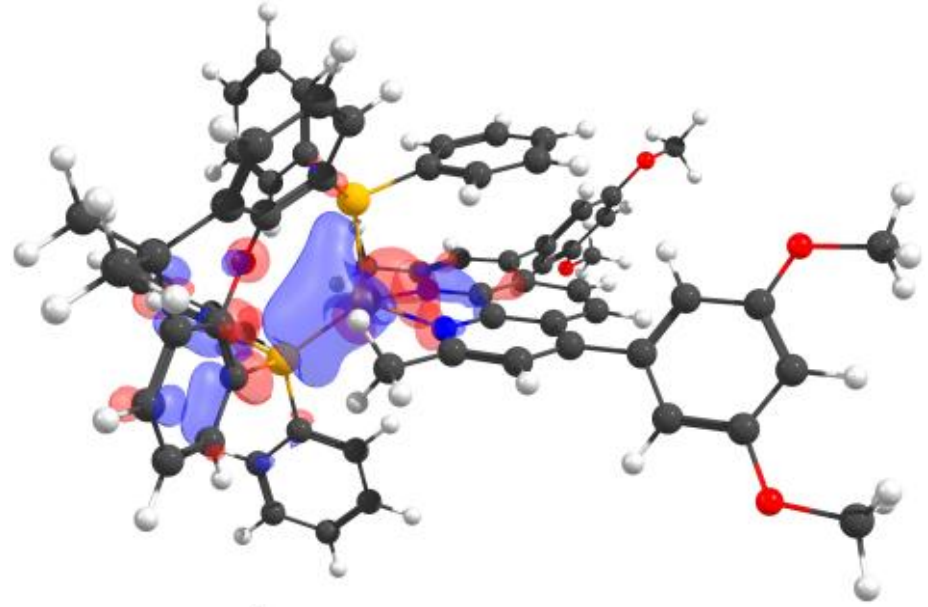	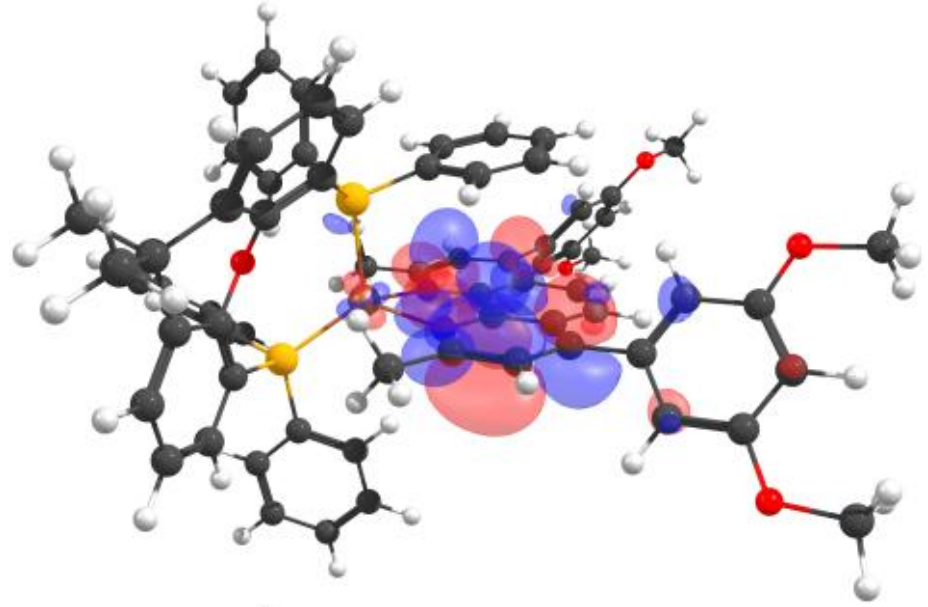	$ML_{phen}CT$
S_8	362	3.421	0.0111	99	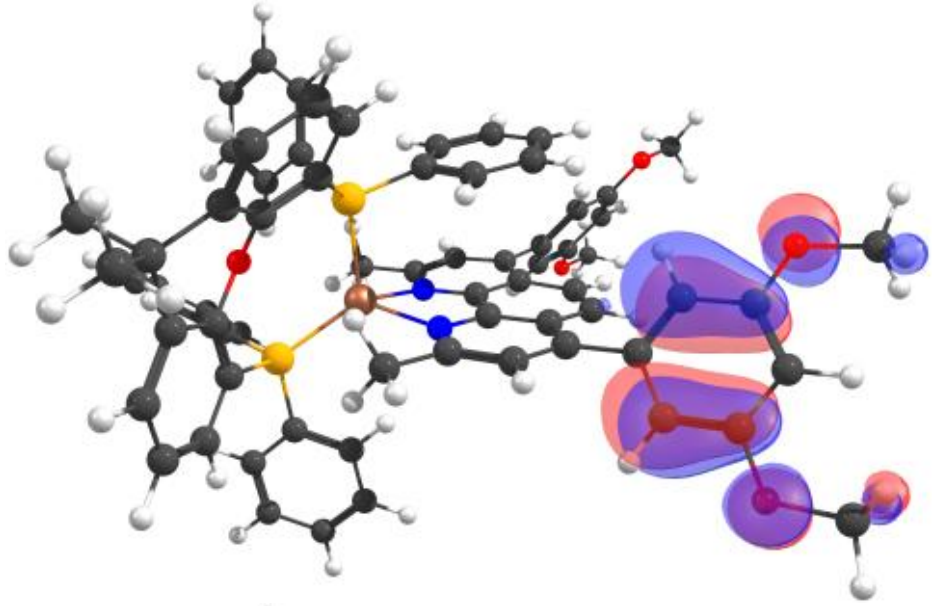	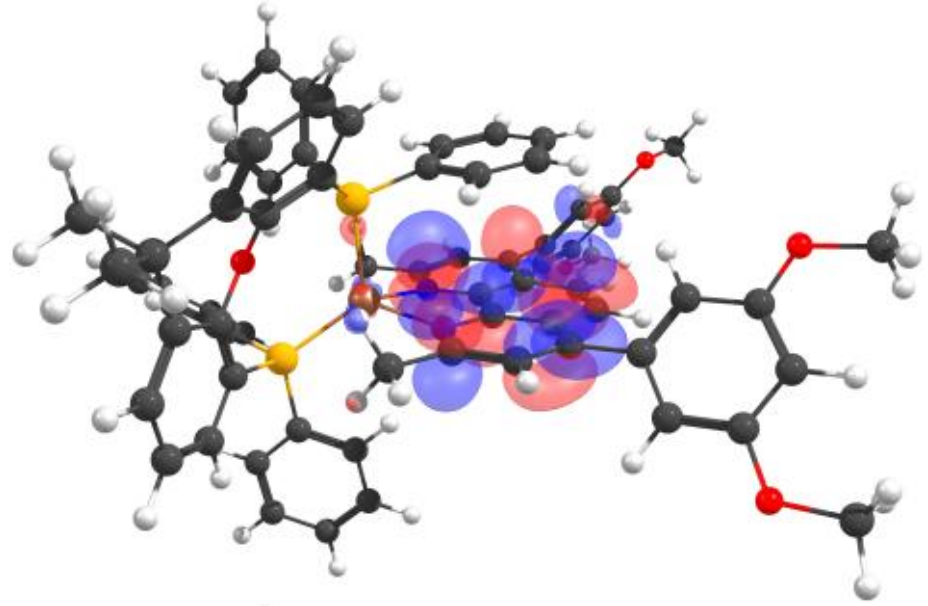	$IL_{py \rightarrow phen}CT$
S_9	350	3.542	0.0072	98	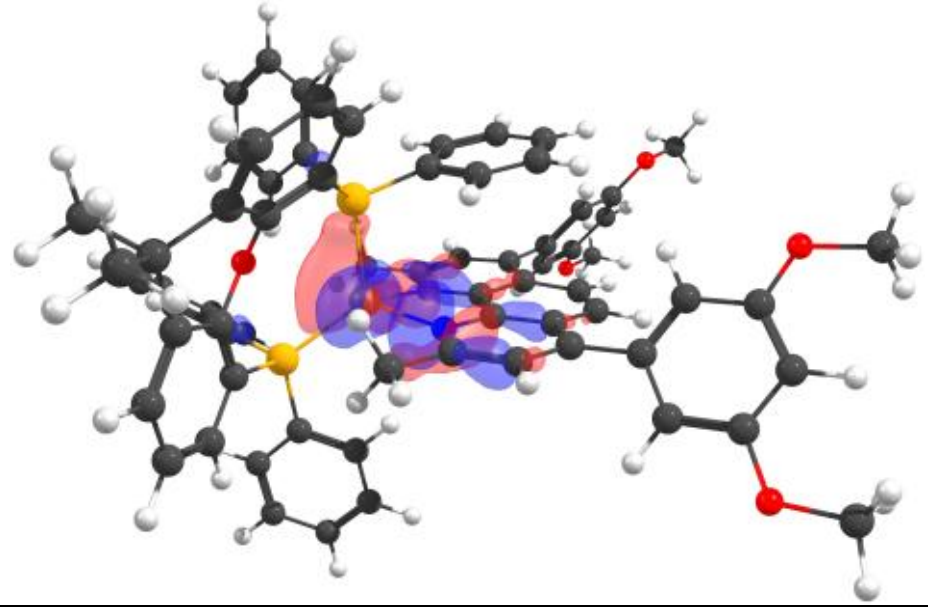	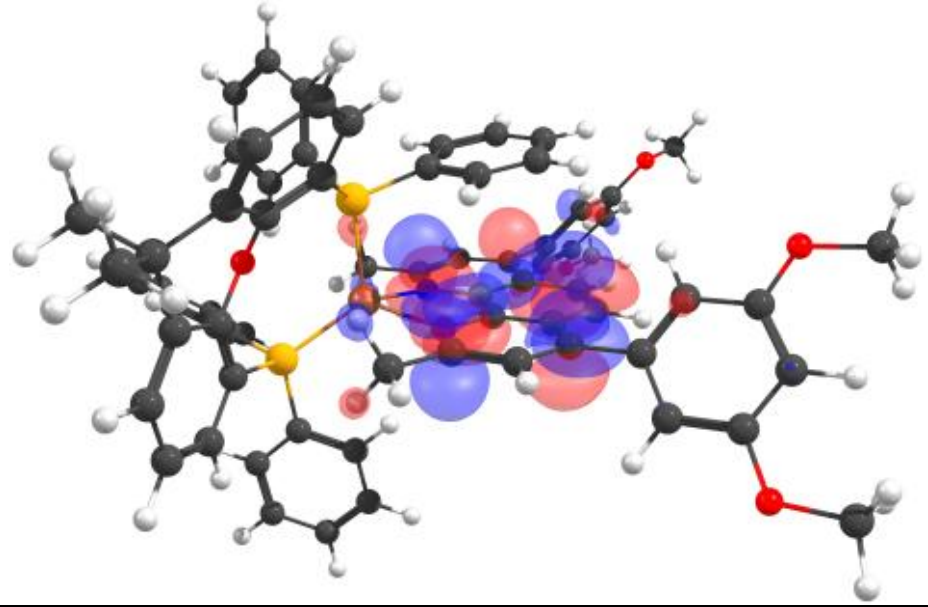	$ML_{phen}CT$

state	λ / nm	ΔE / eV	f	cont. / %	initial state	final state	character
S_{10}	338	3.667	0.0047	98	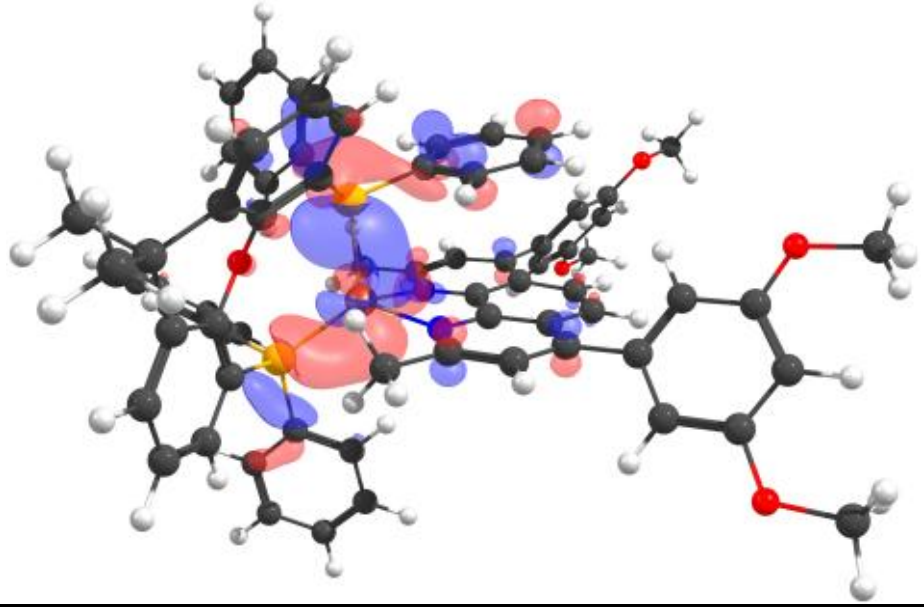	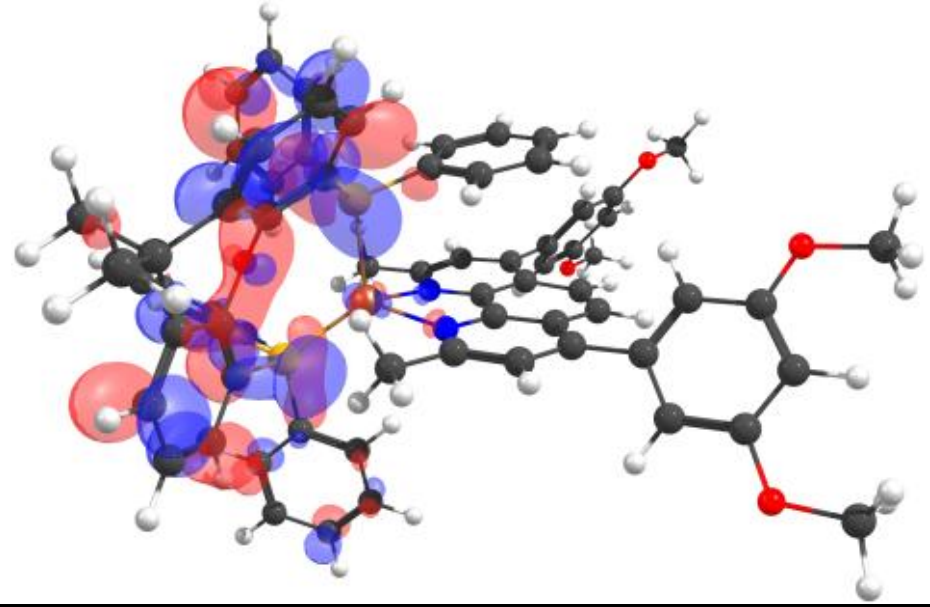	$ML_{xant}CT$

Table S5.4 Excitation energies (ΔE in eV), wavelengths (λ in nm) and oscillator strengths (f) of the first ten singlet-singlet vertical excitations of **C246**. The transitions are visualized in as natural transition orbitals (NTOs) of the initial and final state with the highest contribution (cont. in %) of each transition is displayed.

state	λ / nm	ΔE / eV	f	cont. / %	initial state	final state	character
S_1	415	2.986	0.1037	99	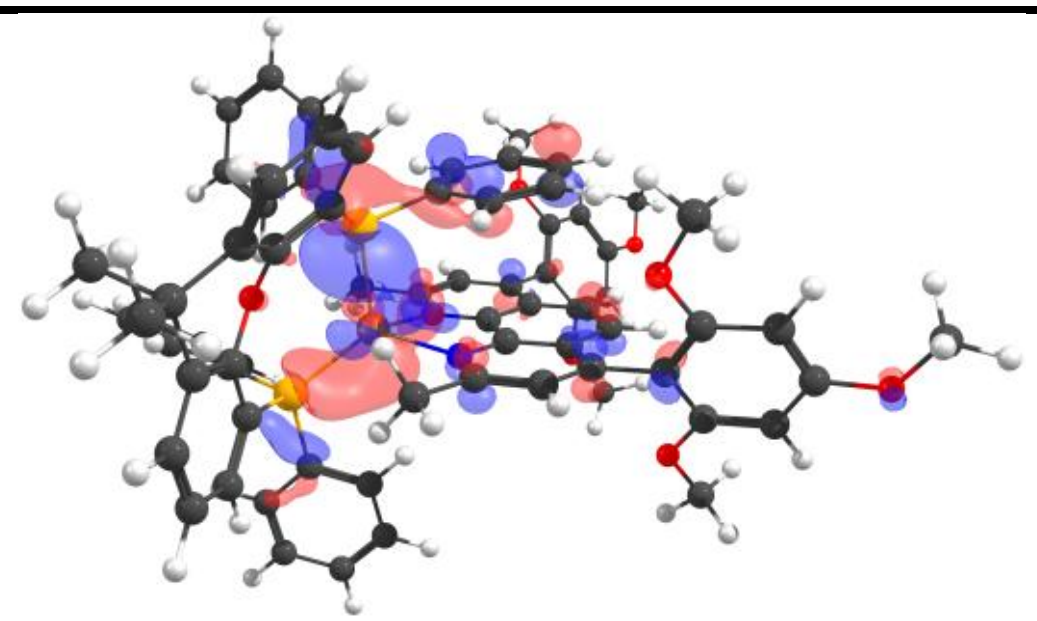	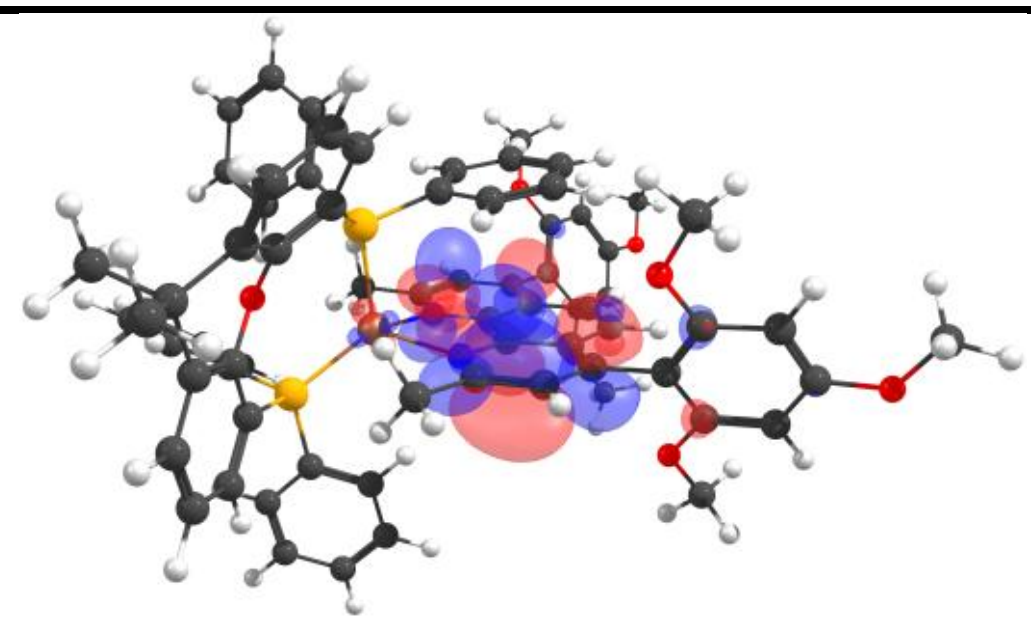	$ML_{phen}CT$

state	λ / nm	ΔE / eV	f	cont. / %	initial state	final state	character
S_2	385	3.219	0.0693	94	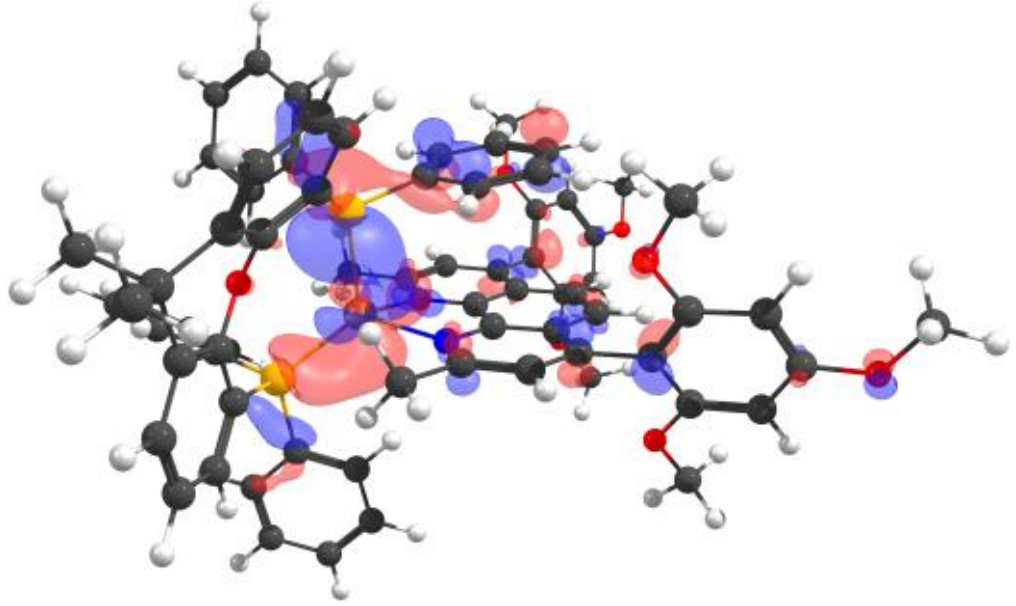	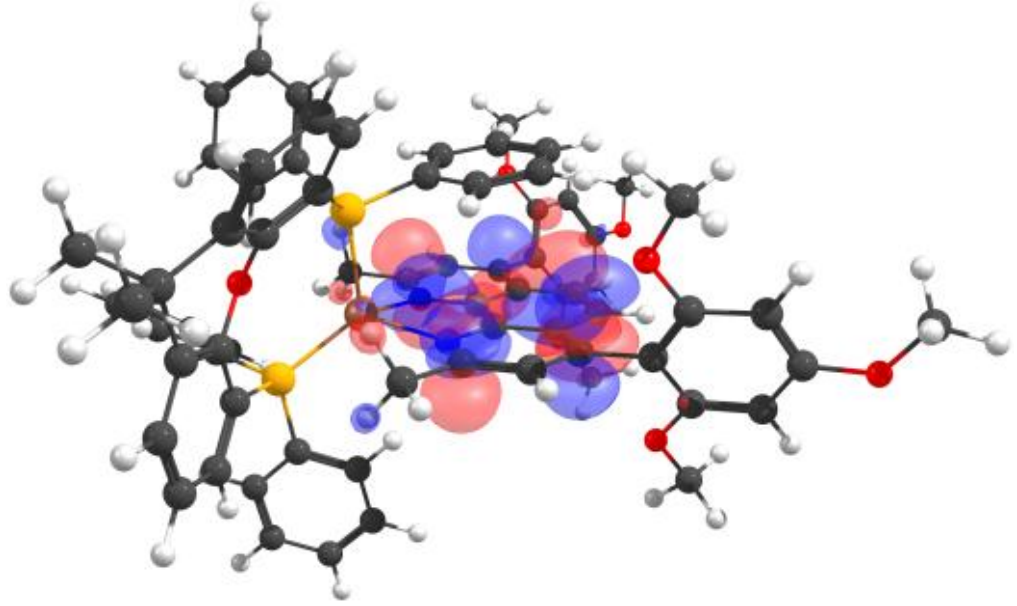	$ML_{phen}CT$
S_3	379	3.274	0.0108	98	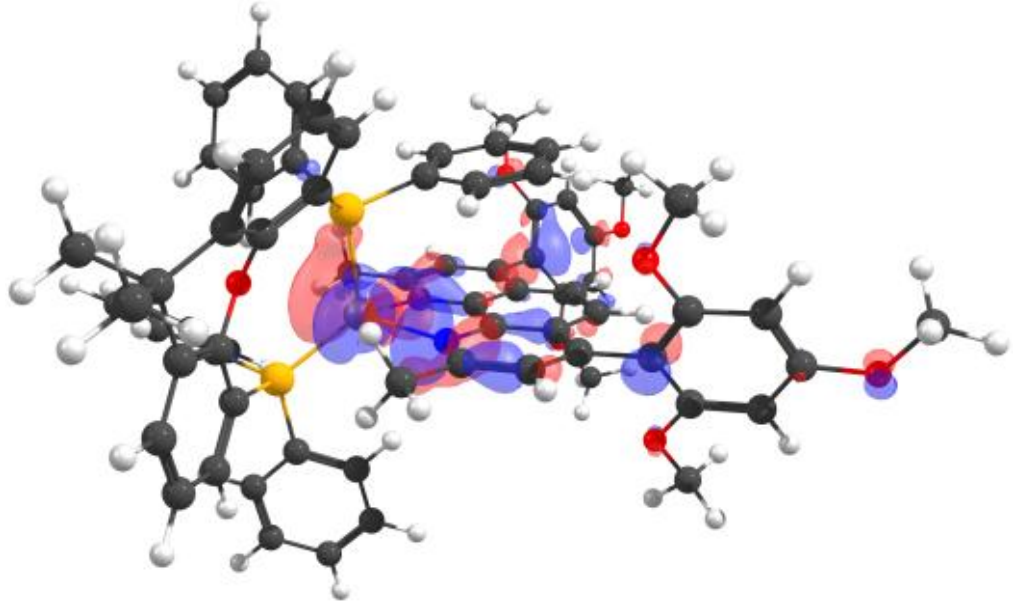	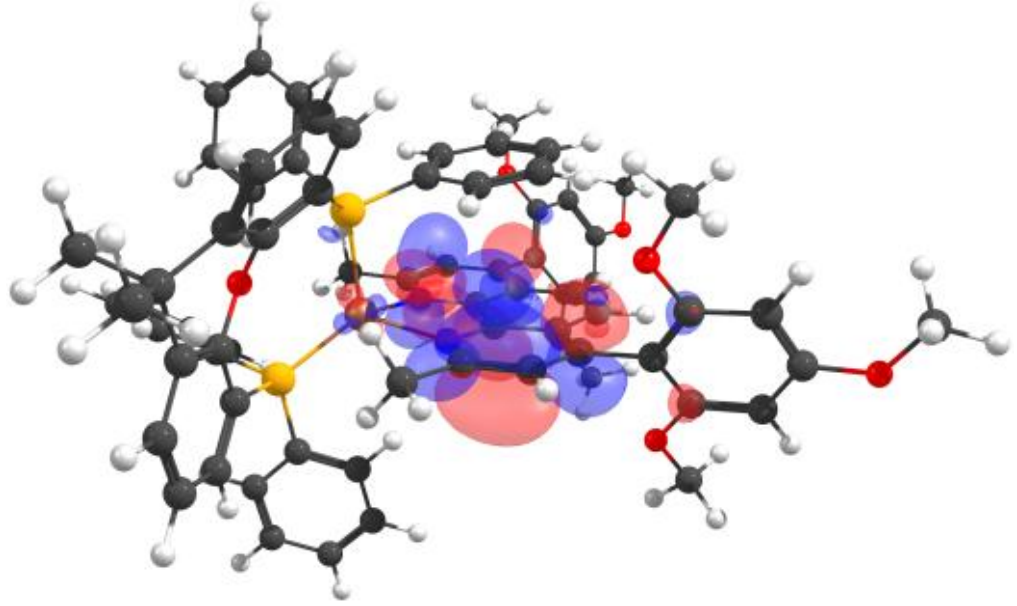	$ML_{phen}CT$
S_4	370	3.349	0.0468	95	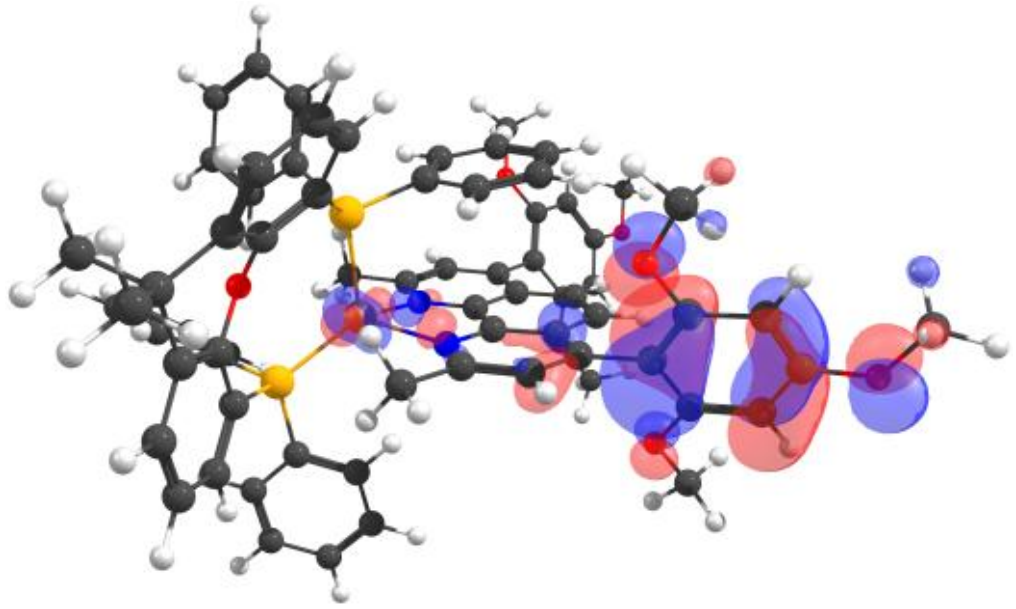	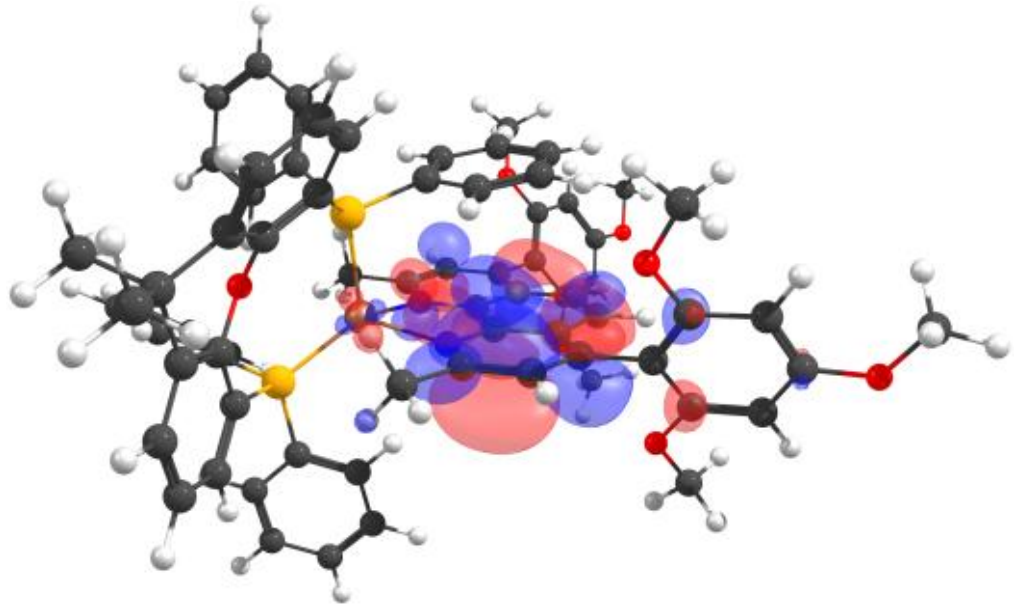	$IL_{py\rightarrow phen}CT$

state	λ / nm	ΔE / eV	f	cont. / %	initial state	final state	character
S_5	365	3.395	0.0395	94	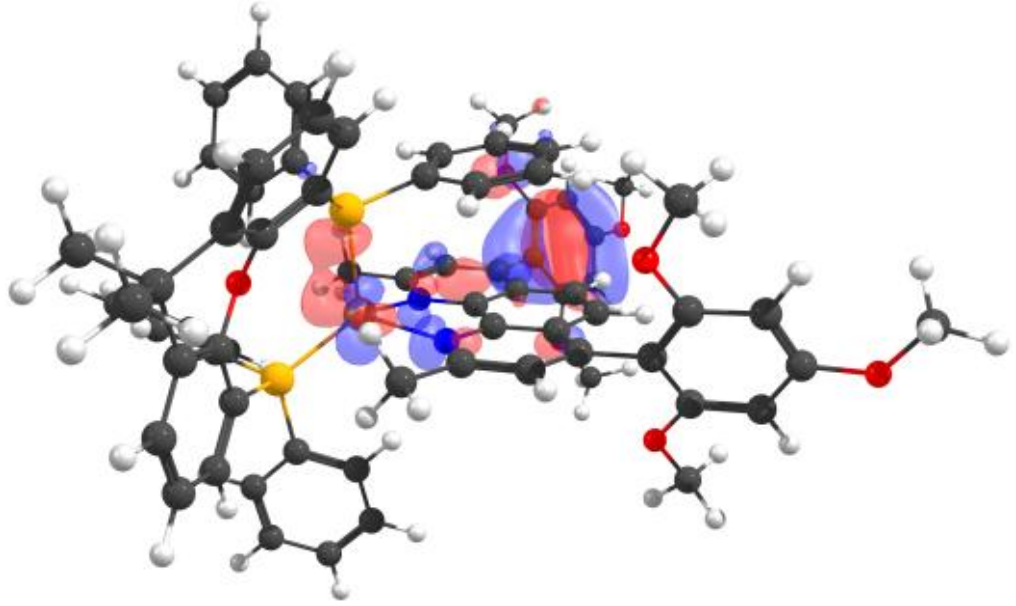	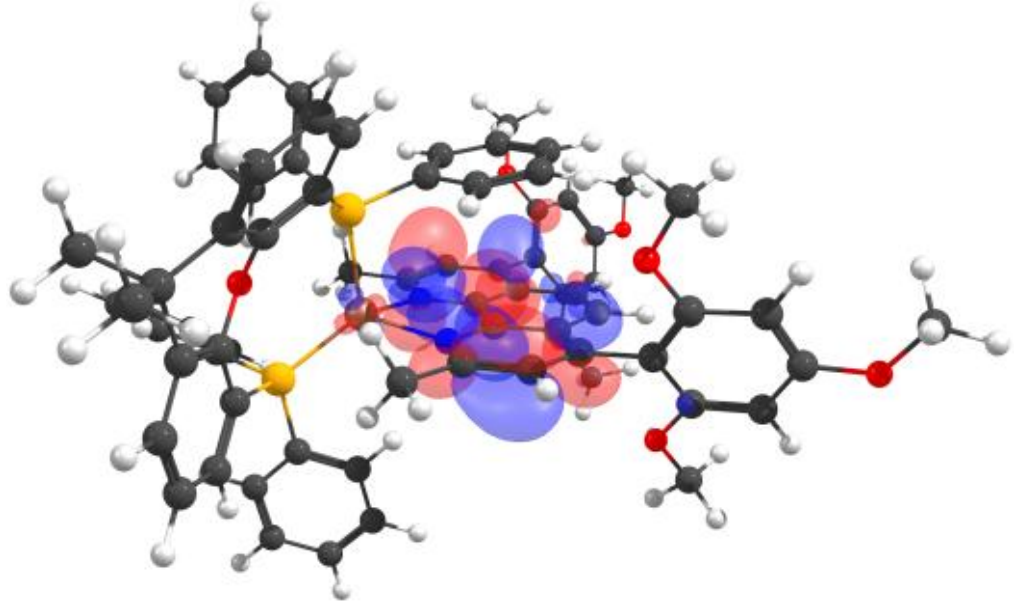	$IL_{py\rightarrow phen}CT$
S_6	353	3.513	0.0141	70	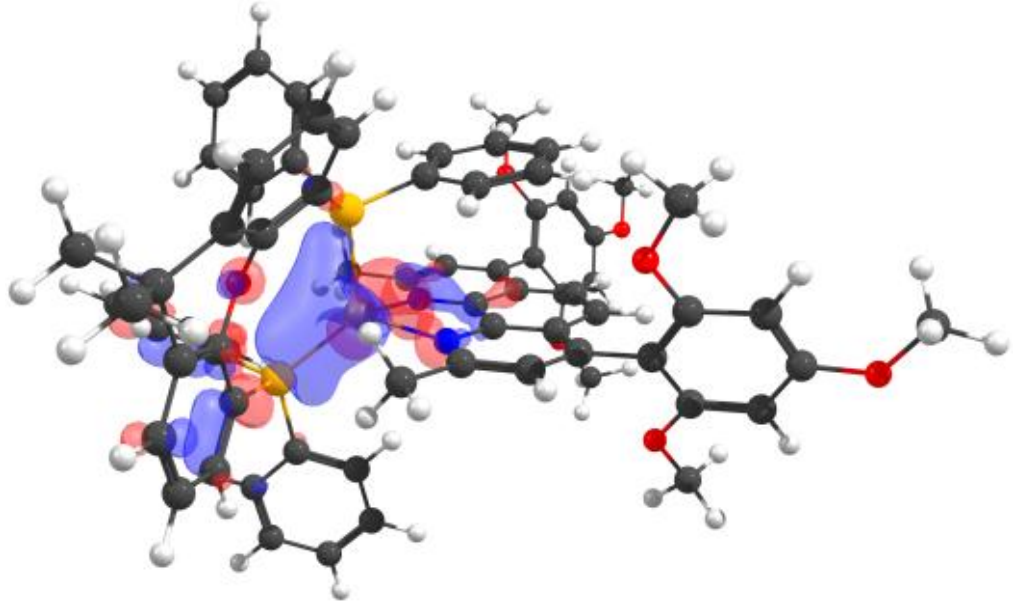	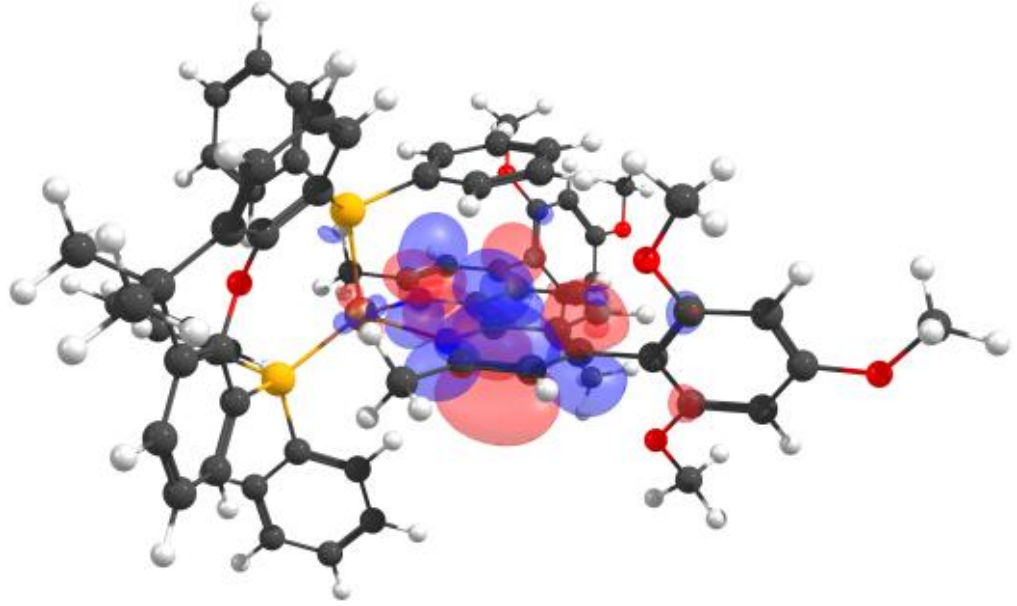	$ML_{phen}CT$
S_7	352	3.527	0.0293	75	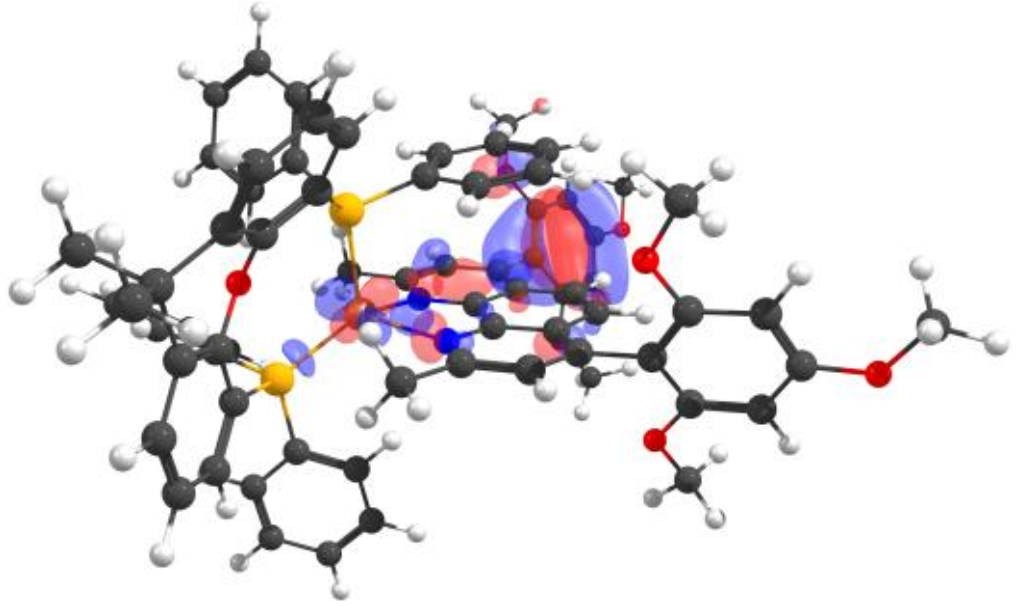	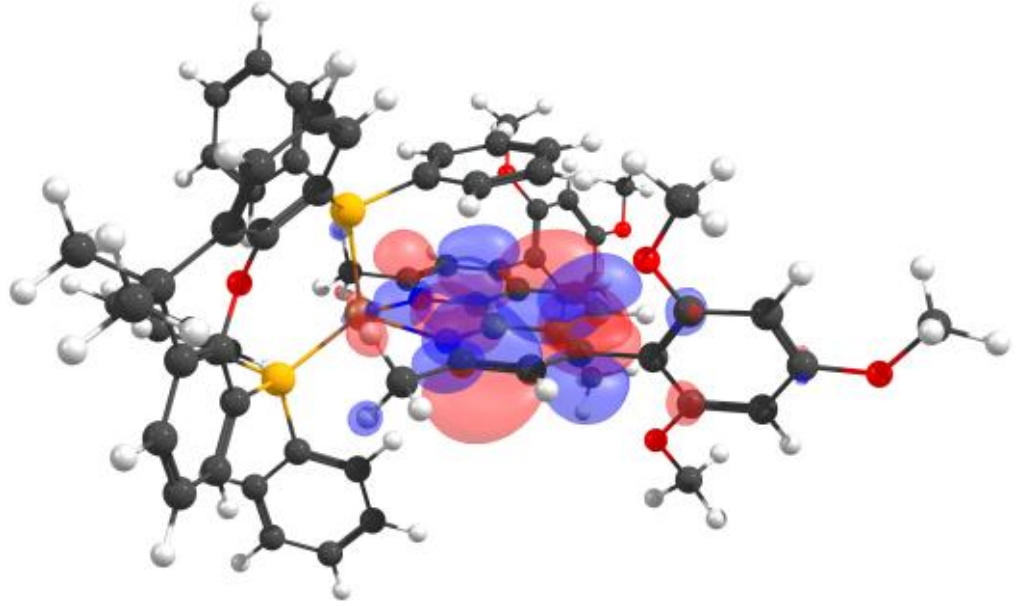	$IL_{py\rightarrow phen}CT$

state	λ / nm	ΔE / eV	f	cont. / %	initial state	final state	character
S_8	344	3.602	0.0049	97	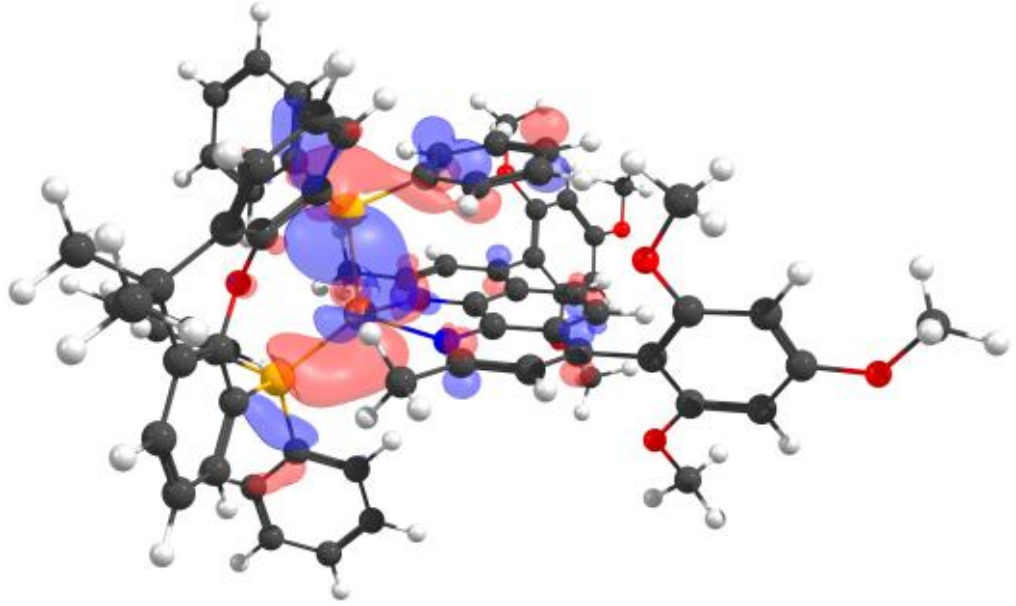	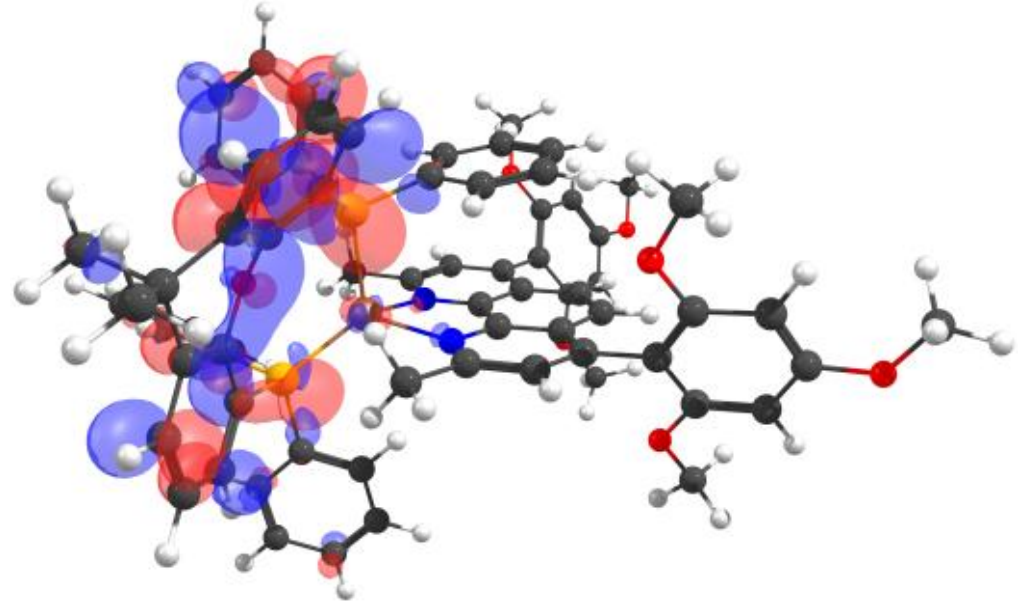	$ML_{xant}CT$
S_9	343	3.618	0.0078	95	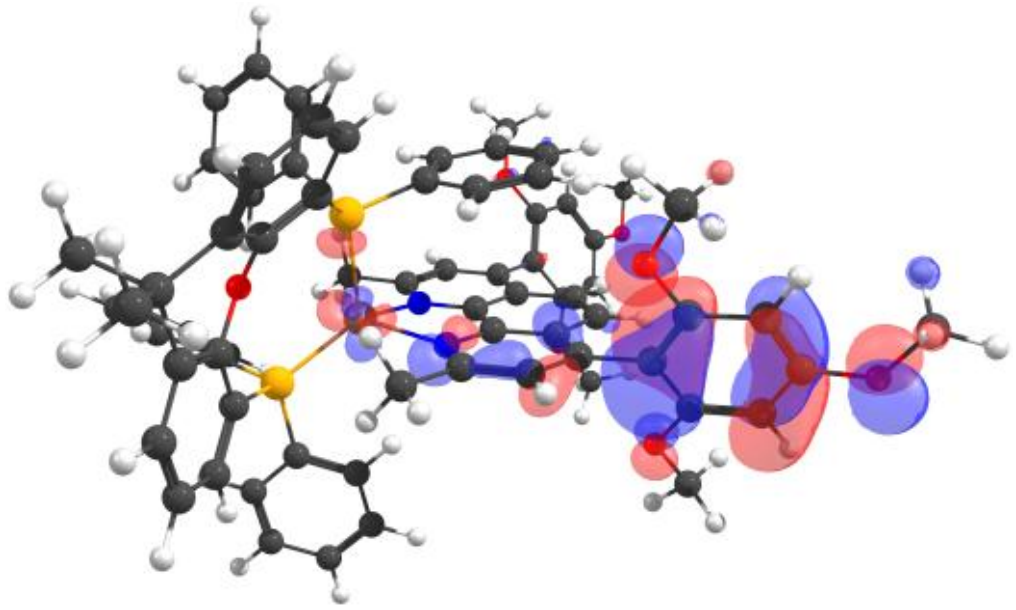	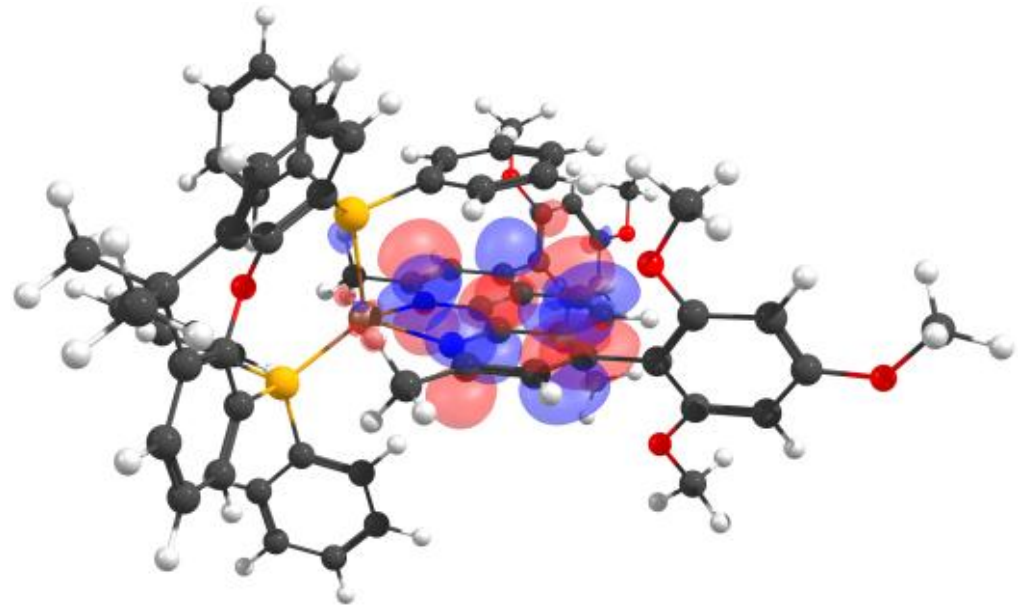	$IL_{py\rightarrow phen}CT$
S_{10}	340	3.642	0.0011	95	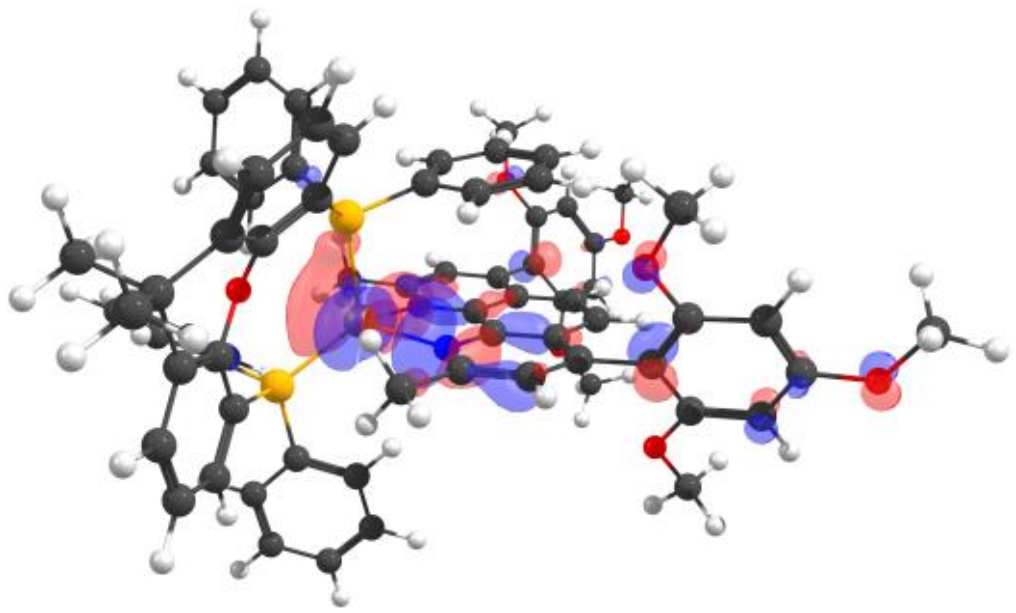	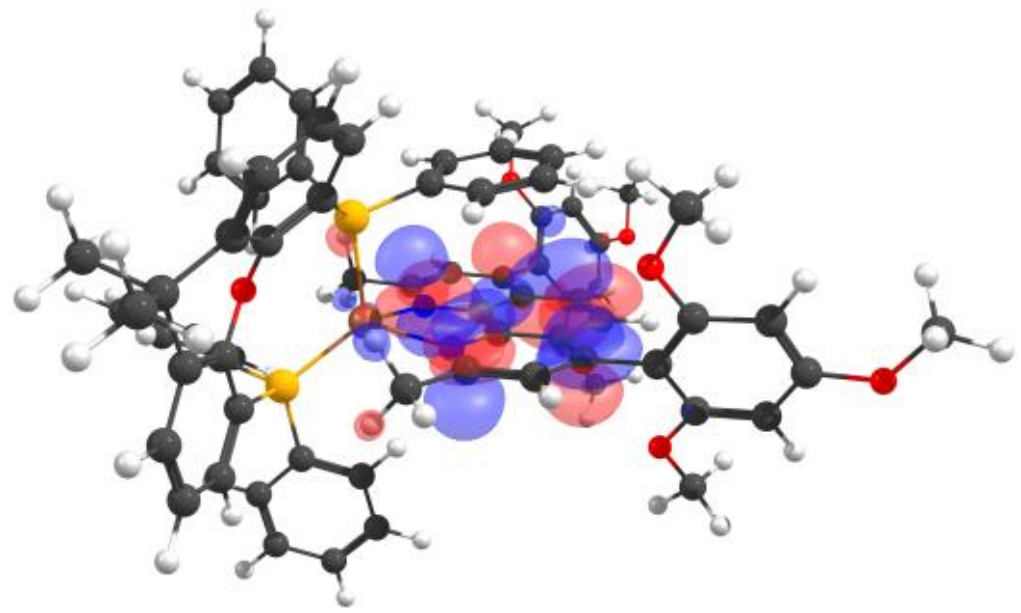	$ML_{phen}CT$

Table S5.5 Excitation energies (Δ*E* in eV), wavelengths (*λ* in nm) and oscillator strengths (*f*) of the first ten singlet-singlet vertical excitations of **C345**. The transitions are visualized in as natural transition orbitals (NTOs) of the initial and final state with the highest contribution (cont. in %) of each transition is displayed.

state	*λ* / nm	Δ*E* / eV	*f*	cont. / %	initial state	final state	character
S_1	438	2.830	0.1111	99	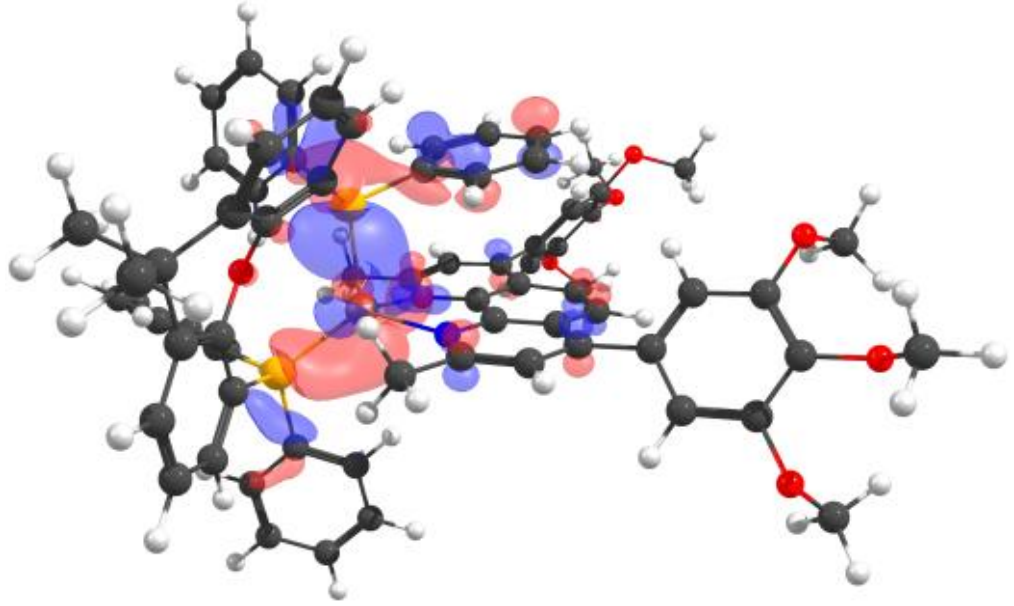	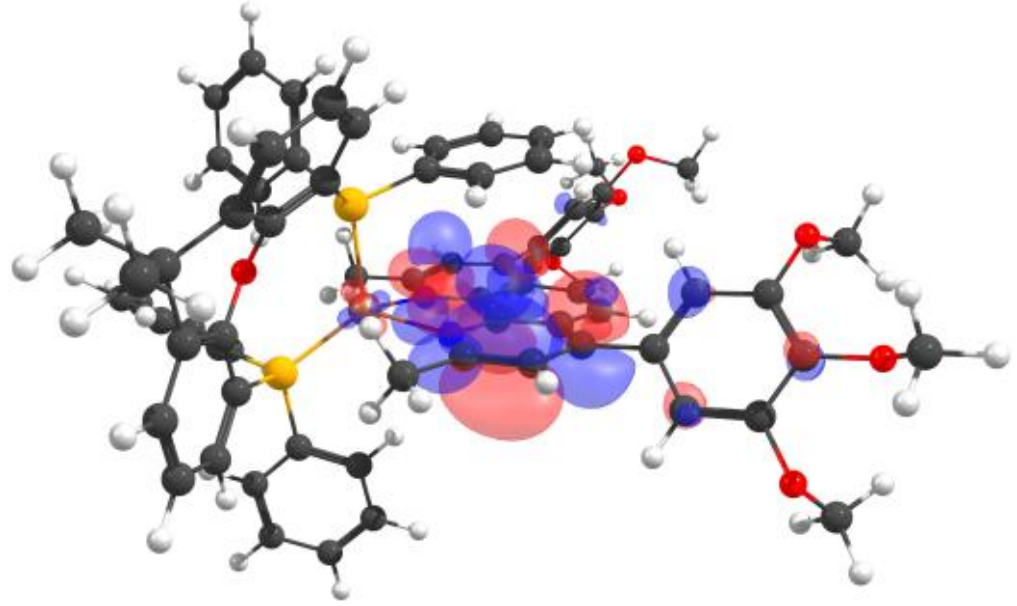	$ML_{phen}CT$
S_2	405	3.058	0.0560	98	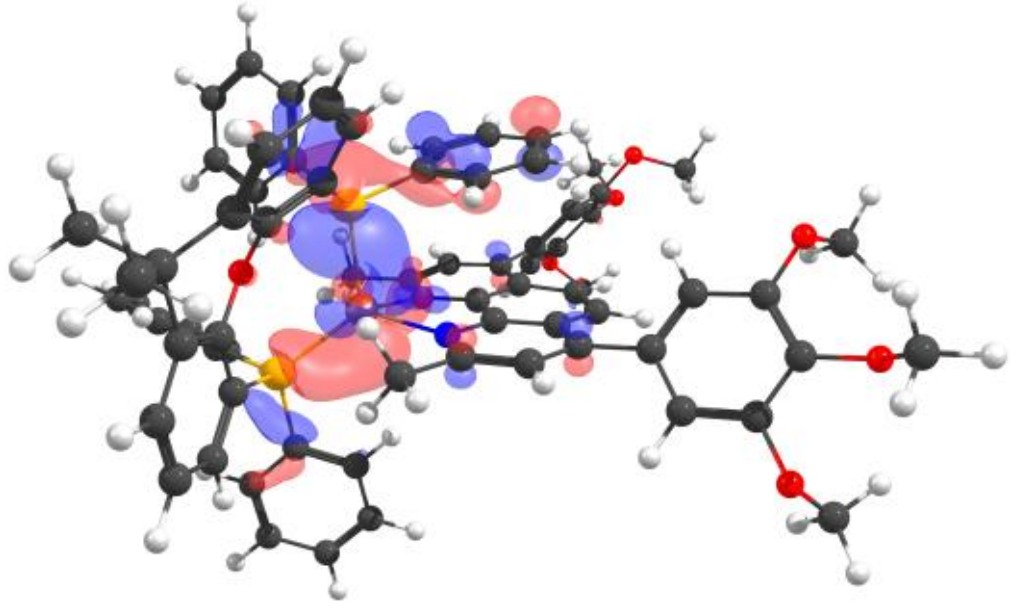	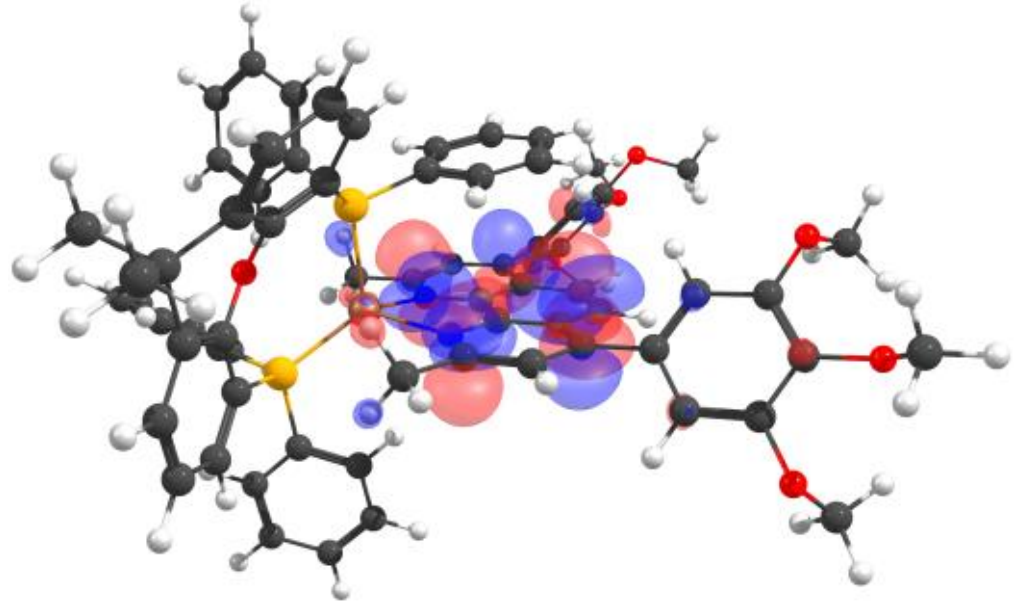	$ML_{phen}CT$
S_3	392	3.159	0.0087	99	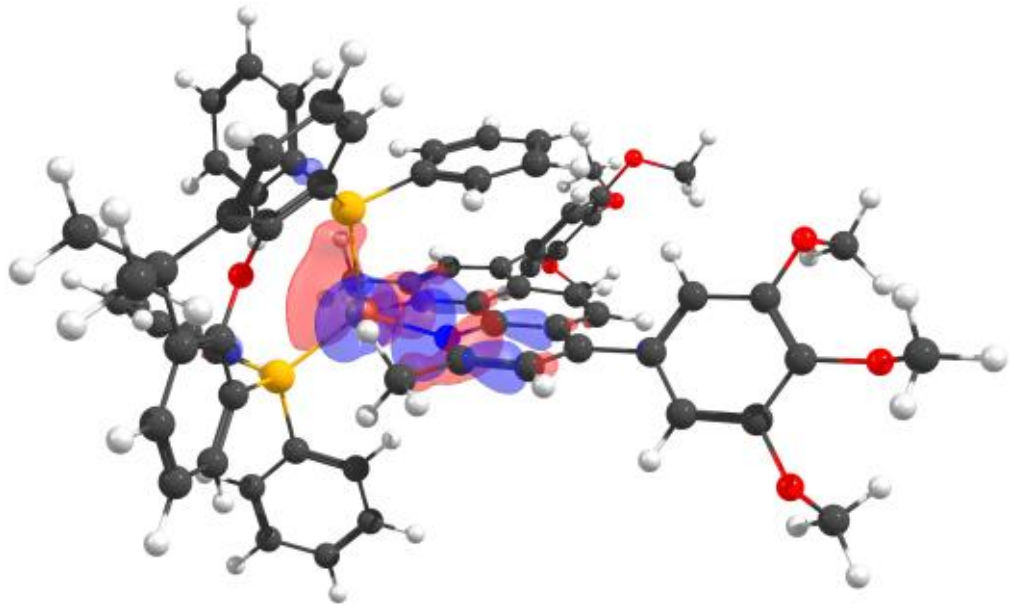	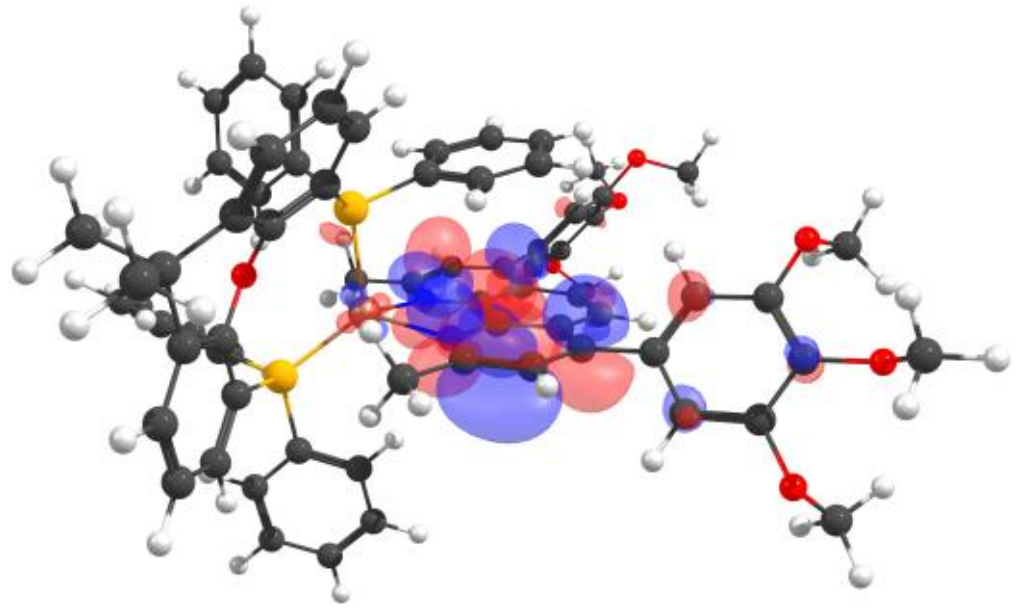	$ML_{phen}CT$

state	λ / nm	ΔE / eV	f	cont. / %	initial state	final state	character
S_4	370	3.350	0.0106	99	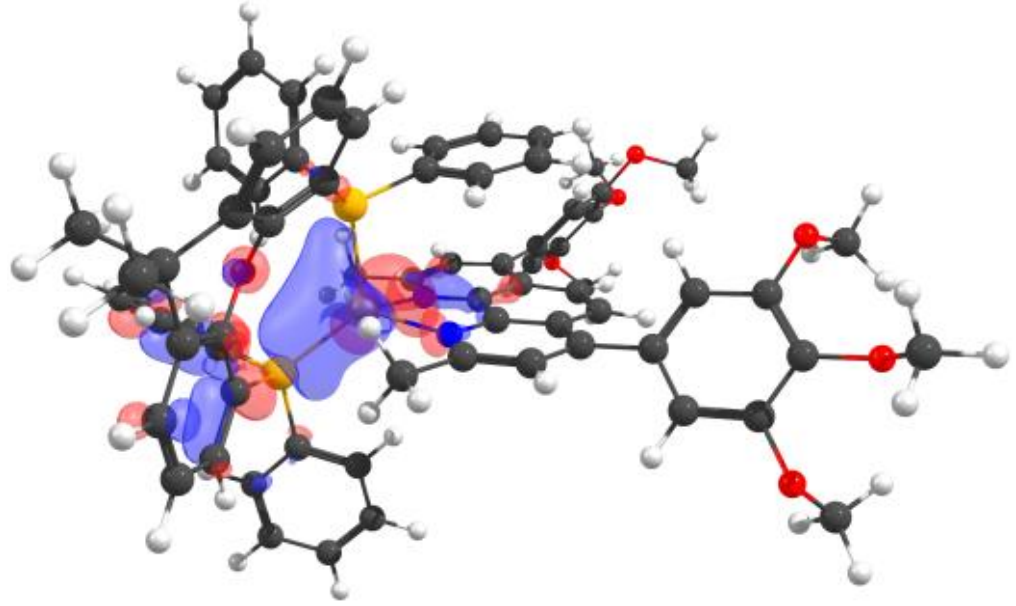	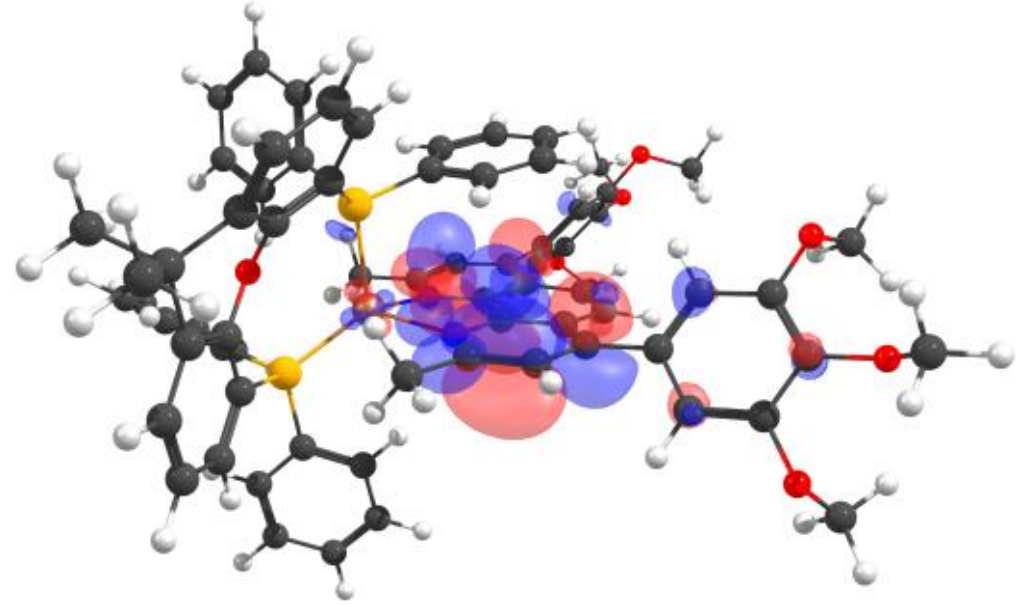	$ML_{phen}CT$
S_5	355	3.494	0.0150	98	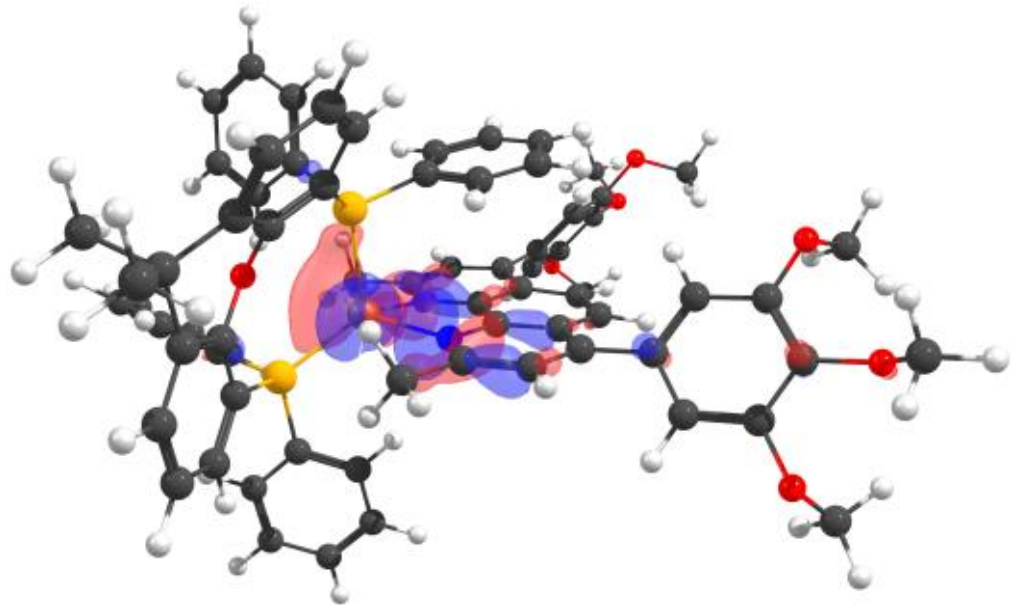	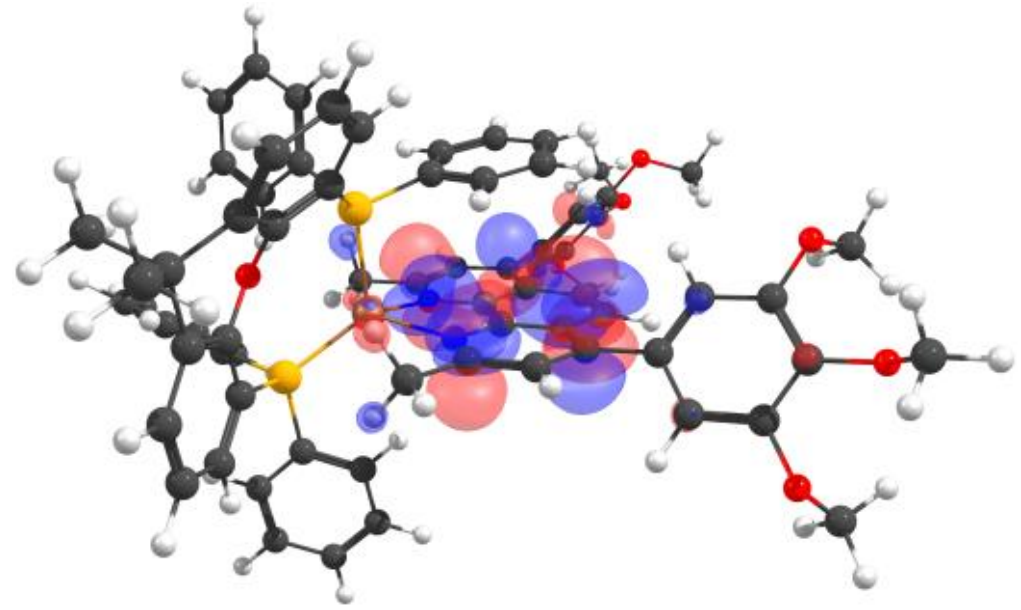	$ML_{phen}CT$
S_6	348	3.561	0.0522	96	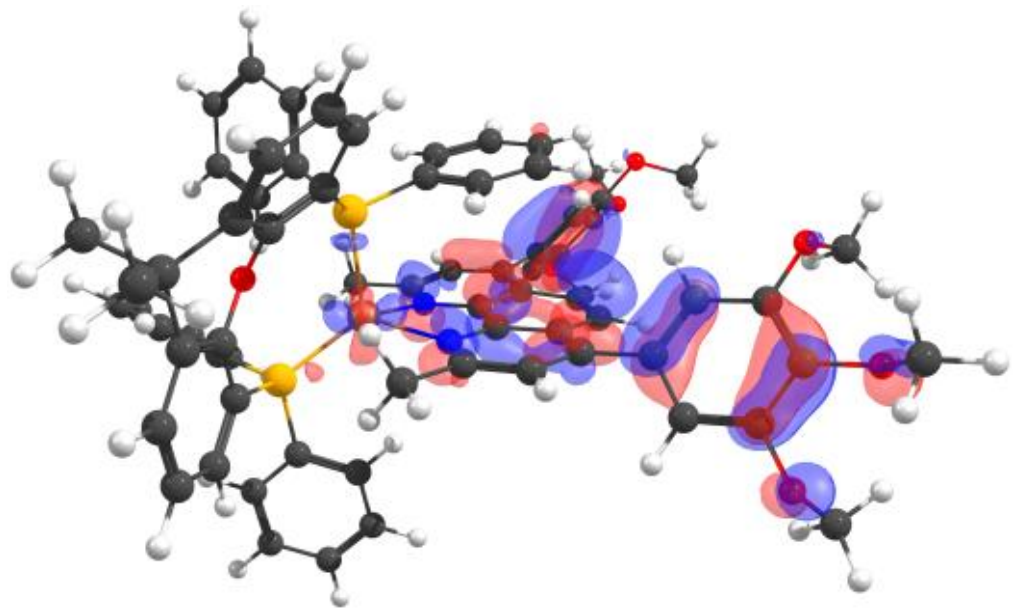	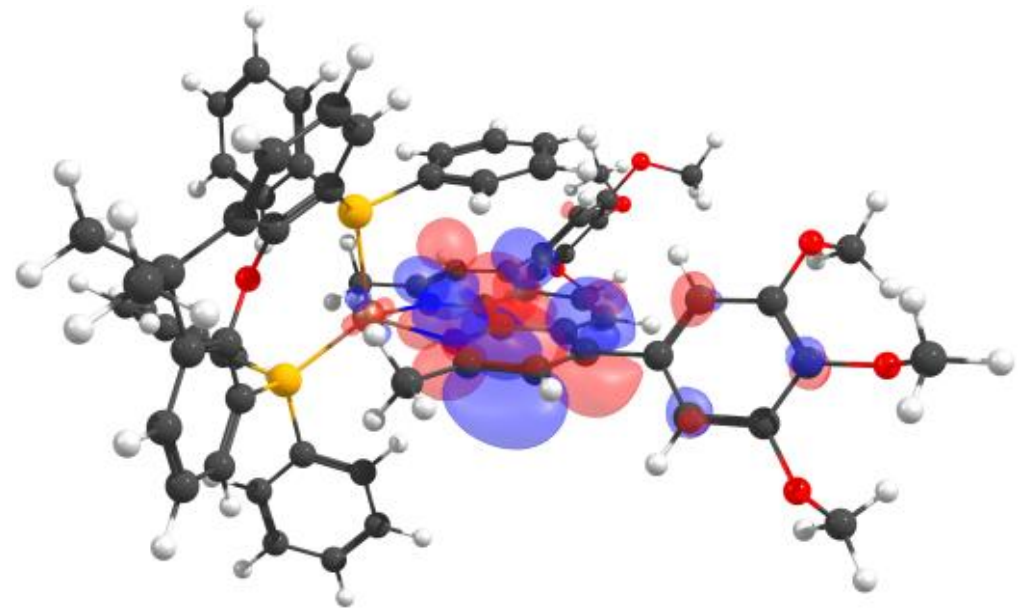	$IL_{py\rightarrow phen}CT$

state	λ / nm	ΔE / eV	f	cont. / %	initial state	final state	character
S_7	340	3.649	0.0100	94	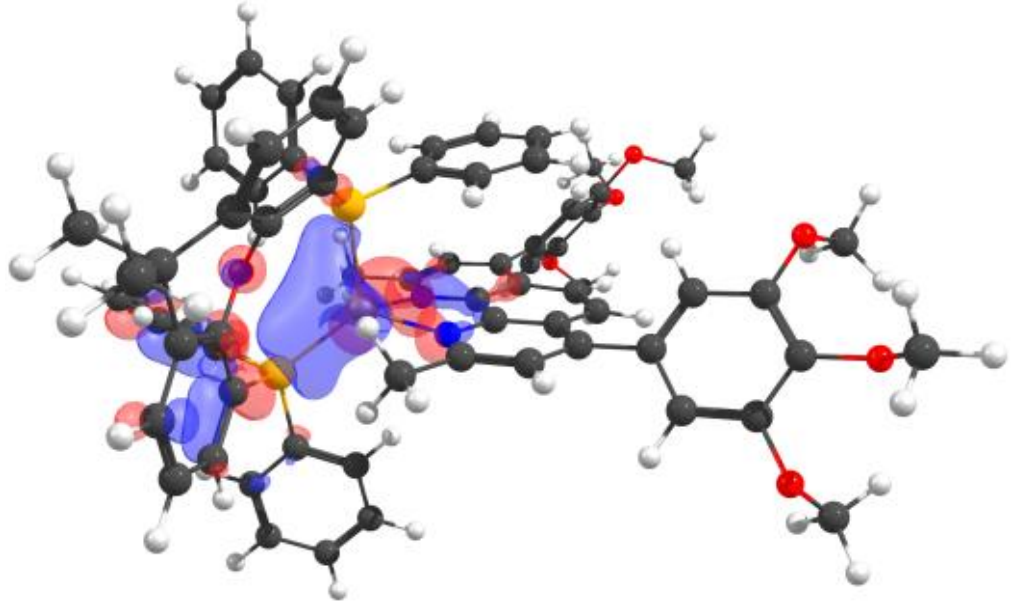	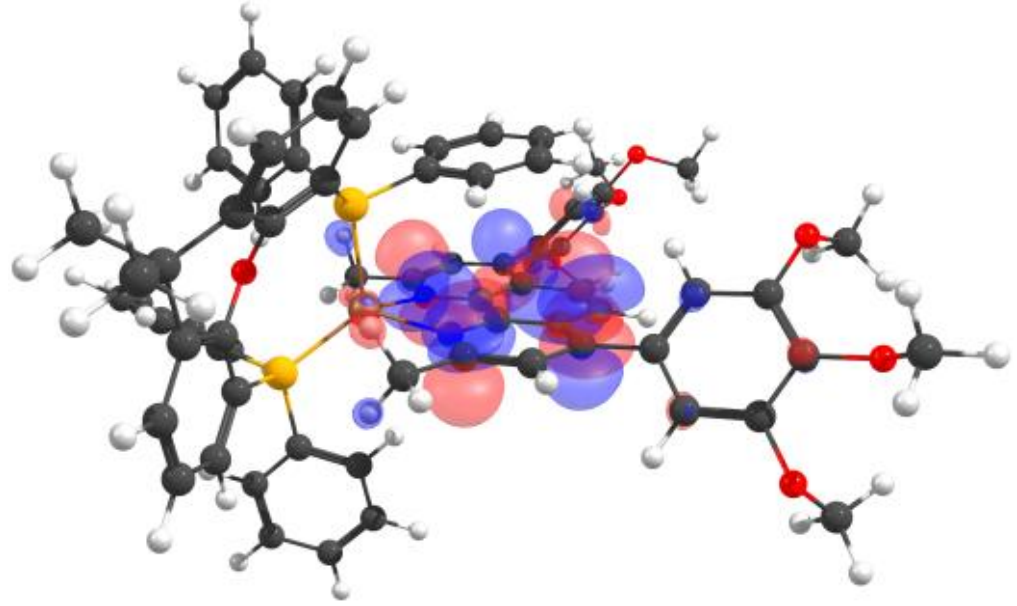	ML$_{\mathrm{phen}}$CT
S_8	338	3.666	0.1223	70	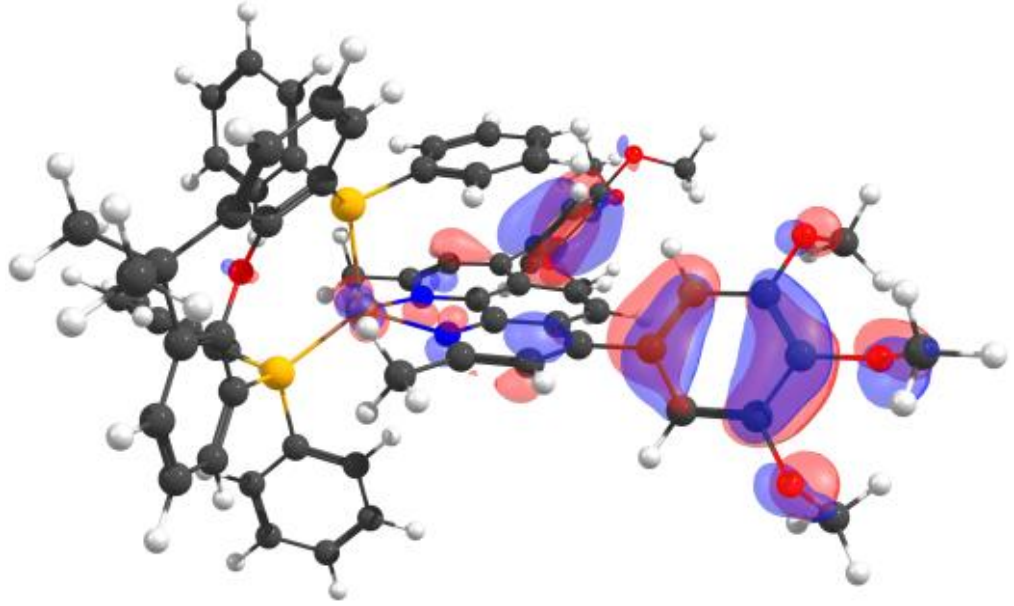	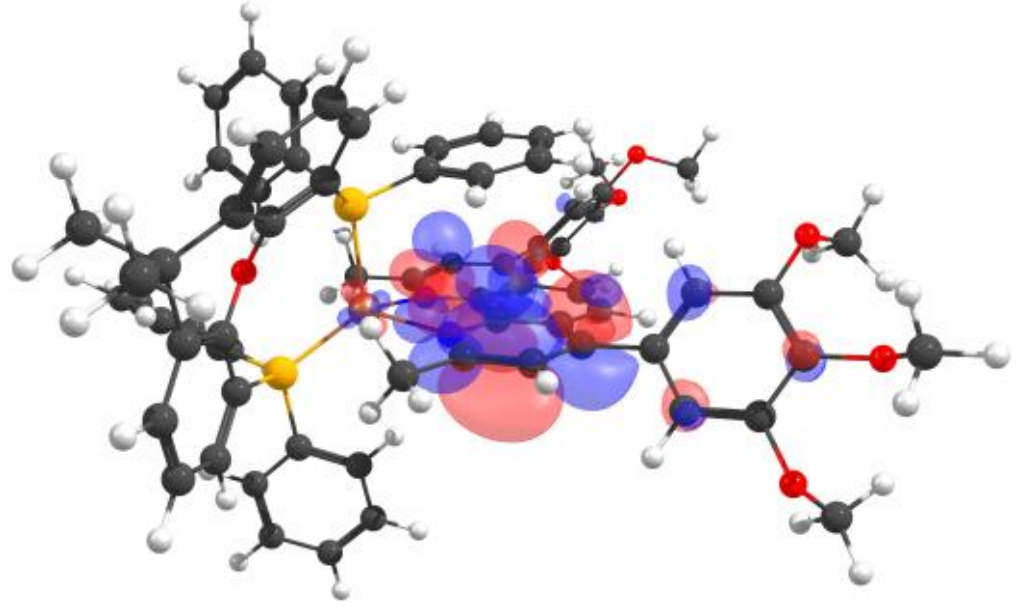	IL$_{\mathrm{py}\rightarrow\mathrm{phen}}$CT
S_9	337	3.680	0.0062	96	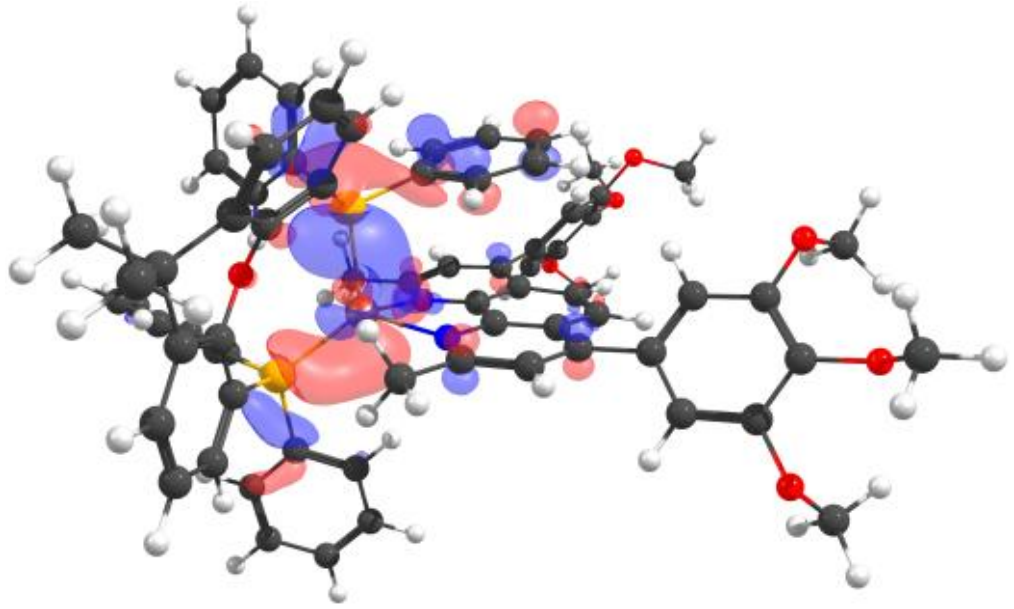	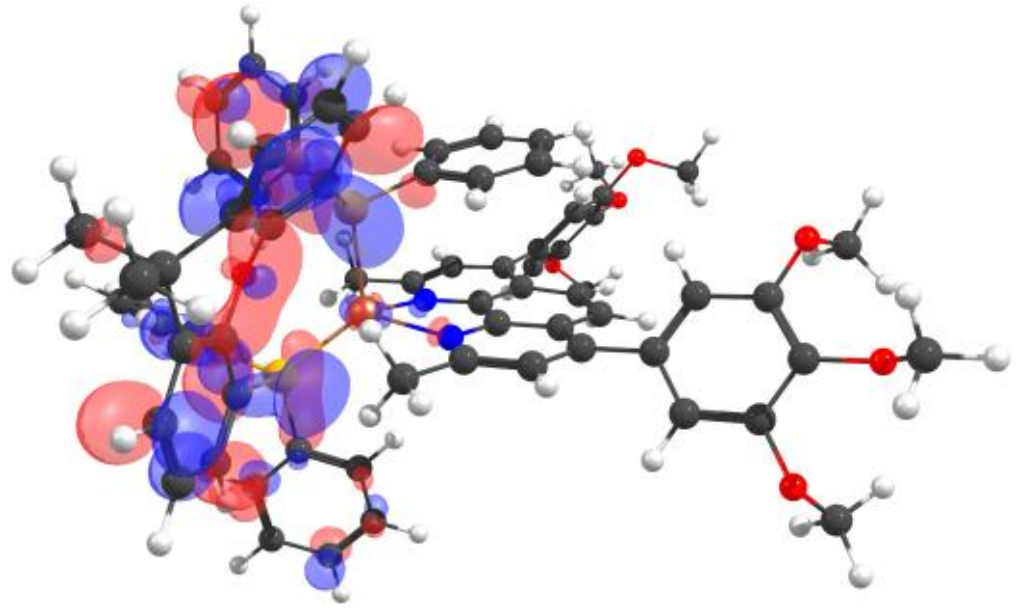	ML$_{\mathrm{xant}}$CT

state	λ / nm	ΔE / eV	f	cont. / %	initial state	final state	character
S_{10}	333	3.728	0.0033	90	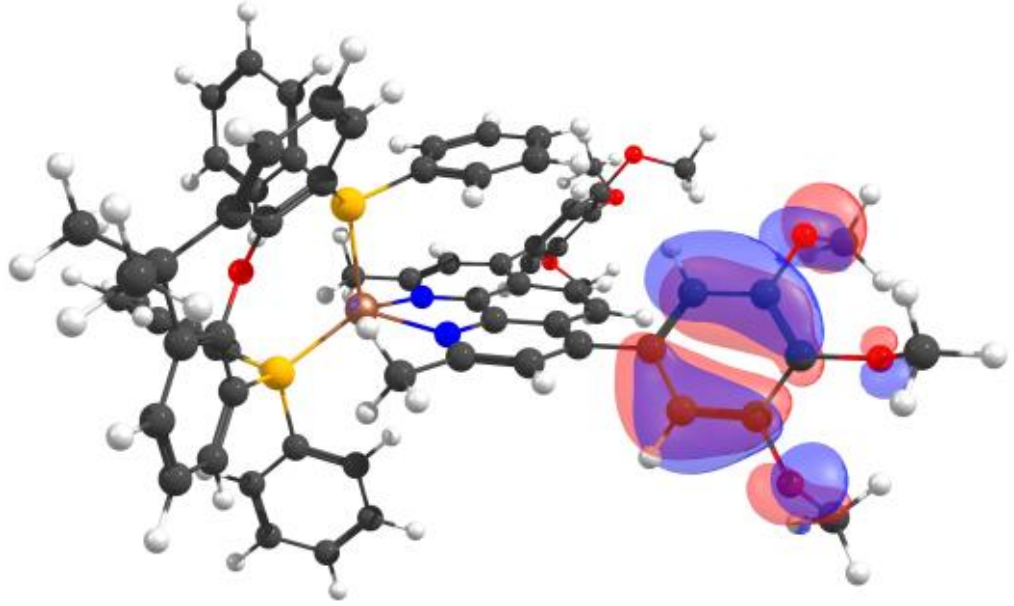	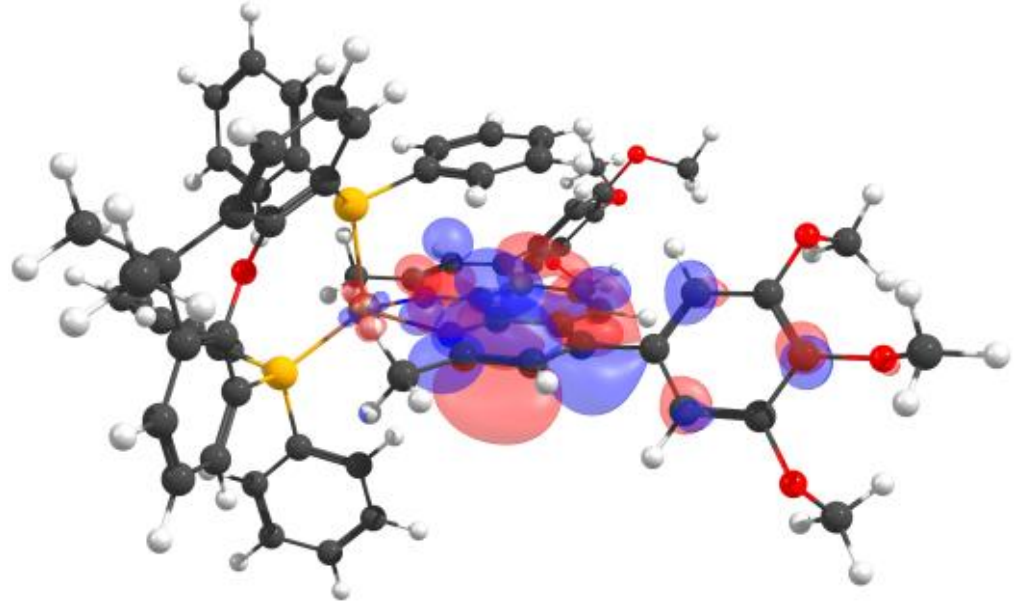	$IL_{py\rightarrow phen}CT$

6 Steady-State UV/vis Absorption and Emission Spectroscopy

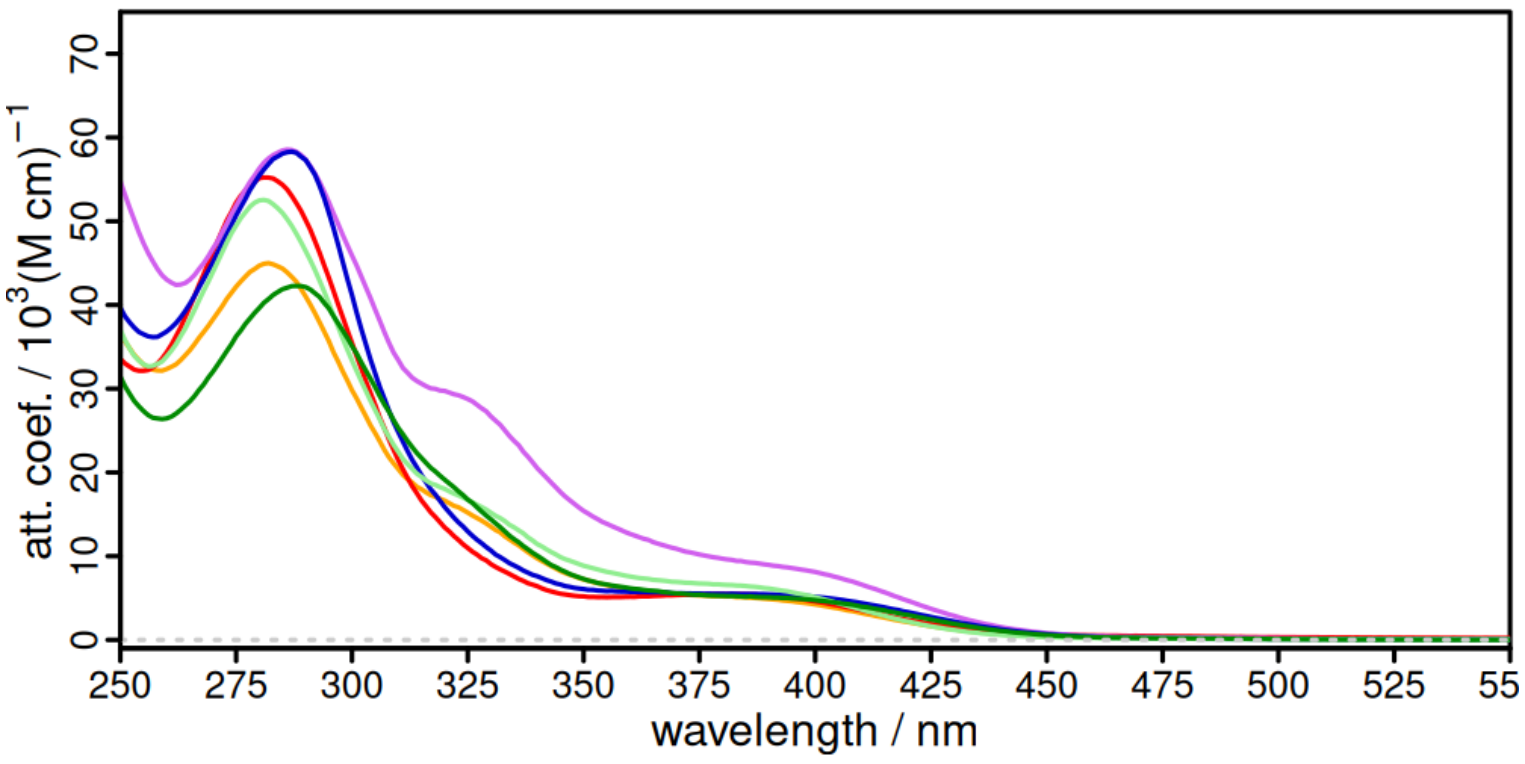

Figure S6.1. UV/vis absorption spectra of **Cu24** (orange), **Cu26** (red), **Cu34** (purple), **Cu35** (blue), **Cu246** (light green), and **Cu345** (dark green) in acetonitrile solution at room temperature.

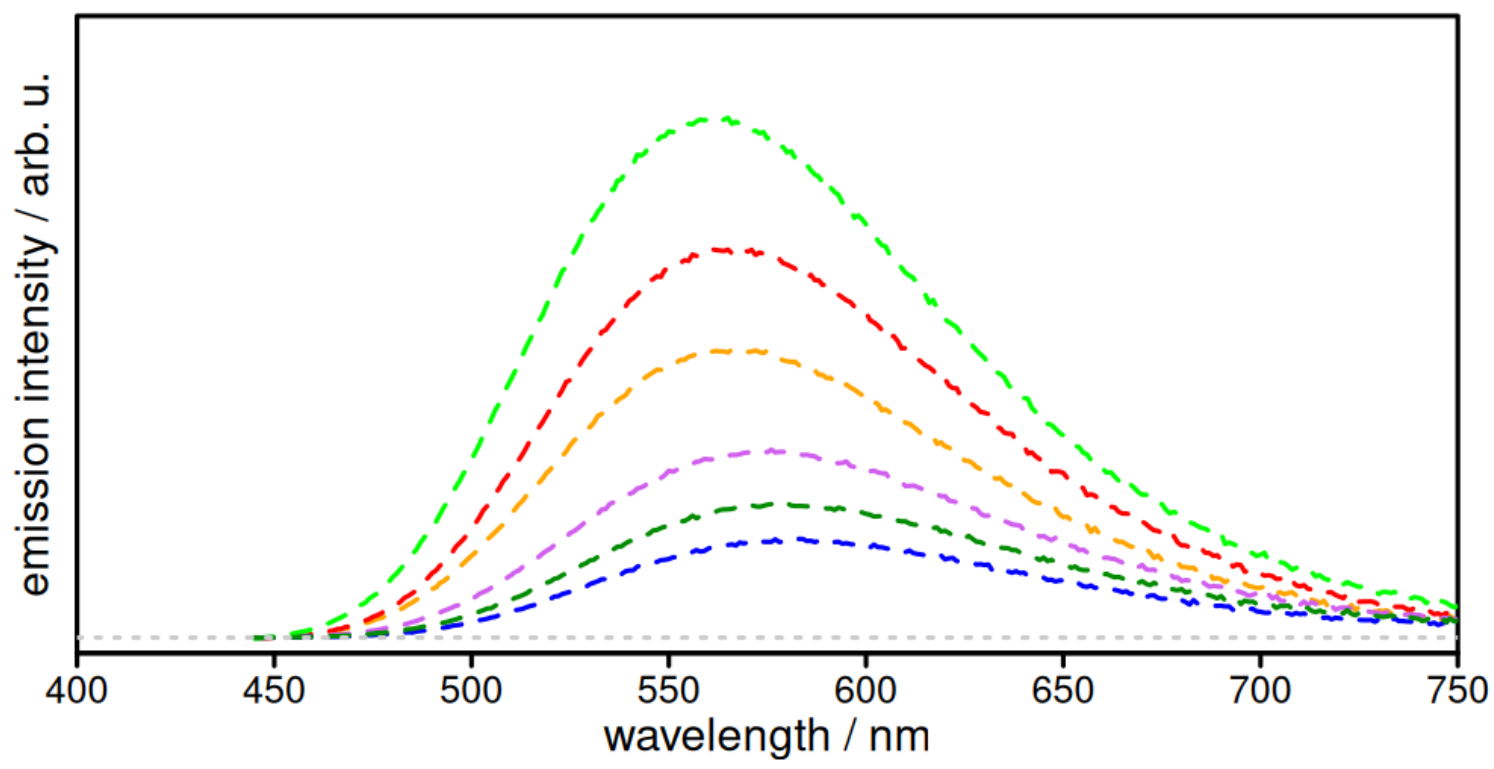

Figure S6.2. Emission spectra of **Cu24** (orange), **Cu26** (red), **Cu34** (purple), **Cu35** (blue), **Cu246** (light green), and **Cu345** (dark green) in acetonitrile under inert conditions upon excitation at 385 nm.

7 Time-Resolved Emission Spectroscopy

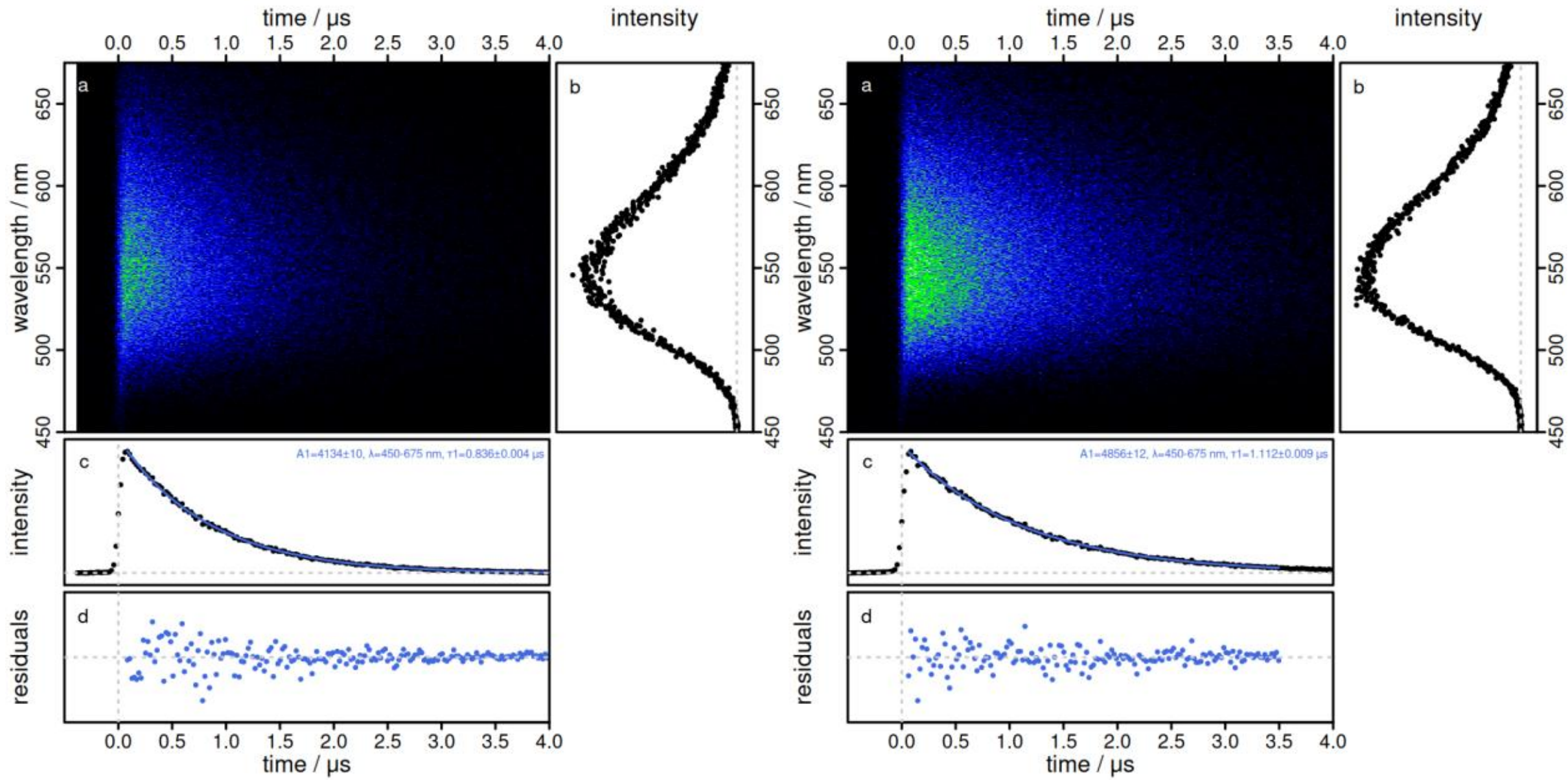

Figure S7.1. Streak-camera measurements of **Cu24** (left) and **Cu26** (right) in acetonitrile under inert conditions upon excitation at 385 nm. The plots display (a) the streak images, (b) the emission spectra, (c) the kinetic traces with the fit curve, and (d) the residuals of the decay fits.

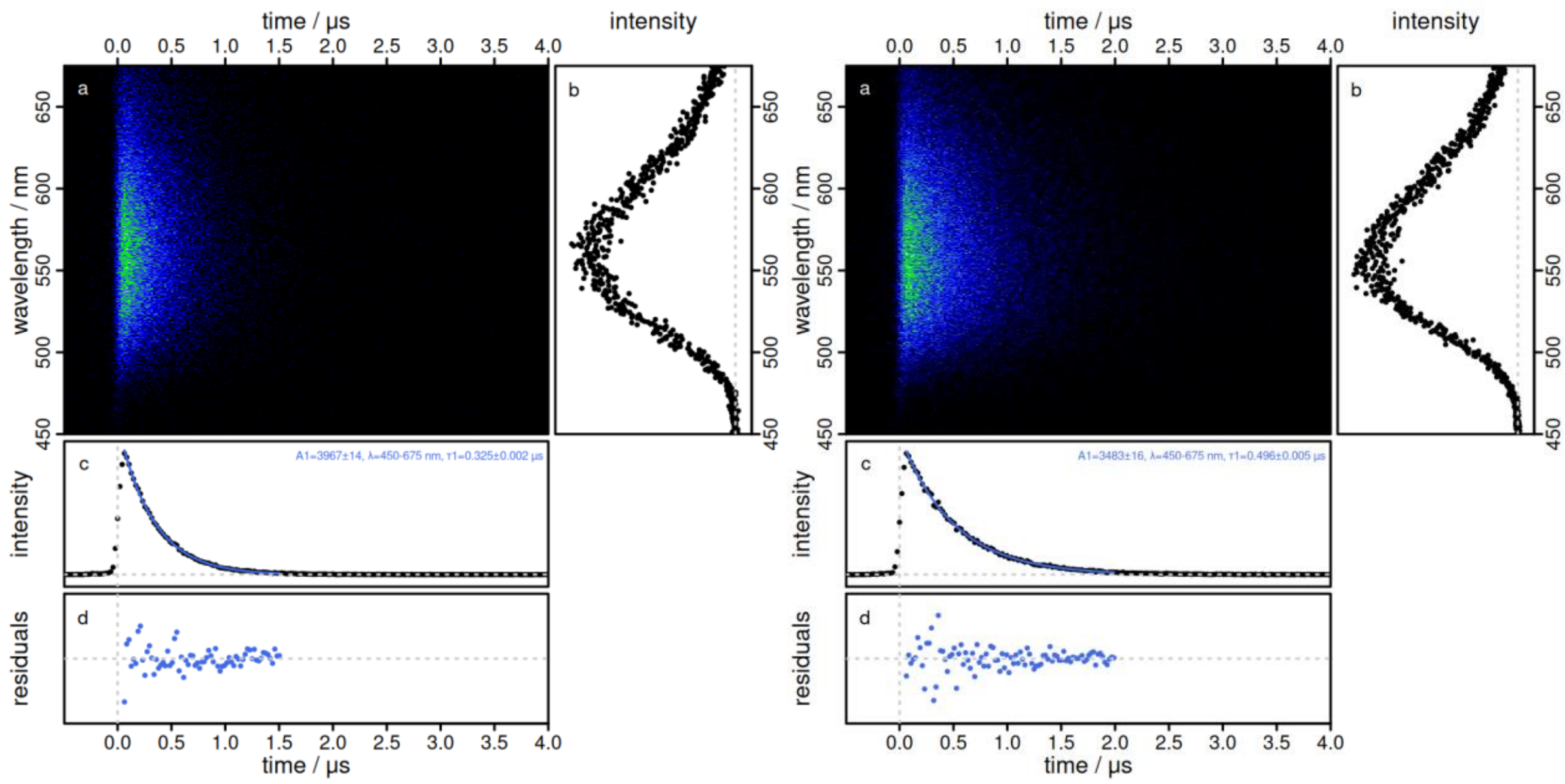

Figure S7.2. Streak-camera measurements of **Cu35** (left) and **Cu345** (right) in acetonitrile under inert conditions upon excitation at 385 nm. The plots display (a) the streak images, (b) the emission spectra, (c) the kinetic traces with the fit curve, and (d) the residuals of the decay fits.

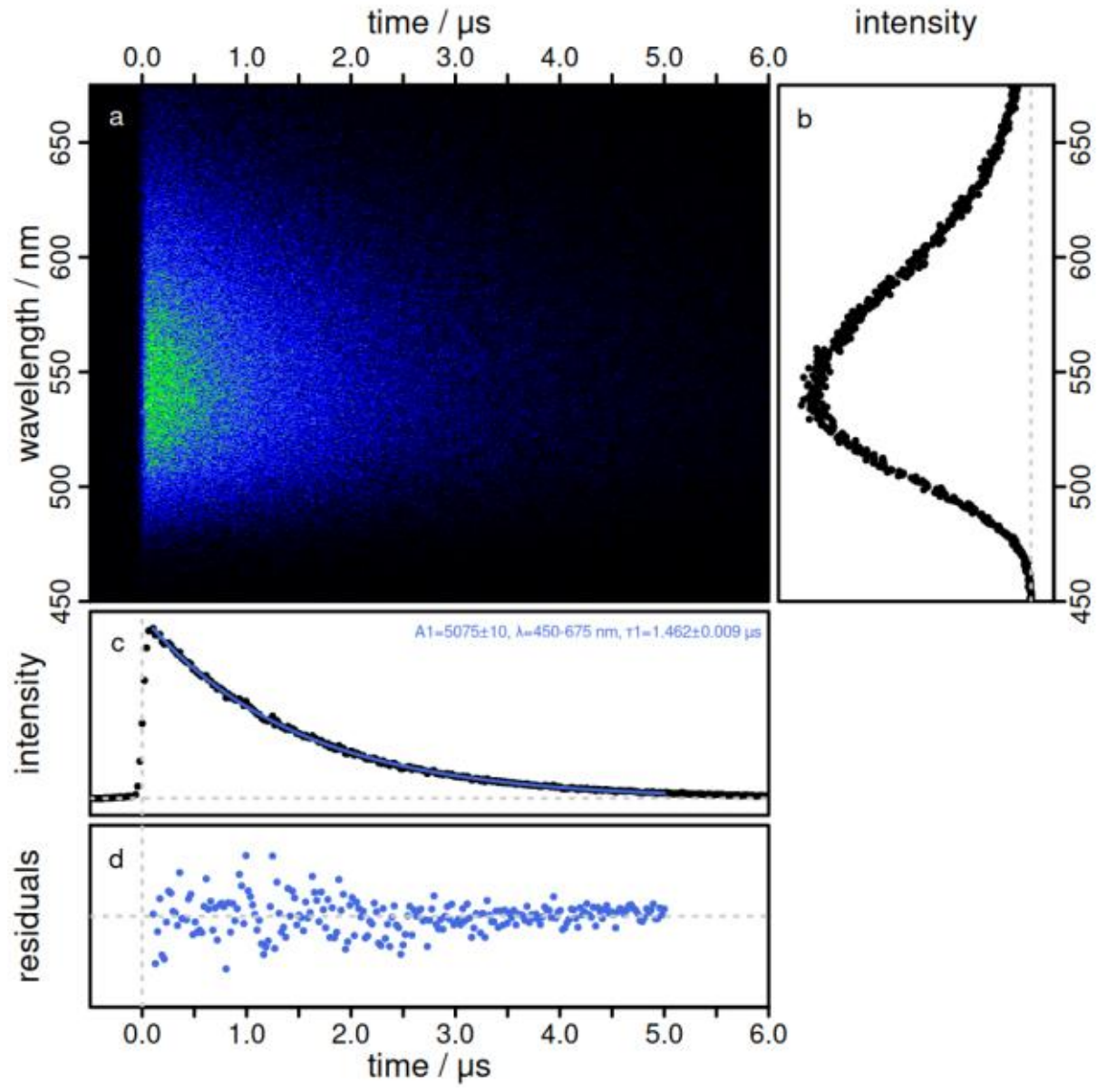

Figure S7.3. Streak-camera measurements of **Cu246** in acetonitrile under inert conditions upon excitation at 385 nm. The plots display (a) the streak image, (b) the emission spectrum, (c) the kinetic traces with the fit curve, and (d) the residuals of the decay fits.

Table S7.1. Emission lifetimes of the investigated Cu(I) complexes in acetonitrile under inert conditions.

Complex	**Cu24**	**Cu26**	**Cu35**	**Cu246**	**Cu345**
Lifetime $\tau_{\mathbf{em}}$	836 ± 4 ns	1112 ± 9 ns	325 ± 2 ns	1462 ± 9 ns	496 ± 5 ns

8 Electrochemical Data

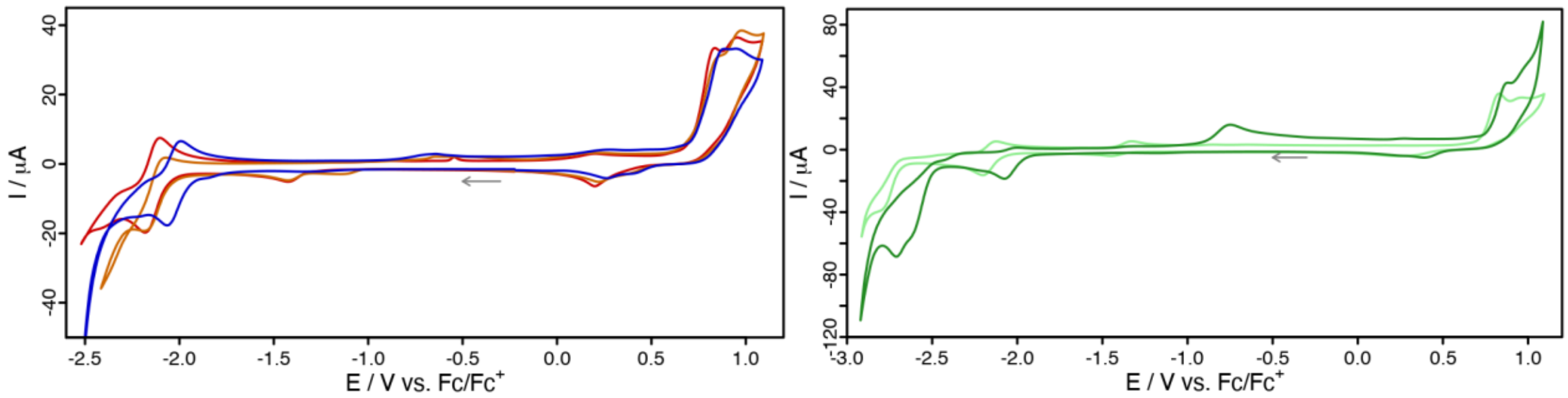

Figure S8.1. Cyclic voltammograms of **Cu24** (orange, left), **Cu26** (red, left), **Cu35** (blue, left), **Cu246** (light green, right), and **Cu345** (dark green, right) in deaerated acetonitrile solution (c = 1 mM) with $[Bu_4N][PF_6]$ as the supporting electrolyte and referenced *vs.* the ferrocene/ferrocenium (Fc/Fc^+) couple. The initial scan direction is indicated by the grey arrow.

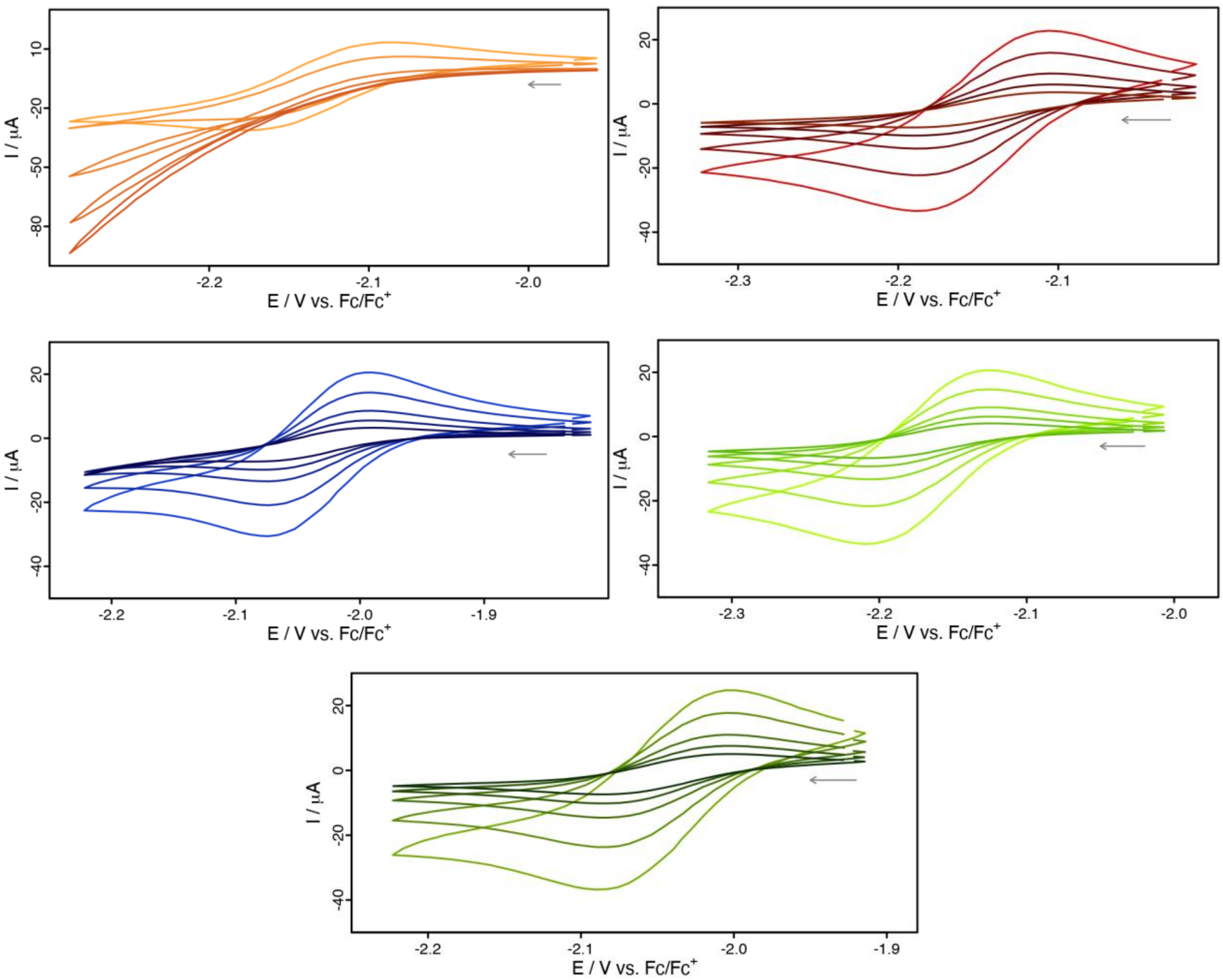

Figure S8.2. Excerpt of the first reversible reduction event of **Cu24** (orange, top left), **Cu26** (red, top right), **Cu35** (blue, middle left), **Cu246** (light green, middle right), and **Cu345** (dark green, bottom) recorded at different scan rates: 0.5 V/s, 0.25 V/s, 0.1 V/s, 0.05 V/s, 0.025 V/s (from dark to bright). The initial scan direction is indicated by the grey arrow.

9 Excited-State Reduction Potentials

The excited-state reduction potential was estimated via the simplified REHM–WELLER[13] equation:

$$E^{*}_{1/2}{}^{red} = E_{1/2}{}^{red} + E_{0,0} \qquad \text{eq. 9.1}$$

For the determination of E_{00} the intercept wavelength was estimated from the emission spectrum in acetonitrile (Fig. S9.1) and converted into eV.

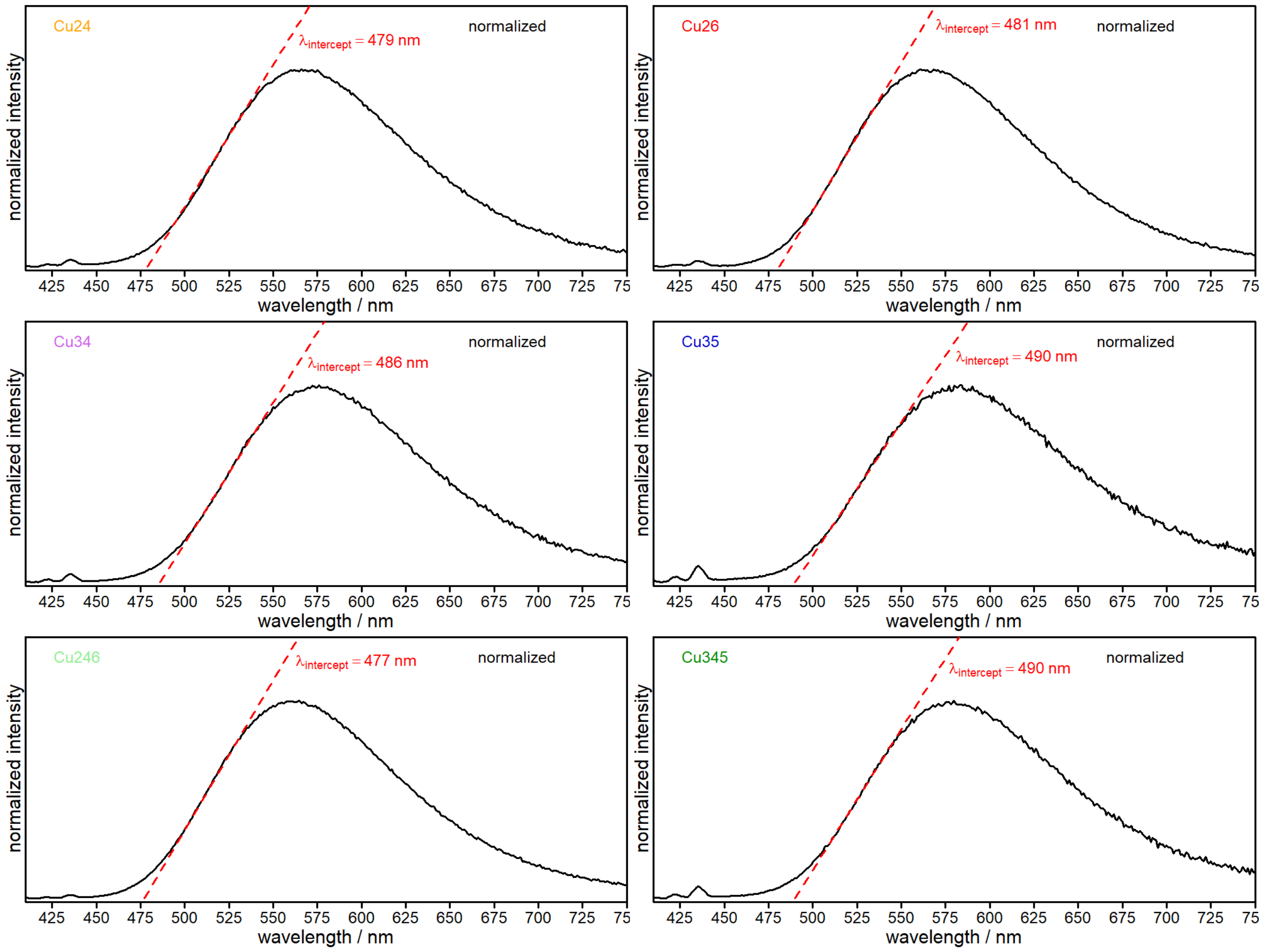

Figure S9.1. Determination of the intercept wavelength for **Cu24** (top left), **Cu26** (top right), **Cu34** (middle left), **Cu35** (middle right), **Cu246** (bottom left), and **Cu345** (bottom right).

Table S9.1. Determined and calculated values used for the estimation of $E^{*}_{1/2}{}^{red}$.

**Complex**	$E_{1/2}$red [V]	E_{00} [nm]	E_{00} [eV]	$E^{*}_{1/2}{}^{red}$ [V *vs*. Fc/Fc^{+}]
**Cu24**	-2.13	479	2.59	0.46
**Cu26**	-2.15	481	2.58	0.43
**Cu34**	-2.07[a]	486	2.55	0.48
**Cu35**	-2.03	490	2.53	0.50
**Cu246**	-2.18	477	2.60	0.42
**Cu345**	-2.05	490	2.53	0.48

[a] Taken from reference 14.

10 Photocatalysis

Singlet Oxygen Formation

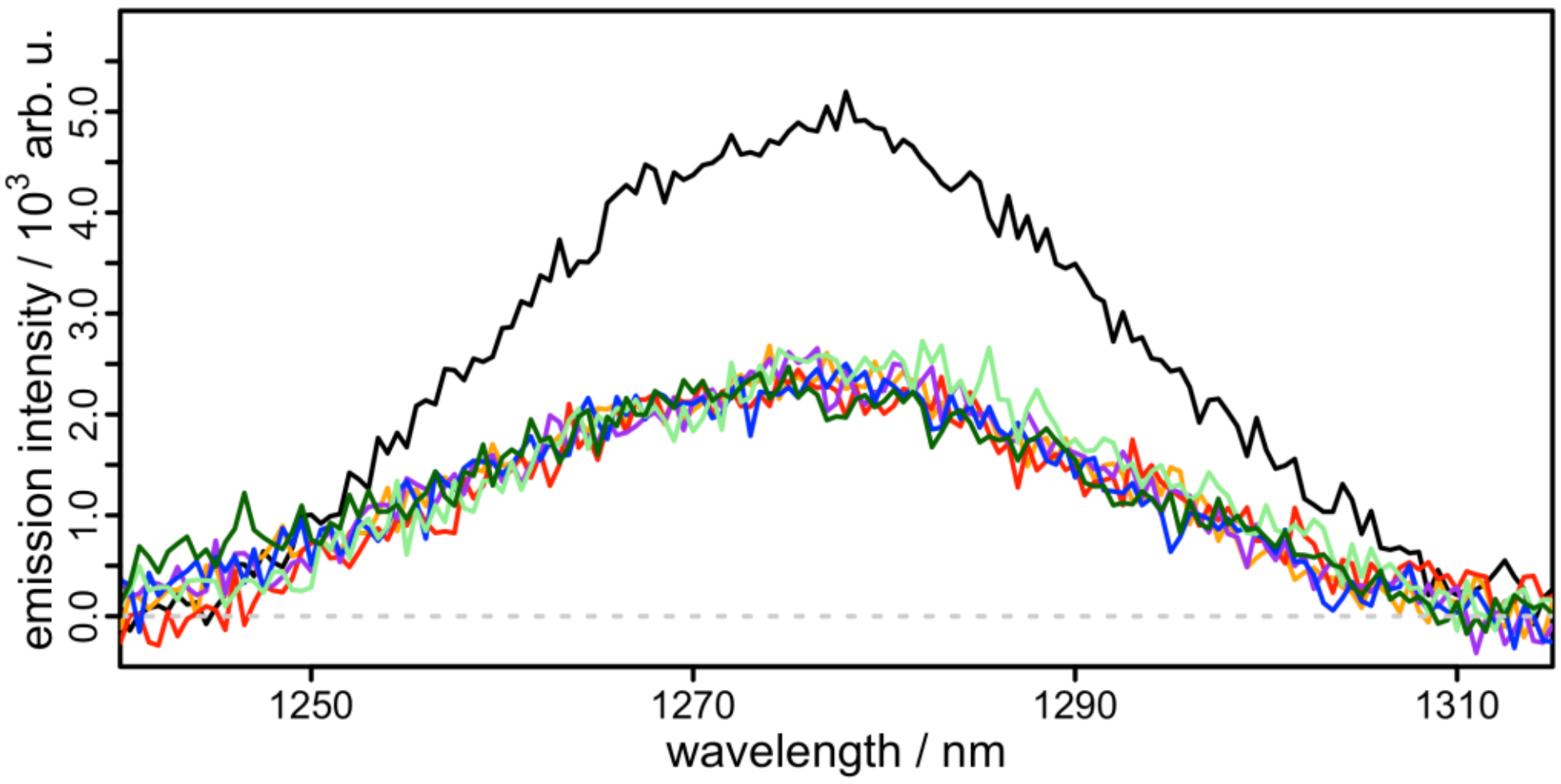

Figure S10.1. Near-infrared emission spectra arising from the decay of generated $^1O_2$ for **Cu24** (orange), **Cu26** (red), **Cu35** (blue), **Cu246** (light green), and **Cu345** (dark green), together with phenalenone (black) as the reference. The spectra are baseline corrected at 1315-1325 nm.

Photooxidation of 2,5-Diphenylfuran

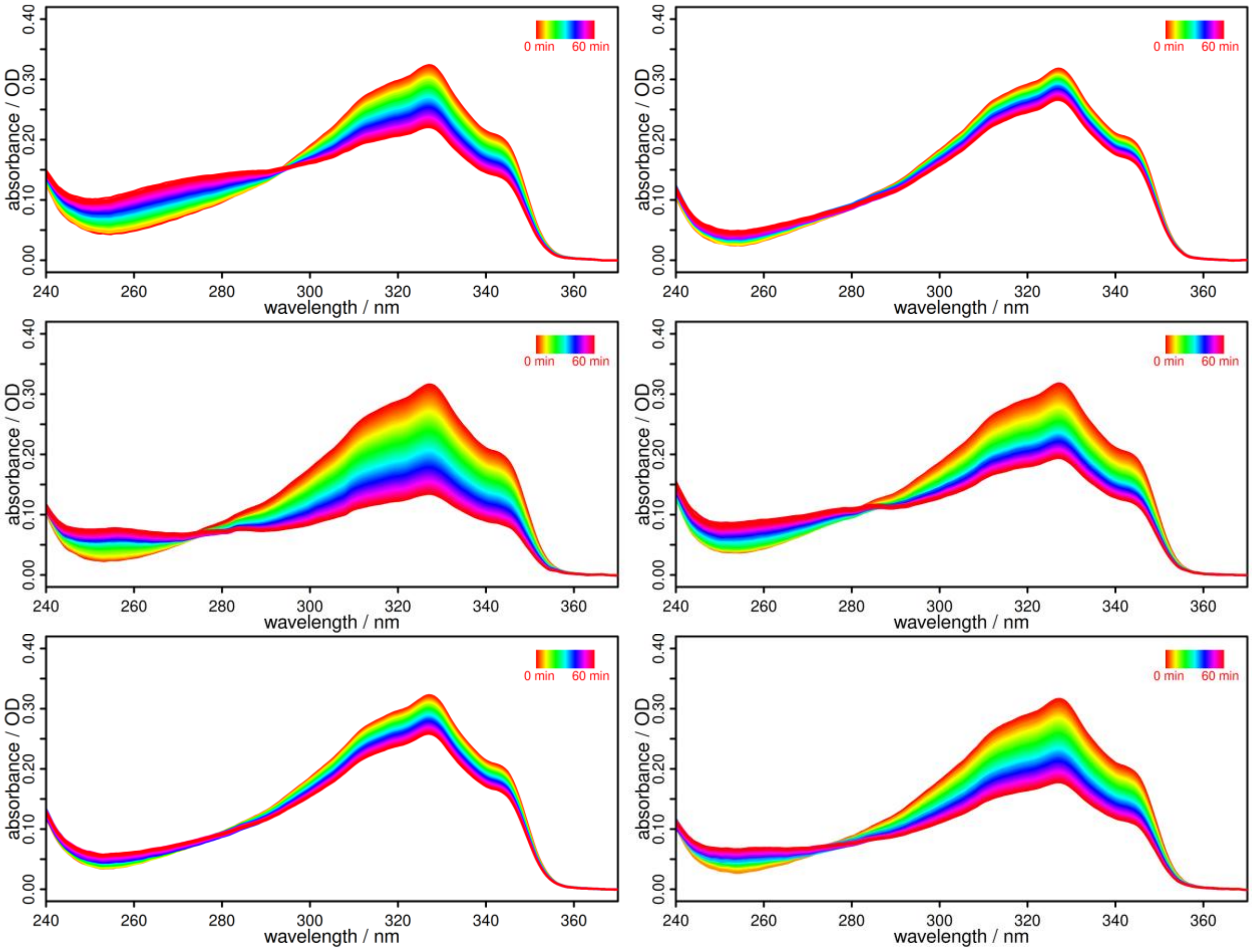

Figure S10.2. Summary of the *in-situ* UV/vis absorption spectral changes during the photocatalytic oxygenation of 2,5-diphenylfuran (DPF, c = $1.9 \cdot 10^{-5}$ M) in aerated dichloromethane under irradiation with a starlight lamp over 60 min for each complex (c = $0.95 \cdot 10^{-6}$ M): **Cu24** (top left), **Cu26** (top right), **Cu34** (middle left), **Cu35** (middle right), **Cu246** (bottom left) and **Cu345** (bottom right).

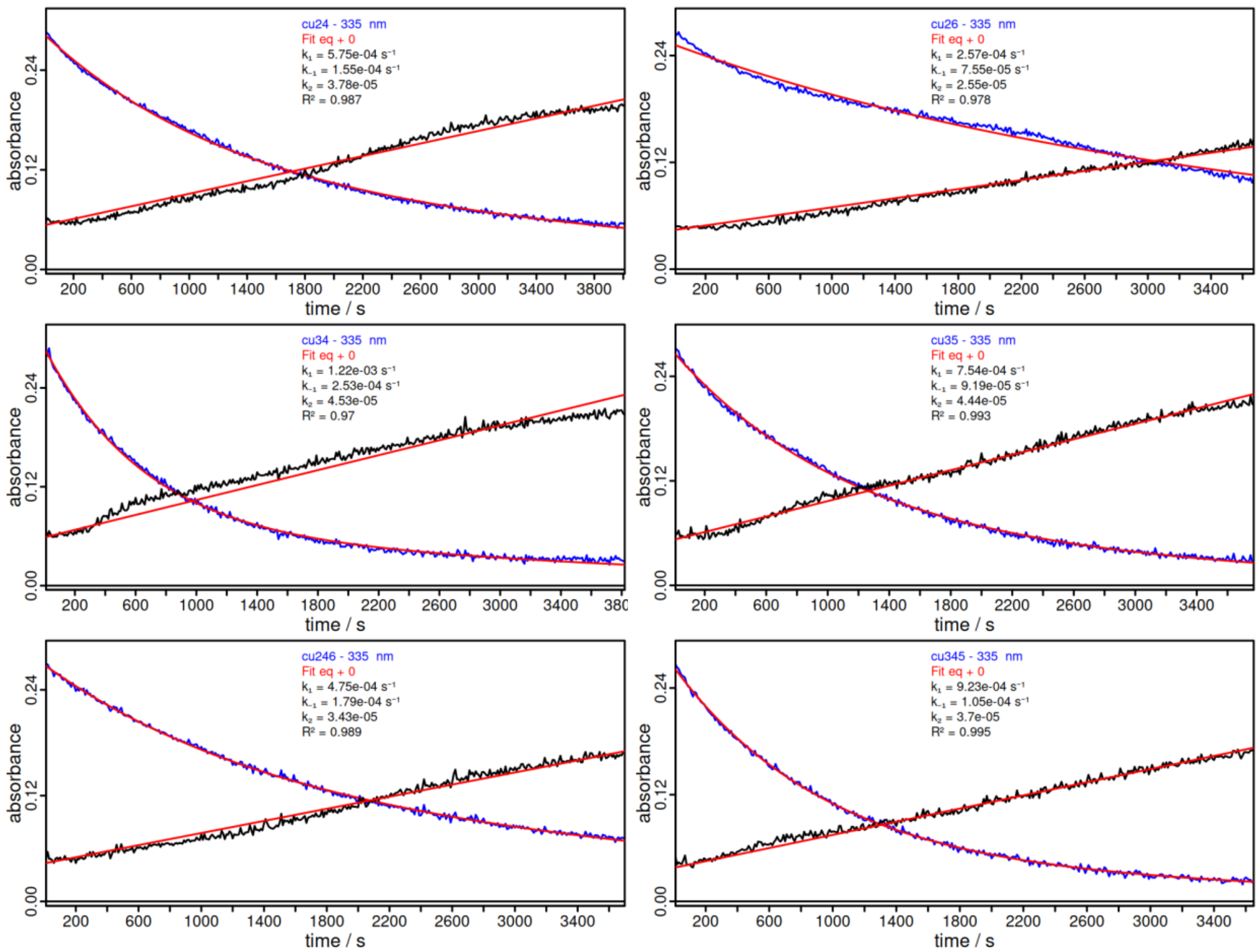

Figure S10.3. Kinetics of the photocatalytic oxygenation of 2,5-diphenylfuran (DPF) at 335 and 255 nm: **Cu24** (top left), **Cu26** (top right), **Cu34** (middle left), **Cu35** (middle right), **Cu246** (bottom left) and **Cu345** (bottom right). The red-lines are the respective fits of the general reaction scheme $A \rightleftharpoons B \rightarrow C$, with $k_1$belongs to $A \rightarrow B$, k_{-1}belongs to $B \rightarrow A$ with first order kinetics and $k_2$belongs to $B \rightarrow C$ as 0. order.

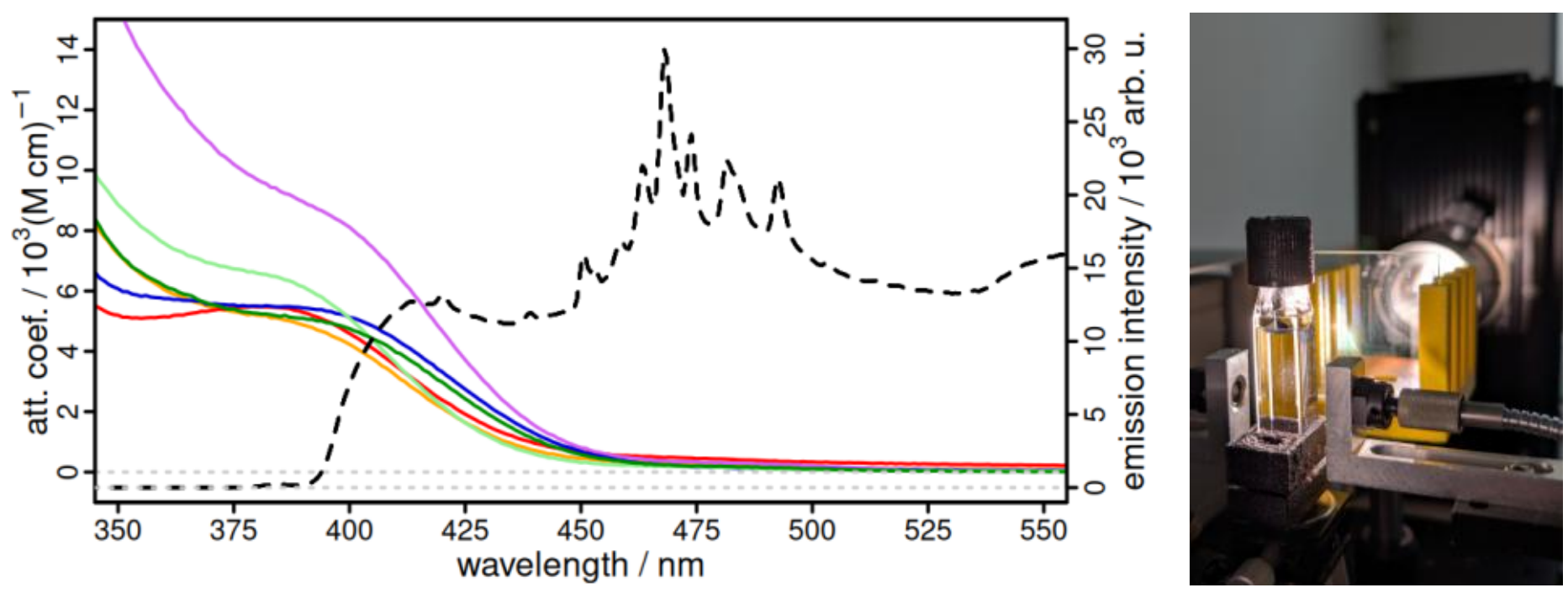

Figure S10.4. Left: Overlap of the attenuation at room temperature in the MLCT-region of **Cu24** (orange), **Cu26** (red), **Cu34** (purple), **Cu35** (blue), **Cu246** (light green) and **Cu345** (dark green) in acetonitrile solution with the emission spectrum of the xenon lamp (0.850 W at the cuvette, distance: 16 cm). The comparison shows the highest absorption in the emission region for **Cu34**, followed by **Cu35** and **Cu345**, which exhibit very similar behavior. **Cu24**, **Cu26**, and **Cu246** show lower absorption and are approximately comparable to one another. Right: Photograph of the experimental setup.

Photocatalytic Reductive Debromination

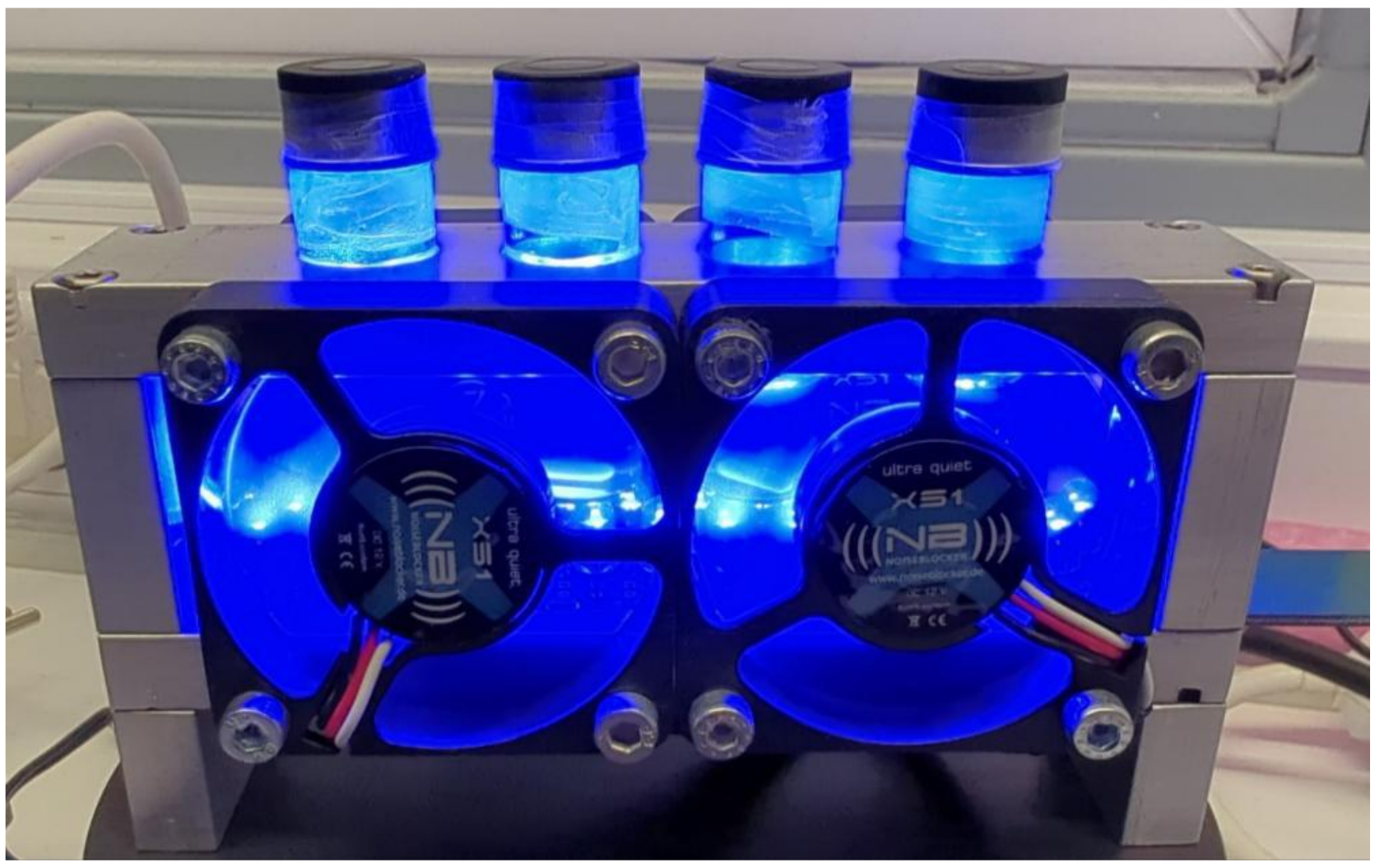

Figure S10.5. Photograph of the photoreactor setup.

The photoreactor used for the reductive debromination experiments (Figure S10.4) accommodates four vials with a diameter of 1.5 cm, positioned 0.7 cm apart. To dissipate the generated heat, the reactor is cooled by four fans (two on each side), ensuring that temperature fluctuations remain within ±1 °C over a period of 5 h. The vials are placed directly on the LED (λ = 460 nm) used for irradiation. The emission spectrum of the LED is shown in Figure S10.6.

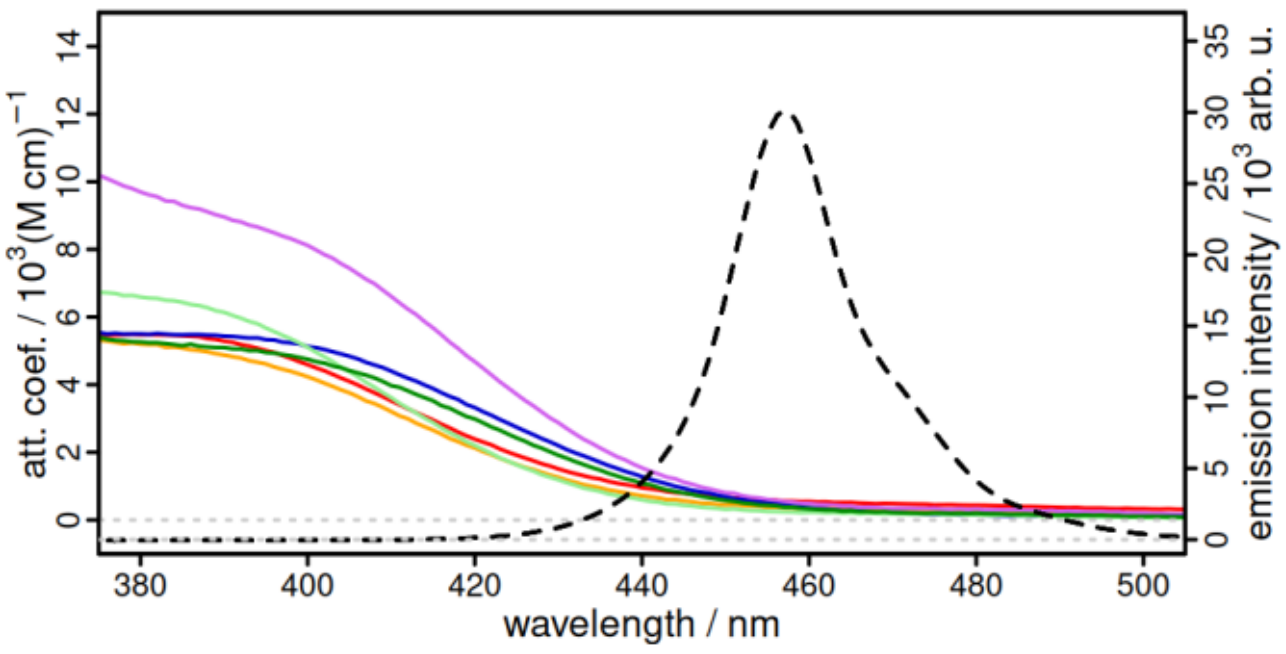

Figure S10.6. Overlap of the attenuation of **Cu24** (orange), **Cu26** (red), **Cu34** (purple), **Cu35** (blue), **Cu345** (dark green), and **Cu246** (light green) with the emission spectrum of the blue LED (460 nm) used for irradiation (black solid line).

Table S10.1. Conversions and yields of the reductive debromination of 2-bromobenzoate (E1) after 10 min of irradiation.

Complex	Cu24	Cu26	Cu34	Cu35	Cu246	Cu345
Conversion [%]	73.3 ± 1.1	74.9 ± 0.7	89.9 ± 0.1	92.8 ± 0.3	61.5 ± 1.7	81.7 ± 0.6
Yield [%]	64.3 ± 1.2	62.0 ± 0.0	78.0 ± 1.7	72.5 ± 0.8	51.5 ± 0.1	72.6 ± 0.9

Table S10.2. Conversions and yields of the reductive debromination of 4-bromobenzophenone (E2) after 15 min of irradiation.

Complex	Cu24	Cu26	Cu34	Cu35	Cu246	Cu345
Conversion [%]	71.5 ± 0.7	66.4 ± 1.3	81.4 ± 0.2	79.8 ± 0.3	65.1 ± 0.5	74.7 ± 1.8
Yield [%]	69.1 ± 0.0	62.4 ± 0.9	76.3 ± 0.6	76.2 ± 0.1	61.7 ± 0.4	72.4 ± 1.7

Table S10.3. Conversions and yields of the reductive debromination of 2-bromoacetanilide (E3) after 5 h of irradiation.

Complex	Cu24	Cu26	Cu34	Cu35	Cu246	Cu345
Conversion [%]	51.1 ± 1.7	55.8 ± 1.2	42.8 ± 0.0	33.5 ± 0.4	54.6 ± 0.0	37.4 ± 0.9
Yield [%]	51.1 ± 1.7	55.8 ± 1.2	42.8 ± 0.0	33.5 ± 0.4	54.6 ± 0.0	37.4 ± 0.9

Stern-Volmer-Quenching

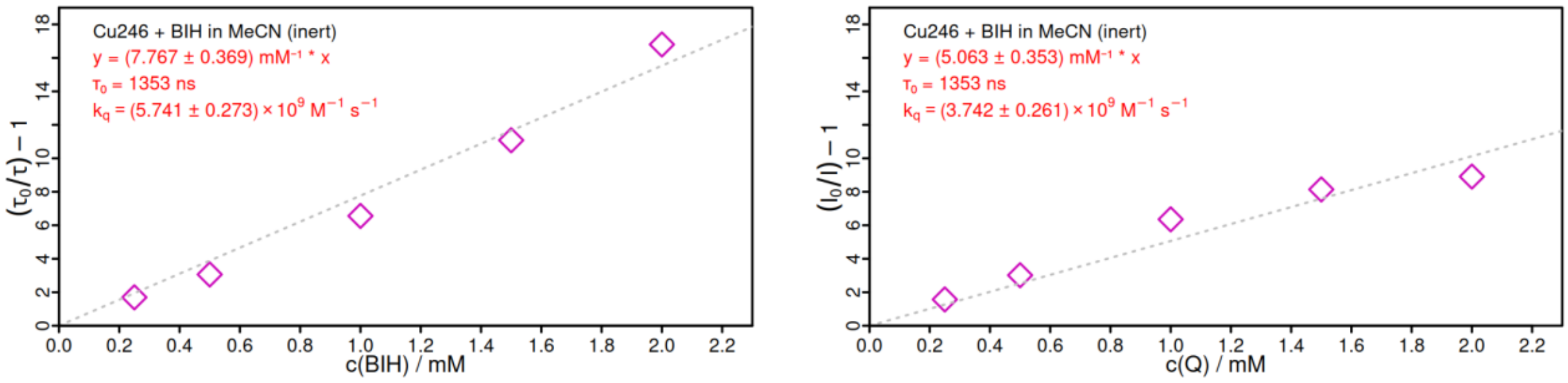

Figure S10.7. Stern-Volmer plot of the emission lifetime (left) and of the emission intensity (right) for **Cu246** with BIH in inert acetonitrile at room temperature. The kinetic constants for the reductive quenching (k_q) are determined *via* the slope of the Stern-Volmer plot are k_q = (5.741 ± 0.273) · 10^9 M^{-1} s^{-1} (lifetime) and k_q = (3.742 ± 0.261) · 10^9 M^{-1} s^{-1} (intensity).

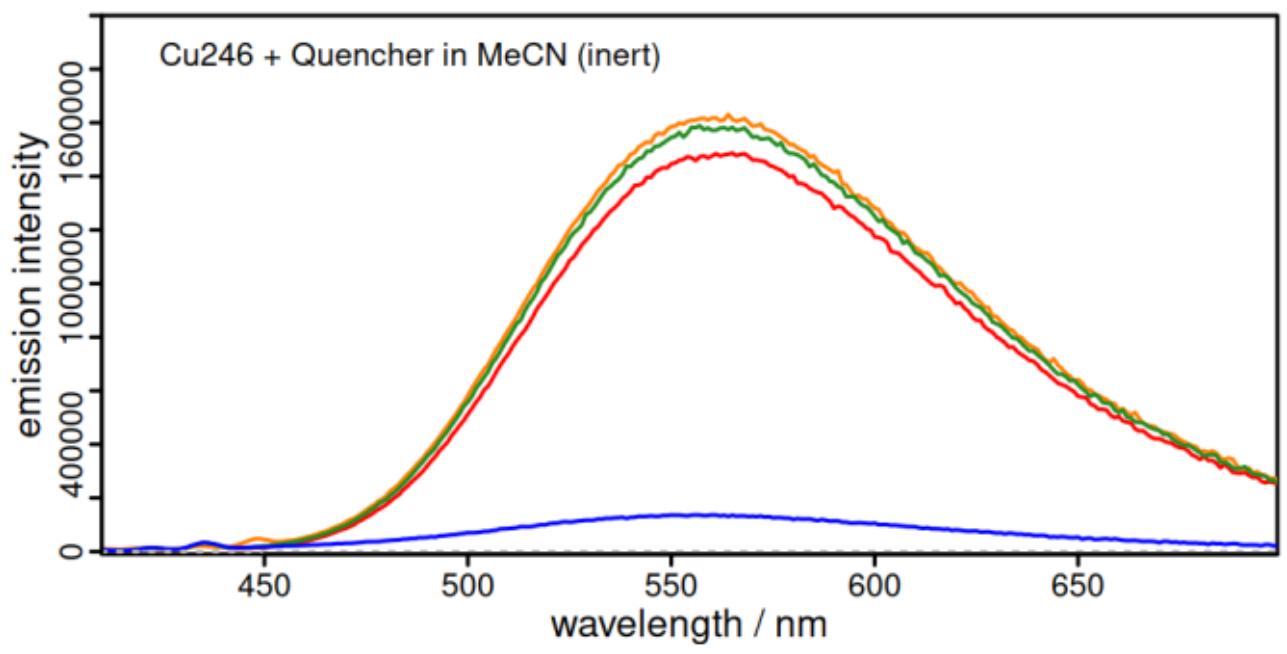

Figure S10.8. Steady-state emission spectra of **Cu246** in inert acetonitrile at room temperature are displayed for the following conditions: **Cu246** in the absence of a quencher (red line), in the presence of E2 (orange line, 2 mM), in the presence of E3 (green line, 2 mM), and in the presence of BIH (blue line, 2 mM). All other experimental conditions were identical.

Table S10.4. Emission lifetimes of **Cu246** in inert acetonitrile at room temperature in the absence and presence of the different Quenchers E2, E3 and BIH ([Q] = 2mM).

**Quencher**	**-**	**E2**	**E3**	**BIH**
**Emission lifetime**	1353 ± 13 ns	1396 ± 17 ns	1426 ± 13 ns	76 ± 4 ns

The received results underline, that effective reductive quenching *via* BIH is the dominant quenching pathway (k_q in the order of 10^9 M^{-1} s^{-1}), whereas no significant oxidative quenching with the substrates E2 or E3 can be observed.

Photocatalytic Aza-Henry Reaction

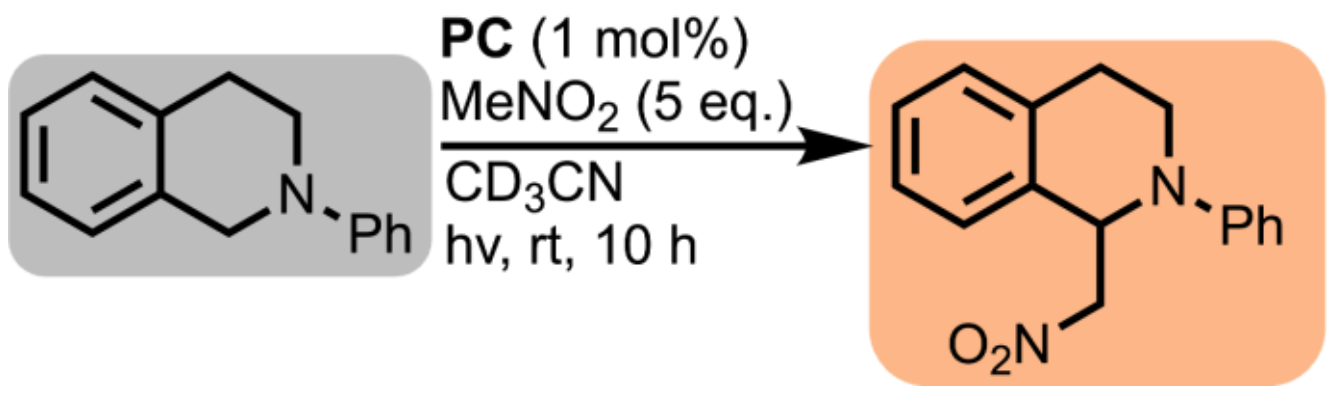

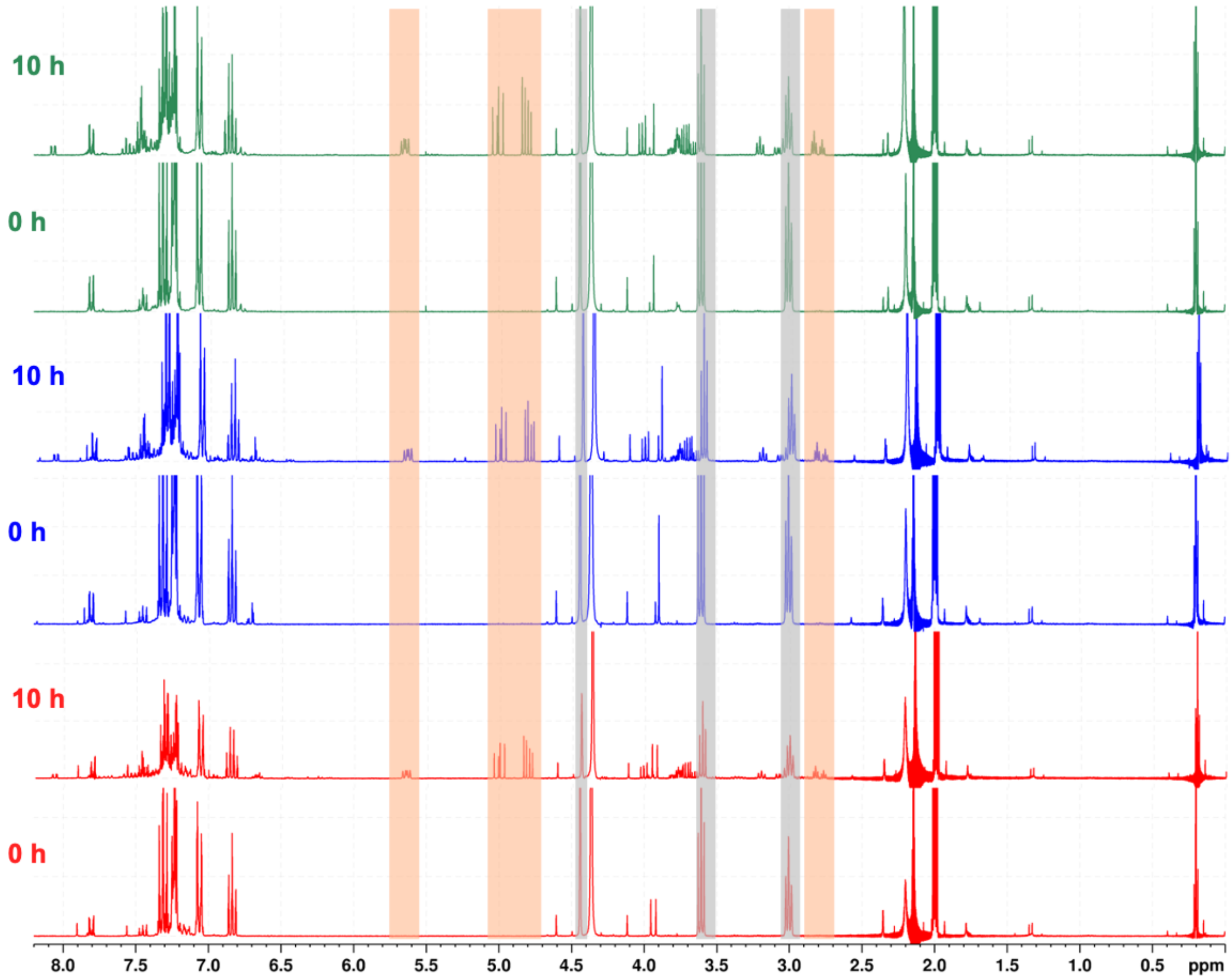

Figure S10.9. ^{1}H NMR spectra (400 MHz, 25 °C) of the corresponding Cu(I) complexes **Cu24** (green, top), **Cu35** (blue, middle), and **Cu34** (red, bottom) recorded before (0 hours) and after irradiation (10 hours). Spectra were referenced using hexamethylcyclotrisiloxane (D3, δ = 0.2 ppm) as the internal standard. The grey-shaded region corresponds to the starting material THIQ, whereas the ivory-shaded regions correspond to the Aza-Henry product.

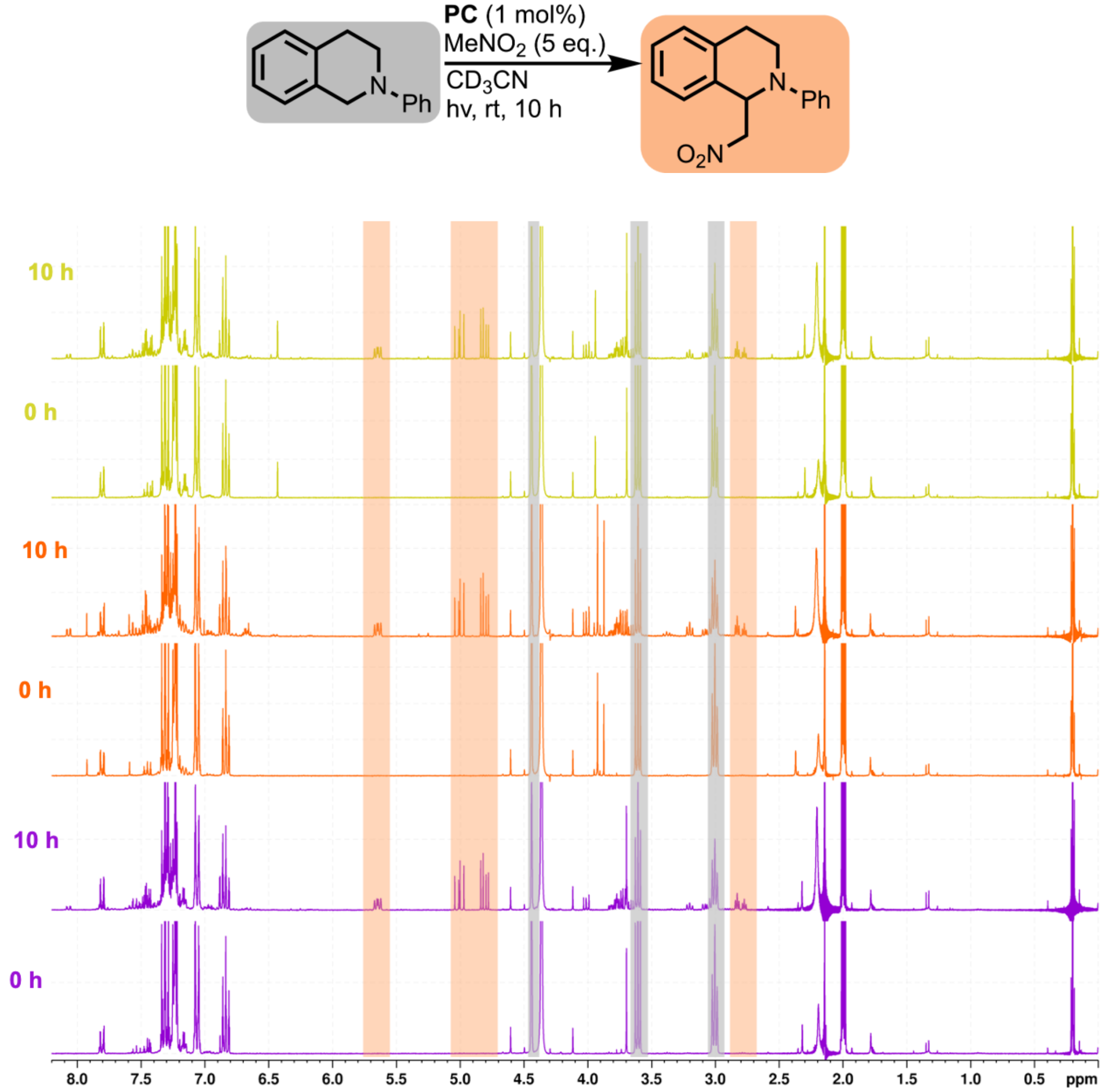

Figure S10.10. ^{1}H NMR spectra (400 MHz, 25 °C) of the corresponding Cu(I) complexes **Cu246** (yellow, top), **Cu345** (orange, middle), and **Cu26** (purple, bottom) recorded before (0 hours) and after irradiation (10 hours). Spectra were referenced using hexamethylcyclotrisiloxane (D3, δ = 0.2 ppm) as the internal standard. The grey-shaded region corresponds to the starting material THIQ, while the ivory-shaded regions correspond to the Aza-Henry product.

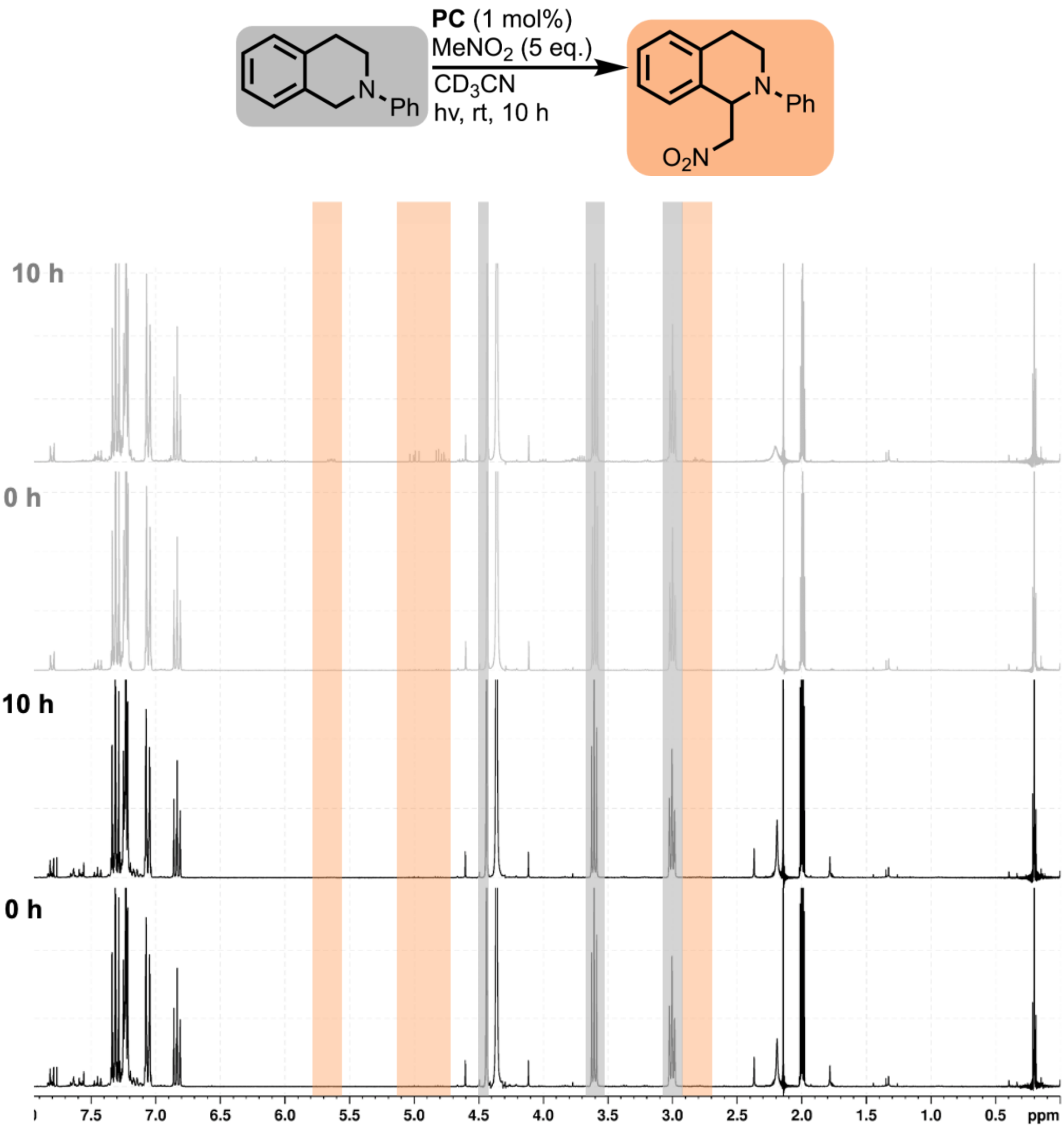

Figure S10.11. ^{1}H NMR spectra (400 MHz, 25 °C) in the absence of photocatalyst (grey, top) and in the absence of irradiation (black, bottom) before (0 hours) and after (10 hours). Spectra were referenced using hexamethylcyclotrisiloxane (D3, δ = 0.2 ppm) as the internal standard. The grey-shaded region corresponds to the starting material THIQ, while the ivory-shaded regions correspond to the Aza-Henry product.

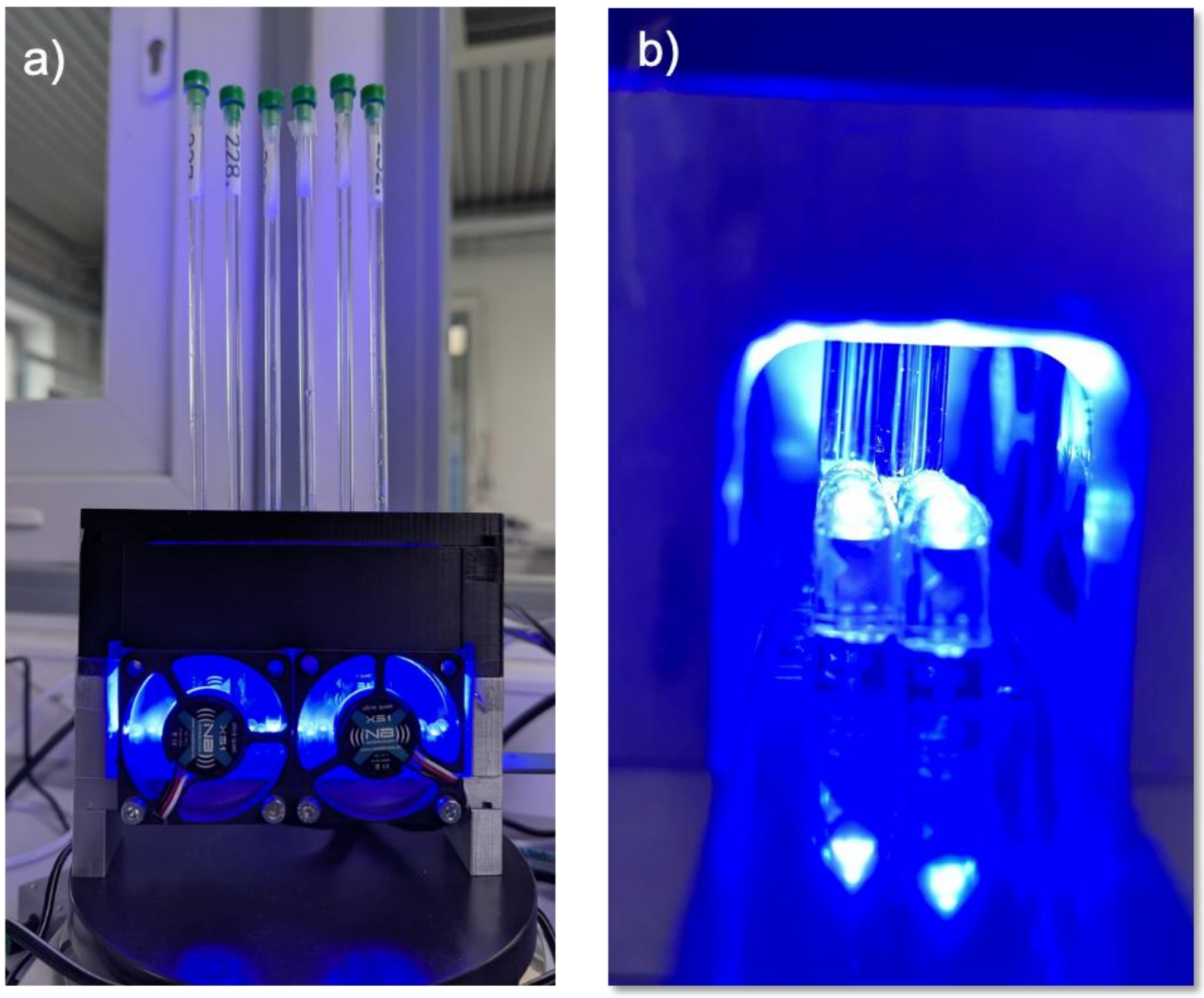

Figure S10.12. Experimental setup for the Aza-Henry reaction using a photoreactor equipped with air cooling and a top holder for NMR tubes (a). Each tube is positioned directly above the LEDs to ensure uniform irradiation conditions (b). The same LED as in the reductive dehalogenation experiments was employed, with the emission spectrum shown in Figure S10.6.

11 References

1 D. Stalke, "Cryo crystal structure determination and application to intermediates," *Chemical Society Reviews* 27, no. 3 (1998): 171. https://doi.org/10.1039/a827171z.

2 CrysAlisPro, Rigaku Oxford Diffraction, Versions 1.171.43.104a–1.171.44.144a, 2024–2026.

3 a) G. M. Sheldrick, "*SHELXT* – Integrated space-group and crystal-structure determination," *Acta Crystallographica Section A Foundations and Advances* 71, no. 1 (2015): 3–8. https://doi.org/10.1107/S2053273314026370G; G. M. Sheldrick, "Crystal structure refinement with *SHELXL*," *Acta Crystallographica Section C Structural Chemistry* 71, no. 1 (2015): 3–8. https://doi.org/10.1107/S2053229614024218; c) G. M. Sheldrick, "A short history of *SHELX*," *Acta Crystallographica Section A Foundations of Crystallography* 64, no. 1 (2008): 112–122. https://doi.org/10.1107/S0108767307043930.

4 a) O. V. Dolomanov, L. J. Bourhis, R. J. Gildea, J. A. K. Howard, and H. Puschmann, "*OLEX2*: a complete structure solution, refinement and analysis program," *Journal of Applied Crystallography* 42, no. 2 (2009): 339–341. https://doi.org/10.1107/S0021889808042726; b) A. L. Spek, "Structure validation in chemical crystallography," *Acta Crystallographica Section D Biological Crystallography* 65, no. 2 (2009): 148–155. https://doi.org/10.1107/S090744490804362X; c) C. F. Macrae, I. Sovago, S. J. Cottrell, P. T. A. Galek, P. McCabe, E. Pidcock, M. Platings, G. P. Shields, J. S. Stevens, M. Towler, and P. A. Wood, "*Mercury 4*: from visualization to analysis, design and prediction," *Journal of Applied Crystallography* 53, no. 1 (2020): 226–235. https://doi.org/10.1107/S1600576719014092.

5 Diamond – Crystal and Molecular Structure Visualization, Crystal Impact - H. Putz and K. Brandenburg GbR, Bonn, Germany, 2024.

6 Gaussian 16, Revision A.03, M. J. Frisch, G. W. Trucks, H. B. Schlegel, G. E. Scuseria, M. A. Robb, J. R. Cheeseman, G. Scalmani, V. Barone, G. A. Petersson, H. Nakatsuji, X. Li, M. Caricato, A. V. Marenich, J. Bloino, B. G. Janesko, R. Gomperts, B. Mennucci, H. P. Hratchian, J. V. Ortiz, A. F. Izmaylov, J. L. Sonnenberg, D. Williams-Young, F. Ding, F. Lipparini, F. Egidi, J. Goings, B. Peng, A. Petrone, T. Henderson, D. Ranasinghe, V. G. Zakrzewski, J. Gao, N. Rega, G. Zheng, W. Liang, M. Hada, M. Ehara, K. Toyota, R. Fukuda, J. Hasegawa, M. Ishida, T. Nakajima, Y. Honda, O. Kitao, H. Nakai, T. Vreven, K. Throssell, J. A. Montgomery, Jr., J. E. Peralta, F. Ogliaro, M. J. Bearpark, J. J. Heyd, E. N. Brothers, K. N. Kudin, V. N. Staroverov, T. A. Keith, R. Kobayashi, J. Normand, K. Raghavachari, A. P. Rendell, J. C. Burant, S. S. Iyengar, J. Tomasi, M. Cossi, J. M. Millam, M. Klene, C. Adamo, R. Cammi, J. W. Ochterski, R. L. Martin, K. Morokuma, O. Farkas, J. B. Foresman, and D. J. Fox, Gaussian, Inc., Wallingford CT, 2016.

7 TURBOMOLE V7.8 2024, a development of University of Karlsruhe and Forschungszentrum Karlsruhe GmbH, 1989-2007, TURBOMOLE GmbH, since 2007; available from https://www.turbomole.org.

8 F. Doettinger, C. Kleeberg, C. Queffélec, S. Tschierlei, Y. Pellegrin, and M. Karnahl, "Rich or poor: the impact of electron donation and withdrawal on the photophysical and photocatalytic properties of copper(i) complexes," Catalysis Science & Technology 13, no. 14 (2023): 4092–4106. https://doi.org/10.1039/D3CY00132F.

9 J. Godard, F. Brégier, P. Arnoux, B. Myrzakhmetov, Y. Champavier, C. Frochot, and V. Sol, "New Phenalenone Derivatives: Synthesis and Evaluation of Their Singlet Oxygen Quantum Yield," *ACS Omega* 5, no. 43 (2020): 28264–28272. https://doi.org/10.1021/acsomega.0c04172.

10 a) S. Lv, Q. Li, J.-W. Sang, Y. Zhang, J. Wang, and W.-D. Zhang, "Uranyl nitrate as a recyclable homogeneous photocatalyst for selective cross-coupling of *N* -substituted amines and indoles," *RSC Advances* 13, no. 18 (2023): 11929–11937. https://doi.org/10.1039/D3RA01037F; b) W. Liang, T. Zhang, Y. Liu, Y. Huang, Z. Liu, Y. Liu, B. Yang, X. Zhou, and J. Zhang, "Polydimethylsiloxane Sponge-Supported Nanometer Gold: Highly Efficient Recyclable Catalyst for Cross-Dehydrogenative Coupling in Water," *ChemSusChem* 11, no. 20 (2018): 3586–3590. https://doi.org/10.1002/cssc.201801180.

11 F. Doettinger, Y. Yang, M. Karnahl, and S. Tschierlei, "Bichromophoric Photosensitizers: How and Where to Attach Pyrene Moieties to Phenanthroline to Generate Copper(I) Complexes," *Inorganic Chemistry* 62, no. 21 (2023): 8166–8178. https://doi.org/10.1021/acs.inorgchem.3c00482.

12 N. Chen, L. Xia, A. J. J. Lennox, Y. Sun, H. Chen, H. Jin, H. Junge, Q. Wu, J. Jia, M. Beller, and S. Luo, "Structure-Activated Copper Photosensitisers for Photocatalytic Water Reduction," *Chemistry – A European Journal* 23, no. 15 (2017): 3631–3636. https://doi.org/10.1002/chem.201602598.

13 a) J. R. Lakowicz, *Principles of Fluorescence Spectroscopy*, 3rd ed.; Springer: Boston, MA, 2006. ISBN 978-0-387-31278-1. https://doi.org/10.1007/978-0-387-46312-4; b) D. Rehm and A. Weller, "Kinetics of Fluorescence Quenching by Electron and H-Atom Transfer," *Israel Journal of Chemistry* 8, no. 2 (1970): 259–271. https://doi.org/10.1002/ijch.197000029.

14 L. Burmeister, F. Doettinger, K. J. Haseloff, C. Kleeberg, M. Boujtita, S. Pascal, F. Odobel, S. Tschierlei, Y. Pellegrin, M. Karnahl, "Dye-Sensitized Solar Cells Based on Cu(I) Complexes Containing Catechol Anchor Groups That Operate with Aqueous Electrolytes," *JACS Au* 5, no. 8 (2025), 3960-3973. https://doi.org/10.1021/jacsau.5c00601.
